# A Hierarchy of Ligands Controls Formation and Reaction of Aryl Radicals in Pd-Catalyzed Ground-State Base-Promoted Coupling Reactions

**DOI:** 10.1021/jacs.3c05470

**Published:** 2023-09-15

**Authors:** Kenneth F. Clark, Seb Tyerman, Laura Evans, Craig M. Robertson, David J. Nelson, Alan R. Kennedy, John A. Murphy

**Affiliations:** † Department of Pure and Applied Chemistry, University of Strathclyde, 295 Cathedral Street, Glasgow G1 1XL, U.K.; ‡ Medicinal Chemistry, Research and Early Development, Oncology R&D, AstraZeneca, Cambridge CB10 1XL, U.K.; § GSK Medicines Research Centre, Gunnels Wood Road, Stevenage SG1 2NY, Herts, U.K.

## Abstract

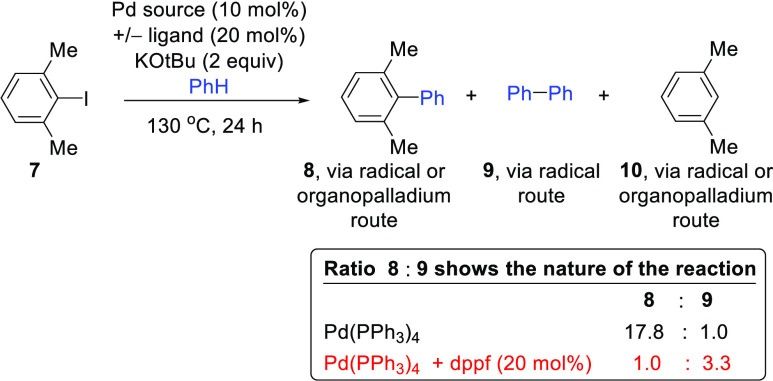

Palladium salts and complexes were tested separately and in the presence of added ligands as potential sources of aryl radicals in ground-state coupling reactions of aryl halide with arenes under basic conditions (KO*t*Bu). Our recently developed assay for aryl radicals was employed to test for aryl radicals. In this assay, aryl radicals derived from the test substrate, 1-iodo-2,6-dimethylbenzene **7**, undergo base-promoted homolytic aromatic substitution (BHAS) with benzene to produce 2,6-dimethylbiphenyl **8** and biphenyl **9** in an approximately 1:4 ratio as well as *m*-xylene **10.** The biphenyl arises from a diagnostic radical transfer reaction with the solvent benzene. Using substrate **7** with a range of Pd sources as potential initiators led to formation of **8**, **9**, and **10** in varying amounts. However, when any one of a range of diphosphinoferrocenes (e.g., dppf or dippf) or BINAP or the monophosphine, diphenylphosphinoferrocene, was added as a ligand to Pd(OAc)_2_, the ratio of [2,6-dimethylbiphenyl **8**: biphenyl **9**] moved decisively to that expected from the BHAS (radical) pathway. Further studies were conducted with dppf. When dppf was added to each of the other Pd sources, the ratio of coupled products was also diverted to that expected for radical BHAS chemistry. Deuterium isotope studies and radical trap experiments provide strong additional support for the involvement of aryl radicals. Accordingly, under these ground-state conditions, palladium sources, in the presence of defined ligands, convert aryl iodides to aryl radicals. A rationale is proposed for these observations.

## Introduction

Organometallic chemistry affords some of the most useful reactions in synthesis today. Recently, a lot of interest has been shown in the possible involvement of radicals in reactions using palladium or nickel salts and complexes.^1−4^ An early comparison of the reactions of aliphatic α-iodoesters (i) with hexabutylditin and (ii) with Pd(0) reagents by Curran et al.^5,6^ identified the formation of alkyl radicals in both cases. Reports of the formation of radicals from reactions involving palladium chemistry have increased markedly in recent years, with notable examples from Ryu, Alexanian, and Gevorgyan, among others.^7−13^ Many of these relate to aryl halide substrates. In particular, Gevorgyan has pioneered the use of Pd sources, usually under photoactivated conditions, to give rise to “hybrid Pd-radicals”. This may mean that C–Pd(II) bonds can undergo reversible cleavage under the reaction conditions to form carbon radicals and Pd(I) intermediates. Harnessing this very interesting duality affords significant opportunities for synthetic planning.^14−16^ However, specialized ligands are often employed, and the use of visible light brings with it additional mechanistic questions relating to photoactivation that could lead to electron transfer, energy transfer, or photochemistry of intermediates. We are interested in understanding more generally whether aryl radicals can be formed from the reaction between substrates and different Pd sources using ground-state conditions. Recently, we have studied coupling reactions of aryl radicals that were generated from organic initiators, leading to a novel and specific assay for aryl radicals.^17^ In this paper, we now apply this assay to ground-state coupling reactions triggered by palladium salts and complexes.

**Scheme 1:**
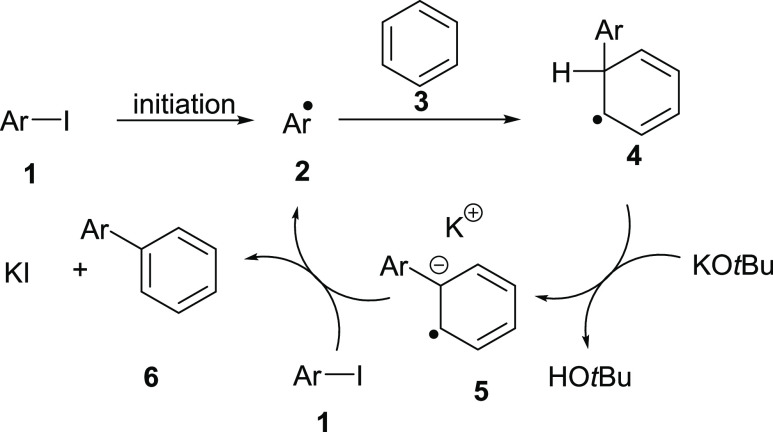
BHAS Coupling

Radical intermediates are central to the mechanism of base-promoted homolytic aromatic substitution (BHAS),^18−23^ where an aryl radical adds to an arene in the presence of KO*t*Bu to form a biaryl. In the accepted mechanism, an aryl radical **2** adds to arene **3**, forming arylcyclohexadienyl radical **4**, which then undergoes easy deprotonation by *tert*-butoxide. The resulting radical anion **5** then transfers an electron to another molecule of the haloarene **1**, forming a new aryl radical **2** and the observed coupled product **6**, starting a chain reaction (Scheme 1).^22^ The reactions are usually initiated by electron transfer when one of a wide range of organic additives reacts with KO*t*Bu in situ to form an organic electron donor.^23−25^

## Results and Discussion^26^

**Scheme 2:**
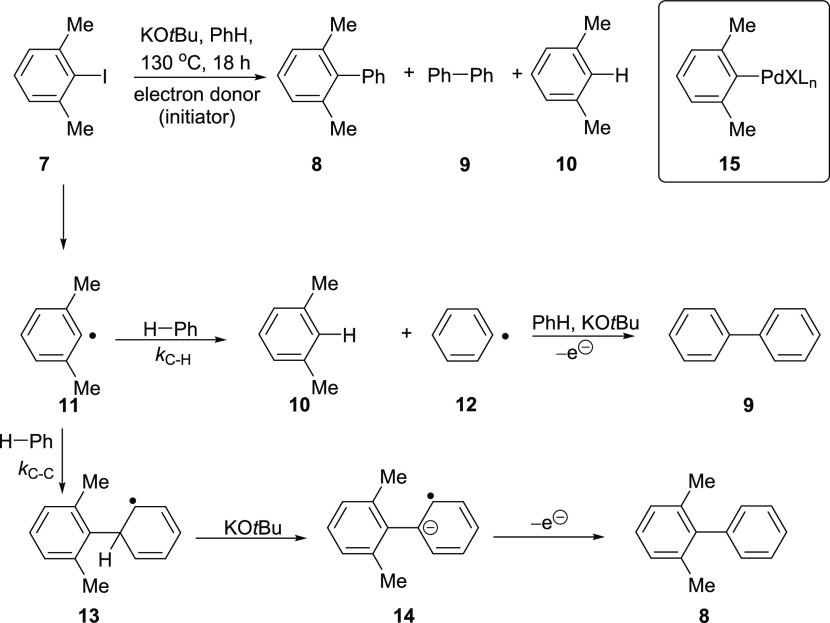
Anomalous BHAS Coupling of Substrate **7**

Our assay for aryl radicals arises from the anomalous BHAS chemistry of the hindered substrate 2,6-dimethyliodobenzene **7**.^27^ Reaction of 2,6-dimethyliodobenzene **7** with benzene and KO*t*Bu in the presence of an initiator provides a characteristic ratio (ca. 1:4) of 2,6-dimethylbiphenyl **8**: biphenyl **9** regardless of the initiator. The formation of **9** as the principal coupling product results from the sterically hindered nature of the 2,6-dimethylphenyl radical **11**, slowing the expected attack on the π-system of benzene that would lead to the formation of the normal BHAS product **8**. Radical **11** can alternatively undertake hydrogen atom abstraction from benzene, forming the phenyl radical **12** together with *m-*xylene **10**. (The xylyl radicals can also abstract hydrogen from other sources,^27^ accounting for the much greater yields of xylene than of coupled products.) This phenyl radical **12** can then carry out BHAS coupling with benzene, producing biphenyl **9** (Scheme 2). In our development of the assay, the ratio of the yields of the two coupled products **8** and **9** was effectively the same (ca. 1:4) regardless of the initiation source, likely reflecting control by the ratio of the rate constants for the two possible reactions of radical **11** with benzene, i.e., *k*_C–H_ for hydrogen atom abstraction from benzene and *k*_C–C_ for addition to benzene.^27^

Taking 2,6-dimethyliodobenzene, **7**, as the substrate, palladium sources (10 mol %) were examined (Table 1) in benzene, with KO*t*Bu (2 equiv) as the base, and in the absence of any other additives under the conditions used in Scheme 2. All of the Pd sources (entries 2–10) engaged substrate **7** and afforded increased yields of **8** when compared to the blank reaction (entry 1). Interestingly, biphenyl **9** was also formed in all cases in noteworthy amounts, although the ratios of **8**:**9** varied widely, and with the important exception of entries 5 and 6, using Pd(dppf)_2_ and Pd(BINAP)_2_, none of the entries showed the ca. 1:4 ratio that would be the hallmark of exclusive radical BHAS chemistry.

**Table 1:**
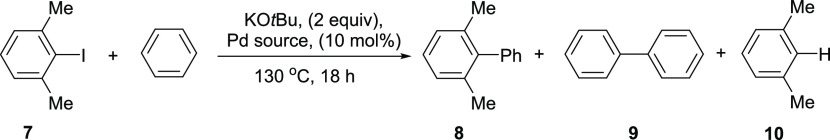
Reaction of 2,6-Dimethyliodobenzene with KO*t*Bu in Benzene

entry	Pd source	2-iodo-*m*-xylene **7**	2,6-dimethylbiphenyl **8**	biphenyl **9**	*m*-xylene **10**	ratio 8:9
1	none	93%	0.3%	0.5%	1.5%	1.0:1.7
2	Pd(OAc)_2_	6.3%	15.4%	5.5%	36.4%	2.8:1.0
3	Pd(PPh_3_)_4_	2.7%	30.3%	1.7%	25.8%	17.8:1.0
4	Pd[P(*o*-tol)_3_]_2_	19.4%	2.7%	3.7%	49.4%	1.0:1.4
5	Pd(dppf)_2_	0.3%	2.7%	11.2%	32.2%	1.0:4.1
6	Pd(BINAP)_2_	4.9%	5.2%	16.7%	47.9%	1.0:3.2
7	Pd(PPh_3_)_2_Cl_2_	1.7%	14.9%	4.5%	46.7%	3.3:1.0
8	PdCl_2_	86.8%	0.6%	1.0%	3.2%	1.0:1.7
9	Pd(TFA)_2_	10.3%	6.7%	7.1%	45.1%	1.0:1.0
10	Pd(dppf)Cl_2_	0%	7.0%	8.0%	42.5%	1.0:1.1

**Scheme 3:**
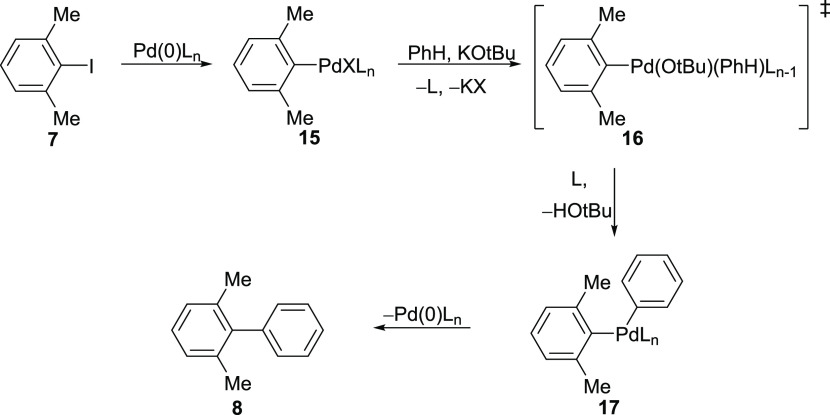
Organopalladium Coupling

The coupled product **8** potentially arises from coupling of the arylpalladium derivative **15** (inset in Scheme 2; see also Scheme 3 for discussion) with benzene. Competing with this organopalladium-mediated coupling mechanism, products **8** and **9** can alternatively both arise (ratio of ca. 1:4) by a BHAS coupling undertaken by aryl radicals. Thus, at this stage, Table 1 indicates that entries 5 and 6 involving Pd(dppf)_2_ and Pd(BINAP)_2_ show all of the characteristics of BHAS reactions, with biphenyl **9** being formed in much greater quantities than dimethylbiphenyl **8**. In contrast, a minor amount of aryl radical formation could be occurring for all other Pd sources, and indeed for some sources, e.g., Pd(PPh_3_)_4_ (where the ratio of **8**:**9** was 17.8:1), this was a very small fraction of the total reactivity.

Clearly, the Pd source impacts the product distribution. A ligand screen of different phosphines was undertaken. Pd(OAc)_2_ was adopted as the palladium source for this study (Table 2). The results were intriguing and showed that the reactions fell into three classes: (i) phosphinoferrocene ligands as well as BINAP (entries 2–9), which gave much more biphenyl **9** than 2,6-dimethylbiphenyl **8**, just as expected for a radical-based BHAS pathway;^7,23^ (ii) ligands like PCy_3_ and PPh_3_, which afford much more 2,6-dimethylbiphenyl **8** than biphenyl **9** (entries 10*–*14); PCy_3_ itself shows an impressive ratio of 2,6-dimethylbiphenyl **8**: biphenyl **9** of 46:1; and (iii) ligands that produce roughly equal amounts of **8** and **9** (entries 15–18). Within entries 2–9, most ligands are ferrocenes, which makes them the center of interest. Considering whether steric and/or electronic effects could be at play, we noted the range of bite angles associated with the diphosphine ligands in Table 2 that support BHAS chemistry including BINAP 93°, dppf 99°, and dippf 103° (entries 9, 3, and 4), while those that do not support BHAS chemistry^28^ in Pd-catalyzed reactions ranged from dppe, 86° and dcpe, 87° (entries 14 and 12) to DPEPhos 104° and XantPhos 108° (entries 15 and 16). Three monophosphines PCy_3_, P*t*Bu_3_, and PPh_3_ with a range of reported cone angles^28^ (entries 10, 11, and 13) did not support BHAS chemistry and so to probe whether this was a property of monophosphines that could be overcome by a ferrocenylmonophosphine, we examined FcPPh_2_. This ligand indeed promoted the radical behavior (entry 8) and influenced us to focus on the electronic properties of these ligands.

**Table 2:**
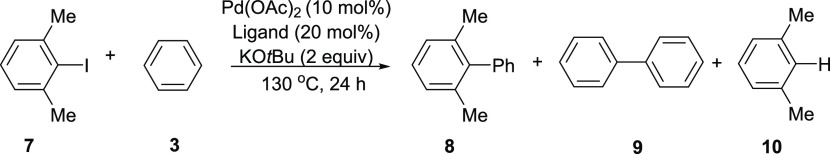
Effect of Addition of Different Ligands on the Pd(OAc)_2_-Induced Coupling Reaction

entry	ligand	2-iodo-*m*-xylene **7**	2,6-dimethyl-biphenyl **8**	biphenyl **9**	*m*-xylene **10**	ratio 8:9
1		6.3%	15.4%	5.5%	36.4%	2.8:1.0
radical formation dominant						
2	Dtbdppf	4.7%	2.5%	12.8%	42.9%	1.0:5.1
3	Dppf	0%	3.1%	12.4%	34.6%	1.0:4.0
4	Dippf	0.2%	2.0%	8.8%	41.6%	1.0:4.4
5	D(tol)ppf	0%	3.1%	13.1%	43.2%	1.0:4.2
6	Dppf[(CF_3_)_2_]_2_	0.8%	2.4%	9.9%	24.1%	1.0:4.1
7	Fc(PCy_2_)_2_	0.2%	1.4%	5.0%	24.9%	1.0:3.6
8	FcPPh_2_	0%	2.3%	6.6%	42.8%	1.0:2.9
9	BINAP	4.1%	3.7%	10.6%	34.8%	1.0:2.9
organopalladium mechanism dominant						
10	PCy_3_	6.9%	27.7%	0.6%	8.2%	46.0:1.0
11	P*t*Bu_3_^a^	0%	6.9%	0.8%	14.5%	8.6:1.0
12	Dcpe	0.9%	11.0%	1.9%	28.6%	5.8:1.0
13	PPh_3_	0%	21.3%	4.0%	41.6%	5.3:1.0
14	Dppe	0%	12.9%	5.1%	22.2%	2.5:1.0
both mechanisms similarly promoted						
15	DPEPhos	16.9%	3.3%	3.8%	17.7%	1.0:1.2
16	XantPhos	9.3%	6.0%	6.6%	25.8%	1.0:1.1
17	IMes.HCl	3.2%	6.1%	7.8%	54.3%	1.0:1.3
18	XPhos	8.9%	8.8%	7.3%	45.5%	1.2:1.0

Dtbdppf-1-diphenylphosphino-1′-(di-*tert*-butylphosphino)ferrocene, Dppf-1,1′-ferrocenediyl-bis(diphenylphosphine), Dippf-1,1′-bis(diisopropylphosphino)ferrocene, D(tol)ppf-1,1′-ferrocenediyl-bis(*p*-tolylphosphino)ferrocene, Dppf(CF_3_)_2_-1,1′-ferrocenediyl-bis(*p*-trifluoromethylphenylphosphino)ferrocene, Fc(PCy_2_)_2_-1,1′-bis(dicyclohexylphosphino)ferrocene, FcPPh_2_-diphenylphosphinoferrocene, BINAP-(±)-2,2′-Bis(diphenylphosphino)-1,1′-binaphthalene, PCy_3_-tricyclohexylphosphine, P*t*Bu_3_-tri*tert*-butylphosphine, Dcpe-ethylenebis(dicyclohexylphosphine), PPh_3_-triphenylphosphine, Dppe-ethylenebis(diphenylphosphine), DPEPhos-(oxybis(2,1-phenylene))bis(diphenylphosphane), XantPhos-4,5-bis(diphenylphosphino)-9,9-dimethylxanthene, IMes. HCl-1,3-Dimesitylimidazolium chloride, XPhos-dicyclohexyl(2′,4′,6′-triisopropyl-[1,1′-biphenyl]-2-yl)phosphane. ^a^ This experiment afforded 2-*tert*-butoxy-1,3-dimethylbenzene as a reaction product.

Given that the phosphinoferrocene ligands and BINAP appeared to mediate potential radical pathways, further examination was conducted. Dppf was chosen as the test ligand (20 mol %) and was added to reactions containing the alternative sources of palladium that had been examined in Table 1 to observe if this ligand would affect the distribution of coupled products (Table 3).

**Table 3:**
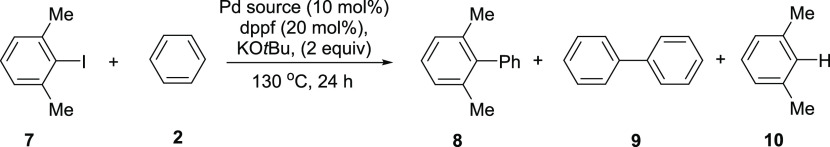
Effect of dppf on the Reaction of Substrate 7 with KO*t*Bu and a Range of Palladium Sources

entry	Pd source	2-iodo-*m*-xylene **7**	2,6-dimethylbiphenyl **8**	biphenyl **9**	*m*-xylene **10**	ratio 8:9
1	Pd(OAc)_2_	0%	3.1%	12.4%	34.6%	1.0:4.0
2	Pd(PPh_3_)_4_	0%	2.6%	8.6%	31.2%	1.0:3.3
3	Pd(PPh_3_)_2_Cl_2_	0%	3.2%	11.6%	36%	1.0:3.6
4	PdCl_2_	72.6%	0.6%	1.4%	5.9%	1.0:2.3
5	Pd(TFA)_2_	0.7%	3.5%	13.0%	36%	1.0:3.7
6	Pd(dppf)Cl_2_	0%	3.2%	11.0%	41.3%	1.0:3.5
7	Pd[P(*o*-tol)_3_]_2_	0.3%	2.8%	9.3%	41.4%	1.0:3.3

Comparing Table 3 with Table 1, it is seen that dramatic alterations occur. Although the absolute yields within Table 3 still vary significantly, the ratios of **8**:**9** are essentially all converted to BHAS values. Thus, it seems that dppf alters the mechanism at Pd regardless of the source.

Several control reactions were conducted (Table 4). In the absence both of an added Pd source and of dppf, trace amounts of both **8** and **9** were noted earlier in entry 1, Table 1. Accordingly, entry 1 in Table 4 is imported from entry 1, Table 1. One possible source of the radical intermediates was that they arose following reductive electron transfer. Ferrocenes and phosphinoferrocenes are potential electron donors [Fc/Fc^+^ is a standard calibrant in cyclic voltammetry].^29^ Adding dppf, (entry 2) but in the absence of any added Pd source, led to yields that were very similar to entry 1, indicating that this ligand alone did not bring about the coupling reaction, contrasting with the report by Wang et al.^30^ Pd(OAc)_2_ was then tested in conjunction with ferrocene (entry 3). The yields compared very well with those in Table 1, entry 2, where no ferrocene was present. This indicated that ferrocene did not have the transformative effect associated with dppf as shown in Table 3. Entry 4 shows, as might be expected, that in the absence of iodoxylene **7**, no evidence of coupled product **8** or biphenyl **9** was present.

**Table 4:**
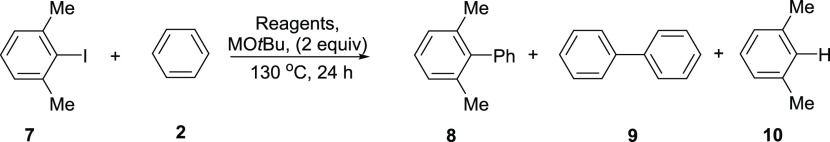
Control Reactions

entry	reagents	**7**	**8**	**9**	**10**	8:9
1		93%	0.3%	0.5%	1.5%	1.0:1.6
2	KO*t*Bu, dppf (20 mol %)	93%	0.2%	0.6%	1.8%	1.0:3.0
3	KO*t*Bu, Pd(OAc)_2_ (10 mol %), ferrocene (20 mol %)	6.2%	15.0%	5.3%	34.1%	2.8:1.0
4	KO*t*Bu, Pd(OAc)_2_ (10 mol %), dppf (20 mol %). (no **7**)	0%	0%	0%	0%	N/A
5	KO*t*Bu, Pd(OAc)_2_, dppf (from Table 2, entry 3)	0%	3.1%	12.4%	34.6%	1.0:4.0
6	NaO*t*Bu, Pd(OAc)_2_, dppf	72.2%	0.1%	0.9%	9.0%	1.0:9.0
7	KO*t*Bu, Pd(OAc)_2_, PCy_3_ (from Table 2, entry 10)	6.9%	27.7%	0.6%	8.2%	46.0:1.0
8	NaO*t*Bu, Pd(OAc)_2_, PCy_3_	6.5%	31.9%	0.8%	35.9%	44.9:1.0

Table 2 (entries 3 and 10) showed that dppf and PCy_3_ follow very different pathways. We know that BHAS pathways are facilitated by KO*t*Bu but that NaO*t*Bu is not an effective promoter in our hands for reactions with substrate **7**(31) (but see ref (20)). Hence, we compared the effect of KO*t*Bu with that of NaO*t*Bu in transformations that used these phosphines. Table 4, entries 5 and 7, reproduces Table 2, entries 3 and 10. Comparing Table 4 entry 5 with entry 6 shows significant suppression of the BHAS pathway with NaO*t*Bu, as expected. That PCy_3_ follows a pathway completely different from that of dppf is seen by comparing entries 7 and 8 in Table 4. Here, the yields of coupled product **8** and biphenyl **9** show no evidence of suppression by NaO*t*Bu. To probe the coupling reactions further, possible mechanisms were considered. The nonradical coupling route to form **8** likely occurs by a CMD (concerted metalation-deprotonation) step (Scheme 3). This type of mechanism had been explored in depth, notably by Fagnou et al.^32−36^ although using different bases. One characteristic observed in those studies was that when the arene partner was deuterated, this led to a substantial primary kinetic isotope effect for the removal of the H/D in Ar–H/D. Although our reactions use a different base, KO*t*Bu, the deprotonation of a benzene molecule would still likely be a challenging step.

Specifically, the product of oxidative addition, **15**, could progress through the transition state **16**(32−36) to the intermediate **17.** The loss of a proton from benzene in the conversion of **15** to **17** might well give rise to a primary kinetic isotope effect (see below). Complex **17** then undergoes reductive elimination to afford product **8**.

**Scheme 4:**
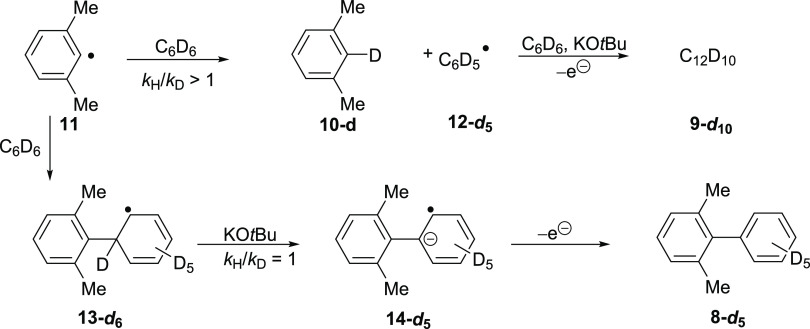
BHAS Coupling of Radical **11** (Derived from **7**) with C_6_H_6_/C_6_D_6_

In contrast, for the formation of 2,6-dimethylbiphenyl **8** from a radical BHAS process (Scheme 4), the addition of the xylyl radical **11** to benzene and benzene-*d*_6_ should occur with almost identical rate constants. In the known examples, the deprotonation step (here **13 → 14** versus **13-*****d***_**6**_**→ 14-*****d***_**5**_) is not the rate-determining step in BHAS and does not lead to a primary kinetic isotope effect, i.e., *k*_H_/*k*_D_**∼**1, and so BHAS reactions would have quite different profiles from the CMD route discussed above.^37,38^ Although the rate of formation of 2,6-dimethylbiphenyl **8** is unaffected by the change of the solvent, the formation of biphenyl **9** by BHAS is retarded when the reaction is conducted in benzene-*d*_6_, with an isotope effect noted for the hydrogen (deuterium) atom transfer (HAT) step from benzene to the xylyl radical **11** (Scheme 4).^27^ This was shown recently in studies that we conducted in C_6_D_6_ and in C_6_H_6_, which were initiated by organic electron donors (see the SI).

Repeating the isotope experiments with the Pd(OAc)_2_/dppf system (Table 5) using benzene versus benzene-*d*_6_ as the solvent produced very similar results to those seen with our previously studied organic electron donor-initiated systems.^27^ The formation of the deuterated biphenyl (1.4%) was suppressed in benzene-*d*_6_ (entry 2) relative to the biphenyl (12.4%) in benzene (entry 1), while the amount of deuterated 2,6-dimethylbiphenyl **8-*****d***_**10**_ was mostly unaffected in benzene-*d*_6_ (3.9% entry 2) relative to 2,6-dimethylmethylbiphenyl **8** (3.1%) in benzene (entry 1), consistent with the reaction through a radical manifold.

**Table 5:**
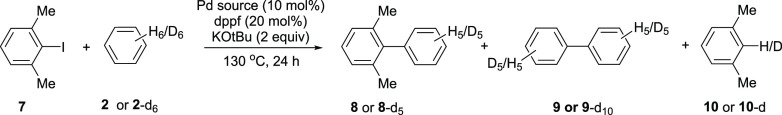
Illustrating Differences in Reactions Involving “Radical” and “Nonradical” Conditions

					2,6-dimethyl-biphenyl		biphenyl		*m*-xylene
entry	Pd source	added ligand	solvent	2-iodo-m-xylene **7**	**8**	**8**-*d*_5_	**9**	**9**-*d*_10_	**10**
1	Pd(OAc)_2_	dppf	C_6_H_6_	0%	3.1%		12.4%		34.6%
2	Pd(OAc)_2_	dppf	C_6_D_6_	0%		3.9%		1.4%	33.0%
3	Pd(PPh_3_)_4_		C_6_H_6_	2.7%	30.3%		1.7%		25.8%
4	Pd(PPh_3_)_4_		C_6_D_6_	7.4%		6.9%		0.3%	45.6%

We previously mentioned that the coupling reaction carried out with Pd(PPh_3_)_4_ showed only a minor amount of biphenyl **9** (1.7%, Table 1, entry 3 also shown as Table 5, entry 3) relative to 2,6-dimethylbiphenyl **8** (30.3%) and so appeared to be one of the most organometallic (nonradical) of the coupling reactions. Accordingly, isotope studies were performed on the reaction with Pd(PPh_3_)_4_ (Table 5, entries 3 and 4) and produced very different results from those of the Pd(OAc)_2_/dppf system. In this case, the amounts of both 2,6-dimethylbiphenyl and biphenyl were affected, being significantly decreased when the reaction was carried out in benzene-*d*_6_ (entry 4). The decrease in the 2,6-dimethylbiphenyl formation from 30.3 to 6.9% is very different from the radical process but is totally consistent with the organometallic CMD process. The decrease in the formation of biphenyl (from 1.7 to 0.3%) is consistent with this compound being formed by a BHAS radical process.

Further to this, radical trapping experiments were conducted in benzene with 2,2,6,6-tetramethylpiperidine-1-oxyl (TEMPO) added to the reaction. The reaction with Pd(OAc)_2_ + dppf was inhibited by the addition of TEMPO, hindering the formation of both coupled products (cf. Table 6, entry 1; Table 5, entry 1; the decreases were from 12.4 to 1.8% for **9** and from 3.1 to 1.6% for **8**). With the Pd(OAc)_2_/dppf case proceeding principally through radical pathways, it explains why this particular experiment was affected by the addition of TEMPO. The greater proportional decrease for **9** than for **8** may indicate that some **8** is still formed by the non-BHAS route.

**Table 6:**
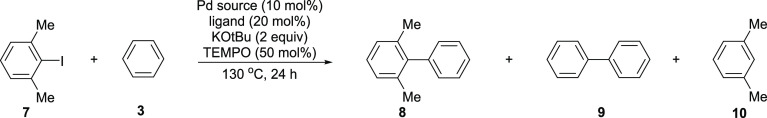
Effect of TEMPO

entry	Pd source	ligand added	2-iodo-*m*-xylene **7**	2,6-dimethylbiphenyl **8**	biphenyl **9**	*m*-xylene **10**
1	Pd(OAc)_2_	dppf	1.4%	1.6%	1.8%	22.5%
2	Pd(PPh_3_)_4_		2.2%	34.7%	0.7%	21.6%

Interestingly, addition of TEMPO to the reaction with Pd(PPh_3_)_4_ did not lead to a decreased level of formation of 2,6-dimethylbiphenyl (*cf*. Table 6, entry 2 with Table 5, entry 3, the amount of **8** increased from 30.3 to 34.7%, while the yield of **9** decreased from 1.7 to 0.7%). The Pd(PPh_3_)_4_ reaction is proposed to proceed through conventional Pd(0)/Pd(II) chemistry to afford **8**. When the minor radical pathway to **8** + **9** is inhibited by TEMPO, this leaves more substrate to be converted through Pd(0)/Pd(II) chemistry to **8**, supporting the observed increase in yield.

**Scheme 5:**
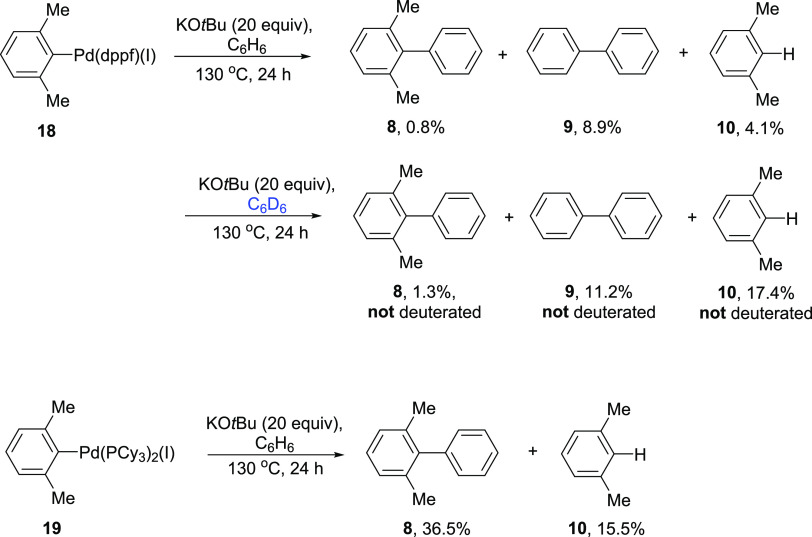
Reactivity of Two Oxidative Addition Complexes.

An important question relating to these reactions is whether aryl radicals form reversibly from oxidative addition complexes. Accordingly, we prepared two oxidative addition complexes **18** and **19** derived from dppf and PCy_3_, respectively. If the dppf complex, on treatment with KO*t*Bu in C_6_H_6_, afforded the same outcomes as seen for substrate **7** + Pd(OAc)_2_ + dppf (Table 2, entry 3), this would indicate reversible formation of aryl radicals. However, this was not the case. The reaction led to the results shown in Scheme 5 [dimethylbiphenyl **8** (0.8%), biphenyl **9** (8.9%), and xylene **10** (4.1%)].

Further examination in C_6_D_6_ showed that the biphenyl product **9** was not deuterated, while the dimethylbiphenyl **8** was predominantly **8-***d*_0_ but showed also some **8**-*d*_5_ isotopologue. The involvement of the P-Ph rings in the formation of unlabeled biaryls in this experiment has a precedent, and similar activity has been documented by numerous authors in the past.^39−41^ Accordingly, this process is quite different from the radical coupling described in Table 5. We conclude that radicals are not formed once the oxidative addition complex has been formed and that the radical coupling in Table 5 occurs faster than the formation of the oxidative addition complex.

The alternative complex studied was the complex ArPd(PCy_3_)_2_(I), **19**. This is an expected intermediate en route to product **8** by the concerted metalation-deprotonation pathway. This complex underwent clean conversion to 2,6-dimethylbiphenyl **8** (36.5%) together with xylene **10** (15.5%) under the conditions of the reaction. Thus, for the CMD route, passage through the oxidative addition complex is fine. Overall, we can conclude that when the BHAS pathway occurs, it occurs before an oxidative addition complex is formed. Once the oxidative addition complex is formed, there is no evidence of BHAS chemistry.

The above results clearly indicate that radicals are formed under specific circumstances; therefore, questions arise about the source of the radicals that initiate the reactions.

Our thoughts were guided by the fact that a peak was observed for low levels of trimethylbenzene in numerous GCMS data from our experiments. Comparison with the three authentic trimethylbenzene isomers surprisingly showed it to be 1,2,3-trimethylbenzene.^42^ The other isomers 1,2,4-trimethylbenzene and the 1,3,5-isomer mesitylene were not present. 1,2,3-Trimethylbenzene would arise by an *ipso*-methylation of iodoxylene (see below). The methyl group would arise by fragmentation of a *tert*-butoxyl radical, which, in turn, would arise following SET from a butoxide salt (*E*_ox_ = +0.10 V vs SCE)^37^ anion to an unknown electron acceptor. In support of this proposal that KO*t*Bu is the source of the methyl group, when the experiments were conducted in KO*t*Bu-*d*_9_, the trimethylbenzene was principally trideuterated C_9_H_9_D_3_ (see the SI).

Initial thoughts were that a methyl radical might have formed a C–Pd bond before or after oxidative addition of Pd to the iodoarene substrate and that this would be followed by reductive elimination to form the methylated arene. That would account for the formation of the 1,2,3-trimethyl regioisomer. However, when a BHAS experiment was performed under our non-Pd conditions, using an organic additive (phenanthroline) that would be converted into an electron donor on treatment with KO*t*Bu,^17^ this also showed 1,2,3-trimethylbenzene as a single isomer. Computational studies (see the SI) show that the methyl radical can undergo a concerted substitution of the C–I bond of 2-iodo-*m*-xylene with a barrier of 19.9 kcal mol^–1^. Comparable barriers (18–20 kcal mol^–1^) are seen for addition to any of the arene carbons of 2-iodo-*m*-xylene, but these reactions are all exergonic by 3–4 kcal mol^–1^ and so are reversible under the reaction conditions, while the displacement of the iodine atom (ΔG* = 19.9 kcal mol^–1^; Δ*G*_rel_ = −33.4 kcal mol^–1^) is significantly exergonic and not reversible, and so this unexpected reaction is favored. Evidence that the product arises from iodoxylene rather than from xylene is that no toluene was ever observed in our reactions where benzene was the solvent (even though toluene can be detected on our GC systems). Therefore, the reaction of methyl radicals with haloarenes, rather than with arenes, gives a methylated product.^42^

We also examined whether iodine atom abstraction from the iodoarene substrate by methyl radicals would be possible, but this reaction is endergonic (ΔG* = 15.5 kcal mol^–1^; Δ*G*_rel_ = +8.9 kcal mol^–1^; see the SI).

**Scheme 6:**
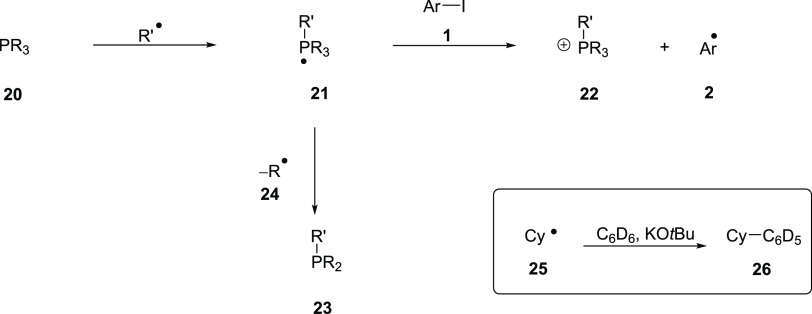
Formation and Reactions of Phosphoranyl Radicals

The possibility of KO*t*Bu acting as an electron donor to ArI has been proposed before in BHAS chemistry.^43^ However, as shown in Table 1 (entry 1), this background source of methyl radicals cannot sustain more than trace levels of formation of coupled products, so the methyl radicals are themselves ineffective in carrying out BHAS. Some factors need to be present in the reactions to assist the initiation of BHAS chemistry. In this regard, phosphines are excellent radical traps. Radical addition to a phosphorus atom would give hypervalent phosphoranyl radicals^44−47^ that are reasonably strong electron donors; indeed, some phosphoranyl radicals (derived from phosphites) have been proposed to undergo electron transfer to iodobenzenes.^48^ [Computational calculations show that the transition states for such reactions might be achievable (see the SI).] Thus, addition of radical *R*^•^ to the phosphorus atom in phosphine **20** would afford the phosphoranyl radical **21**, which would undergo inner-sphere or outer-sphere electron transfer to iodoarene **1** and would generate phosphonium salt **22** and aryl radical **2** (Scheme 6).

However, we know that free dppf is not able to sustain BHAS in our system (Table 4, entry 2) and so it may be that some further assistance is needed to make the reaction more easily achievable.

We have seen evidence of phosphoranyl radicals in our experiments. Phosphoranyl radicals, e.g., **21**, that bear an alkyl–P bond undergo cleavage of such bonds^44,45^ to liberate the alkyl radical **24** and, in a BHAS reaction in benzene, the alkyl radical will form an alkylbenzene, which we have detected in our GCMS traces. Thus, for example, cyclohexylbenzene **26** was detected in the reaction of Fc(PCy_2_)_2_ in benzene. Attack of a methyl, phenyl, or other radical on Fc(PCy_2_)_2_ forms an intermediate phosphoranyl radical **21** from which a cyclohexyl radical **25** fragments. Attack by **25** on benzene could then lead, by BHAS chemistry, to cyclohexylbenzene **26**.

Returning to our reaction system, the ligands that support the BHAS reaction are phosphinoferrocenes and BINAP. To trigger BHAS, an electron donor that is strong enough to donate an electron to an iodoarene is needed. These ligands themselves are not strong-enough donors to support BHAS as electron donors (Table 4, entry 2), but a phosphoranyl radical that can be supported by an electron-rich Pd(0) atom would have good credentials to act as an electron donor.

From what was said earlier in the paper, the BHAS mechanism is in competition with the oxidative addition at Pd. Ligands that form Pd complexes, which are very effective at oxidative addition (e.g., PCy_3_), may convert to the oxidative addition intermediates too rapidly for BHAS to have a chance to occur. Accordingly, Pd complexes that give successful BHAS reactions will have ligands that are less electron-rich than these. Hence, BHAS can compete or even dominate their chemistry. Dppf and BINAP can be members of this intermediate group. These ligands feature ferrocenes and naphthalenes bonded to phosphorus; these arenes are a lot more electron-releasing than benzenes.^49^ The third class of ligands with phenyl groups attached to phosphorus (e.g., PPh_3_) will be less electron-rich and less supportive of electron transfer. Their complexes may also be relatively slow at oxidative addition, and so for them, both BHAS and CMD chemistry compete. This proposal would then explain the selectivity seen across the range of ligands of Pd.

## Conclusions

Strong evidence is presented here that the reaction mechanism for the formation of biaryl coupled products through ground-state activation by Pd salts and complexes is controlled by the ligands. Two pathways appear likely, one involving Pd(0)/Pd(II) chemistry and the other progressing through formation of aryl radicals. Different ligands form a hierarchy with phosphines of intermediate electron-richness, e.g., phosphinoferrocenes and BINAP promoting the tendency for radical coupling. Very electron-rich phosphines PCy_3_ and DCPE promote organopalladium coupling likely because they undergo very rapid oxidative addition, while less electron-rich phosphines, e.g., DPEPhos and XantPhos, do not discriminate notably. Products of methyl radicals and phosphoranyl radicals are observed in these reactions. These observations can alert chemists to a new facet of ligand chemistry and highlight that even the choice of alkali metal alkoxide can have important implications for reaction outcome. We are currently studying the effects of alkyl radicals and phosphoranyl radicals on different metal systems.

## Data Availability

A data set collection of computational results is available in the ioChem-BD repository^50^ and can be accessed via 10.19061/iochem-bd-6-281.

## Supporting Information Available

Experimental procedures, spectroscopic, and chromatographic data supporting the experiments, DFT data as well as X-ray crystal structure data for Pd(dppf)(Xyl)(I) (PDF)
