# Supplementary material for: A Hierarchy of Ligands Controls Formation and Reaction of Aryl Radicals in Pd-Catalyzed Ground-State Base-Promoted Coupling Reactions
Source: J Am Chem Soc. 2023 Sep 15;145(38):20849–58. doi: 10.1021/jacs.3c05470 (PMC10540214; doi:10.1021/jacs.3c05470)
Supplement: Supplementary file 1 — ja3c05470_si_001.pdf [file ja3c05470_si_001.pdf]

A Hierarchy of Ligands Controls Formation and Reaction of Aryl Radicals in Pd-Catalyzed Ground State Base-Promoted Coupling Reactions

Kenneth F. Clark,[a] Seb Tyerman,[a] Laura Evans,[b] Craig M. Robertson,[c] David J. Nelson,[a] Alan R. Kennedy[a] and John A. Murphy[a]*

[a]Department of Pure and Applied Chemistry, University of Strathclyde, 295 Cathedral Street, Glasgow G1 1XL, United Kingdom. [b]Medicinal Chemistry, Research and Early Development, Oncology R&D, AstraZeneca, Cambridge, CB10 1XL, United Kingdom. [c]GSK Medicines Research Centre, Gunnels Wood Road, Stevenage, Herts SG1 2NY, United Kingdom.

Supplementary Information

Contents

General Information……………………………………………………………………S2

Experimental details……………………………………………………………………S3

General Procedures……………………………………………………………………S3

Verifying identity of 1,2,3-trimethylbenzene……………………………………S60

Deuterated 1,2,3-trimethylbenzene from KOtBu-d_9……………………………S66

Example GCMS Mass Traces…………………………………………………………S69

Calibration Information………………………………………………………………S73

GC Data…………………………………………………………………………………S78

NMR spectra……………………………………………………………………………S102

X-ray crystallography…………………………………………………………………S116

DFT data ………………………………………………………………………………S118

General Information

All reagents and solvents were obtained from commercial suppliers and were used without further purification unless mentioned otherwise. Where used, diethyl ether and THF were dried using a Pure-Solv 400 solvent purification system (Innovative Technology Inc., USA). Where stated, reactions were prepared in a glovebox supplied by Innovative Technology Inc., USA, which is operated with a nitrogen atmosphere.

Analysis Techniques

i) **Thin Layer Chromatography** (TLC) was performed on silica gel pre-coated aluminium plates (60 Å, F254 UV indicator) purchased from Merck. The thin layer chromatograms were analysed by UV (254 nm, UVP mineralight UVG-11 lamp) and staining either with basic $KMnO_4$ [$KMnO_4$ (6 g), K_2CO_3 (40 g), NaOH (5 mL, 10% w/w) in water (600 mL)] or an ethanolic solution of phosphomolybdic acid [phosphomolybdic acid hydrate (10 g) in ethanol (100 mL)].

ii) **Flash Column Chromatography** purification was performed with 35-70 µm particle size silica gel 60 Å (200-400 mesh) purchased from Prolabo.

iii) **Melting points** were determined using a Gallenkamp Griffin Melting Point Apparatus.

iv) **NMR** spectroscopy was performed using Bruker spectrometers, either: an AV3-400 and AV3-400Nano. ^{1}H NMR, ^{13}C NMR and ^{31}P NMR spectra were recorded on these spectrometers operating at 400 MHz, 100 MHz and 162 MHz, respectively. All spectral data were acquired at 295 K. Chemical shifts (δ) are quoted in parts per million (ppm) relative to the following residual solvent peaks, δ_H 7.26 and δ_C 77.16 for $CDCl_3$ and δ_H 5.32 and δ_C 53.50 for CD_2Cl_2, was used as a reference. Coupling constants (*J*) are reported in Hertz (Hz) to the nearest 0.1 Hz. The multiplicity abbreviations used are: s (singlet), d (doublet) and m (multiplet).

v) **Infrared (IR)** spectra were obtained on a Shimadzu IRAffinity-1 FTIR-ATR spectrometer instrument.

vi) **GCMS** spectra were obtained on an Agilent 7890A GC system coupled to a 5975C inert XL EI/CI MSD triple axis-mass detector. Electron impact (EI) ionisation was utilised, specifically the method EI320. The column temperature was 320 °C, and the carrier gas was helium with a flow rate of 1 mL/min and was operated in splitless mode.

vii) **GCFID** analyses were carried out using an Agilent 7890A gas chromatograph fitted with an Agilent HP5 column (30 m x 0.25 mm x 0.25 µm). Helium was used as the carrier gas (2.0 mL/min flow rate). The injector temperature was 320 °C and was operated in splitless mode. Calibration curves were determined for Individual compounds (pp. S50-S54).

Experimental Details

Assigned peaks in the GCFID spectra have been quantitatively calibrated (for calibrations see pages S50 – S54. During the investigation, the column was changed on the GCFID instrument, and so the retention times for all peaks shifted. A repeat calibration for all calibrated compounds was carried out.

General Procedures

General Procedure A - Reactions of 2-Iodo-*m*-xylene **7** with KO^{t}Bu with Pd Salts

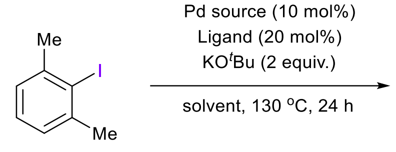

7

To an oven-dried microwave vial, primed with a stirrer bar, in a glovebox was added 2-iodo-*m*-xylene **7** (102 µL, 0.7 mmol, 1 equiv.), Pd source (0.07 mmol, 10 mol%) unless otherwise stated, ligand (0.14 mmol, 20 mol%) unless otherwise stated, base (1.4 mmol, 2 equiv.) and solvent(s) (7 mL) with the vial subsequently sealed and stirred at 130 °C for 24 h. Once complete, the crude mixture was allowed to cool to room temperature and dodecane (23 µL) was added as a GC calibrant and mixed. A 100 µL aliquot of the crude mixture was then analysed by both GCMS and GCFID.

Preparation of 2,6-Dimethylbiphenyl **8**

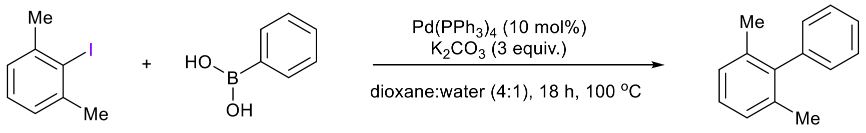

7 **S1** **8**

To an oven-dried microwave vial, primed with a stirrer bar, was added 2-iodo-*m*-xylene **7** (94 µL, 0.65 mmol, 1 equiv.), phenylboronic acid **S1** (95 mg, 0.78 mmol, 1.2 equiv.), K_2CO_3 (269 mg, 1.95 mmol, 3 equiv.), $Pd(PPh_3)_4$ (81 mg, 0.07 mmol, 10 mol%) and a dioxane/water (5 mL, 4:1). The vial was subsequently sealed and stirred for 18 h at 100 °C. Once complete, the reaction mixture was diluted with water and extracted with DCM, before being concentrated *in vacuo*. Purification by column chromatography (eluent 100% hexane) afforded 2,6-dimethylbiphenyl **8** as a colourless oil (82 mg, 0.45 mmol, 69%). **^{1}H NMR** (400 MHz, $CDCl_3$) δ 7.48 – 7.40 (m, 2H), 7.37 – 7.31 (m, 1H), 7.20 – 7.09 (m, 5H) 2.04 (s, 6H). **^{13}C NMR** (101 MHz, $CDCl_3$) δ 141.9, 141.1, 136.1, 129.0, 128.4, 127.3, 127.0, 126.6, 20.8. **ATR-IR** ν_{max} (neat)/cm^{-1} 2920, 2851, 1460, 1377, 1186. ***m/z*** (EI) 182 (M^+, 85), 167 (100), 89 (14), 63 (6), 55 (6). The data for this compound are consistent with those reported in the literature.[1]

Reaction of 2-Iodo-*m*-xylene **7** with KO^{t}Bu (Table 1, Entry1)

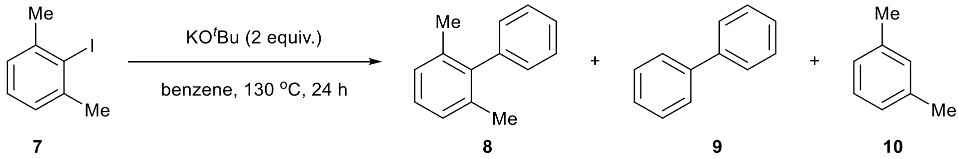

To an oven-dried microwave vial, primed with a stirrer bar, in a glovebox, was added 2-iodo-*m*-xylene **7** (102 µL, 0.7 mmol, 1 equiv.), KO^{t}Bu (157 mg, 1.4 mmol, 2 equiv.) and benzene (7 mL), with the vial subsequently sealed and stirred at 130 °C for 24 h. Once complete, the crude mixture was allowed to cool to room temperature and dodecane (23 µL) was added and mixed. A 100 µL aliquot of the crude mixture was then analysed by both GCMS and GCFID.

Below, please find GCFID of reaction of **7** with KO^{t}Bu in C_6H_6, followed by Tables quantitating the compounds that had been calibrated:

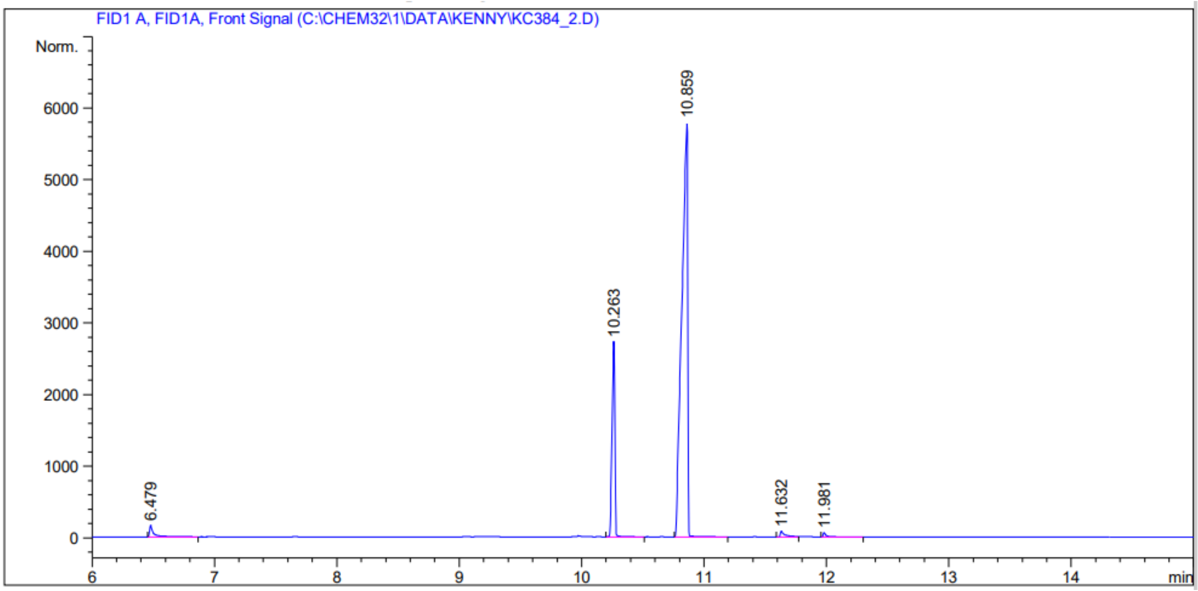

Retention Time (min)	Sample	Peak Area	%Yield
6.479	*m*-Xylene **10**	161.60567	1.5%
10.263	Dodecane (calibrant)	2731.02173	N/A
10.859	2-Iodo-*m*-xylene **7**	5787.97021	93.0%
11.632	Biphenyl 9	78.18199	0.5%
11.981	2,6-Dimethylbiphenyl **8**	56.76885	0.3%

Outcome - The Reaction of 2-Iodo-*m*-xylene **7** with KO^{t}Bu led to trace formation of **8**, **9** and **10** with large quantities of **7** remaining after the 24 h.

Reaction of 2-Iodo-*m*-xylene **7** with $Pd(OAc)_2$ and KO^{t}Bu (Table 1, Entry 2)

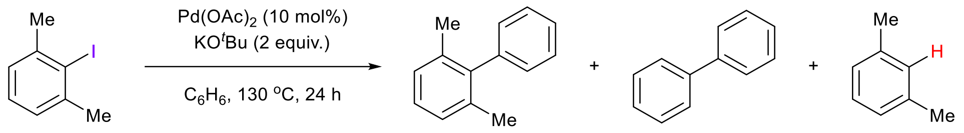

7 **8** **9** **10**

The reaction was conducted according to General Procedure A with $Pd(OAc)_2$ (16 mg, 0.07 mmol, 10 mol%), KO^{t}Bu (157 mg, 1.4 mmol, 2 equiv.) and benzene.

Below, please find GCFID data including tables quantitating components that had been quantitatively calibrated (for calibrations see pages S73 - S77)

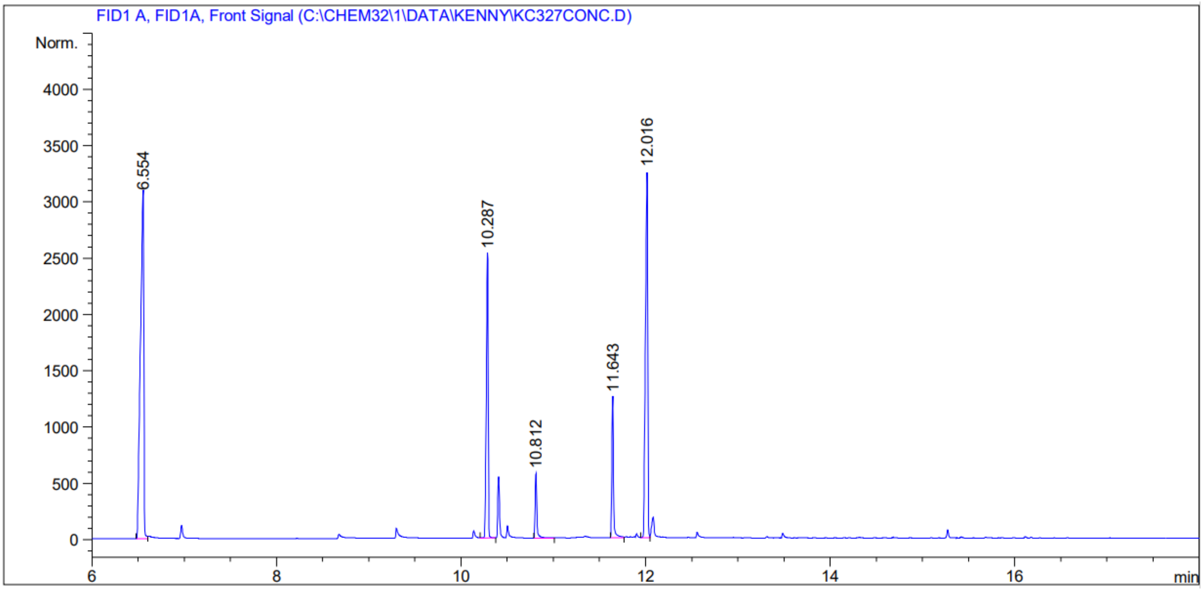

Retention Time	Sample	Peak Area	%Yield
6.554	*m*-Xylene **10**	7833.24902	36.8%
10.287	Dodecane	3568.38916	N/A
10.812	2-Iodo-*m*-xylene **7**	727.10522	3.5%
11.643	Biphenyl 9	1589.42285	5.2%
12.016	2,6-Dimethylbiphenyl **8**	5748.82373	15.9%

*This reaction was carried out in duplicate with the average below

Sample	%Yield
m-Xylene **10**	36.4%
2-Iodo-*m*-xylene **7**	6.3%
Biphenyl **9**	5.5%
2,6-Dimethylbiphenyl **8**	15.4%

Outcome - The reaction of 2-Iodo-*m*-xylene **7** with $Pd(OAc)_2$ and KO^{t}Bu led to the formation of the 3 principal compounds **8**- **10**. The ratio of **8**:**9** [2.8:1] suggests that an organometallic mechanism principally underpins C-C bond formation with this Pd salt.

Reaction of 2-Iodo-*m*-xylene **7** with $Pd(PPh_3)_4$ and KO^{t}Bu (Table 1, Entry 3)

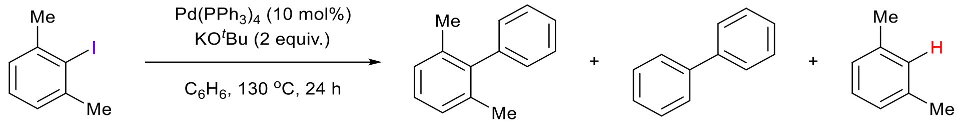

The reaction was conducted according to General Procedure A with $Pd(PPh_3)_4$ (81 mg, 0.07 mmol, 10 mol%), KO^{t}Bu (157 mg, 1.4 mmol, 2 equiv.) and benzene.

GCFID data follow including tables quantitating components that had been quantitatively calibrated (for calibrations see pages S73 - S77).

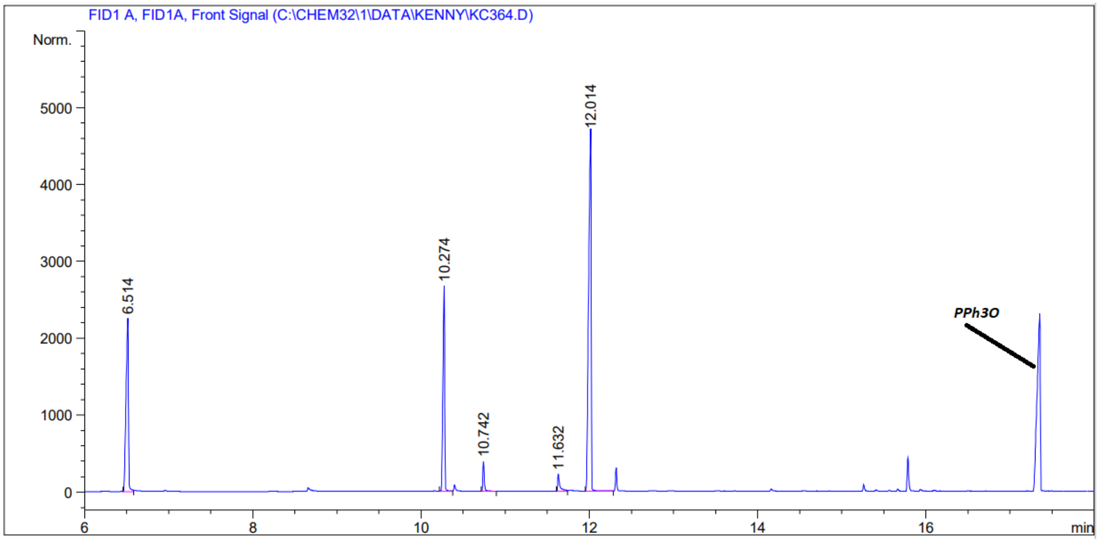

Retention Time	Sample	Peak Area	%Yield
6.514	*m*-Xylene **10**	4569.34717	24.6%
10.274	Dodecane	3907.17798	N/A
10.742	2-Iodo-*m*-xylene **7**	453.20984	2.6%
11.632	Biphenyl **9**	388.08752	1.5%
12.014	2,6-Dimethylbiphenyl **8**	9783.77148	31.0%

*This reaction was carried out in duplicate with the average below

Sample	%Yield
m-Xylene **10**	25.8%
2-Iodo-*m*-xylene **7**	2.7%
Biphenyl **9**	1.7%
2,6-Dimethylbiphenyl **8**	30.3%

Outcome - The reaction of 2-Iodo-*m*-xylene **7** with $Pd(PPh_3)_4$ and KO^{t}Bu led to the formation of the 3 principal compounds **8**, **9** and **10**. The ratio of **8**:**9** [17.8:1]suggests that an organometallic mechanism principally underpins C-C bond formation with this Pd salt.

Synthesis of Pd[P(*o*-tol)$_3$]$_2$ needed for Table 1, Entry 4

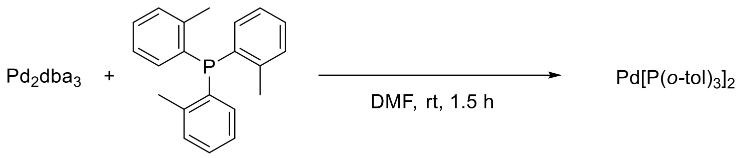

An oven dried 25 mL microwave vial was charged with Pd_2dba_3 (458 mg, 0.5 mmol), P(*o*-tol)$_3$ (609 mg, 2 mmol, 4 eq.) and a stirbar in a glovebox. The vial was capped, removed from the box and dry DMF (18 mL) was added *via* syringe under argon. The purple reaction mixture was stirred at room temperature for 1.5 h, after which a yellow solid had precipitated from a brown solution. The solid was collected by vacuum filtration in a glovebox and washed with Et_2O yielding Pd[P(*o*-tol)$_3$]$_2$ as a yellow powder (556 mg, 0.777 mmol, 78 %). **^{1}H NMR** (400 MHz, C_6D_6) δ 7.08 – 7.02 (m, 18H), 6.85 – 6.80 (m, 6H), 2.96 (s, 18H). **^{31}P NMR** (162 MHz, C_6D_6) δ -6.81. The ^{31}P NMR shows a small impurity of free P(*o*-tol)$_3$ at δ -29.98. Data for the compound agrees with literature values.[6,7]

Reaction of 2-Iodo-*m*-xylene **7** with Pd[P(*o*-tol)$_3$]$_2$ and KO^{t}Bu (Table 1, Entry 4)

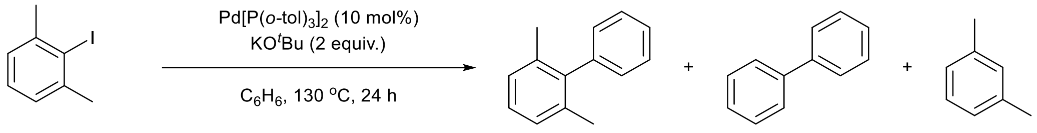

7 **8** **9** **10**

The reaction was conducted according to General Procedure A with Pd[P(*o*-tol)$_3$]$_2$ (25 mg, 0.035 mmol, 10 mol%), KO^{t}Bu (78 mg, 0.7 mmol, 2 equiv.) and benzene (2.5 mL).

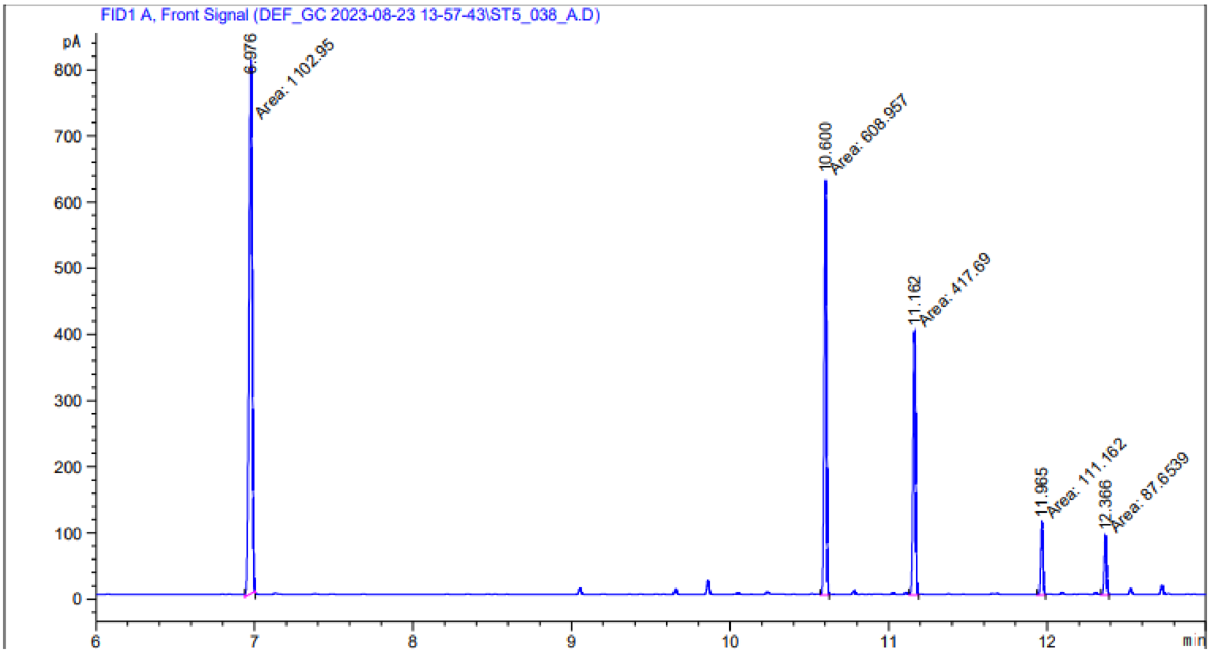

Retention Time	Sample	Peak Area	%Yield
6.976	*m*-Xylene **10**	1102.95	49.4
10.600	Dodecane	608.96	N/A

11.162	Iodoxylene **7**	417.69	19.4
11.965	Biphenyl **9**	111.16	3.7
12.366	2,6-Dimethylbiphenyl **8**	87.65	2.7

Formation of $Pd(dppf)_2$ (for Table 1, entry 5)

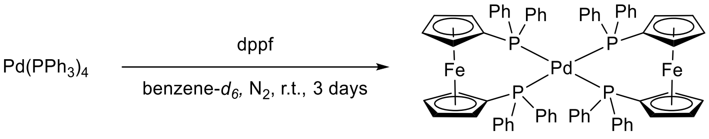

An oven-dried microwave vial was transferred to a glovebox, where $Pd(PPh_3)_4$ (400 mg, 0.35 mmol, 1 equiv.), dppf (384 mg, 0.7 mmol, 2 equiv.) and benzene (4 mL) was added, with the vial subsequently sealed left to stand for 3 days. Once complete, pentane was slowly added to the solution until a yellow precipitate formed. This was then filtered by vacuum filtration to yield the Pd complex, $Pd(dppf)_2$ (182 mg, 0.15 mmol, 42%) as a yellow/orange solid. **^{1}H NMR** (400 MHz, C_6D_6) δ 7.79 (br s, 16H), 6.97 – 6.90 (m, 24H), 4.29 (s, 8H), 3.99 (s, 8H). **^{31}P NMR** (162 MHz, C_6D_6) δ 8.11 (s). ***m/z*** (ESI+) 1371 ($[M+(PhH)_2]^+$). The data for this compound are consistent with those reported in the literature.[2]

Reaction of 2-Iodo-*m*-xylene **7** with $Pd(dppf)_2$ and KO^{t}Bu (Table 1, Entry 5)

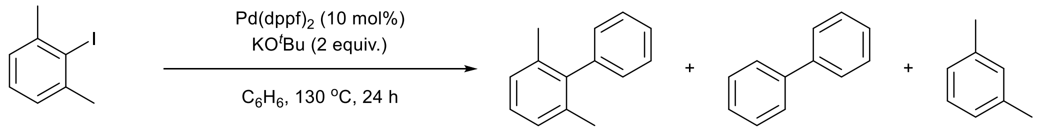

7 **8** **9** **10**

Experiment	2-iodo-*m*-xylene	2,6-dimethylbiphenyl **8**	Biphenyl *9*	*m*-xylene **10**
**1**	0.3%	2.8%	11.3%	32.2%
**2**	0.3%	2.7%	11.2%	32.2%

The reaction was conducted according to General Procedure A with $Pd(dppf)_2$ (85 mg, 0.07 mmol, 10 mol%), KO^{t}Bu (157 mg, 1.4 mmol, 2 equiv.) and benzene (5 mL).

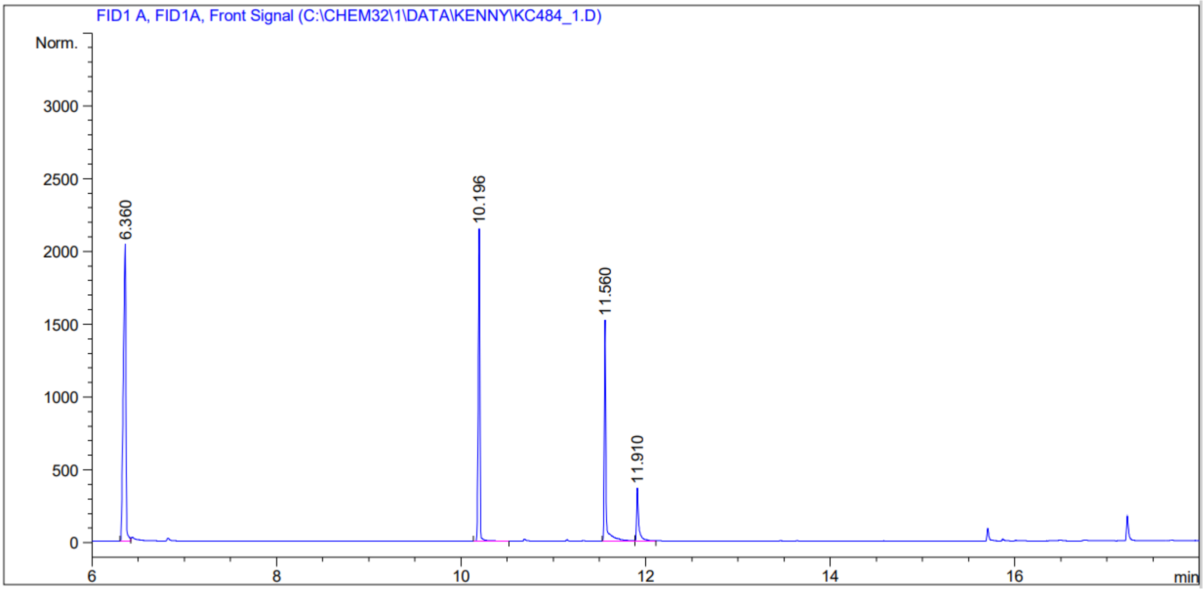

Retention Time	Sample	Peak Area	%Yield
6.360	*m*-Xylene	4068.96313	32.2%
10.196	Dodecane	2755.40234	N/A
11.560	Biphenyl	2038.75659	11.3%
11.910	2,6-Dimethylbiphenyl	591.57062	2.8%

*This reaction was carried out in duplicate with the average below

Sample	%Yield
m-Xylene	32.2%
2-Iodo-*m*-xylene	0
Biphenyl	11.3%
2,6-Dimethylbiphenyl	2.8%

Synthesis of Pd(BINAP)$_2$ (for Table 1, entry 6)

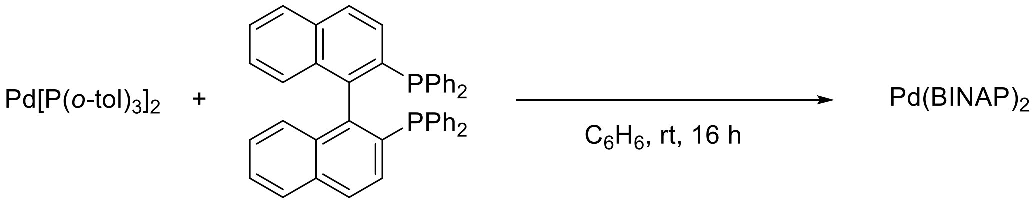

An oven dried 25 mL microwave vial was charged with Pd[P(*o*-tol)$_3$]$_2$ (357 mg, 0.5 mmol), *rac*-BINAP (621 mg, 1 mmol, 2 eq.), anhydrous benzene (10 mL) and a stirbar in a glovebox. The flask was capped and stirred for 16 h, giving a deep purple solution and insolubles. The mixture was filtered *in vacuo* in a glovebox, and the solids in the filter further washed with benzene (5 mL). The filtrate was then layered with pentane and left to crystalize at room temperature overnight, and the resulting solid was triturated with further pentane yielding Pd(BINAP)$_2$ as a dark red solid (217 mg, 0.16 mmol, 32 %). **^{1}H NMR** (400 MHz, C_6D_6) δ 8.40 – 8.33 (m, 8H), 7.85 – 7.81 (m, 4H), 7.51 – 7.44 (m, 8H), 7.39 – 7.35 (m, 8H), 7.26 – 7.17 (m, 16H), 7.06 (ddd, *J* = 8.0, 6.8, 1.0 Hz, 4H), 6.85 (ddd, *J* = 8.3, 6.8, 1.3 Hz, 4H), 6.19 (t, *J* = 7.4 Hz, 4H), 5.88 (t, *J* = 7.6 Hz, 8H). **^{31}P NMR** (162 MHz, C_6D_6) δ 27.20. Data for the compound agrees with literature values.[2]

Reaction of 2-Iodo-*m*-xylene **7** with Pd(BINAP)$_2$ and KO^{t}Bu (Table 1, Entry 6)

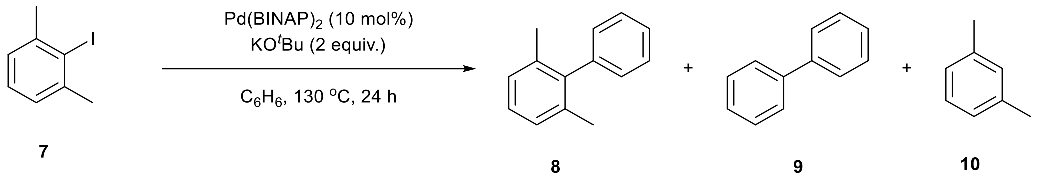

The reaction was conducted according to General Procedure A with Pd(BINAP)$_2$ (47 mg, 0.035 mmol, 10 mol%), KO^{t}Bu (79 mg, 0.7 mmol, 2 equiv.) and benzene (2.5 mL).

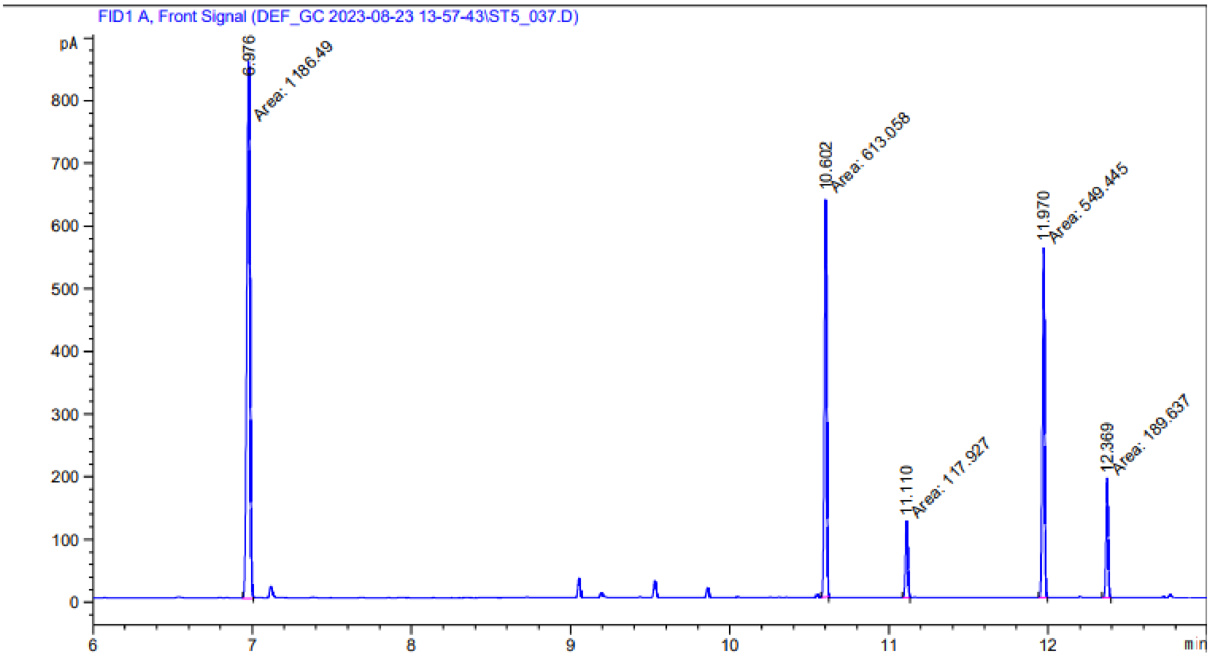

Retention Time	Sample	Peak Area	%Yield
6.976	*m*-Xylene **10**	1186.49	47.9
10.602	Dodecane	613.06	N/A
11.110	Iodoxylene **7**	117.93	4.9
11.970	Biphenyl **9**	549.45	16.7
12.369	2,6-Dimethylbiphenyl **8**	189.64	5.2

Reaction of 2-Iodo-*m*-xylene **7** with Pd(PPh$_3$)$_2$Cl$_2$ and KO^{t}Bu (Table 1, Entry 7)

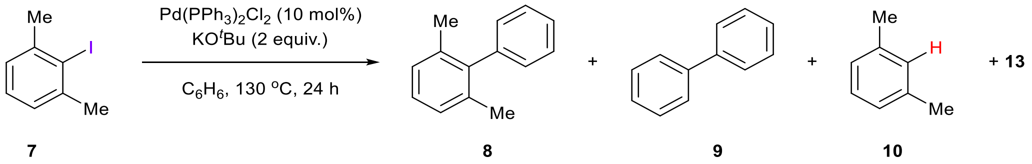

The reaction was conducted according to General Procedure A with $Pd(PPh_3)_2Cl_2$ (49 mg, 0.07 mmol, 10 mol%), KO^{t}Bu (157 mg, 1.4 mmol, 2 equiv.) and benzene.

GCFID data are below including tables quantitating components that had been quantitatively calibrated (for calibrations see pages S73 - S77).

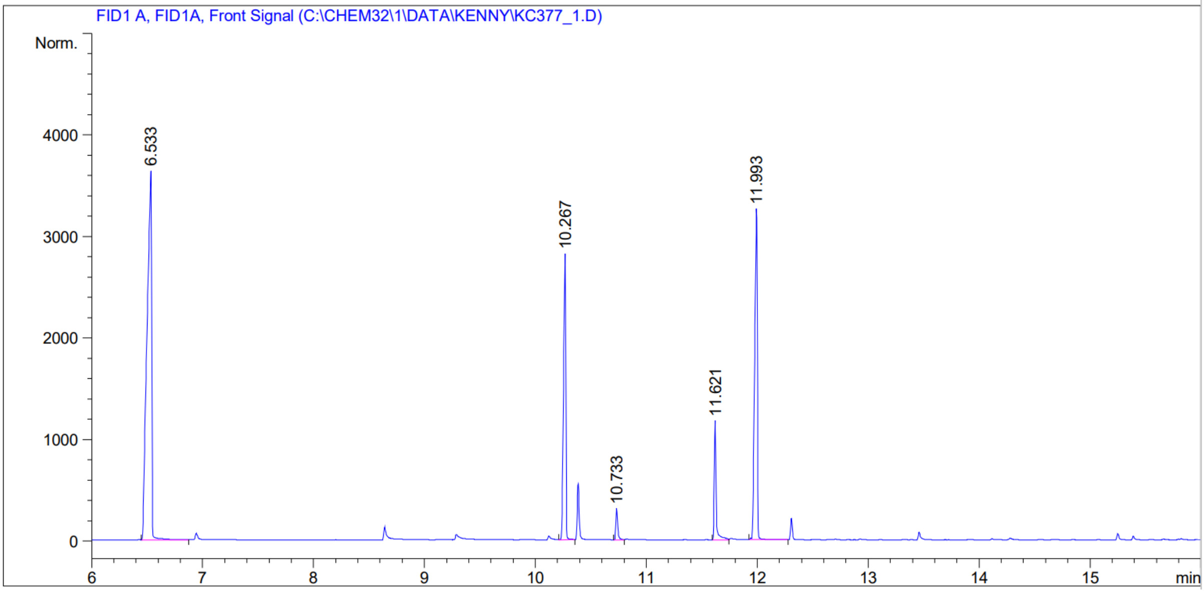

Retention Time	Sample	Peak Area	%Yield
6.533	*m*-Xylene **10**	10520.8	48.1%
10.267	Dodecane	4126.54346	N/A
10.733	2-Iodo-*m*-xylene **7**	360.01434	1.7%
11.621	Biphenyl **9**	1423.20239	4.6%
11.993	2,6-Dimethylbiphenyl **8**	5510.81934	14.8%

*This reaction was carried out in duplicate with the average below

Sample	%Yield
m-Xylene **10**	46.7%
2-Iodo-*m*-xylene **7**	1.7%
Biphenyl **9**	4.5%
2,6-Dimethylbiphenyl **8**	14.9%

Outcome: The reaction of 2-iodo-*m*-xylene **7** with $Pd(PPh_3)_2Cl_2$ and KO^{t}Bu led to the formation of the 3 principal compounds **8**, **9** and **10**. The ratio of **8**:**9** [3.3:1] suggests that an organometallic mechanism principally underpins C-C bond formation with this Pd salt.

Reaction of 2-Iodo-*m*-xylene **7** with $PdCl_2$ and KO^{t}Bu (Table 1, Entry 8)

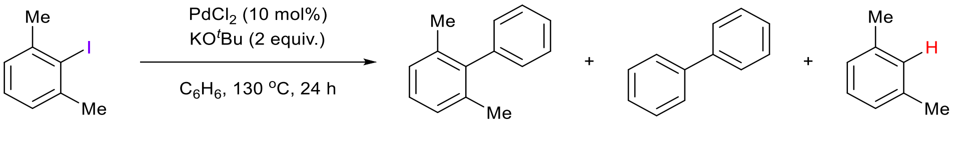

The reaction was conducted according to General Procedure A with $PdCl_2$ (12 mg, 0.07 mmol, 10 mol%), KO^{t}Bu (157 mg, 1.4 mmol, 2 equiv.) and benzene.

GCFID data are below, including tables quantitating components that had been quantitatively calibrated (for calibrations see pages S73 - S77).

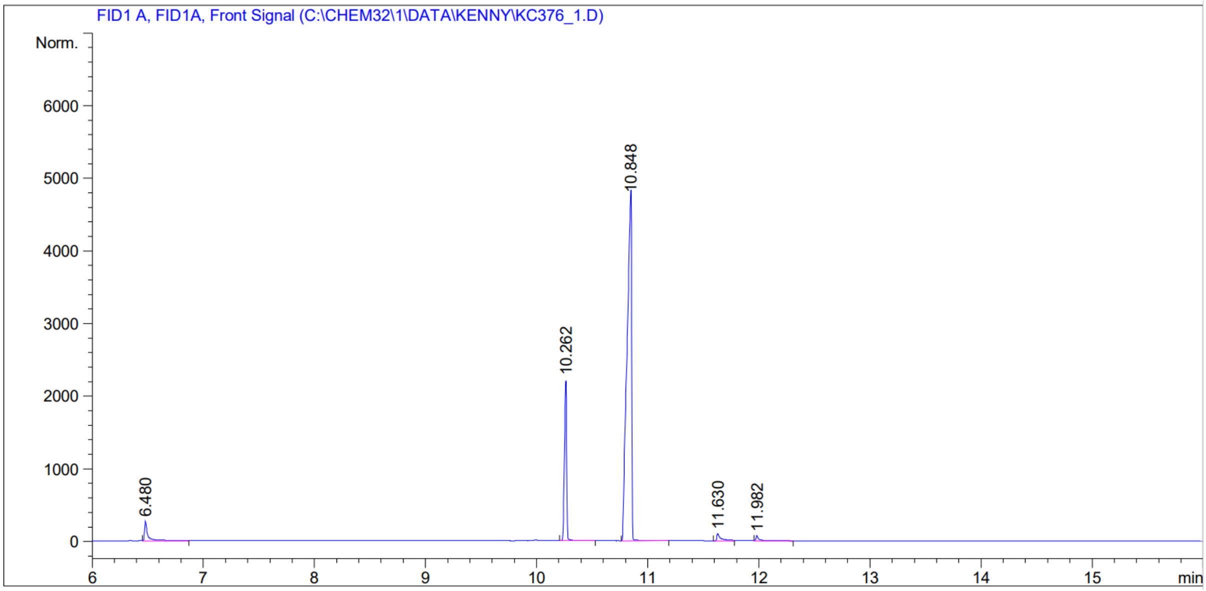

Retention Time	Sample	Peak Area	%Yield
6.480	*m*-Xylene **10**	564.74121	3.2%
10.262	Dodecane	3045.12256	N/A
10.848	2-Iodo-*m*-xylene **7**	13952.7	86.6%
11.630	Biphenyl **9**	255.63853	0.8%
11.982	2,6-Dimethylbiphenyl **8**	132.48004	0.6%

*This reaction was carried out in duplicate with the average below

Sample	%Yield
m-Xylene **10**	3.2%
2-Iodo-*m*-xylene **7**	86.8%
Biphenyl **9**	1.0%
2,6-Dimethylbiphenyl **8**	0.6%

Outcome - The reaction of 2-iodo-*m*-xylene **7** with $PdCl_2$ and KO^{t}Bu led to the formation of the 3 principal compounds **8**, **9** and **10**. The ratio of **8**:**9** [1:1.7] suggests that both an organometallic mechanism and a BHAS mechanism play a role in formation of C-C bonds here.

<u>Reaction of 2-Iodo-*m*-xylene **7** with $Pd(TFA)_2$ and KO^{t}Bu (Table 1, Entry 9)</u>

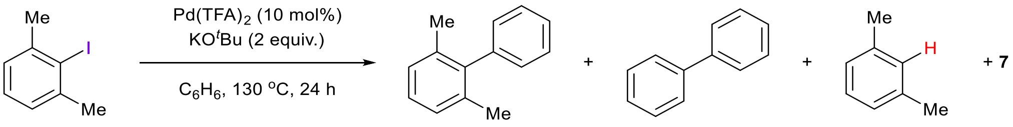

The reaction was conducted according to General Procedure A with palladium trifluoroacetate [Pd(TFA)$_2$] (23 mg, 0.07 mmol, 10 mol%), KO^{t}Bu (157 mg, 1.4 mmol, 2 equiv.) and benzene.

GCFID data follow, including tables quantitating components that had been calibrated (for calibrations, see pages S73 - S77).

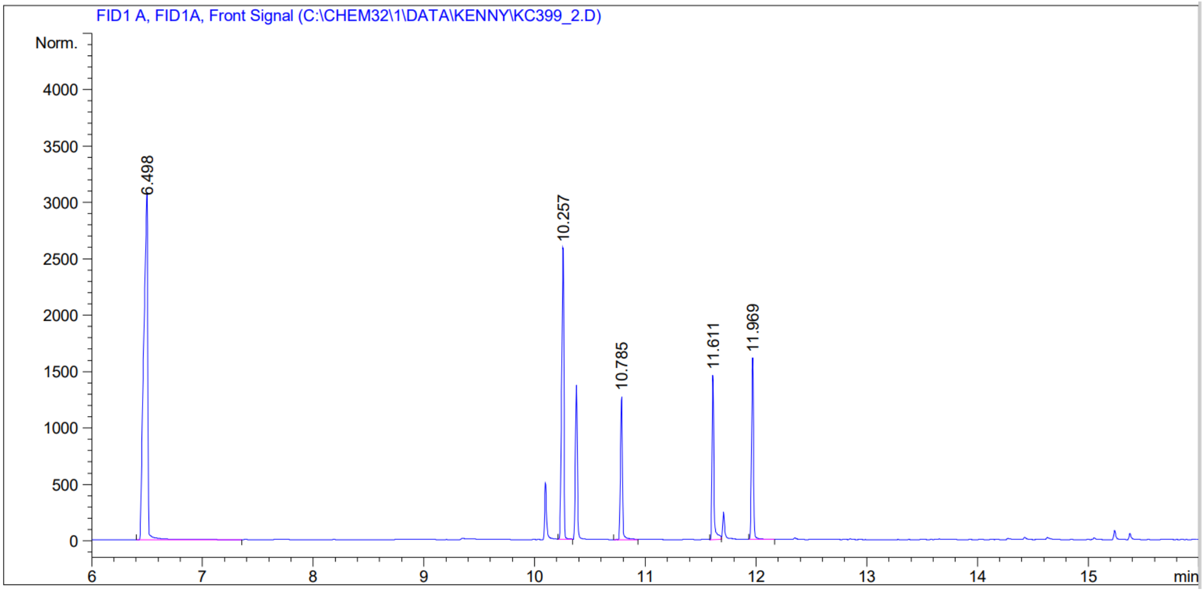

Retention Time	Sample	Peak Area	%Yield
6.498	*m*-Xylene **10**	7932.22363	44.9%
10.257	Dodecane	3695.10742	N/A
10.785	2-Iodo-*m*-xylene **7**	1627.69214	10.8%
11.611	Biphenyl **9**	1754.34570	7.2%
11.969	2,6-Dimethylbiphenyl **10**	2005.48328	6.6%

*This reaction was carried out in duplicate with the average below

Sample	%Yield
m-Xylene **10**	45.1%
2-Iodo-*m*-xylene **7**	10.3%
Biphenyl **9**	7.1%
2,6-Dimethylbiphenyl **8**	6.7%

Outcome - The Reaction of 2-Iodo-*m*-xylene **13** with Pd(TFA)$_2$ and KO^{t}Bu led to the formation of the 3 principal compounds **8**, **9** and **10**. The ratio of **8**:**9** [1:1.03] suggested that both radical and organometallic pathways contributed to C-C bond formation.

<u>Reaction of 2-Iodo-*m*-xylene **7** with Pd(dppf)Cl$_2$ and KO^{t}Bu (Table 1, Entry 10)</u>

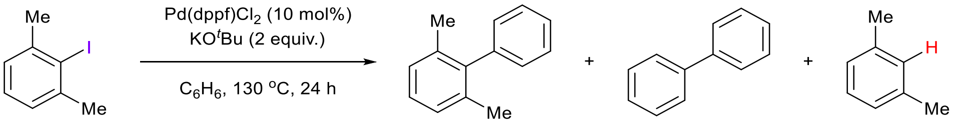

7 **8** **9** **10**

The reaction was conducted according to General Procedure A with Pd(dppf)Cl_2 (51 mg, 0.07 mmol, 10 mol%), KO^{t}Bu (157 mg, 1.4 mmol, 2 equiv.) and benzene.

GCFID data follow, including table quantitating components that had been calibrated (for calibrations see pages S73 - S77).

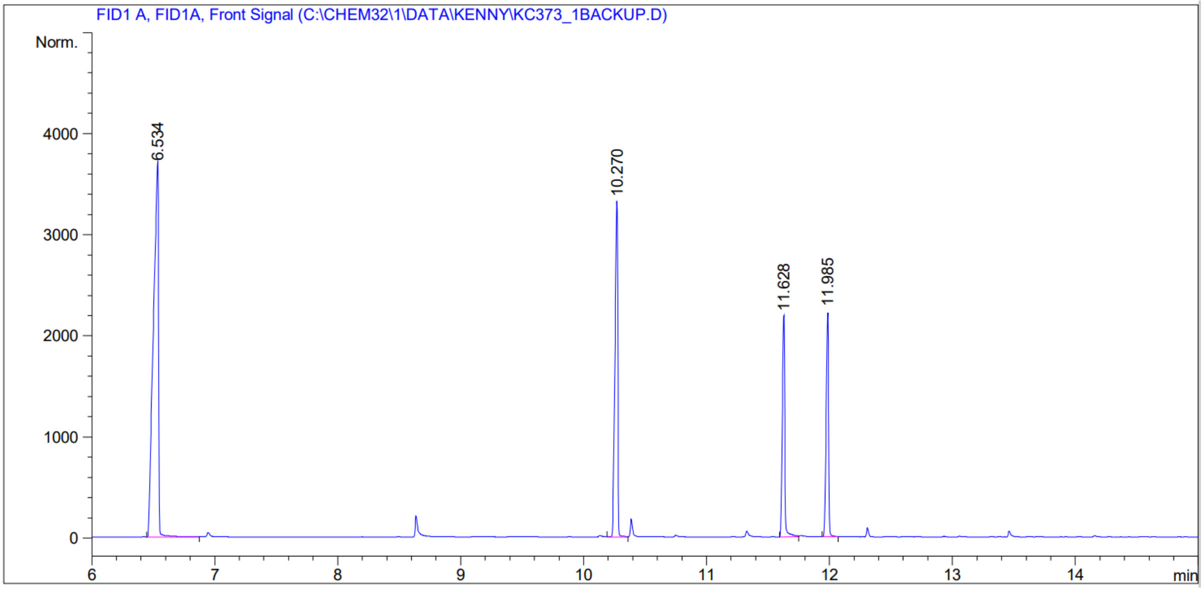

Retention Time	Sample	Peak Area	%Yield
6.534	*m*-Xylene **10**	10887.9	43.2%
10.270	Dodecane	5232.24414	N/A
11.628	Biphenyl **9**	3100.19043	8.2%
11.985	2,6-Dimethylbiphenyl **8**	3070.04272	7.2%

*This reaction was carried out in duplicate with the average below

Sample	%Yield
m-Xylene **10**	42.5%
2-Iodo-*m*-xylene **7**	0
Biphenyl **9**	8%
2,6-Dimethylbiphenyl **8**	7%

Outcome – The reaction of 2-Iodo-*m*-xylene **7** with Pd(dppf)Cl_2 and KO^{t}Bu led to the formation of the 3 principal compounds **8**, **9** and **10**. The ratio of **8**:**9** [1:1.14] suggested that both radical and organometallic pathways contributed to C-C bond formation.

<u>Reaction of 2-Iodo-*m*-xylene **7** with Pd(OAc)$_2$, KO^{t}Bu and dtbdppf (Table 2, Entry 2)</u>

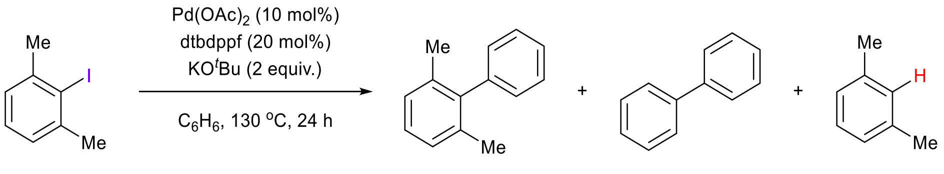

7 **8** **9** **10**

The reaction was conducted according to General Procedure A with $Pd(OAc)_2$ (16 mg, 0.07 mmol, 10 mol%), dtbdppf (72 mg, 0.14 mmol, 20 mol%), KO^{t}Bu (157 mg, 1.4 mmol, 2 equiv.) and benzene.

GCFID data follow, including tables quantitating components that had been previously calibrated (for calibrations see pages S73 - S77).

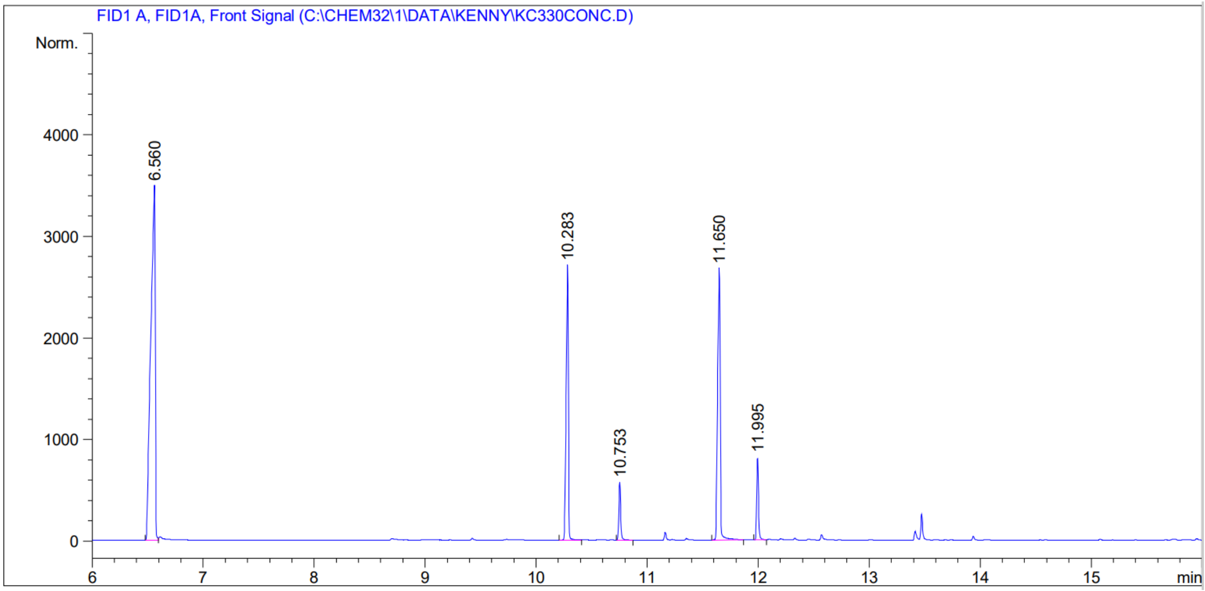

Retention Time	Sample	Peak Area	%Yield
6.560	*m*-Xylene **10**	9761.95898	44.5%
10.283	Dodecane	3787.19434	N/A
10.753	2-Iodo-*m*-xylene **7**	650.67633	3.1%
11.650	Biphenyl **9**	4342.70557	13.9%
11.995	2,6-Dimethylbiphenyl **8**	938.10687	2.5%

*This reaction was carried out in duplicate with the average below

Sample	%Yield
m-Xylene **10**	42.9%
2-Iodo-*m*-xylene **7**	4.7%
Biphenyl **9**	12.8%
2,6-Dimethylbiphenyl **8**	2.5%

Outcome - The reaction of 2-Iodo-*m*-xylene **7** with $Pd(OAc)_2$, KO^{t}Bu and dtbdppf led to the formation of the 3 principal compounds **8**, **9** and **10**. The ratio of **8**:**9** [1:5.12] indicated that BHAS chemistry played the major role in C-C bond formation.

Reaction of 2-Iodo-*m*-xylene **7** with $Pd(OAc)_2$, KO^{t}Bu and dppf (Table 2, Entry 3)

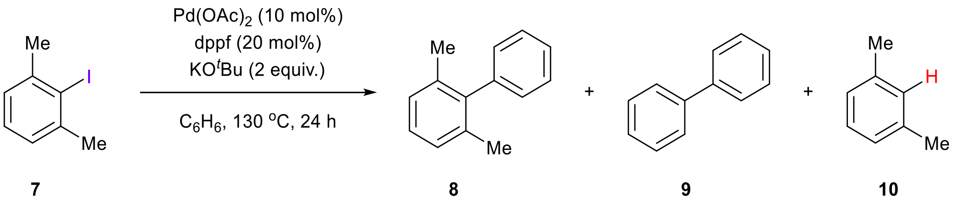

The reaction was conducted according to General Procedure A with $Pd(OAc)_2$ (16 mg, 0.07 mmol, 10 mol%), dppf (79 mg, 0.14 mmol, 20 mol%), KO^{t}Bu (157 mg, 1.4 mmol, 2 equiv.) and benzene.

GCFID data follow below, including tables quantitating components that had been calibrated (for calibrations see pages S73 - S77

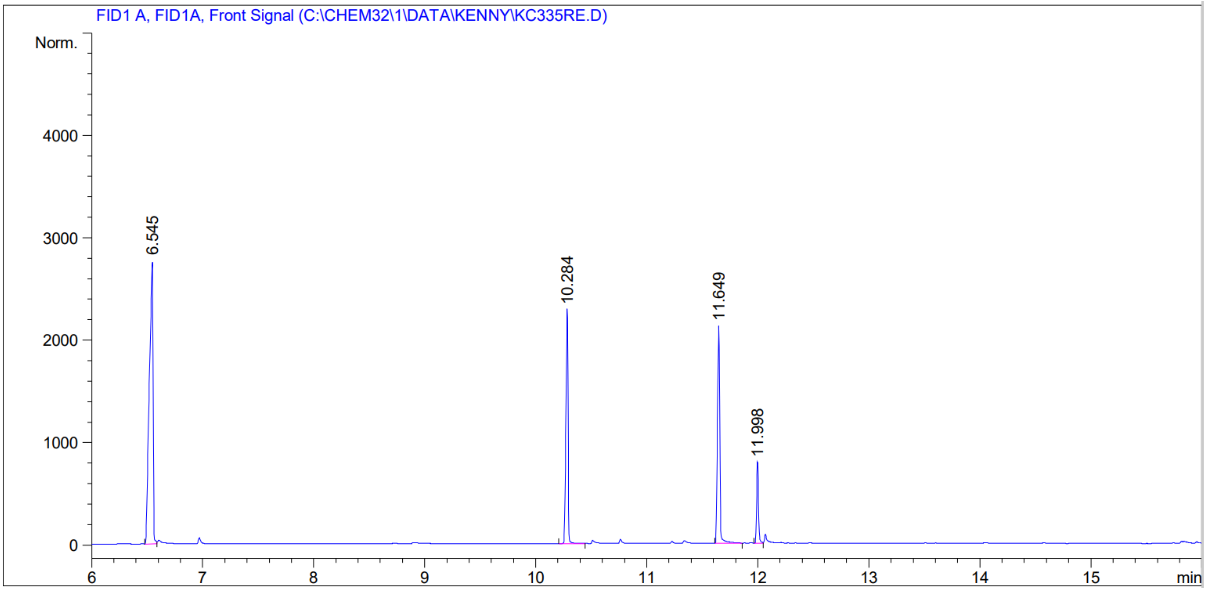

Retention Time	Sample	Peak Area	%Yield
6.545	*m*-Xylene **10**	6430.45459	36.2%
10.284	Dodecane	3109.32690	N/A
11.649	Biphenyl **9**	3136.57227	12.4%
11.998	2,6-Dimethylbiphenyl **8**	923.89362	3.1%

*This reaction was carried out in duplicate with the average below

Sample	%Yield
m-Xylene **10**	34.6%
2-Iodo-*m*-xylene **7**	0
Biphenyl **9**	12.4%
2,6-Dimethylbiphenyl **8**	3.1%

Outcome - The reaction of 2-iodo-*m*-xylene **7** with $Pd(OAc)_2$, KO^{t}Bu and dppf led to the formation of the 3 principal compounds **8**, **9** and **10**. The ratio of **8**:**9** [1:4.0] indicated that BHAS chemistry played the major role in C-C bond formation.

Reaction of 2-Iodo-*m*-xylene **7** with $Pd(OAc)_2$, KO^tBu and dippf (Table 2, Entry 4)

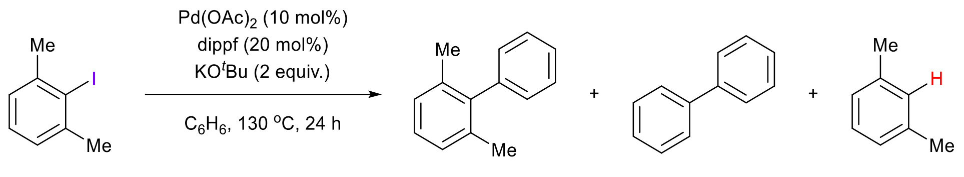

The reaction was conducted according to General Procedure A with $Pd(OAc)_2$ (16 mg, 0.07 mmol, 10 mol%), dippf (59 mg, 0.14 mmol, 20 mol%), KO^tBu (157 mg, 1.4 mmol, 2 equiv.) and benzene.

GCFID data follow, including tables quantitating components that had been calibrated (for calibrations see pages S73 - S77.

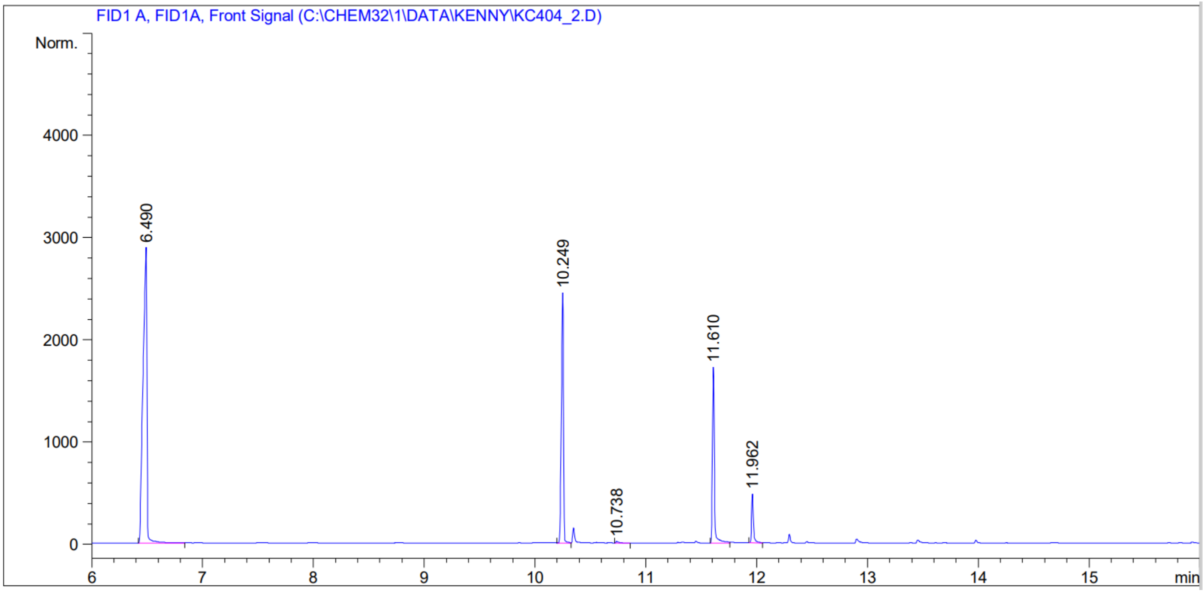

Retention Time	Sample	Peak Area	%Yield
6.490	*m*-Xylene **10**	7175.31299	34.0%
10.249	Dodecane	3257.24609	N/A
10.738	2-Iodo-*m*-xylene **7**	27.97805	0.1%
11.610	Biphenyl **9**	2187.68213	7.0%
11.962	2,6-Dimethylbiphenyl **8**	577.20532	1.6%

*This reaction was carried out in duplicate with the average below

Sample	%Yield
m-Xylene **10**	41.6%
2-Iodo-*m*-xylene **7**	0.2%
Biphenyl **9**	8.8%
2,6-Dimethylbiphenyl **8**	2.0%

Outcome - The reaction of 2-iodo-*m*-xylene **7** with $Pd(OAc)_2$, KO^tBu and dippf led to the formation of the 3 principal compounds **8**, **9** and **10**. The ratio of **8**:**9** [1:4.4] indicated that BHAS chemistry played the major role in C-C bond formation.

Preparation of 1,1'-Bis(di-*p*-tolylphosphino)ferrocene **S4**(needed for Table 2, Entry 5)

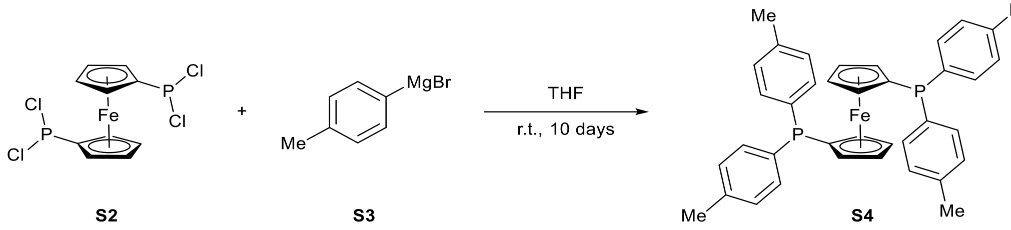

This reaction was carried out according to a modified literature procedure.[3] To an oven-dried flask, equipped with a stirrer bar and backfilled with N_2, was added bis(dichlorophosphino)ferrocene **S2** (500 mg, 1.3 mmol, 1 equiv.) and dry THF (20 mL). The mixture was cooled to 0 ^oC and 0.5 M *p*-tolylmagnesium bromide **S3** (15.5 mL, 7.8 mmol, 6 equiv.) was added dropwise and the solution stirred at room temperature for 10 days. Once complete, the mixture was quenched with water and extracted into Et_2O, passed through a phase separator, with the organic layers concentrated *in vacuo*. The mixture was purified by column chromatography (100% hexane) to yield 1,1'-bis(di-*p*-tolylphosphino)ferrocene **S4** as a viscous orange/yellow oil which crystallised on standing (550 mg, 0.90 mmol, 69%). **^{1}H NMR** (400 MHz, CD_2Cl_2) δ 7.22 – 7.16 (m, 8H), 7.13 – 7.08 (m, 8H), 4.25 – 4.23 (m, 4H), 3.97 (dd, *J* = 3.6, 1.8 Hz, 4H), 2.33 (s, 12H). **^{13}C NMR** (101 MHz, CD_2Cl_2) δ 138.8, 136.2 (d, *J* = 9.3 Hz), 133.7 (d, *J* = 19.9 Hz), 129.2 (d, *J* = 7.2 Hz), 77.7 (d, *J* = 7.9 Hz), 74.0 (d, *J* = 14.6 Hz), 72.7 (d, *J* = 2.6 Hz), 21.4. **^{31}P NMR** (162 MHz, CD_2Cl_2) δ -18.02. **ATR-IR** ν_{max} (neat)/cm^{-1} 2918, 1495, 1161, 1018, 802, 754, 712. **HRMS** (ESI+) [*m/z*] calcd. for $C_{38}H_{37}FeP_2$ $(M+H)^+$ 611.1714, found 611.1703. **M.p**: 130-132 ^oC.

Reaction of 2-Iodo-*m*-xylene **7** with $Pd(OAc)_2$, KO^{t}Bu and dtolppf (Table 2, Entry 5**)**

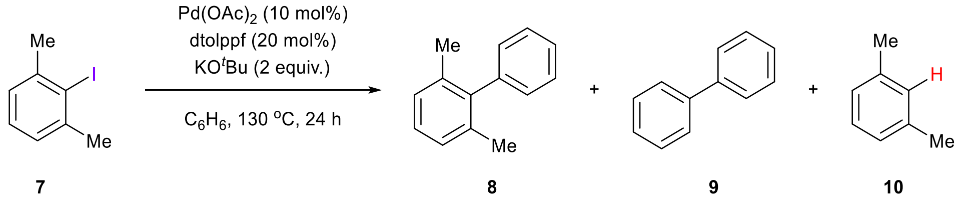

The reaction was conducted according to General Procedure A with $Pd(OAc)_2$ (16 mg, 0.07 mmol, 10 mol%), dtolppf (85 mg, 0.14 mmol, 20 mol%), KO^{t}Bu (157 mg, 1.4 mmol, 2 equiv.) and benzene.

GCMS Chromatogram

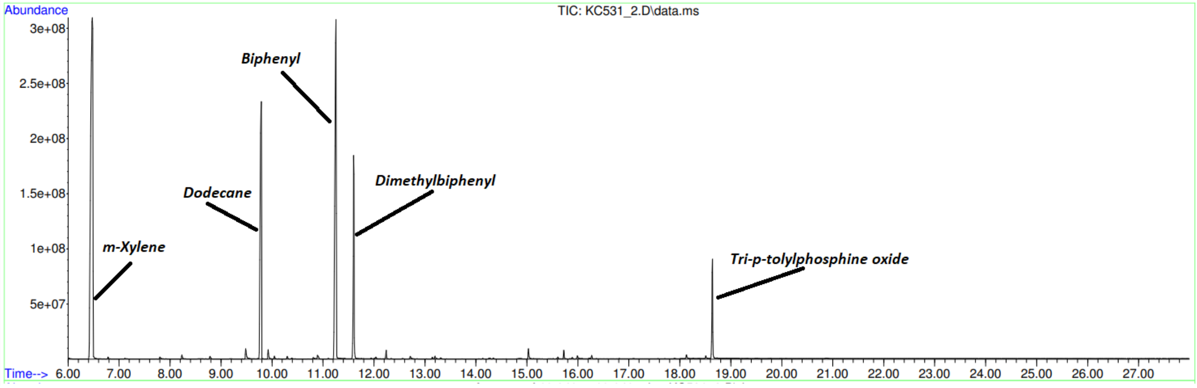

GCFID data are below, including tables quantitating components that had been quantitatively calibrated (for calibrations see pages S73 - S77).

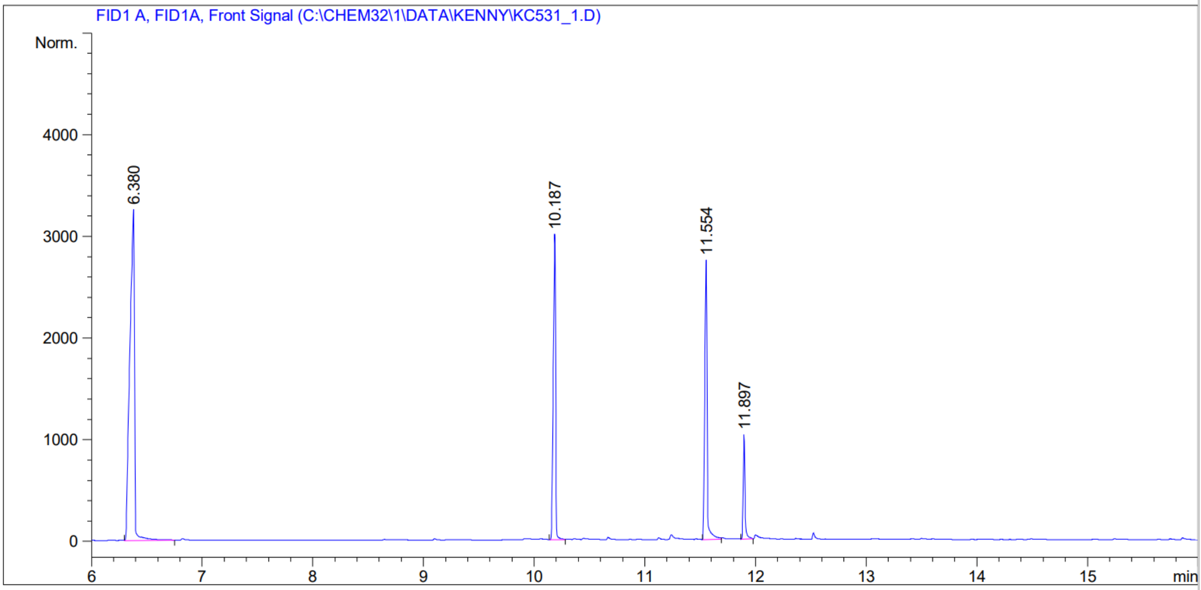

Retention Time	Sample	Peak Area	%Yield
6.380	*m*-Xylene **10**	9608.70898	41.5%
10.187	Dodecane	3787.19434	N/A
11.554	Biphenyl **9**	4342.70557	12.8%
11.897	2,6-Dimethylbiphenyl **8**	938.10687	3.0%

*This reaction was carried out in duplicate with the average below

Sample	%Yield
m-Xylene **10**	43.2%
2-Iodo-*m*-xylene **7**	0
Biphenyl **9**	13.1%
2,6-Dimethylbiphenyl **8**	3.1%

Outcome - The Reaction of 2-Iodo-*m*-xylene **7**with $Pd(OAc)_2$, KO^{t}Bu and dtolppf led to the formation of the 3 principal compounds **8**, **9** and **10**. The ratio of **8**:**9** [1:4.22] indicated that BHAS chemistry played the major role in C-C bond formation.

Preparation of 1,1′-Bis(di-*p*-(trifluoromethyl)phenylphosphino)ferrocene (dppf-CF_3) (needed for Table 2, entry 6)

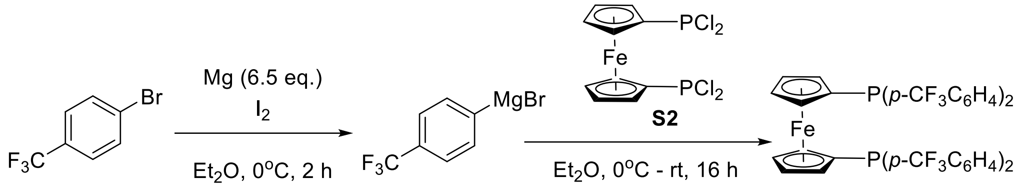

S5 **S6** **S7**

An oven-dried 3-neck round bottom flask was charged with Mg turnings (264.9 mg, 10.9 mmol, 6.5 eq.) and a crystal of I_2, then evacuated and backfilled with argon. Dry Et_2O (30 mL) was added via syringe and stirred for 10 min at room temperature before 4-bromobenzotrifluoride **S5** (1.17 mL, 8.38 mmol, 5 eq.) was added dropwise at 0°C. The resulting brown solution was allowed to stir for 2 h. A solution of bis(dichlorophosphino)ferrocene **S2** (645.0 mg, 1.68 mmol, 1 eq.) in Et_2O (15 mL) was then added dropwise at 0°C, and after the addition the solution was allowed to warm to room temperature and stirred overnight. The reaction was then quenched with H_2O (20 mL) and extracted into Et_2O. The organics were dried over $MgSO_4$, filtered and concentrated *in vacuo* yielding a dark red oil. Purification by column chromatography (50% DCM/hexane) yielded the product as an orange crystalline solid (752.6 mg, 0.913 mmol, 54 %). **^{1}H NMR** (400 MHz, CD_2Cl_2) δ 7.56 (d, *J* = 8.0 Hz, 2H), 7.41 (t, *J* = 7.4 Hz, 2H), 4.34 (t, *J* = 1.8 Hz, 1H), 4.01 (dd, *J* = 3.7, 1.9 Hz, 1H). **^{13}C NMR** (101 MHz, CD_2Cl_2) δ 143.8 (d, *J* = 13.8 Hz), 134.1 (d, *J* = 19.9 Hz), 131.0 (q, *J* = 32.2 Hz), 125.6 – 125.3 (m), 124.5 (q, *J* = 272.1 Hz), 75.3 (d, *J* = 7.9 Hz), 74.3 (d, *J* = 15.5 Hz), 73.2 (d, *J* = 3.6 Hz). **^{31}P NMR** (162 MHz, CD_2Cl_2) δ -16.97 (s). **^{19}F NMR** (376 MHz, CD_2Cl_2) δ -63.10 (s). The data for this compound are consistent with those reported in the literature.[4]

Reaction of 2-Iodo-*m*-xylene **7** with $Pd(OAc)_2$, KO^{t}Bu and dppf^{CF3} (Table 2, Entry 6).

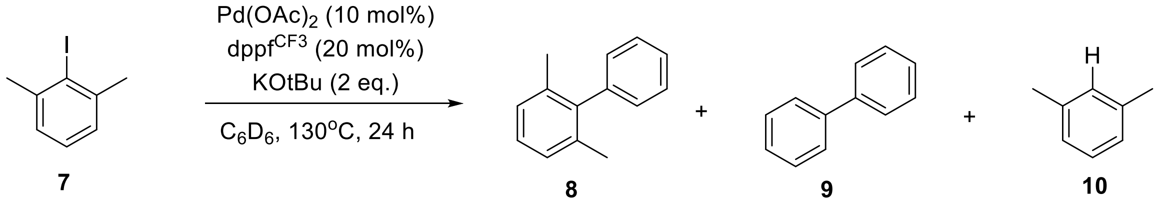

7 **8** **9** **10**

The reaction was conducted according to General Procedure A with $Pd(OAc)_2$ (16 mg, 0.07 mmol, 0.1 equiv.), dppf^{CF3} **S7** (115 mg, 0.14 mmol, 0.2 equiv.), KO*t*Bu (157 mg, 1.4 mmol, 2 equiv.) and benzene (5 mL).

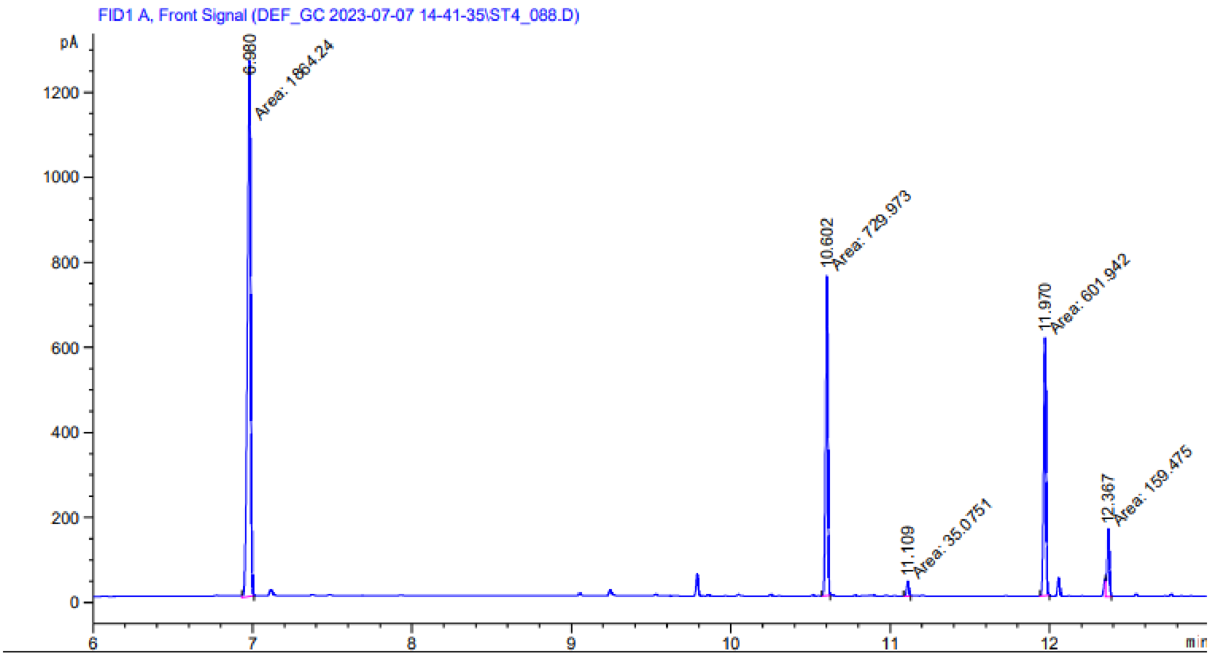

Retention time	Sample	Peak area	% Yield
6.960	*m*-xylene	1864.2	24.1
10.602	*n*-dodecane	729.973	N/A
11.109	Iodoxylene	35.0751	0.8
11.970	Biphenyl	601.942	9.9
12.367	2,6-Dimethylbiphenyl	159.475	2.4

<u>Reaction of 2-Iodo-*m*-xylene **7** with $Pd(OAc)_2$, KO^tBu and $Fc(PCy_2)_2$ in C_6H_6 and C_6D_6 (Table 2, Entry 7).</u>

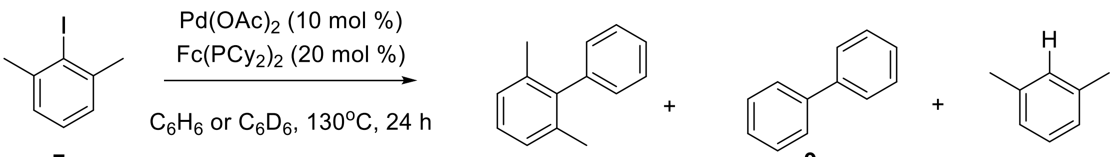

The reaction was conducted according to General Procedure A with $Pd(OAc)_2$ (7.9 mg, 0.035 mmol, 0.1 equiv.), $Fc(PCy_2)_2$ (40.5 mg, 0.07 mmol, 0.2 equiv.), KOtBu (79 mg, 0.7 mmol, 2 equiv.) and benzene (2.5 mL).

Retention time	Sample	Peak area	% Yield
	C_6H_6		
6.973	*m*-xylene **10**	1178.74	24.9
10.600	*n*-dodecane	729.08	N/A

11.108	Iodoxylene **7**	7.18	0.2
11.966	Biphenyl **9**	314.39	5.0
12.366	2,6-Dimethylbiphenyl **8**	94.81	1.4
	**C_6D_6**		
6.973	*m*-xylene **10**	1207.01	22.5
10.601	*n*-dodecane	824.63	N/A
11.108	Iodoxylene **7**	9.82	0.2
11.938	Biphenyl **9**	41.62	0.6
12.355	2,6-Dimethylbiphenyl **8**	96.47	1.2

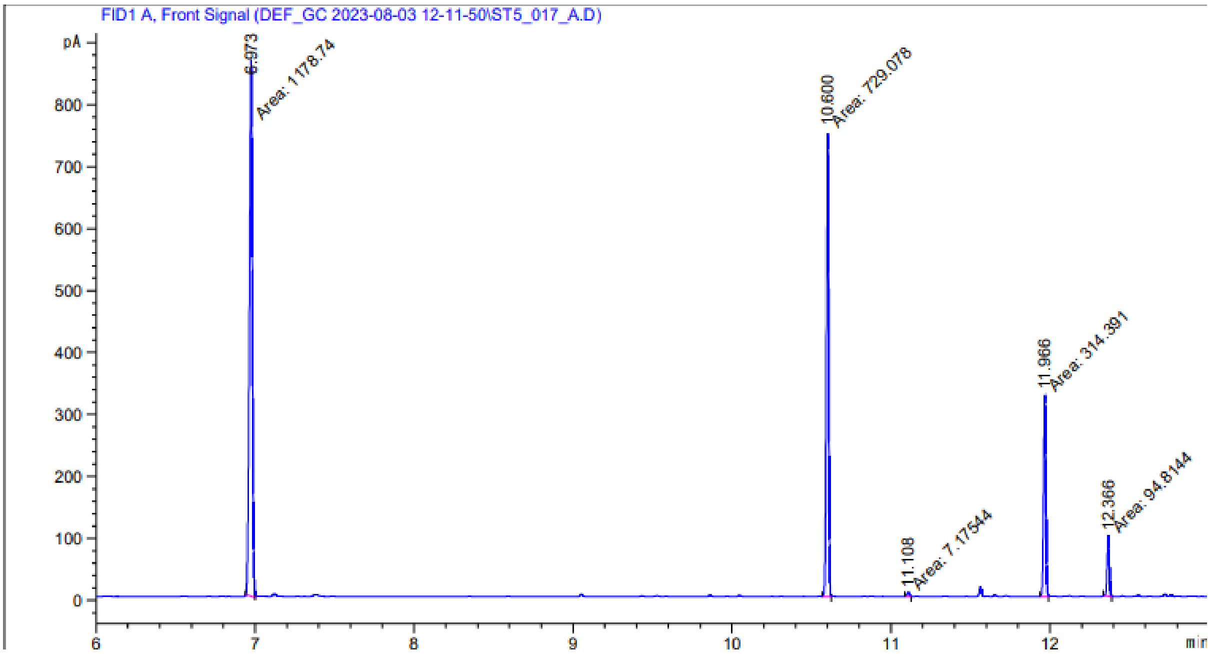

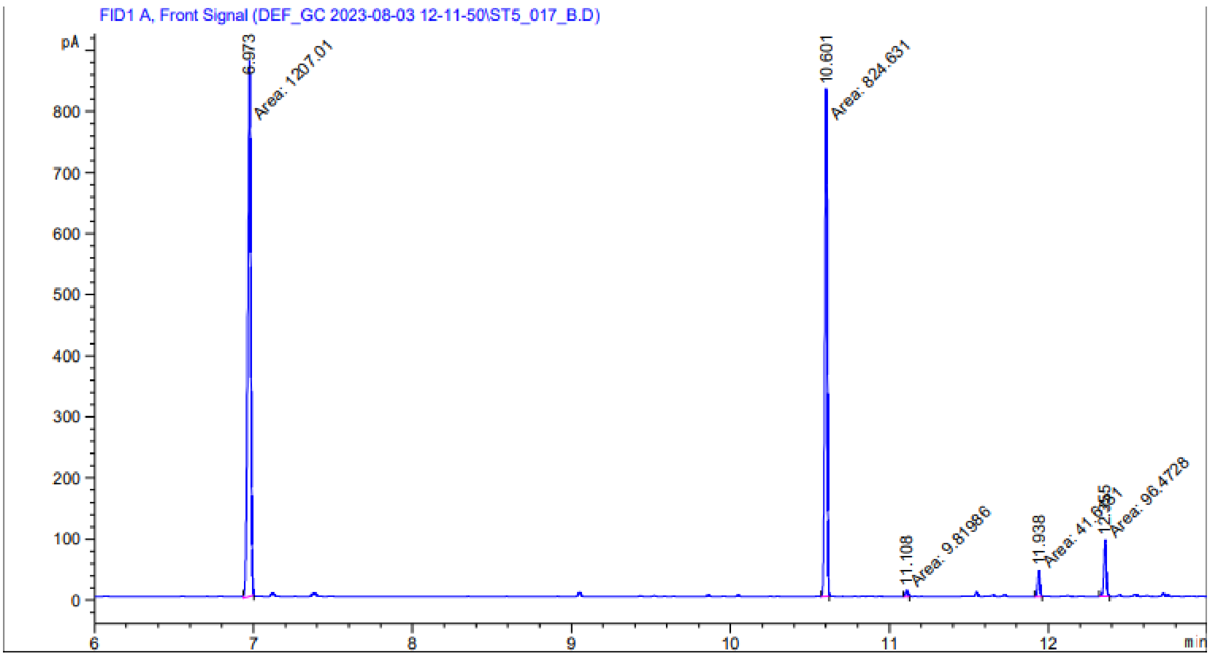

Xylene was completely non-deuterated, while both biphenyl and dimethylbiphenyl were deuterated (*m/z* 164 and 187 respectively). Based on this, $Fc(PCy_2)_2$ promotes BHAS chemistry as seen with other phosphinoferrocenes.

Also a peak was seen for d_5-PhCy, indicating liberation of a cyclohexyl radical from a phosphine and BHAS coupling of that radical with C_6D_6.

Synthesis of $FcPPh_2$[4] (needed for Table 2, entry 8)

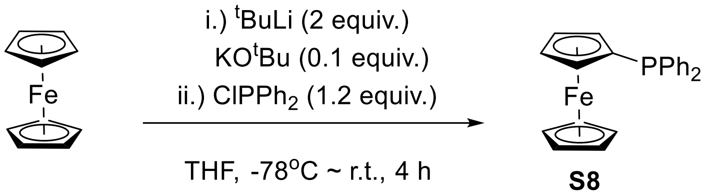

An oven-dried microwave vial was charged in a glovebox with ferrocene (259 mg, 1.4 mmol, 1 eq.), KO^{t}Bu (15.7 mg, 0.14 mmol, 0.1 eq.) and dry THF (3 mL) with a stirbar giving a clear orange solution. The vial was capped, removed from the glovebox and stirred at -78°C in a dry ice/acetone bath giving an orange suspension. A 1.7 M solution of ^{t}BuLi in pentane (1.65 mL, 2.8 mmol, 2 eq.) was added dropwise and left to stir for 15 minutes, with no observable changes. The reaction was then warmed to -20°C in a 35% MeOH/H_2O bath with dry ice and stirred for 5 minutes, before a solution of $ClPPh_2$ (0.306 mL, 1.68 mmol, 1.2 eq.) in THF (3 mL) was added dropwise under Ar over 20 minutes. After stirring for 20 minutes, the solution was allowed to warm to room temperature and stirred for a further 2 h. The reaction was then quenched with H_2O (10 mL) and diluted with DCM (15 mL). The aqueous layers were extracted with DCM (3 x 10 mL), and the combined organic layers were then

dried over $MgSO_4$, filtered and concentrated to give a viscous red oil. Purification by column chromatography (10% EtOAc/hexane) and repeated recrystallization from DCM layered with pentane at -34°C yielded $FcPPh_2$ **S8** as an orange solid (260.4 mg, 0.704 mmol, 50 %). **^{1}H NMR** (400 MHz, $CDCl_3$) δ 7.39 – 7.34 (m, 4H), 7.33 – 7.29 (m, 6H), 4.37 (t, *J* = 1.8 Hz, 2H), 4.10 (dd, *J* = 3.7, 1.9 Hz, 2H), 4.07 (s, 5H). **^{31}P NMR** (162 MHz, $CDCl_3$) δ -16.20. **^{13}C NMR** (101 MHz, $CDCl_3$) δ 139.3 (d, *J* = 9.3 Hz), 133.6 (d, *J* = 19.3 Hz), 128.6 (s), 128.3 (d, *J* = 6.7 Hz), 76.0 (d, *J* = 5.9 Hz), 73.0 (d, *J* = 15.0 Hz), 70.9 (d, *J* = 3.7 Hz), 69.2. ***m/z*** (EI) 370.1 ($[M]^+$). Data agrees with previous literature values.[4]

Reaction of 2-Iodo-*m*-xylene **7** with $Pd(OAc)_2$, KO^tBu and $FcPPh_2$ in C_6H_6 (Table 2, Entry 8)

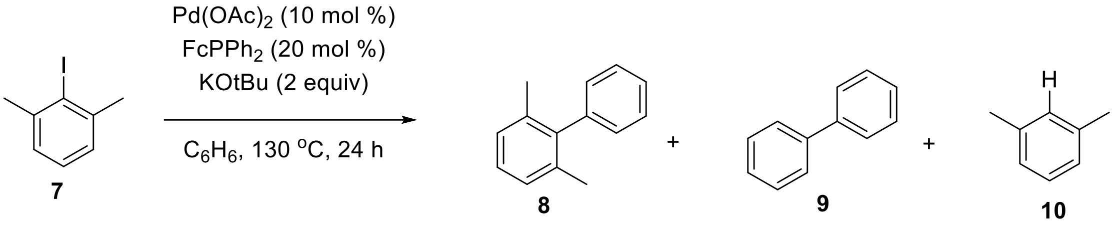

The reaction was conducted according to General Procedure A with $Pd(OAc)_2$ (16 mg, 0.07 mmol, 0.1 equiv.), $FcPPh_2$ (52 mg, 0.14 mmol, 0.2 equiv.), KOtBu (157 mg, 1.4 mmol, 2 equiv.) and benzene (5 mL).

**Retention time**	**Sample**	**Peak area**	**% Yield**
6.973	*m*-xylene	2074.24	43.1
10.601	*n*-dodecane	775.42	N/A
11.109	Iodoxylene	0	0
11.965	Biphenyl	410.56	6.4
12.368	2,6-Dimethylbiphenyl	166.32	2.4

This reaction was carried out in duplicate with the average below

**Sample**	**%Yield**
m-Xylene **10**	42.8
2-Iodo-*m*-xylene **7**	0
Biphenyl **9**	6.6
2,6-Dimethylbiphenyl **8**	2.3

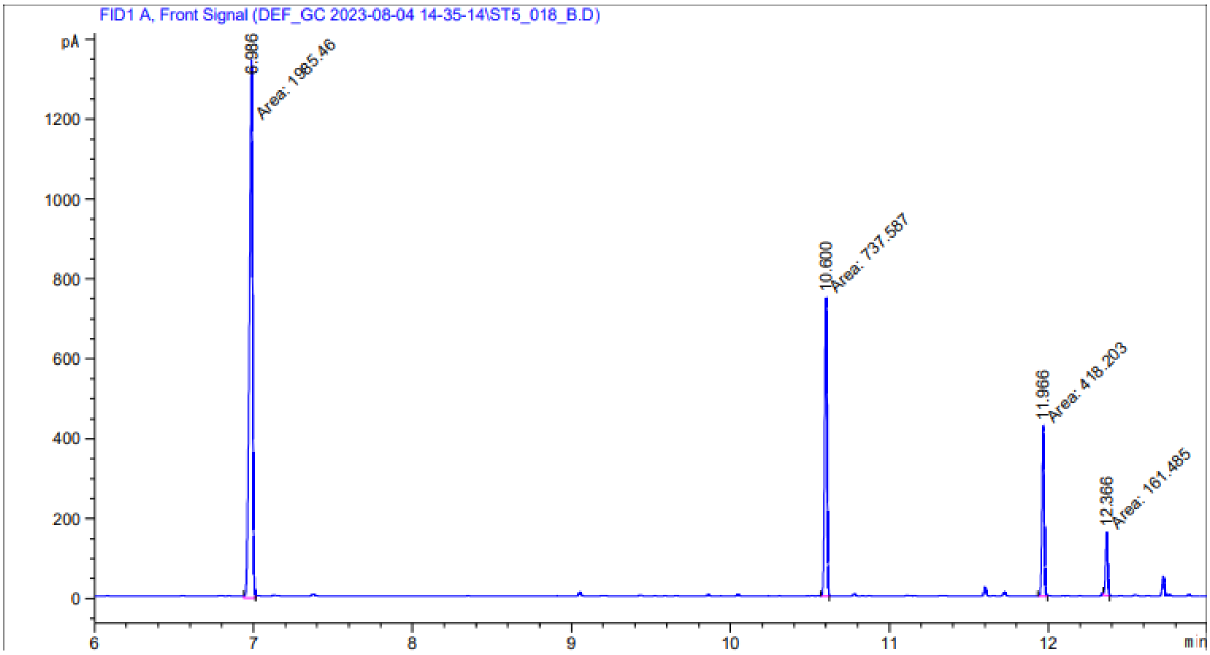

Reaction of 2-Iodo-*m*-xylene **7** with $Pd(OAc)_2$, KO^tBu and $FcPPh_2$ in C_6D_6 (not in Tables but related to Table 2, entry 8)

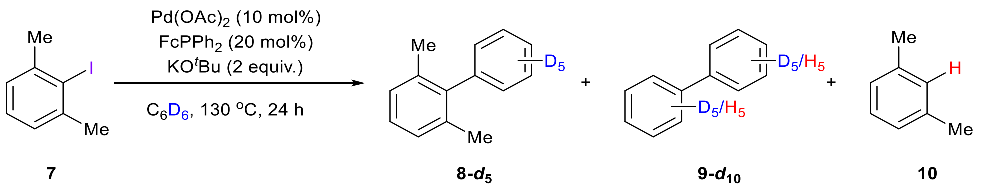

The reaction was conducted according to General Procedure A with $Pd(OAc)_2$ (16 mg, 0.07 mmol, 0.1 equiv.), $FcPPh_2$ (52 mg, 0.14 mmol, 0.2 equiv.), KOtBu (157 mg, 1.4 mmol, 2 equiv.) and d_6-benzene (5 mL).

Retention time	Sample	Peak area	% Yield
6.986	*m*-xylene	1708.70	39.2
10.601	*n*-dodecane	675.35	N/A
11.109	Iodoxylene	0	0
11.939	Biphenyl-d_{10}	42.22	0.73
11.965	Biphenyl-d_0	39.53	0.68
	Total Biphenyl	81.75	1.4
12.357	2,6-Dimethylbiphenyl	147.26	2.3

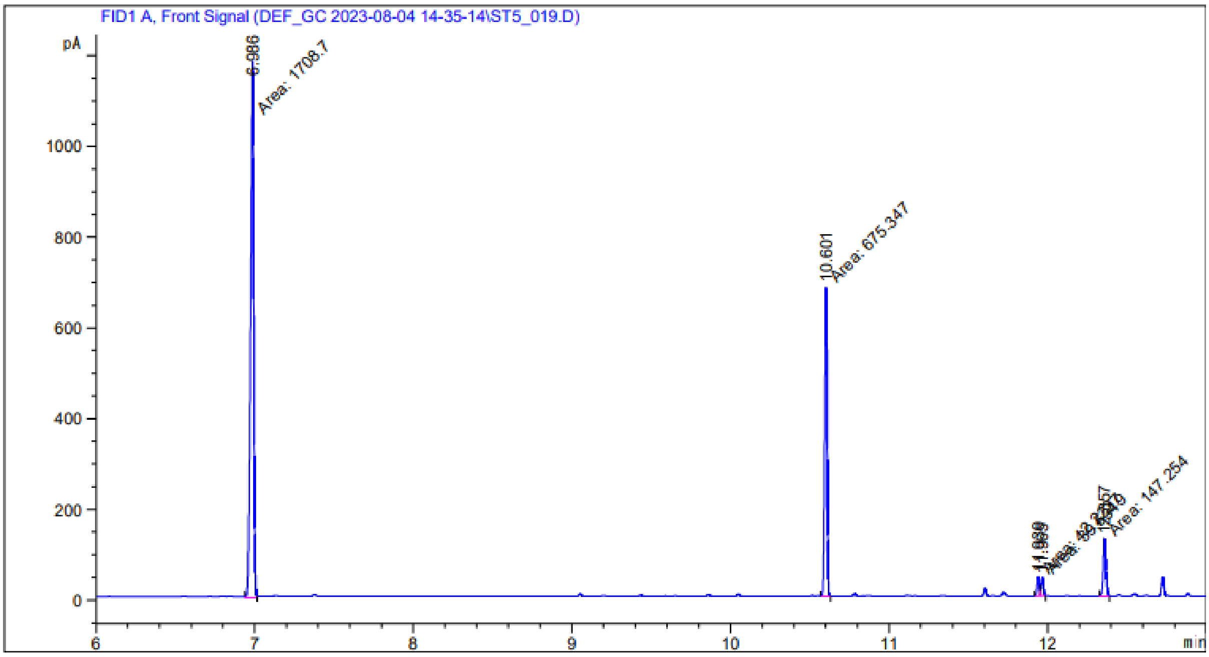

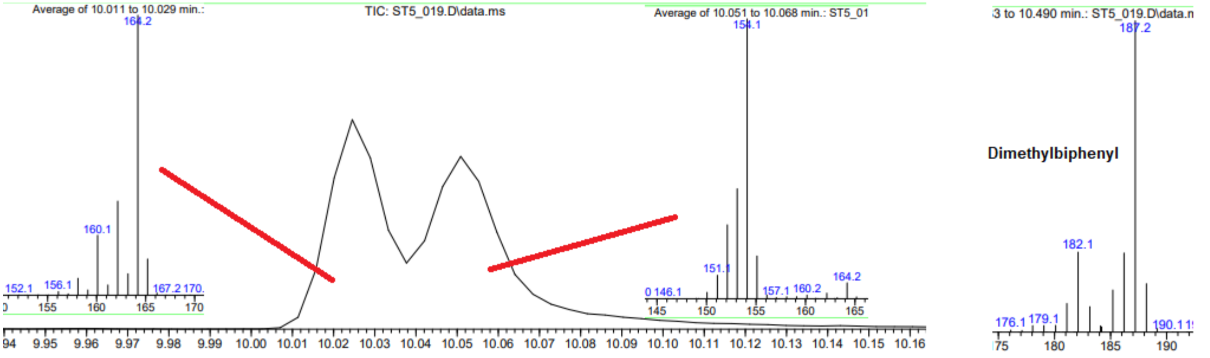

By GCMS we see a mixture of d_{10} (m/z 164) and d_0 biphenyl (m/z 154) in well-defined peaks , indicating that d_{10} is formed by BHAS and d_0 by another process (involving aryl groups from phosphine ligands). There is no biphenyl-d_5 showing that the two processes are independent.

The dimethylbiphenyl is mostly d_5, (m/z 187) formed by BHAS of dimethylphenyl radicals with solvent C_6D_6, with small amounts of d_0. (m/z 182) from coupling of dimethylphenyl groups with Ph groups on the ligands.

Reaction of 2-Iodo-*m*-xylene **7** with $Pd(OAc)_2$, KO^{t}Bu and BINAP (Table 2, Entry 9)

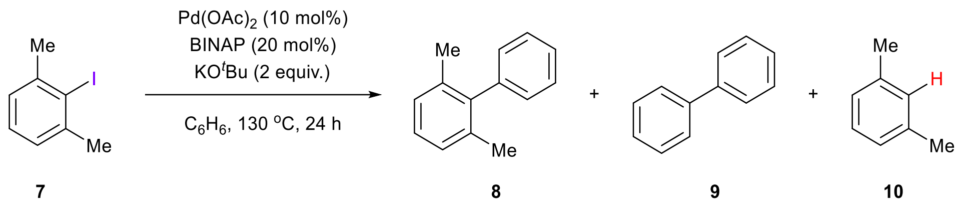

The reaction was conducted according to General Procedure A with $Pd(OAc)_2$ (16 mg, 0.07 mmol, 10 mol%), *rac*-BINAP (87 mg, 0.14 mmol, 20 mol%), KO^{t}Bu (157 mg, 1.4 mmol, 2 equiv.) and benzene.

GCFID data are below, including tables quantitating components that had been calibrated (for calibrations see pages S73 - S77).

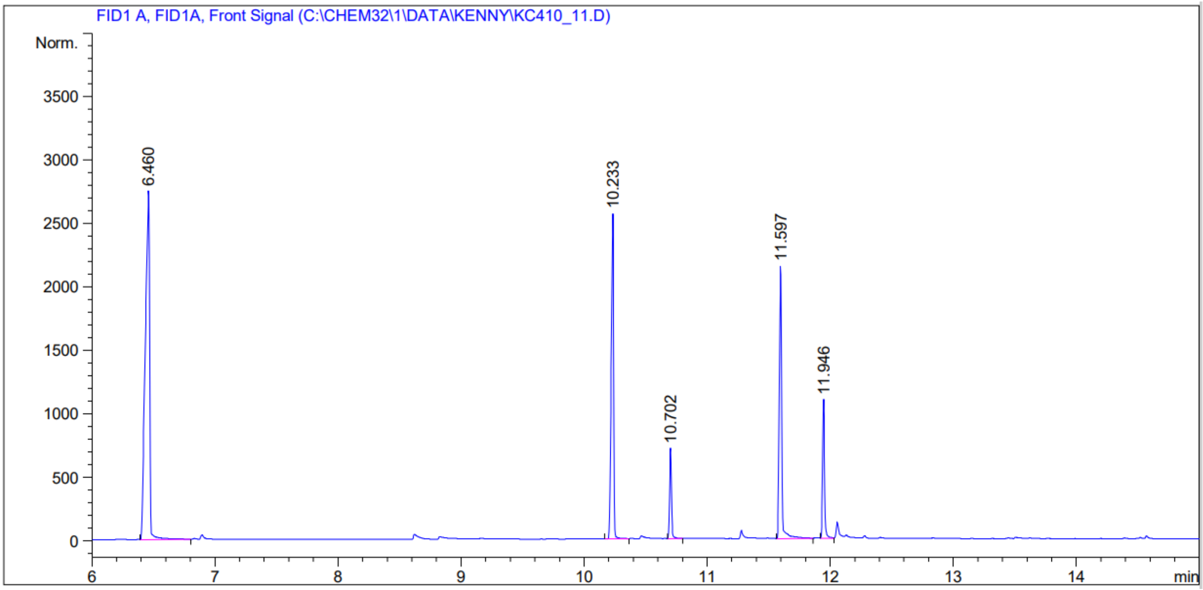

Retention Time	Sample	Peak Area	%Yield
6.460	*m*-Xylene **10**	6796.08057	36.7%
10.233	Dodecane	3544.30371	N/A
10.702	2-Iodo-*m*-xylene **7**	769.87628	4.4%
11.597	Biphenyl **9**	2959.44824	11.2%
11.946	2,6-Dimethylbiphenyl **8**	1226.73206	3.9%

*This reaction was carried out four times with the average below

Sample	%Yield
m-Xylene **10**	34.8%
2-Iodo-*m*-xylene **7**	4.1%

Biphenyl **9**	10.6%
2,6-Dimethylbiphenyl **8**	3.7%

Outcome - The reaction of 2-Iodo-*m*-xylene **7** with $Pd(OAc)_2$, KO^{t}Bu and BINAP led to the formation of the 3 principal compounds **8** - **10**. The ratio of **8**:**9** [1:2.92] indicated that a BHAS mechanism was the principal contributor to C-C bond formation .

<u>Reaction of 2-Iodo-*m*-xylene **7** with $Pd(OAc)_2$, KO^{t}Bu and PCy_3 (Table 2, Entry 10)</u>

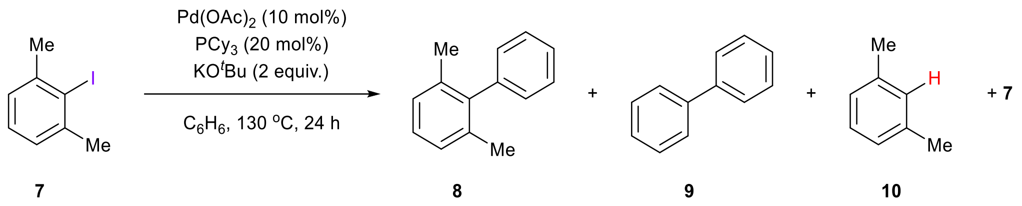

The reaction was conducted according to General Procedure A with $Pd(OAc)_2$ (16 mg, 0.07 mmol, 10 mol%), PCy_3 (39 mg, 0.14 mmol, 20 mol%), KO^{t}Bu (157 mg, 1.4 mmol, 2 equiv.) and benzene.

GCMS Chromatogram

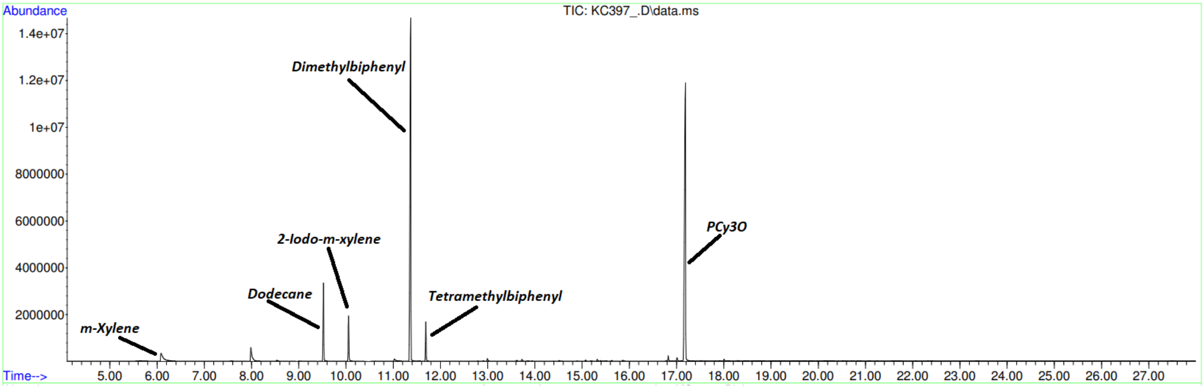

GCFID data are below including tables quantitating components that had been calibrated (for calibrations see pages S73 - S77

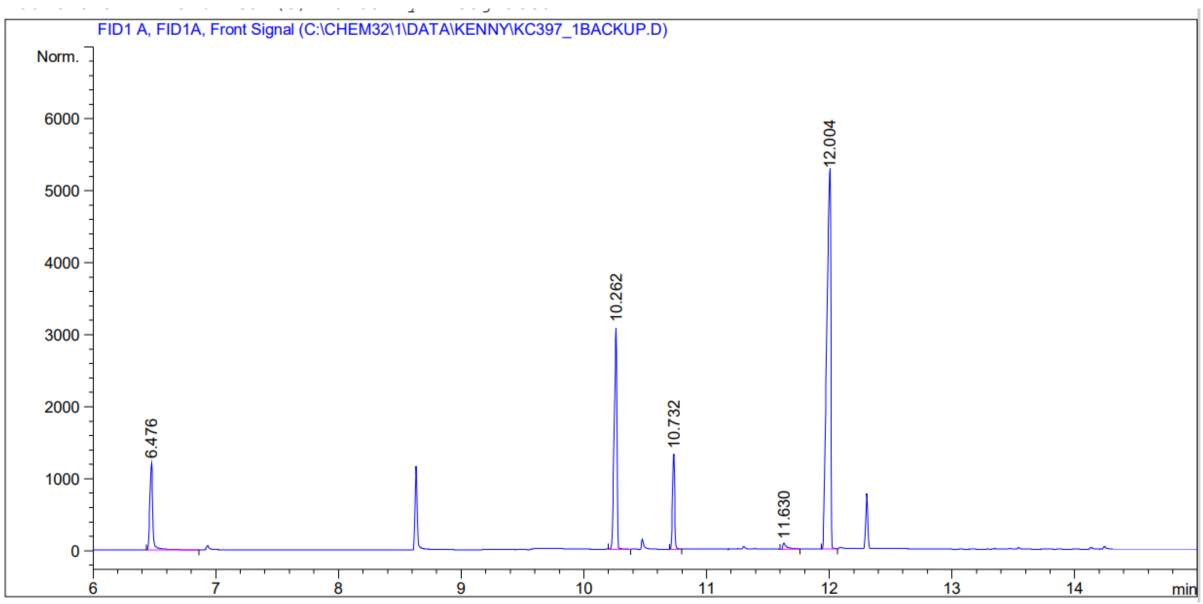

Retention Time	Sample	Peak Area	%Yield
6.476	*m*-Xylene **10**	2051.73340	8.7%
10.262	Dodecane	4778.66260	N/A
10.732	2-Iodo-*m*-xylene **7**	1602.30847	7.1%
11.630	Biphenyl **9**	203.67375	0.6%
12.004	2,6-Dimethylbiphenyl **8**	12004.7	29.8%

*This reaction was carried out in duplicate with the average below

Sample	%Yield
m-Xylene **10**	8.2%
2-Iodo-*m*-xylene **7**	6.9%
Biphenyl **9**	0.6%
2,6-Dimethylbiphenyl **8**	27.7%

Outcome - The reaction of 2-iodo-*m*-xylene **7** with $Pd(OAc)_2$, KO^tBu and PCy_3 led to the formation of the 3 principal compounds **8**, **9** and **10**. The ratio of **8**:**9** [11.5:1] indicated that an organometallic mechanism and was the main contributor to C-C bond formation The reaction of $Pd(OAc)_2$, KO^tBu and PCy_3 mirrors the result from the reaction with $Pd(PPh_3)_4$ and KO^tBu.

<u>Reaction of 2-Iodo-*m*-xylene **7** with $Pd(OAc)_2$, KO^tBu and $PtBu_3$ (Table 2, Entry 11)</u>

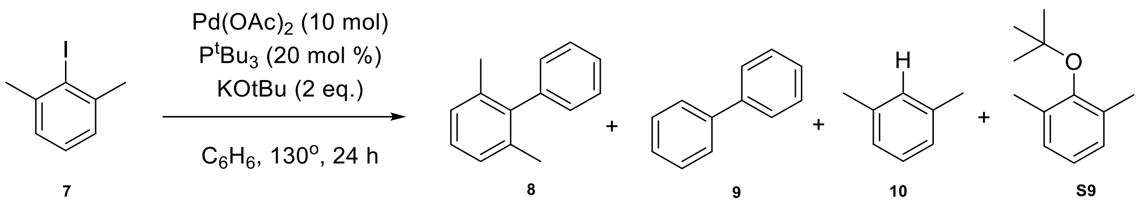

The reaction was conducted according to General Procedure A with $Pd(OAc)_2$ (15.7 mg, 0.07 mmol, 1 equiv.), P^tBu_3 (34 µL, 0.14 mmol, 0.2 equiv.), KOtBu (157 mg, 1.4 mmol, 2 equiv.) and benzene (5 mL). After analysis by GC, the crude mixture was then purified by column chromatography (5% EtOAc/hexane) yielding 2-*t*Bu-1,6-dimethylbenzene **S9** as a colourless oil (67 mg, 0.376, 53.7 %). **^{1}H NMR** (400 MHz, $CDCl_3$) δ 7.00 (d, *J* = 7.2 Hz, 2H), 6.89 (dd, *J* = 7.9, 6.9 Hz, 1H), 2.31 (s, 6H), 1.39 (s, 9H). **^{13}C NMR** (101 MHz, $CDCl_3$) δ 153.7, 133.4, 128.8, 123.2, 80.9, 29.9, 19.0. ***m/z* (EI):** 163.1 ([M-Me]$^+$).

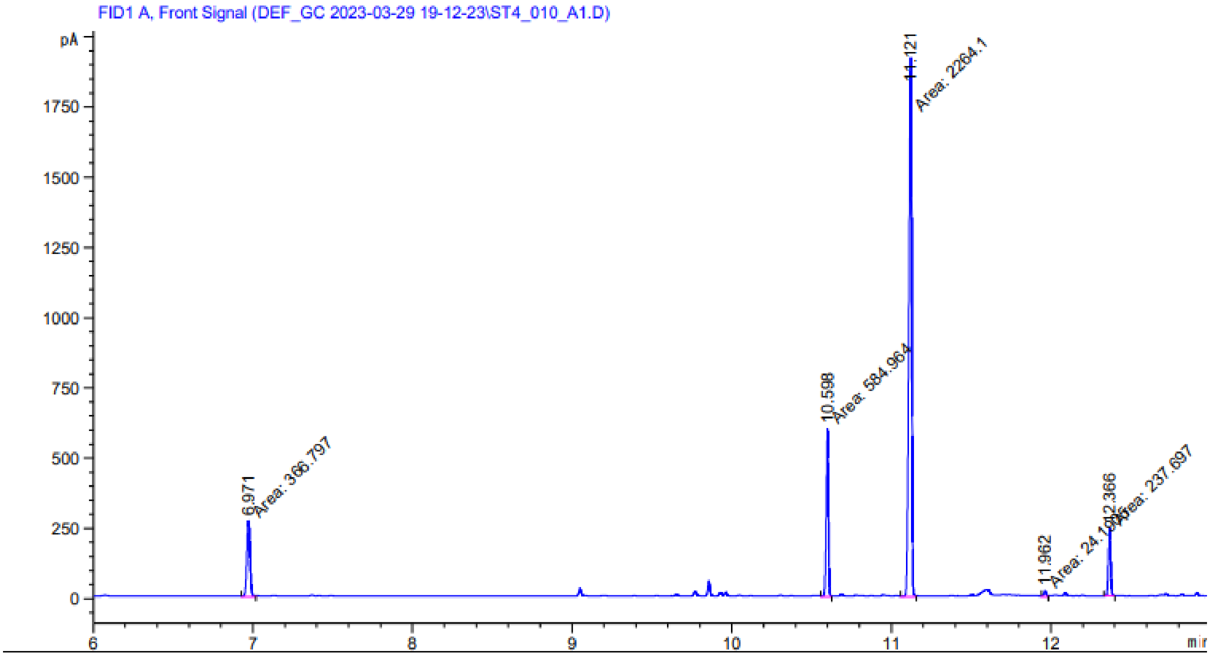

Retention time	Sample	Peak area	% Yield
6.971	*m*-xylene	366.80	14.3
10.598	*n*-dodecane	584.96	N/A
11.121	**S9**	2264.10	53.7*
11.962	Biphenyl	24.19	0.7
12.366	2,6-Dimethylbiphenyl	237.70	6.3

This reaction was carried out in duplicate with the average below

Sample	%Yield
m-Xylene **10**	14.5%
2,6-dimethyl-*t*-butoxybenzene **S9**	50.3%*
Biphenyl **9**	0.8%
2,6-Dimethylbiphenyl **8**	6.9%

*yields of **S9** obtained by column chromatography.

Reaction of 2-Iodo-*m*-xylene **7** with $Pd(OAc)_2$, KO^{t}Bu and dcpe (Table 2, Entry 12)

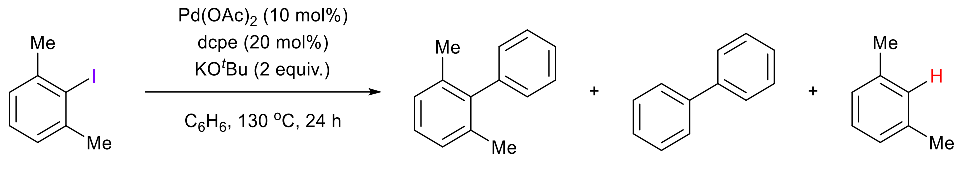

The reaction was conducted according to General Procedure A with $Pd(OAc)_2$ (16 mg, 0.07 mmol, 10 mol%), dcpe (59 mg, 0.14 mmol, 20 mol%), KO^{t}Bu (157 mg, 1.4 mmol, 2 equiv.) and benzene.

GCFID data including table quantitating components that had been calibrated (for calibrations see pages S73 - S77).

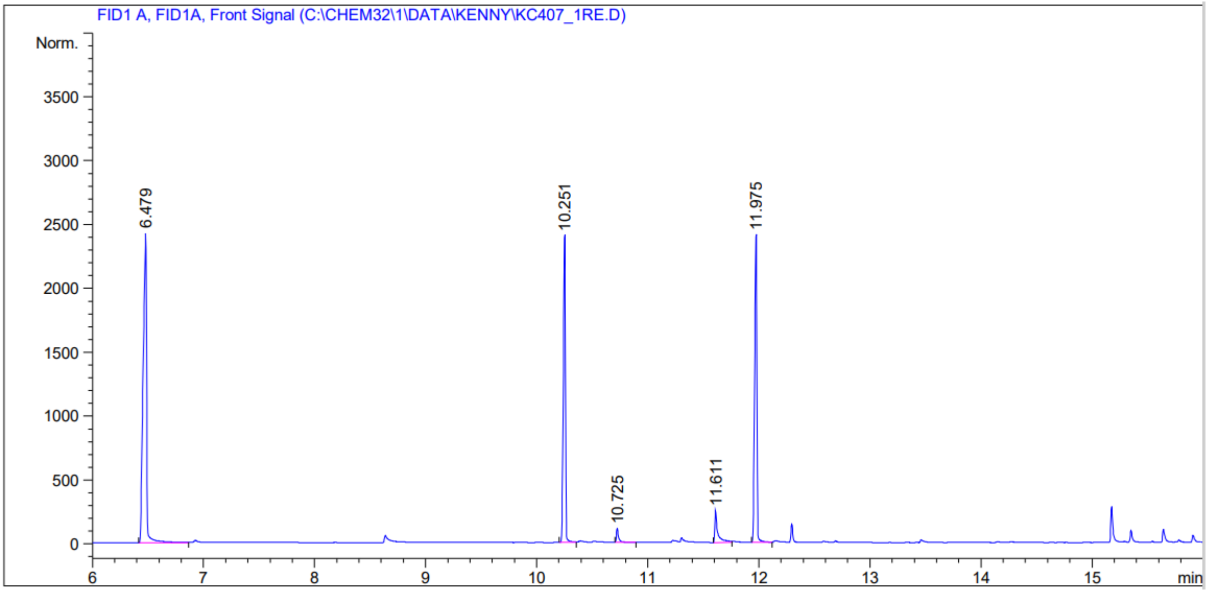

Retention Time	Sample	Peak Area	%Yield
6.479	*m*-Xylene **10**	5360.54639	31.7%
10.251	Dodecane	3252.98486	N/A
10.725	2-Iodo-*m*-xylene **7**	151.19849	0.9%
11.611	Biphenyl **9**	461.69077	1.9%
11.975	2,6-Dimethylbiphenyl **8**	3474.01294	12.1%

*This reaction was carried out in duplicate with the average below

Sample	%Yield
m-Xylene **10**	28.6%
2-Iodo-*m*-xylene **7**	0.9%
Biphenyl **9**	1.9%
2,6-Dimethylbiphenyl **8**	11.0%

Outcome - The reaction of 2-iodo-*m*-xylene **7** with Pd(OAc)$_2$, KO^{t}Bu and dcpe led to the formation of the 3 principal compounds **8**, **9** and **10**. The ratio of **8**:**9** [5.79:1] indicated that an organometallic mechanism was the principal contributor to C-C bond formation

<u>Reaction of 2-Iodo-*m*-xylene **7** with Pd(OAc)$_2$, KO^{t}Bu and PPh$_3$, (Table 2, Entry 13)</u>

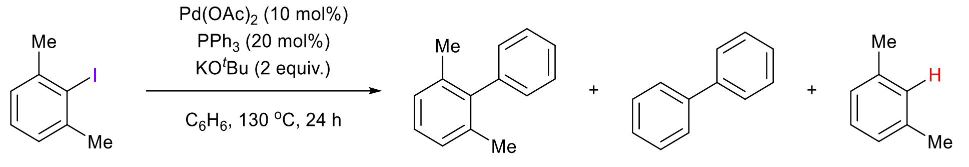

7 8 9 10

The reaction was conducted according to General Procedure A with Pd(OAc)$_2$ (16 mg, 0.07 mmol, 10 mol%), PPh$_3$ (37 mg, 0.14 mmol, 20 mol%), KO^{t}Bu (157 mg, 1.4 mmol, 2 equiv.) and benzene.

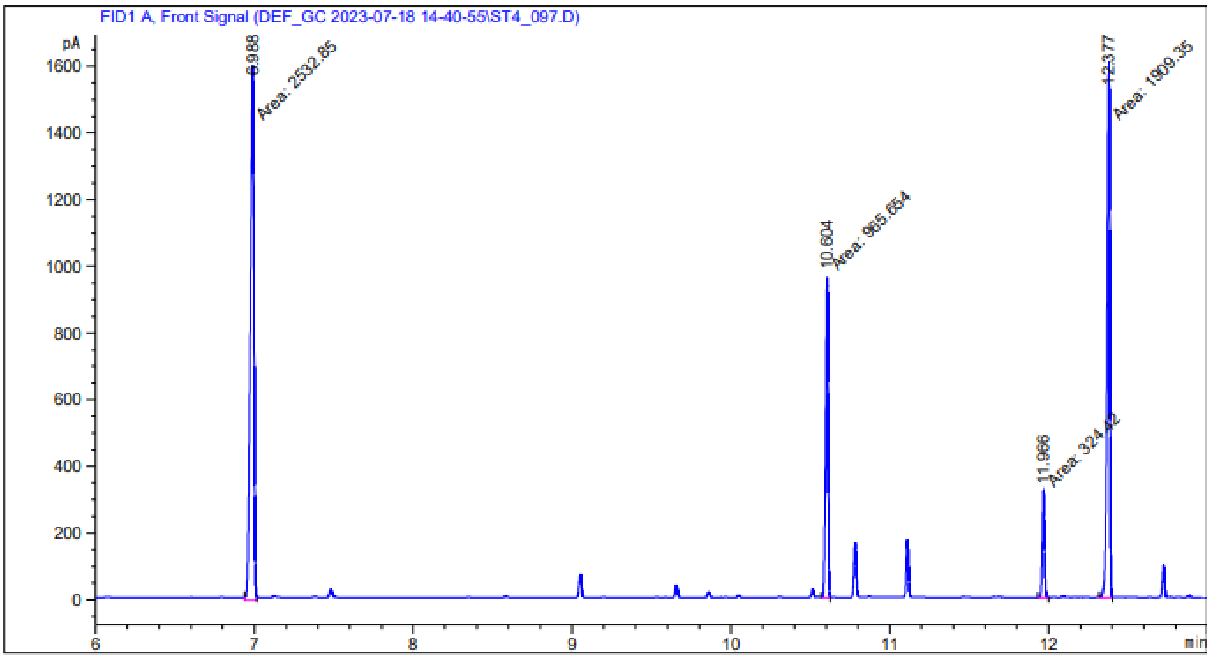

Retention Time	Sample	Peak Area	%Yield
6.988	*m*-Xylene **10**	2532.85	41.6
10.604	Dodecane	965.65	N/A
11.966	Biphenyl **9**	324.42	4.0
12.377	2,6-Dimethylbiphenyl **8**	1909.35	21.3

<u>Reaction of 2-Iodo-*m*-xylene **7** with Pd(OAc)$_2$, KO^{t}Bu and dppe, (Table 2, Entry 14)</u>

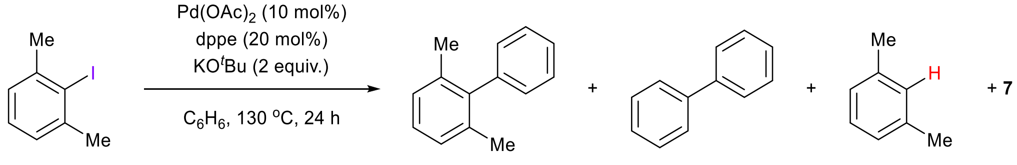

7 8 9 10

The reaction was conducted according to General Procedure A with $Pd(OAc)_2$ (16 mg, 0.07 mmol, 10 mol%), dppe (56 mg, 0.14 mmol, 20 mol%), KO^{t}Bu (157 mg, 1.4 mmol, 2 equiv.) and benzene.

GCFID data are below including tables quantitating components that had been calibrated (for calibrations see pages S73 - S77**).**

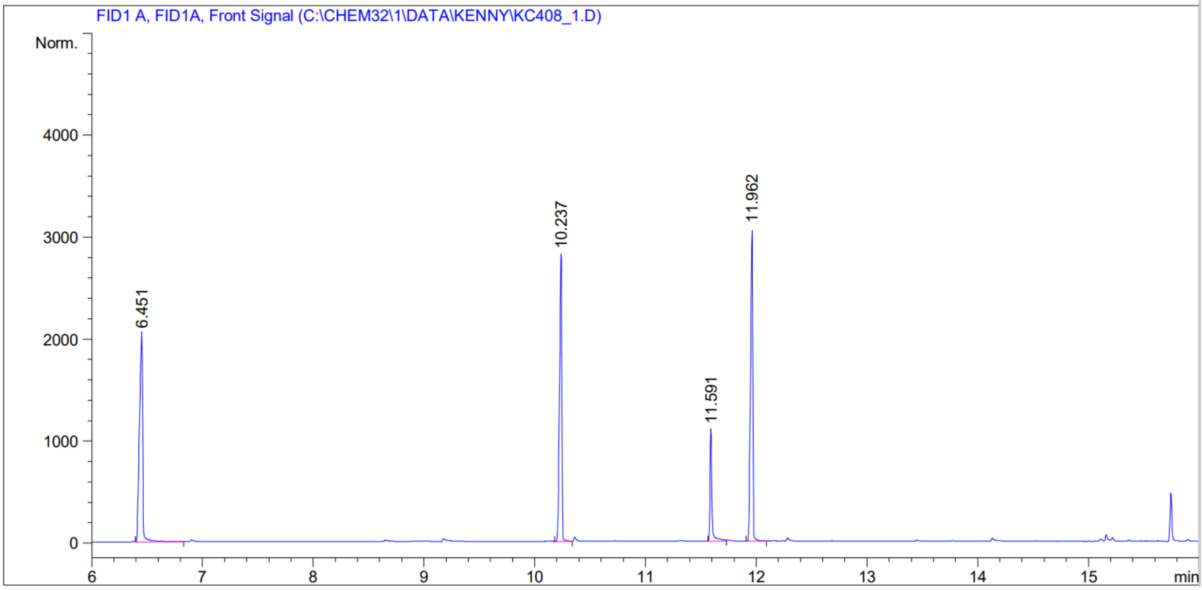

Retention Time	Sample	Peak Area	%Yield
6.451	*m*-Xylene **10**	4266.01953	22.4%
10.237	Dodecane	4146.35156	N/A
11.591	Biphenyl **9**	1342.30005	5.0%
11.962	2,6-Dimethylbiphenyl **8**	4490.98096	13.8%

*This reaction was carried out in duplicate with the average below

Sample	%Yield
m-Xylene **10**	22.2%
2-Iodo-*m*-xylene **7**	0
Biphenyl **9**	5.1%
2,6-Dimethylbiphenyl **8**	12.9%

Outcome - The Reaction of 2-Iodo-*m*-xylene **7** with $Pd(OAc)_2$, KO^{t}Bu and dppe led to the formation of the 3 principal compounds **8**, **9** and **10**. The ratio of **8**:**9** [2.53:1] indicated that an organometallic mechanism contributed majorly to C-C bond formation

<u>Reaction of 2-Iodo-*m*-xylene **7** with $Pd(OAc)_2$, KO^{t}Bu and DPEphos (Table 2, Entry 15)</u>

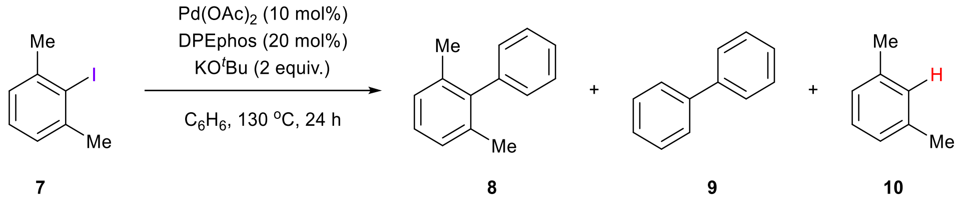

The reaction was conducted according to General Procedure A with $Pd(OAc)_2$ (16 mg, 0.07 mmol, 10 mol%), DPEphos (75 mg, 0.14 mmol, 20 mol%), KO^{t}Bu (157 mg, 1.4 mmol, 2 equiv.) and benzene.

GCFID data are below, including tables quantitating components that had been calibrated (for calibrations see pages S73 - S77).

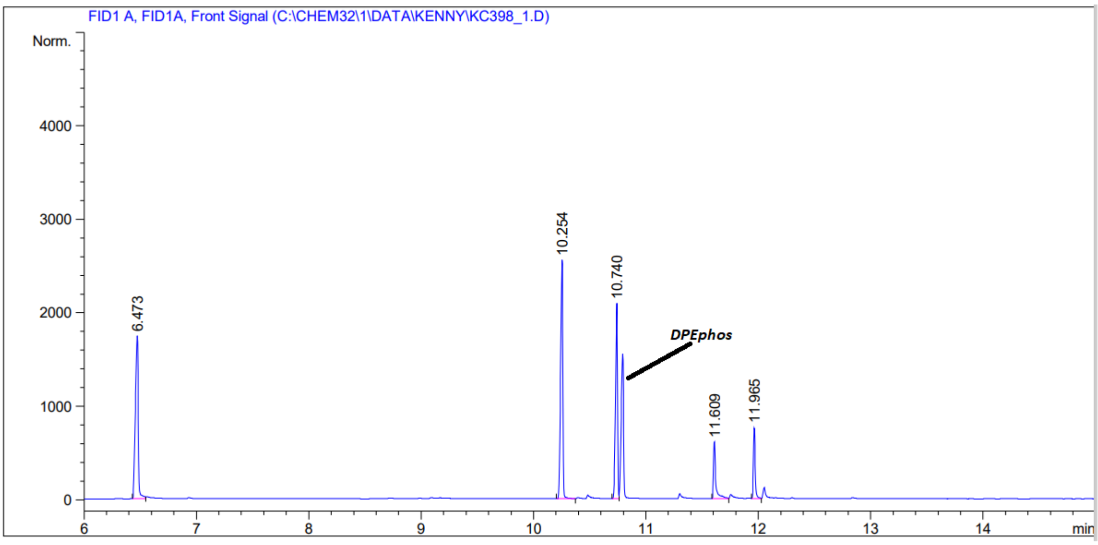

Retention Time	Sample	Peak Area	%Yield
6.473	*m*-Xylene **10**	3087.08472	17.3%
10.254	Dodecane	3647.91284	N/A
10.740	2-Iodo-*m*-xylene **7**	2566.89282	15.1%
11.609	Biphenyl **9**	833.97089	3.3%
11.965	2,6-Dimethylbiphenyl **8**	840.88586	2.8%

*This reaction was carried out in duplicate with the average below

Sample	%Yield
m-Xylene	17.7% **10**
2-Iodo-*m*-xylene	16.9% **7**
Biphenyl	3.8% **9**
2,6-Dimethylbiphenyl	3.3%**8**

Outcome - The reaction of 2-Iodo-*m*-xylene **7** with $Pd(OAc)_2$, KO^{t}Bu and DPEphos led to the formation of the 3 principal compounds **8**, **9** and **10**. The ratio of **8**:**9** [1:1.17] indicated that both an organometallic mechanism and a BHAS mechanism contributed to C-C bond formation.

Reaction of 2-Iodo-*m*-xylene **7** with $Pd(OAc)_2$, KO^{t}Bu and Xantphos (Table 2, Entry 16)

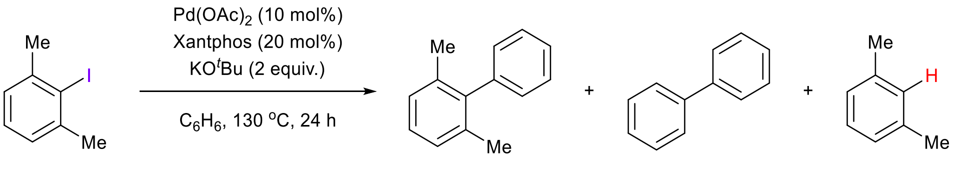

The reaction was conducted according to General Procedure A with $Pd(OAc)_2$ (16 mg, 0.07 mmol, 10 mol%), Xantphos (75 mg, 0.14 mmol, 20 mol%), KO^{t}Bu (157 mg, 1.4 mmol, 2 equiv.) and benzene.

GCFID data including table quantitating components that had been calibrated (for calibrations see pages S73 - S77).

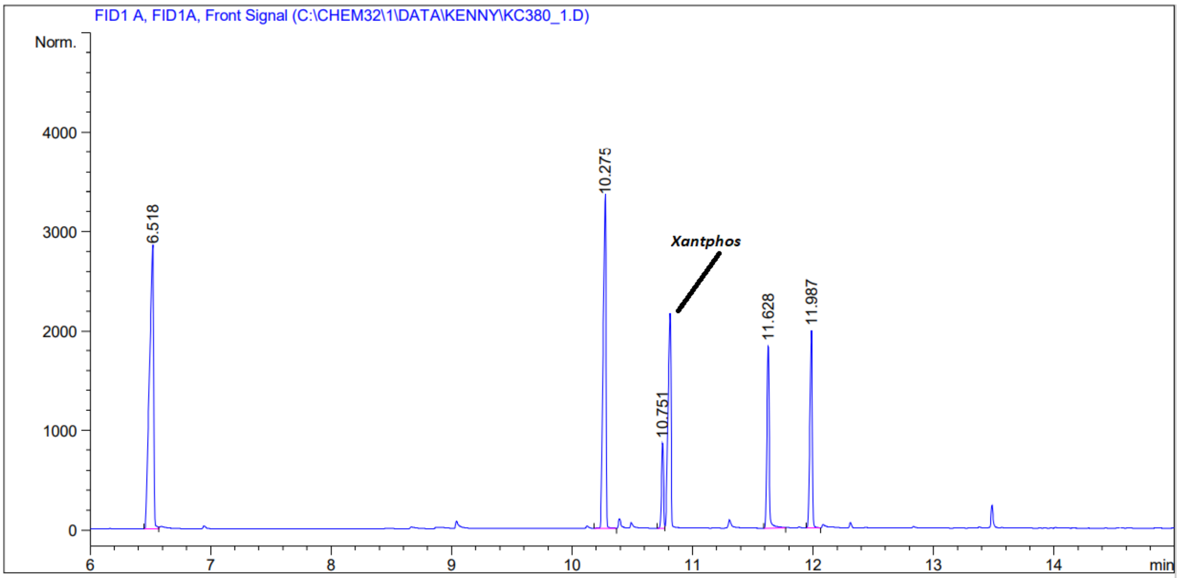

Retention Time	Sample	Peak Area	%Yield
6.518	*m*-Xylene **10**	6931.34082	27.4%
10.275	Dodecane	5506.75977	N/A
10.751	2-Iodo-*m*-xylene **7**	971.05304	15.3%
11.628	Biphenyl **9**	2458.06519	6.8%
11.987	2,6-Dimethylbiphenyl **8**	2748.07275	6.4%

*This reaction was carried out in duplicate with the average below

Sample	%Yield
m-Xylene **10**	25.8%
2-Iodo-*m*-xylene **7**	9.3%
Biphenyl **9**	6.6%
2,6-Dimethylbiphenyl **8**	6.0%

Outcome - The reaction of 2-Iodo-*m*-xylene **7** with $Pd(OAc)_2$, KO^{t}Bu and Xantphos led to the formation of the 3 principal compounds **8**, **9**and **10**. The ratio of **8**:**9** (1:1.1) indicated that both an organometallic mechanism and a BHAS mechanism contributed to C-C bond formation.

Reaction of 2-Iodo-*m*-xylene **7** with $Pd(OAc)_2$, KO^{t}Bu and IMes.HCl (Table 2, Entry 17)

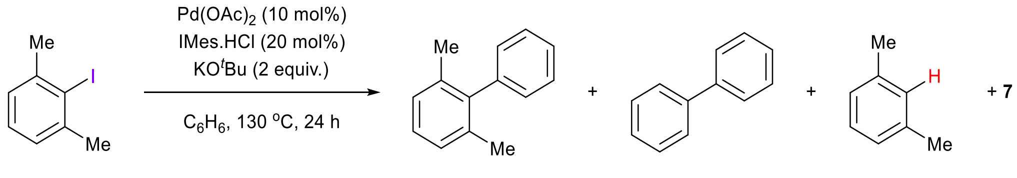

The reaction was conducted according to General Procedure A with $Pd(OAc)_2$ (16 mg, 0.07 mmol, 10 mol%), IMes.HCl (48 mg, 0.14 mmol, 20 mol%), KO^{t}Bu (157 mg, 1.4 mmol, 2 equiv.) and benzene (5 mL).

GCFID data including table quantitating components that had been quantitatively calibrated (for calibrations see pages S73 - S77).

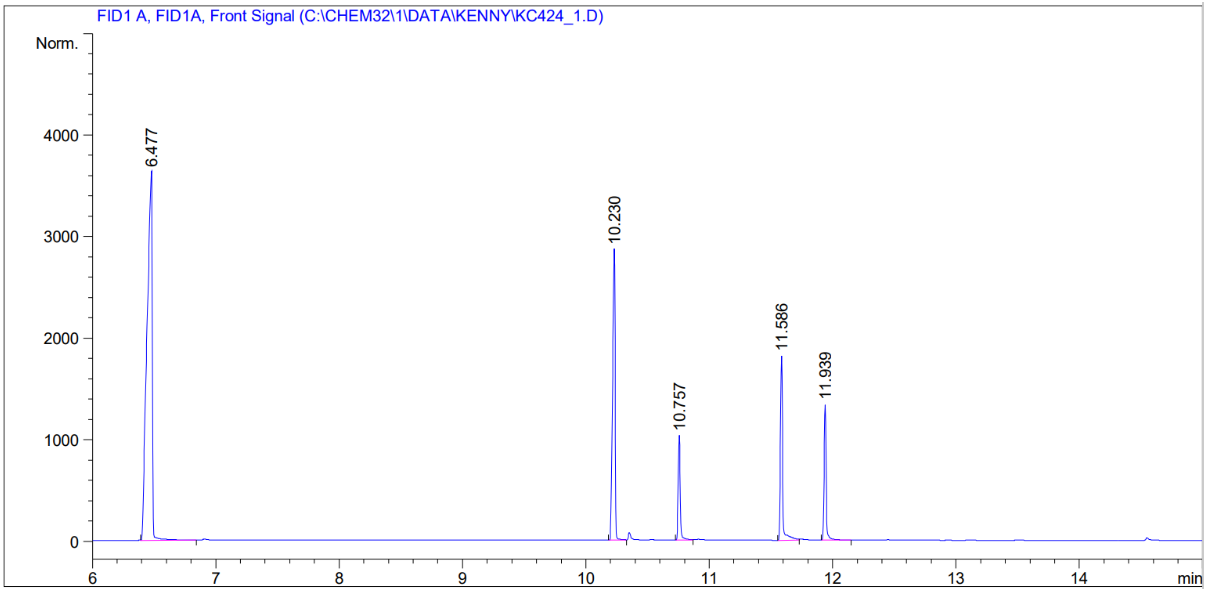

Retention Time	Sample	Peak Area	%Yield
6.477	*m*-Xylene	3628.18848	52.4%
10.230	Dodecane	2870.87354	N/A
10.757	2-Iodo-*m*-xylene	1032.96460	6.3%
11.586	Biphenyl	1816.93005	7.7%
11.939	2,6-Dimethylbiphenyl	1333.48767	4.4%

*This reaction was carried out in duplicate with the average below

Sample	%Yield
m-Xylene	54.3%
2-Iodo-*m*-xylene	3.2%
Biphenyl	7.8%
2,6-Dimethylbiphenyl	4.9%

Outcome - The reaction of 2-Iodo-*m*-xylene **7** with $Pd(OAc)_2$, KO^{t}Bu and IMes.HCl led to the formation of the 3 principal compounds **8**, **9** and **10**. The ratio of **8**:**9** was ~1.0:1.3 and is not consistent with radical chemistry.

Reaction of 2-Iodo-*m*-xylene **7** with $Pd(OAc)_2$, KO^{t}Bu and XPhos (Table 2, Entry 18)

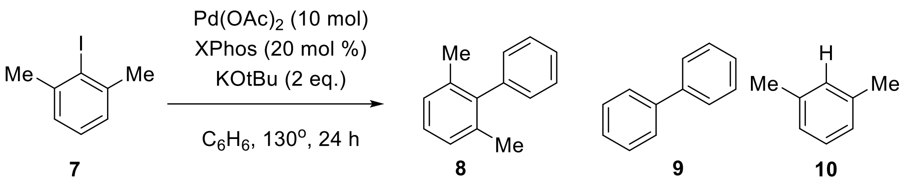

The reaction was conducted according to General Procedure A with $Pd(OAc)_2$ (16 mg, 0.07 mmol, 10 mol%), XPhos (67 mg, 0.14 mmol, 20 mol%), KO^{t}Bu (157 mg, 1.4 mmol, 2 equiv.) and benzene (5 mL).

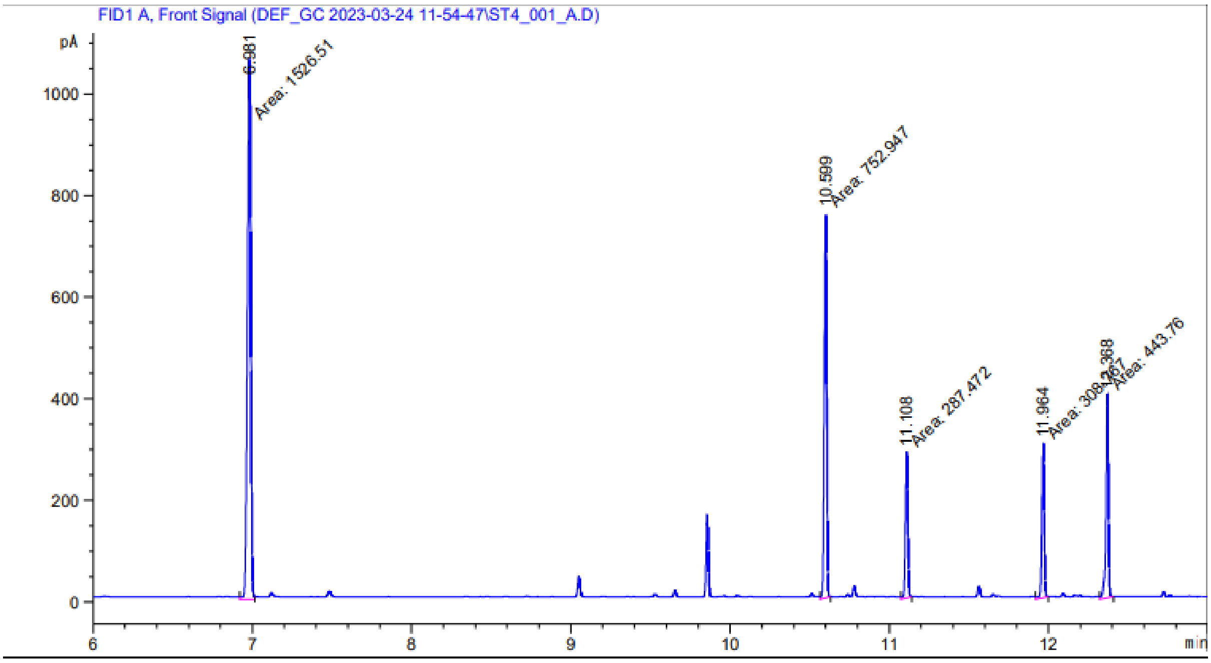

Retention time	Sample	Peak area	% Yield
6.961	*m*-xylene	1526.51	46.1
10.599	*n*-dodecane	752.95	N/A
11.108	iodoxylene	287.47	9.0
11.964	Biphenyl	308.77	7.0
12.378	2,6-Dimethylbiphenyl	443.76	9.1

This reaction was carried out in duplicate with the average below

Sample	%Yield
m-Xylene **10**	45.5%
2-Iodo-*m*-xylene **7**	8.9%
Biphenyl **9**	7.3%
2,6-Dimethylbiphenyl **8**	8.8%

For data for Table 3, entry 1, see Table 2, entry 3

Reaction of 2-Iodo-*m*-xylene **7** with $Pd(PPh_3)_4$, KO^{t}Bu and dppf (Table 3, Entry 2)

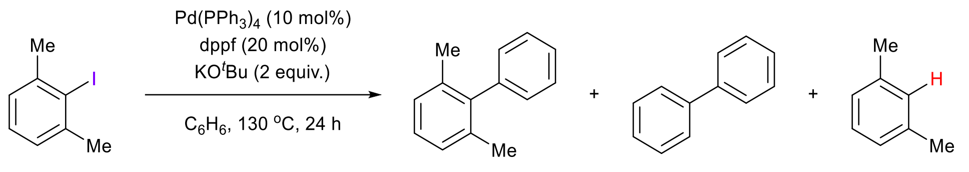

The reaction was conducted according to General Procedure A with $Pd(PPh_3)_4$ (81 mg, 0.07 mmol, 10 mol%), dppf (79 mg, 0.14 mmol, 20 mol%), KO^tBu (157 mg, 1.4 mmol, 2 equiv.) and benzene.

GCMS Chromatogram

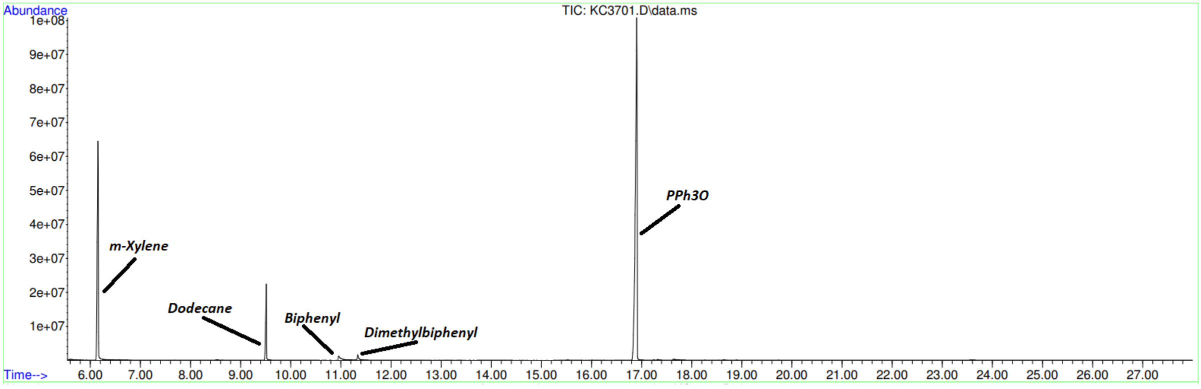

GCFID data including table quantitating components that had been calibrated (for calibrations see pages S73 - S77).

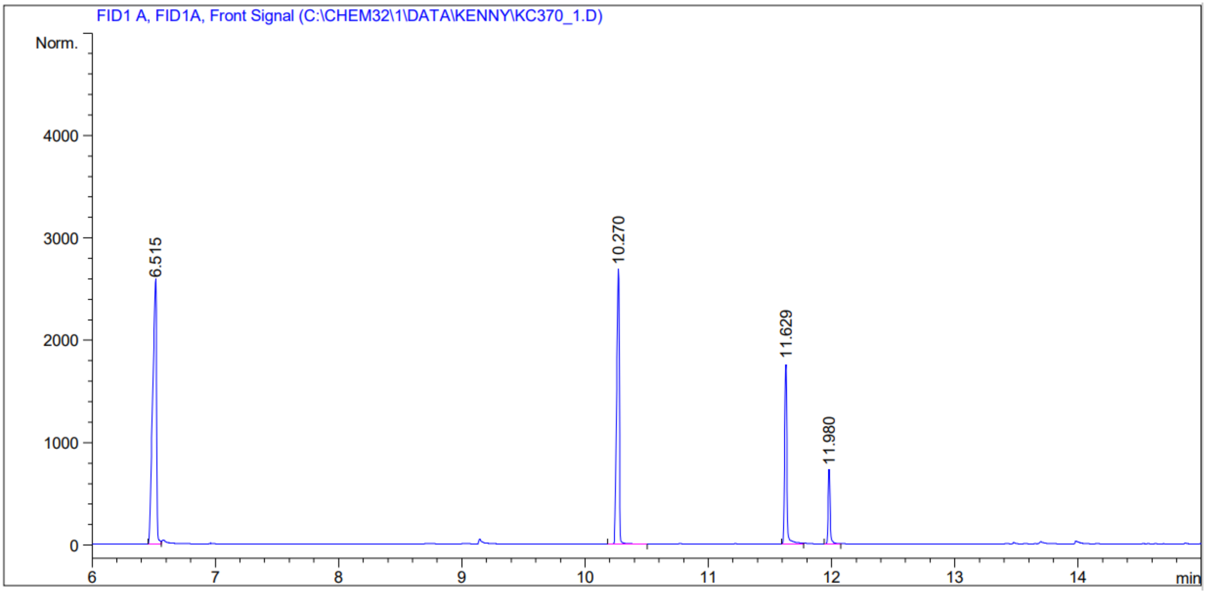

Retention Time	Sample	Peak Area	%Yield
6.515	*m*-Xylene **10**	5723.91699	32.1%
10.270	Dodecane	3970.38550	N/A
11.629	Biphenyl **9**	2313.45850	9.1%
11.980	2,6-Dimethylbiphenyl **8**	820.36578	2.7%

*This reaction was carried out in duplicate with the average below

Sample	%Yield
m-Xylene **10**	31.2%
2-Iodo-*m*-xylene **7**	0
Biphenyl **9**	8.6%
2,6-Dimethylbiphenyl **8**	2.6%

Outcome - The Reaction of 2-Iodo-*m*-xylene **7** with $Pd(PPh_3)_4$, KO^tBu and dppf led to the formation of the 3 principal compounds **8**, **9** and **10**. The ratio of **8**:**9** indicated that the BHAS mechanism was the major contributor to C-C bond formation.

Reaction of 2-Iodo-*m*-xylene **7** with $Pd(PPh_3)_2Cl_2$, KO^tBu and dppf (Table 3, entry 3)

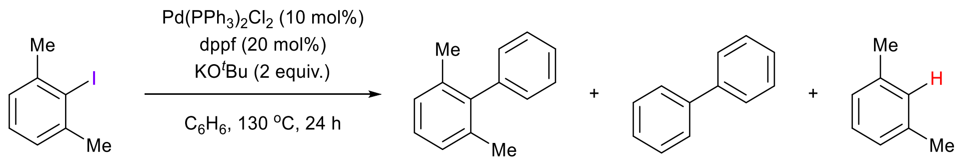

7 **8** **9** **10**

The reaction was conducted according to General Procedure A with $Pd(PPh_3)_2Cl_2$ (49 mg, 0.07 mmol, 10 mol%), dppf (79 mg, 0.14 mmol, 20 mol%), KO^tBu (157 mg, 1.4 mmol, 2 equiv.) and benzene.

GCMS Chromatogram

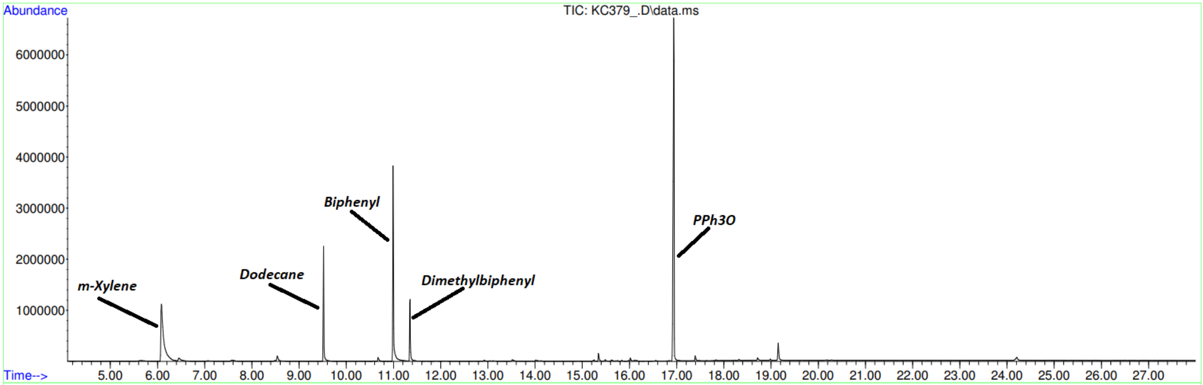

GCFID data below include tables quantitating components that had been calibrated (for calibrations see pages S73 - S77).

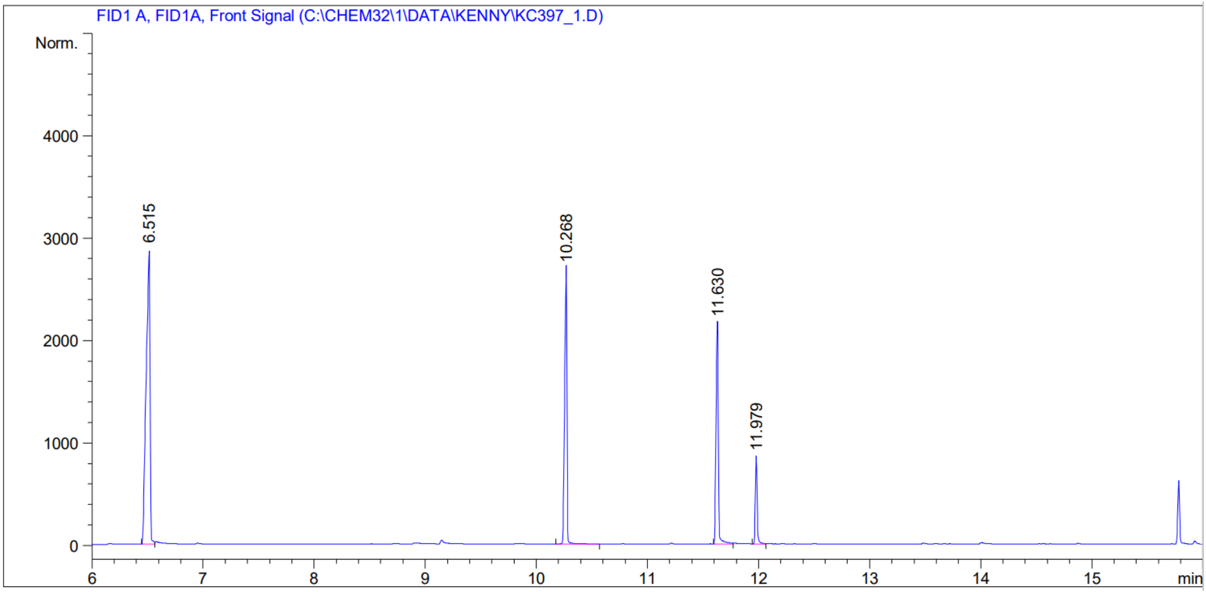

Retention Time	Sample	Peak Area	%Yield
6.515	*m*-Xylene **10**	6944.19189	36.3%
10.268	Dodecane	3937.00317	N/A
11.630	Biphenyl **9**	3193.21777	11.7%
11.979	2,6-Dimethylbiphenyl **8**	1003.66528	3.1%

*This reaction was carried out in duplicate with the average below

Sample	%Yield
m-Xylene **10**	36.0%
2-Iodo-*m*-xylene **7**	0
Biphenyl **9**	11.6%
2,6-Dimethylbiphenyl **8**	3.2%

Outcome - The Reaction of 2-Iodo-*m*-xylene **7** with $Pd(PPh_3)Cl_2$, KO^tBu and dppf led to the formation of the 3 principal compounds **8**, **9** and **10**. The ratio of **8**:**9** [1:3.30] indicated that a BHAS mechanism was the major contributor to C-C bond formation .

Reaction of 2-Iodo-*m*-xylene **7** with $PdCl_2$, KO^tBu and dppf (Table 3, Entry 4)

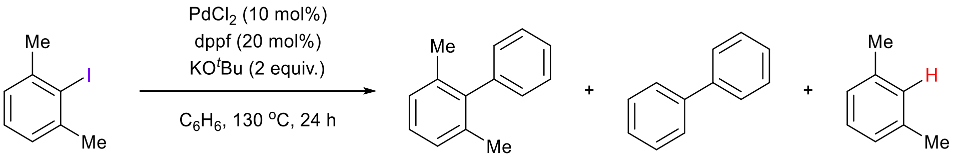

7 **8** **9** **10**

The reaction was conducted according to General Procedure A with $PdCl_2$ (12 mg, 0.07 mmol, 10 mol%), dppf (79 mg, 0.14 mmol, 20 mol%), KO^tBu (157 mg, 1.4 mmol, 2 equiv.) and benzene.

GCFID data below include tables quantitating components that had been calibrated (for calibrations see pages S73 - S77).

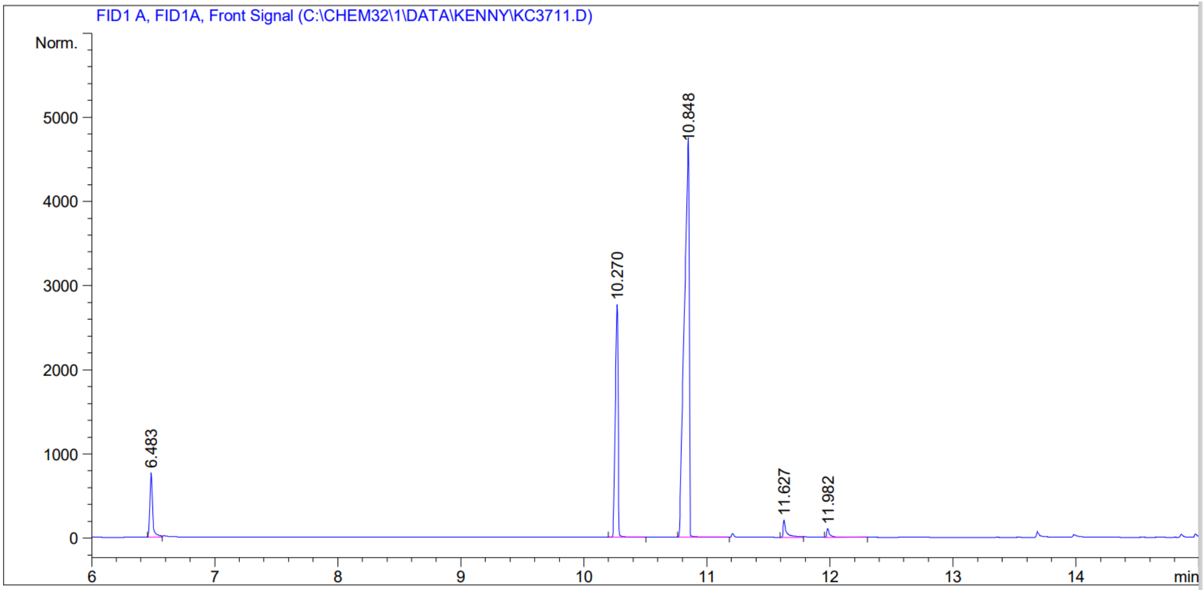

Retention Time	Sample	Peak Area	%Yield
6.483	*m*-Xylene **10**	1204.25879	5.7%
10.270	Dodecane	4286.10010	N/A
10.848	2-Iodo-*m*-xylene **7**	13103.9	66.5%
11.627	Biphenyl **9**	382.57574	1.1%
11.982	2,6-Dimethylbiphenyl **8**	173.43887	0.4%

*This reaction was carried out in duplicate with the average below

Sample	%Yield
m-Xylene **10**	5.9%
2-Iodo-*m*-xylene **7**	72.6%
Biphenyl **9**	1.4%
2,6-Dimethylbiphenyl **8**	0.6%

Outcome - The reaction of 2-Iodo-*m*-xylene **7** with $PdCl_2$, KO^{t}Bu and dppf led to the formation of the 3 principal compounds **8**, **9** and **10**. The ratio of **8**:**9** [1:3.63] indicated that both an organometallic mechanism and a BHAS mechanism contributed to C-C bond formation although the yields of products were very low.

<u>Reaction of 2-Iodo-*m*-xylene **7** with $Pd(TFA)_2$, KO^{t}Bu and dppf (Table 3, Entry 5)</u>

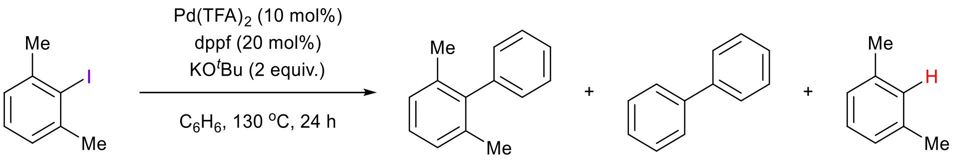

The reaction was conducted according to General Procedure A with $Pd(TFA)_2$ (23 mg, 0.07 mmol, 10 mol%), dppf (79 mg, 0.14 mmol, 20 mol%), KO^tBu (157 mg, 1.4 mmol, 2 equiv.) and benzene.

GCMS Chromatogram

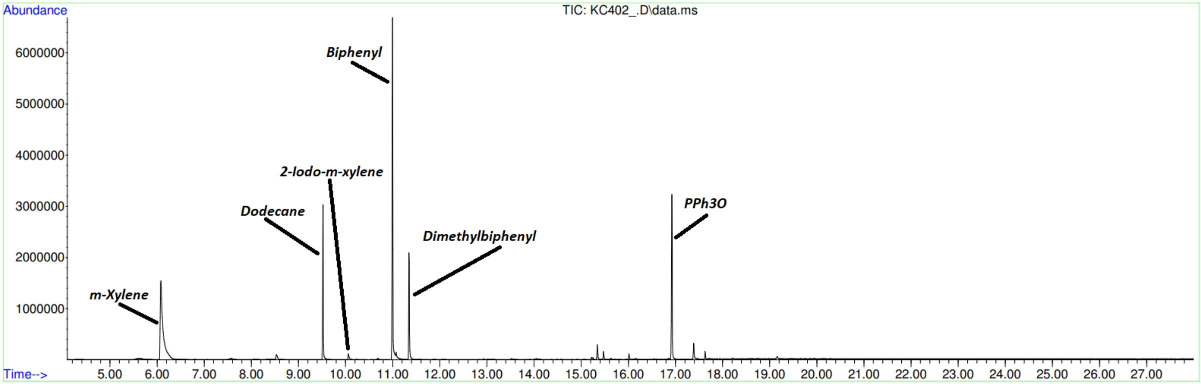

GCFID data below include tables quantitating components that had been calibrated (for calibrations see pages S73 - S77).

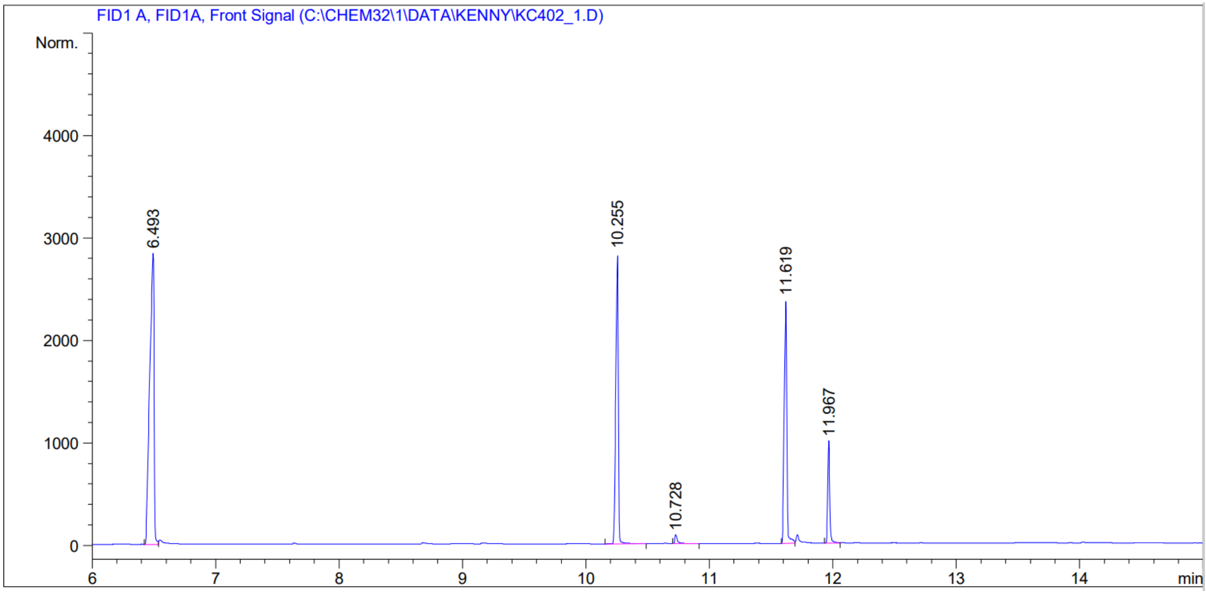

Retention Time	Sample	Peak Area	%Yield
6.493	*m*-Xylene **10**	6963.59766	36.3%
10.255	Dodecane	4114.54785	N/A
10.728	2-Iodo-*m*-xylene **7**	127.59853	0.7%
11.619	Biphenyl **9**	3505.10010	12.8%
11.967	2,6-Dimethylbiphenyl **8**	1159.83948	3.6%

*This reaction was carried out in duplicate with the average below

Sample	%Yield
m-Xylene **10**	36.0%
2-Iodo-*m*-xylene **7**	0.7%
Biphenyl **9**	13.0%
2,6-Dimethylbiphenyl **8**	3.5%

Outcome - The reaction of 2-iodo-*m*-xylene **7** with $Pd(TFA)_2$, KO^{t}Bu and dppf led to the formation of the 3 principal compounds **8**, **9** and **10**. The ratio of **8**:**9** [1:3.71] indicated that a BHAS mechanism was the main contributor to C-C bond formation.

Reaction of 2-Iodo-*m*-xylene **7** with $Pd(dppf)Cl_2$, KO^{t}Bu and dppf (Table 3, Entry 6)

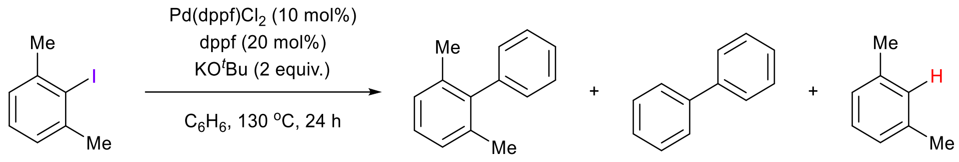

7 **8** **9** **10**

The reaction was conducted according to General Procedure A with $Pd(dppf)Cl_2$ (51 mg, 0.07 mmol, 10 mol%), dppf (79 mg, 0.14 mmol, 20 mol%), KO^{t}Bu (157 mg, 1.4 mmol, 2 equiv.) and benzene.

GCFID data below include tables quantitating components that had been calibrated (for calibrations see pages S73 - S77).

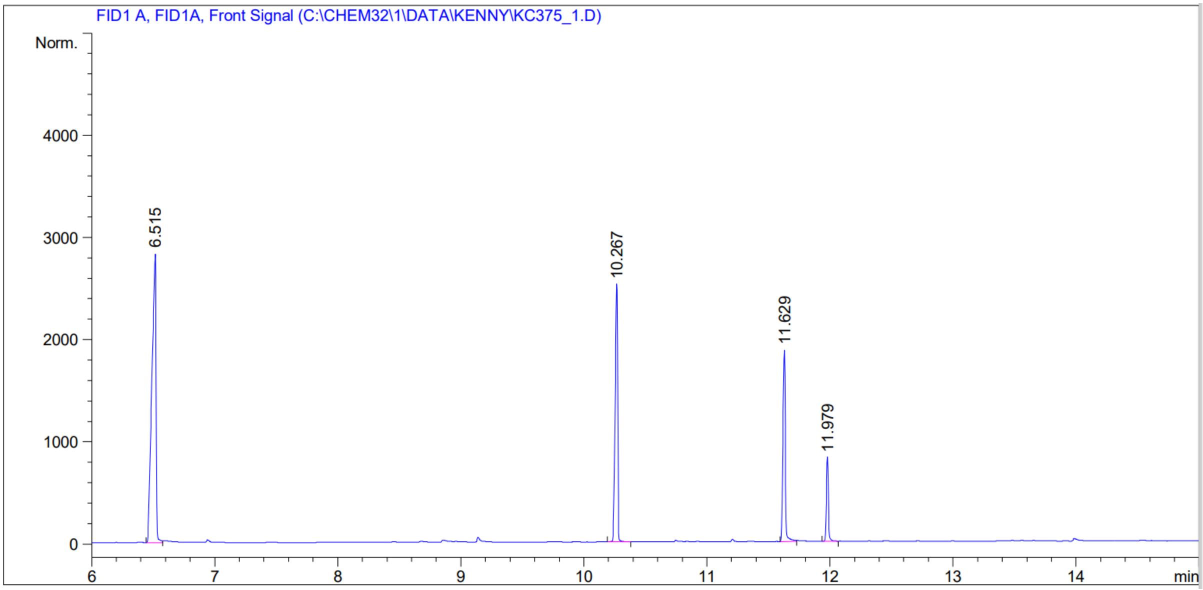

Retention Time	Sample	Peak Area	%Yield
6.515	*m*-Xylene **10**	6794.03418	42.3%
10.267	Dodecane	3555.08350	N/A
11.629	Biphenyl **9**	2549.48804	10.7%
11.979	2,6-Dimethylbiphenyl **8**	930.52515	3.2%

*This reaction was carried out in duplicate with the average below

Sample	%Yield
m-Xylene **10**	41.3%
2-Iodo-*m*-xylene **7**	0
Biphenyl **9**	11.0%
2,6-Dimethylbiphenyl **8**	3.2%

Outcome - The Reaction of 2-Iodo-*m*-xylene **7** with Pd(dppf)Cl_2, KO^{t}Bu and dppf led to the formation of the 3 principal compounds **8**, **9** and **10**. The ratio of **8**:**9** [1:3.44] indicated that a BHAS mechanism was the main contributor to C-C bond formation.

Reaction of 2-Iodo-*m*-xylene **7** with Pd[P(*o*-tol)$_3$]$_2$, KO^{t}Bu and dppf (Table 3, Entry 7)

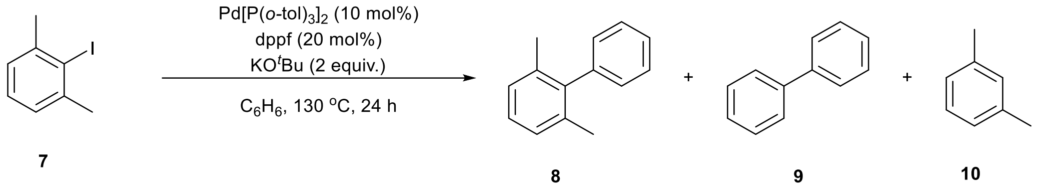

The reaction was conducted according to General Procedure A with Pd[P(*o*-tol)$_3$]$_2$ (25 mg, 0.035 mmol, 10 mol%), dppf (39 mg, 0.07 mmol, 20 mol%), KO^{t}Bu (78 mg, 0.7 mmol, 2 equiv.) and benzene (2.5 mL).

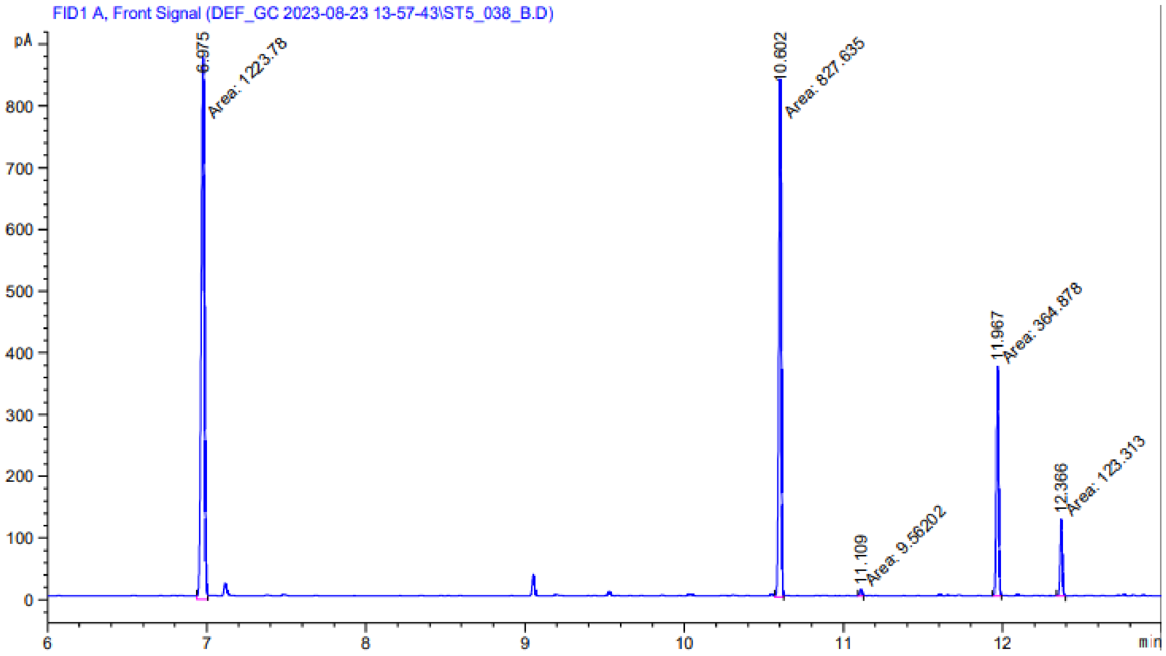

Retention Time	Sample	Peak Area	%Yield
6.975	*m*-Xylene **10**	1223.78	41.4
10.602	Dodecane	827.64	N/A
11.109	Iodoxylene **7**	9.56	0.3
11.967	Biphenyl **9**	364.88	9.3
12.366	2,6-Dimethylbiphenyl **8**	123.31	2.8

Reaction of 2-Iodo-*m*-xylene **7** with dppf (Table 4, Entry 2)

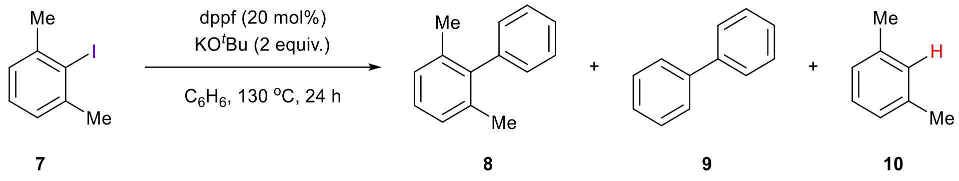

The reaction was conducted according to General Procedure A with dppf (79 mg, 0.14 mmol, 20 mol%), KO^{t}Bu (157 mg, 1.4 mmol, 2 equiv.) and benzene.

GCFID data below includes tables quantitating components that had been calibrated (for calibrations see pages S73 - S77).

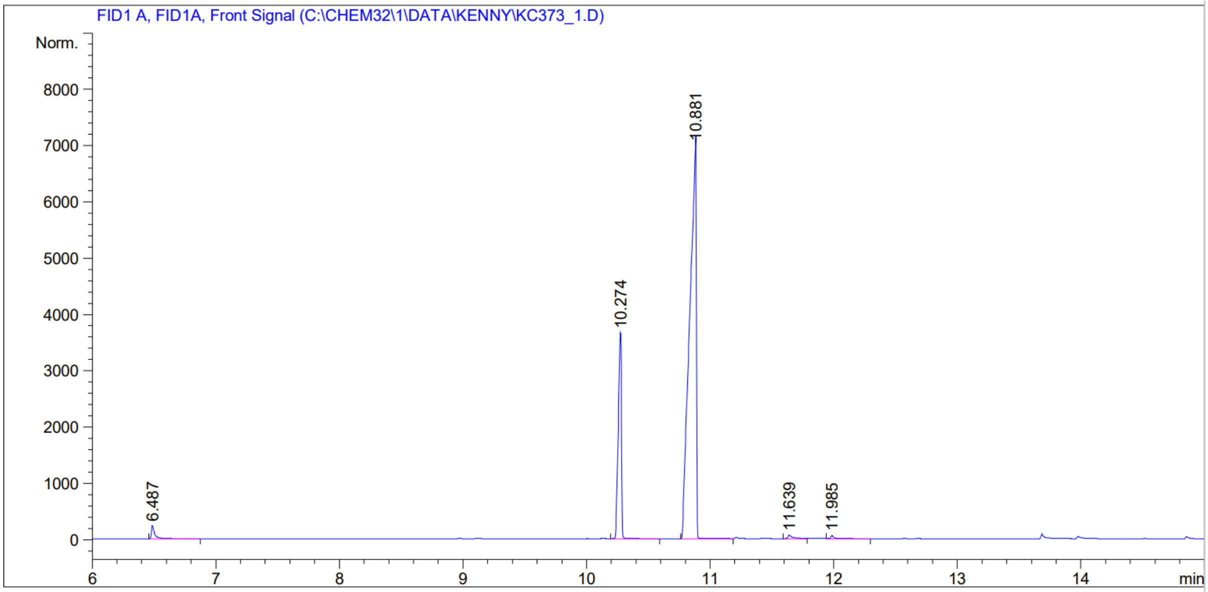

Retention Time	Sample	Peak Area	%Yield
6.487	*m*-Xylene **10**	529.48975	1.8%
10.274	Dodecane	6550.46924	N/A
10.881	2-Iodo-*m*-xylene **7**	26700.6	92.6%
11.639	Biphenyl **9**	193.68407	0.5%
11.985	2,6-Dimethylbiphenyl **8**	102.04285	0.2%

*This reaction was carried out in duplicate with the average below

Sample	%Yield
m-Xylene **10**	1.8%
2-Iodo-*m*-xylene **7**	93.0%
Biphenyl **9**	0.6%
2,6-Dimethylbiphenyl **8**	0.2%

Outcome - The reaction of 2-iodo-*m*-xylene **7** with dppf and no Pd source provided results similar to that in the absence of dppf. This suggests that the dppf on its own has no impact on the reaction.

Reaction of 2-Iodo-*m*-xylene **7** with $Pd(OAc)_2$, KO^tBu and ferrocene (Table 4, Entry 3)

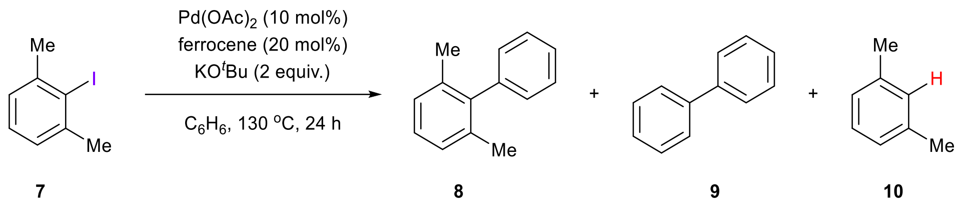

The reaction was conducted according to General Procedure A with $Pd(OAc)_2$ (16 mg, 0.07 mmol, 10 mol%), ferrocene (26 mg, 0.14 mmol, 20 mol%), KO^tBu (157 mg, 1.4 mmol, 2 equiv.) and benzene (5 mL).

GCMS Chromatogram

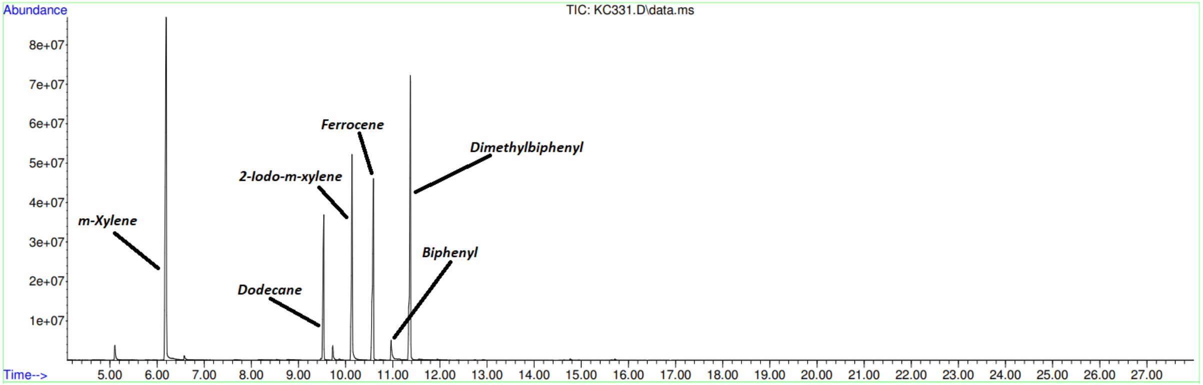

GCFID data below include tables quantitating components that had been calibrated (for calibrations see pages S73 - S77).

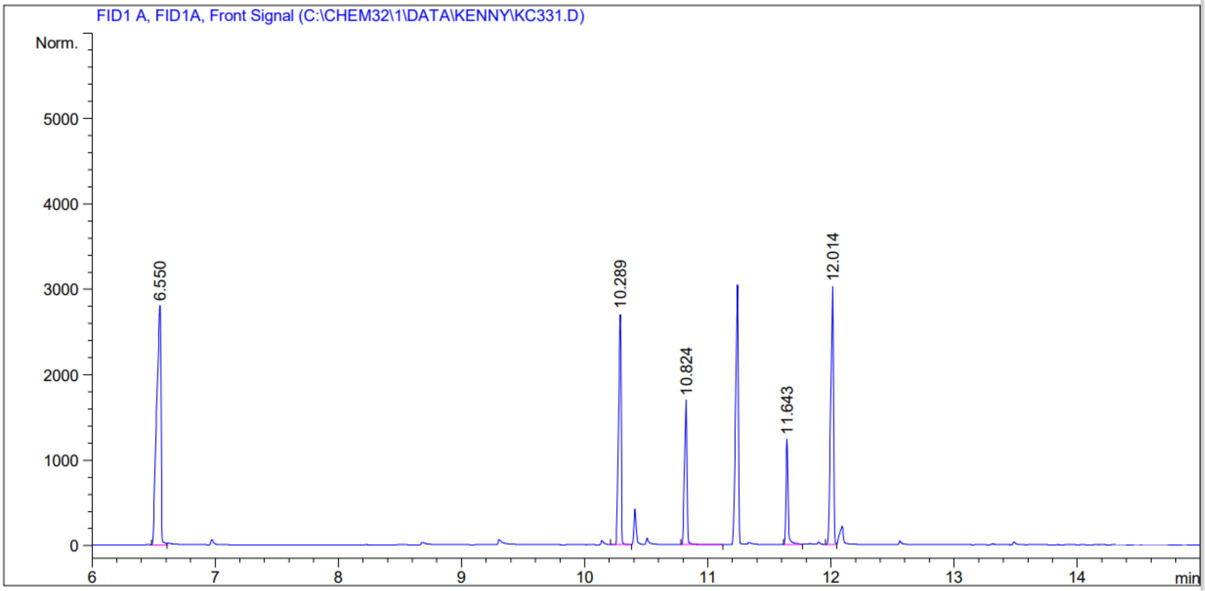

Retention Time	Sample	Peak Area	%Yield
6.550	*m*-Xylene **10**	6771.57471	31.3%
10.289	Dodecane	3930.94873	N/A

10.824	2-Iodo-*m*-xylene **7**	2416.43115	12.3%
11.643	Biphenyl **9**	1494.34814	5.1%
12.014	2,6-Dimethylbiphenyl **8**	4866.01855	13.9%

*This reaction was carried out in duplicate with the average below

Sample	%Yield
m-Xylene **10**	34.1%
2-Iodo-*m*-xylene **7**	6.2%
Biphenyl **9**	5.3%
2,6-Dimethylbiphenyl **8**	15.0%

Outcome - The reaction of 2-iodo-*m*-xylene **7** with $Pd(OAc)_2$, KO^tBu and ferrocene provided results similar to those in the absence of ferrocene (see Table 1, entry 2). This ferrocene has no noticeable impact on the reaction.

Reaction of Benzene **3** with $Pd(OAc)_2$ and KO^tBu (Table 4, Entry 4)

To an oven-dried microwave vial, primed with a stirrer bar, in a glovebox was added $Pd(OAc)_2$ (16 mg, 0.07 mmol, 10 mol%), KO^tBu (157 mg, 1.4 mmol, 2 equiv.) and benzene (7 mL), with the vial subsequently sealed and stirred at 130 °C for 24 h. Once complete, the crude mixture was allowed to cool to room temperature and dodecane (23 µL) was added and mixed. A 100 µL aliquot of the crude mixture was then analysed by both GCMS and GCFID.

GCFID data below including tables quantitating components that had been calibrated (for calibrations see pages S73 - S77).

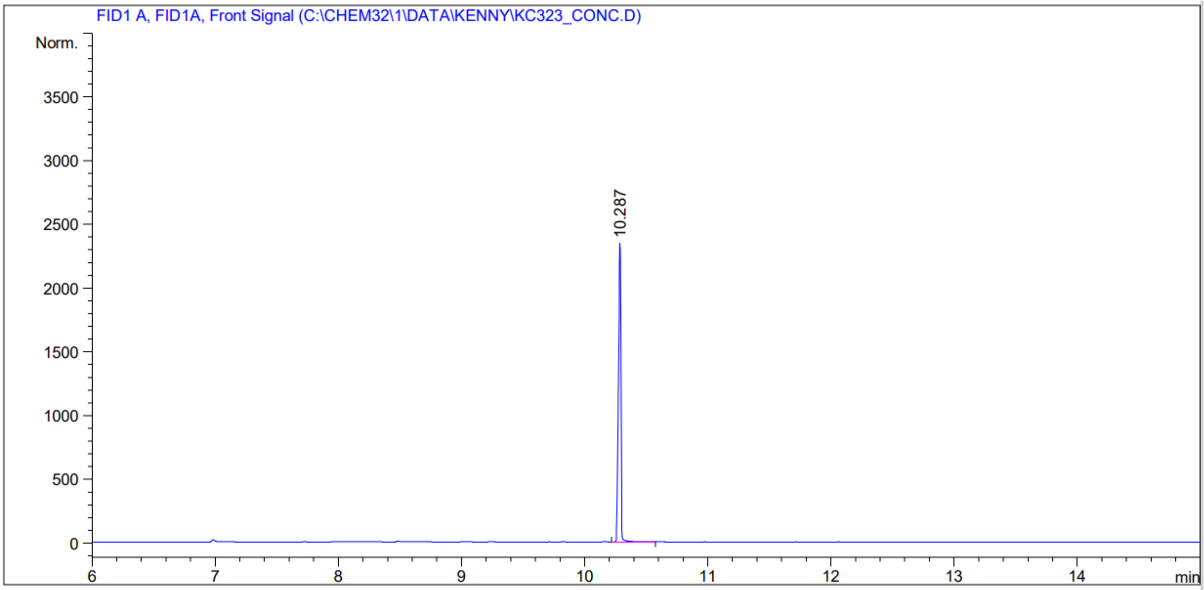

Outcome - The Reaction of Benzene **3** with $Pd(OAc)_2$ and KO^tBu led no evidence of any products.

Reaction of Benzene **3** with $Pd(OAc)_2$, dppf and KO^{t}Bu (Table 4, Entry 5, see Table 2, Entry 3)

Reaction of Benzene **3** with $Pd(OAc)_2$, dppf and NaO^{t}Bu (Table 4, Entry 6)

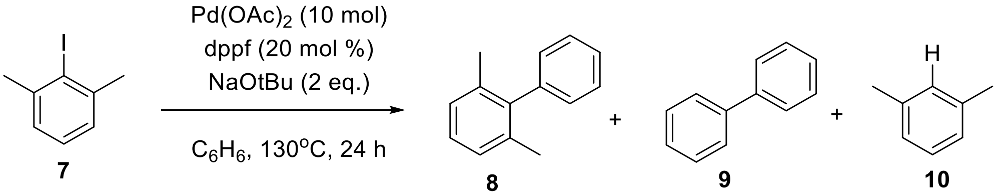

The reaction was conducted according to General Procedure A with $Pd(OAc)_2$ (16 mg, 0.07 mmol, 1 equiv.), dppf (78 mg, 0.14 mmol, 0.2 equiv.), NaOtBu (135 mg, 1.4 mmol, 2 equiv.) and benzene (5 mL).

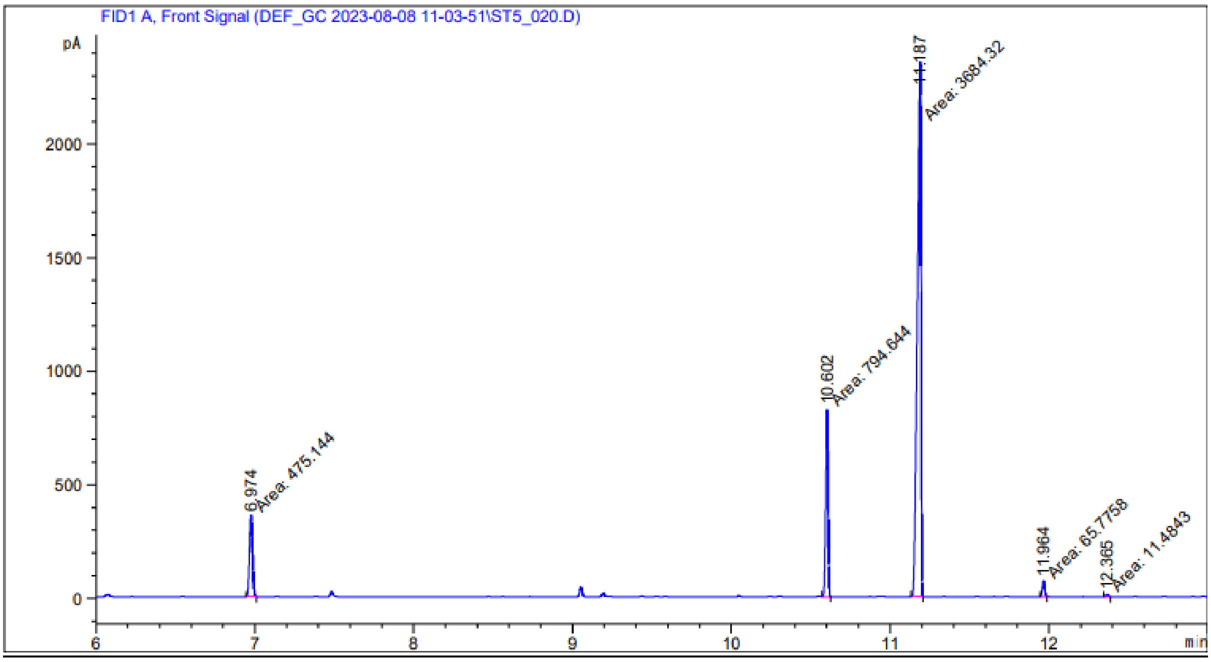

Retention time	Sample	Peak area	% Yield
6.974	*m*-xylene	475.14	9.0
10.602	*n*-dodecane	794.64	N/A
11.187	iodoxylene	3684.32	72.2
11.964	Biphenyl	65.78	0.9
12.365	2,6-Dimethylbiphenyl	11.48	0.1

Reaction of Benzene **3** with $Pd(OAc)_2$ PCy_3 and KO^{t}Bu in C_6H_6 (Table 4, Entry 7, see Table 2, Entry 10)

Reaction of Benzene **3** with $Pd(OAc)_2$, PCy_3 and NaO^tBu (Table 4, Entry 8)

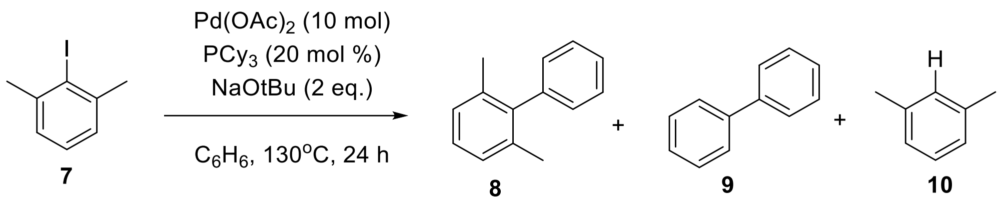

The reaction was conducted according to General Procedure A with $Pd(OAc)_2$ (16 mg, 0.07 mmol, 1 equiv.), PCy_3 (39 mg, 0.14 mmol, 0.2 equiv.), NaOtBu (135 mg, 1.4 mmol, 2 equiv.) and benzene (5 mL).

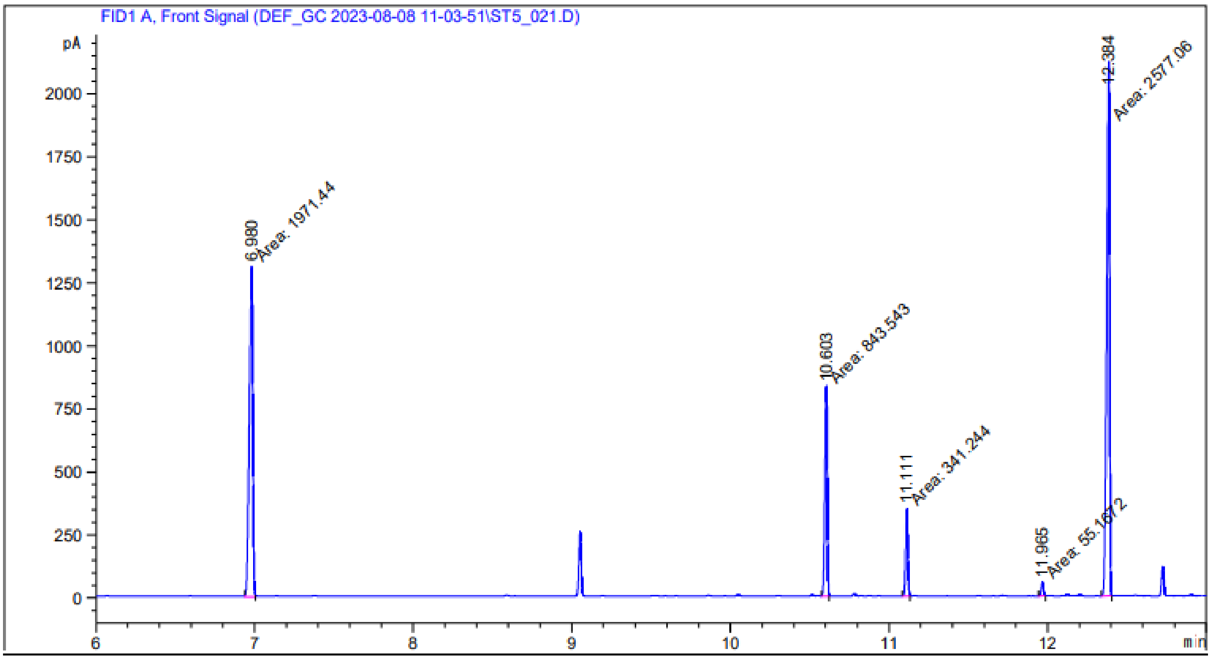

Retention time	Sample	Peak area	% Yield
6.980	*m*-xylene	1971.44	35.9
10.603	*n*-dodecane	843.54	N/A
11.111	iodoxylene	341.24	6.5
11.965	Biphenyl	55.17	0.8
12.384	2,6-Dimethylbiphenyl	2577.06	31.9

Reaction of 2-Iodo-*m*-xylene **7** with $Pd(OAc)_2$, KO^tBu and dppf in C_6D_6 (Table 5, Entry 2)

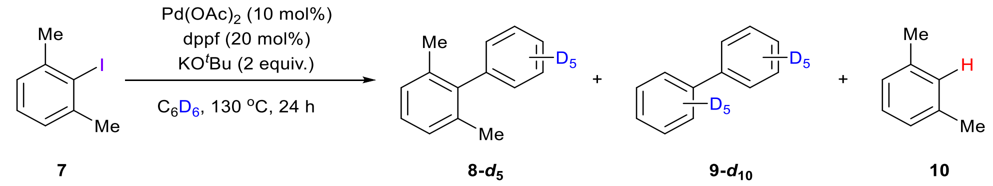

The reaction was conducted according to General Procedure A with $Pd(OAc)_2$ (16 mg, 0.07 mmol, 10 mol%), dppf (79 mg, 0.14 mmol, 20 mol%), KO^tBu (157 mg, 1.4 mmol, 2 equiv.) and benzene-d_6.

GCMS Chromatogram

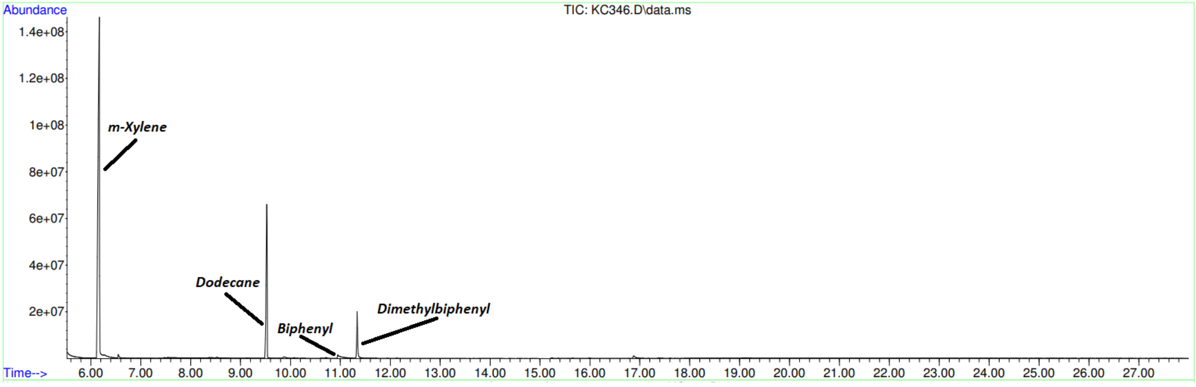

GCFID data below include tables quantitating components that had been calibrated (for calibrations see pages S73 - S77).

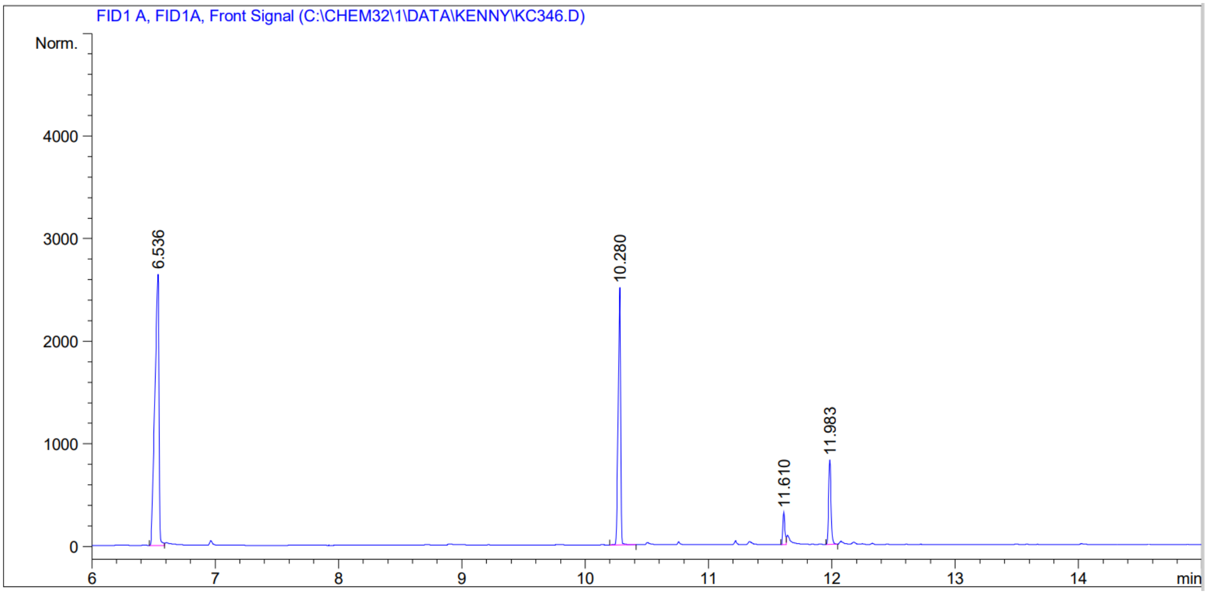

Retention Time	Sample	Peak Area	%Yield
6.536	*m*-Xylene **10**	6232.20068	34.0%
10.280	Dodecane	3463.38135	N/A
11.610	Biphenyl **9**	351.86258	1.4%
11.983	2,6-Dimethylbiphenyl **8**	1112.07434	3.7%

*This reaction was carried out in duplicate with the average below

Sample	%Yield
m-Xylene **10**	33.0%
2-Iodo-*m*-xylene **7**	0
Biphenyl **9**	1.4%
2,6-Dimethylbiphenyl **8**	3.9%

For representative mass spectra of **8-*d*$_5$**, **9-*d*$_{10}$**, and **10-*d*$_1$** see pages S58 et seq.

Outcome - The reaction of 2-iodo-*m*-xylene **7** with $Pd(OAc)_2$, KO^{t}Bu and dppf in C_6D_6 led to the formation of the 3 principal compounds **8-*d*$_5$**, **9-*d*$_{10}$** and **10**. An isotope effect was observed (relative to Table 3, entry 1 which is reproduced as Table 5, entry 1) for the formation of deuterated biphenyl **9-*d*$_{10}$** as expected, while the amount of dimethylbiphenyl-*d*$_5$ **8-*d*$_5$** remained about equal with the parent [undeuterated] conditions. The majority of the *m*-xylene **10** formed in this experiment was not deuterium-labelled. The isotope effect observed for **9-*d*$_{10}$** and lack of isotope effect for **8-*d*$_5$** are consistent with a BHAS mechanism.

Reaction of 2-Iodo-*m*-xylene **7** with $Pd(PPh_3)_4$ and KO^{t}Bu in C_6D_6 (Table 5, Entry 4).

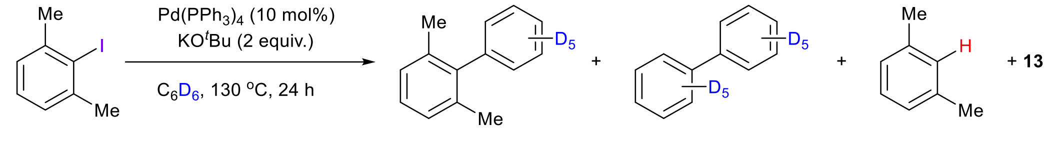

The reaction was conducted according to General Procedure A with $Pd(PPh_3)_4$ (81 mg, 0.07 mmol, 10 mol%), KO^{t}Bu (157 mg, 1.4 mmol, 2 equiv.) and benzene-*d*$_6$.

GCMS Chromatogram

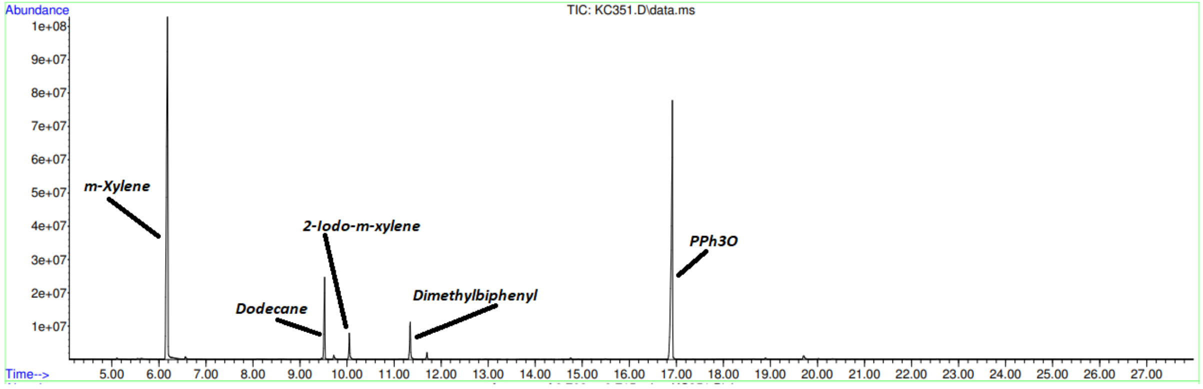

GCFID data below include tables quantitating components that had been calibrated (for calibrations see pages S73 - S77).

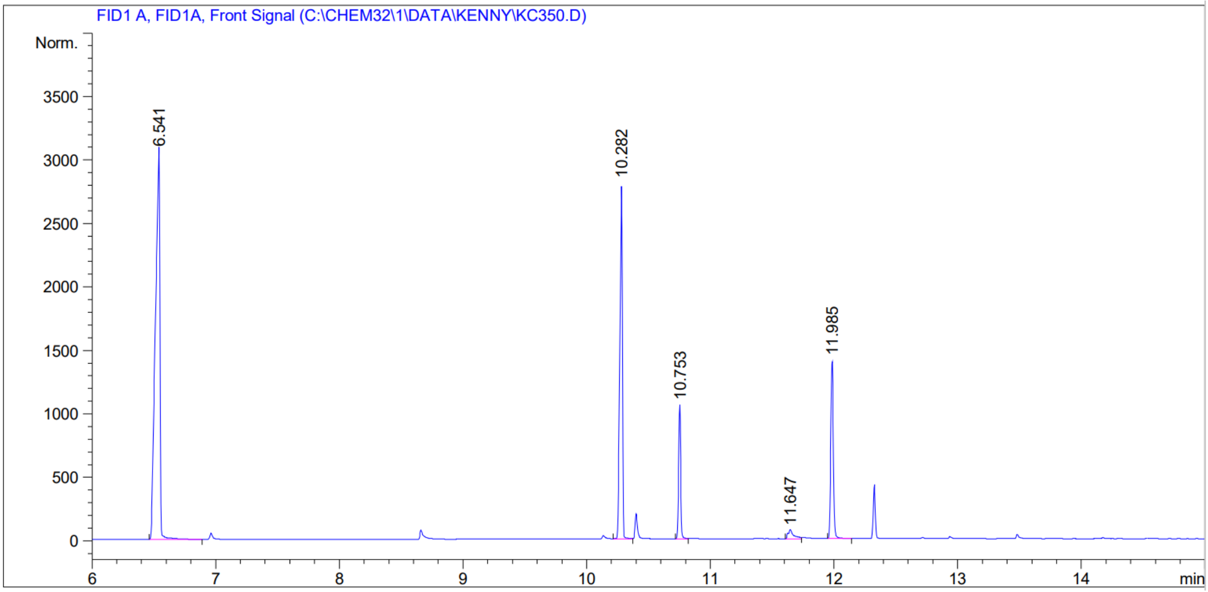

Retention Time	Sample	Peak Area	%Yield
6.541	*m*-Xylene **10**	7937.72803	41.6%
10.282	Dodecane	4041.48413	N/A
10.753	2-Iodo-*m*-xylene **7**	1233.26831	6.8%
11.647	Biphenyl **9**	220.54298	0.3%
11.985	2,6-Dimethylbiphenyl **8**	2029.53699	6.3%

*This reaction was carried out in duplicate with the average below

Sample	%Yield
m-Xylene **10**	45.6%
2-Iodo-*m*-xylene **7**	7.4%
Biphenyl **9**	0.3%
2,6-Dimethylbiphenyl **8**	6.9%

For representative mass spectra of **9-*d*$_{10}$**, **8-*d*$_5$** and **10-*d*$_1$** see pages S58 et seq.

Outcome - The reaction of 2-iodo-*m*-xylene **7** with $Pd(PPh_3)_4$ and KO^{t}Bu in C_6D_6 led to the formation of the 3 principal compounds **8-*d*$_5$**, **9-*d*$_{10}$** and **10**. An isotope effect was observed for the formation of deuterated biphenyl **9-*d*$_{10}$**, while the amount of dimethylbiphenyl-*d*$_5$ **8-*d*$_5$** remained about the same as that of **8** in the parent (undeuterated) reaction [Table 1 entry 3, reproduced as Table 5, entry 3]. The majority of the *m*-xylene **10** formed in this experiment was not labelled. The isotope effect observed **8-*d*$_5$** is not consistent with a BHAS mechanism but is consistent with an organometallic mechanism, but the isotope effect observed for **9-*d*$_{10}$** is consistent with this compound being formed by a BHAS mechanism.

Reaction of 2-Iodo-*m*-xylene **7** with $Pd(OAc)_2$, KO^{t}Bu, dppf and TEMPO (50 mol%) (Table 6, Entry 1)

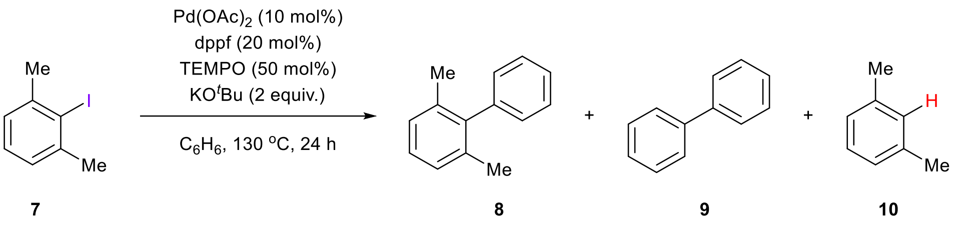

The reaction was conducted according to General Procedure A with $Pd(OAc)_2$ (16 mg, 0.07 mmol, 10 mol%), dppf (79 mg, 0.14 mmol, 20 mol%), TEMPO (55 mg, 0.35 mmol, 50 mol%) KO^{t}Bu (157 mg, 1.4 mmol, 2 equiv.), benzene (7 mL).

GCFID data below include tables quantitating components that had been calibrated (for calibrations see pages S73 - S77).

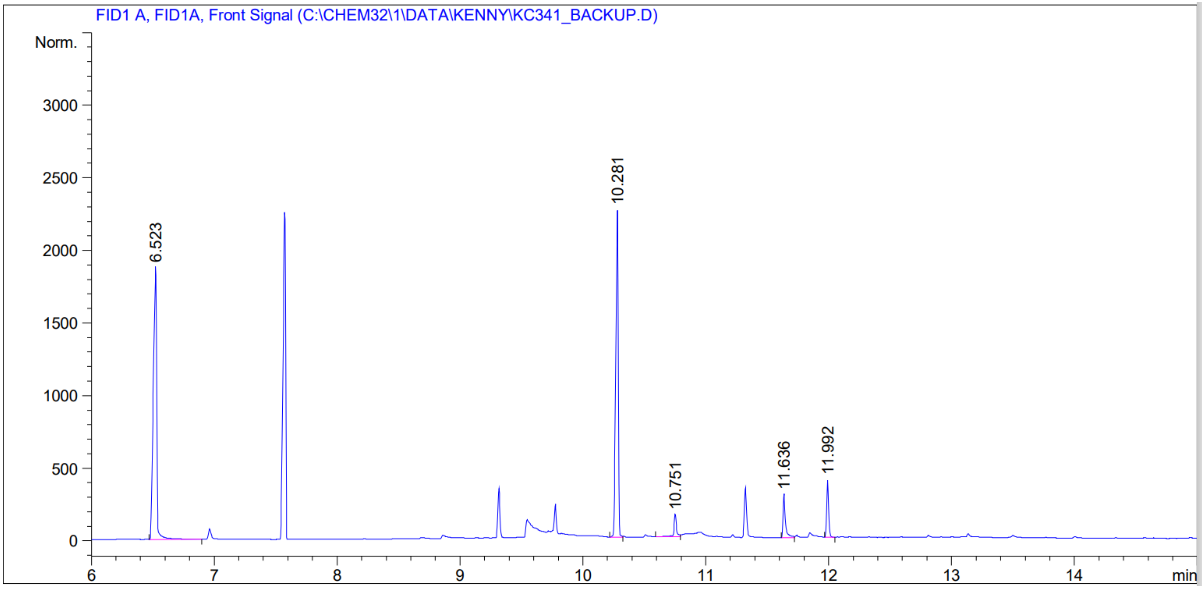

Retention Time	Sample	Peak Area	%Yield
6.523	*m*-Xylene **10**	3681.59058	23.5%
10.281	Dodecane	3184.57202	N/A
10.751	2-Iodo-*m*-xylene **7**	213.29393	1.4%
11.636	Biphenyl **9**	403.45068	1.8%
11.992	2,6-Dimethylbiphenyl **8**	438.30115	1.6%

*This reaction was carried out in duplicate with the average below

Sample	%Yield
m-Xylene **10**	22.5%
2-Iodo-*m*-xylene **7**	1.4%
Biphenyl **9**	1.8%
2,6-Dimethylbiphenyl **8**	1.6%

Outcome - The reaction of 2-iodo-*m*-xylene **7** with $Pd(OAc)_2$, KO^{t}Bu, dppf and TEMPO (50 mol%) led to a significant inhibition in the formation of the biaryl compounds **8** and **9**. This is consistent with interception of radical chemistry by TEMPO.

<u>Reaction of 2-Iodo-*m*-xylene **7** with $Pd(PPh_3)_4$, KO^{t}Bu and TEMPO (50 mol%) (Table 6, Entry 2)</u>

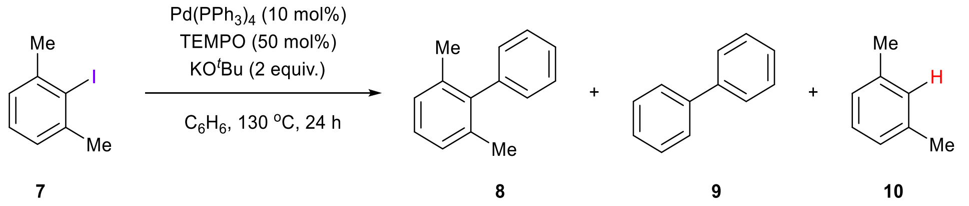

The reaction was conducted according to General Procedure A with $Pd(PPh_3)_4$ (81 mg, 0.07 mmol, 10 mol%), dppf (79 mg, 0.14 mmol, 20 mol%), TEMPO (55 mg, 0.35 mmol, 50 mol%) KO^{t}Bu (157 mg, 1.4 mmol, 2 equiv.), benzene (7 mL).

GCFID data below include tables quantitating components that had been calibrated (for calibrations see pages S73 - S77).

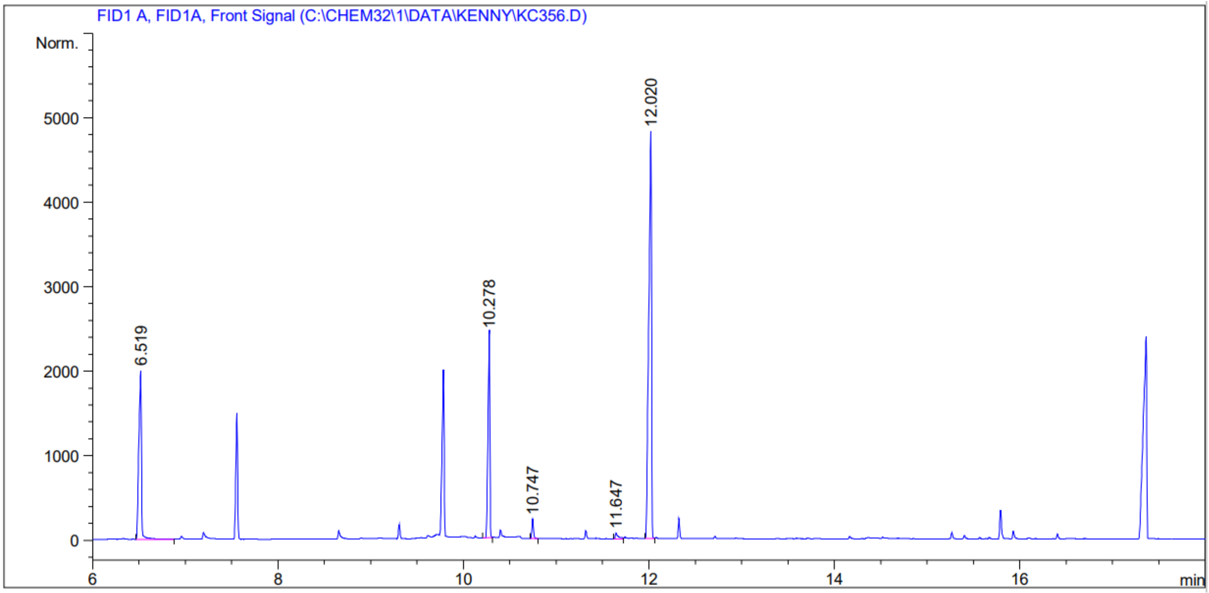

Retention Time	Sample	Peak Area	%Yield
6.519	*m*-Xylene **10**	4004.33496	22.5%
10.278	Dodecane	3507.36987	N/A
10.747	2-Iodo-*m*-xylene **7**	264.33633	1.7%
11.647	Biphenyl **9**	149.64127	0.7%
12.020	2,6-Dimethylbiphenyl **8**	9922.42773	36.4%

*This reaction was carried out in duplicate with the average below

Sample	%Yield
m-Xylene **10**	21.6%

2-Iodo-*m*-xylene **7**	2.2
Biphenyl **9**	0.7%
2,6-Dimethylbiphenyl **8**	34.7%

Outcome - The reaction of 2-iodo-*m*-xylene **7** with $Pd(PPh_3)_4$, KO^{t}Bu and TEMPO (50 mol%) led to no inhibition in the formation of **8**. This result is not consistent with radical (BHAS) chemistry. The formation of **9** was inhibited, consistent with this product being formed by a BHAS mechanism (involving radicals).

Preparation of Pd^{II}(dppf)(I)(xylyl) **18**

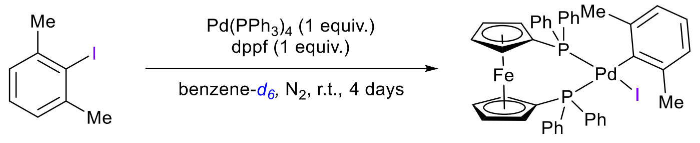

7 **18**

An oven-dried microwave vial was transferred to a glovebox, where $Pd(PPh_3)_4$ (400 mg, 0.35 mmol, 1 equiv.), dppf (192 mg, 0.35 mmol, 1 equiv.), 2-iodo-*m*-xylene **7** (58 µL, 1.15 mmol, 1.2 equiv.) and benzene-*d*$_6$ (6 mL) was added, with the vial subsequently sealed left to stand for 4 days. Once complete, pentane was slowly added to the solution until a yellow precipitate formed. This was then filtered by vacuum filtration to yield the Pd complex, Pd^{II}(dppf)(I)(xylyl) **18** (123 mg, 0.14 mmol, 41%) as brown/yellow crystals. **^{1}H NMR** (400 MHz, C_6D_6) δ 8.37 (br s, 4H), 7.79 – 7.69 (m, 1H), 7.36 (br s, 1H), 7.26 – 7.19 (m, 4H), 7.13 – 7.10 (m, 1H), 7.07 – 6.97 (m, 2H), 6.96 (br s, 1H), 6.91 – 6.85 (m, 2H), 6.76 – 6.71 (m, 4H), 6.69 – 6.64 (m, 1H), 6.60 (br s, 2H), 4.66 (br s, 2H), 4.06 (s, 2H), 3.64 – 3.57 (m, 4H), 2.82 (s, 6H). **^{31}P NMR** (162 MHz, C_6D_6) δ 25.65 (d, *J* = 37.9 Hz), 7.85 (d, *J* = 37.8 Hz). ***m/z*** (ESI+) 765 ($[M-I]^+$).

Treatment of Pd^{II}(dppf)(I)(xylyl) **18** with KO^{t}Bu in C_6H_6 (Scheme 5)

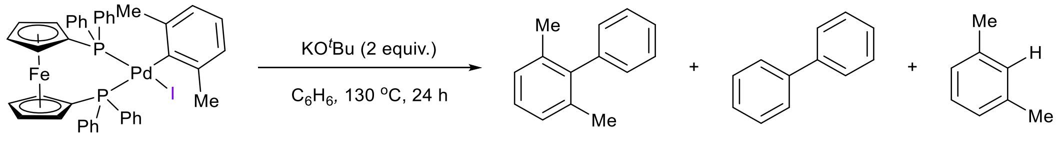

18 **8** **9** **10**

To an oven-dried microwave vial, primed with a stirrer bar, in a glovebox was added Pd(dppf)(xylyl)(I) **18** (67 mg, 0.07 mmol, 1 equiv.), KO^{t}Bu (157 mg, 1.4 mmol, 2 equiv.) and benzene (2 mL) with the vial subsequently sealed and stirred at 130 °C for 24 h. Once complete, the crude mixture was allowed to cool to room temperature and dodecane (23 µL) was added and mixed. A 100 µL aliquot of the crude mixture was then analysed by both GCMS and GCFID.

GCFID data including table quantitating components that had been quantitatively calibrated (for calibrations see pages S73 - S77).

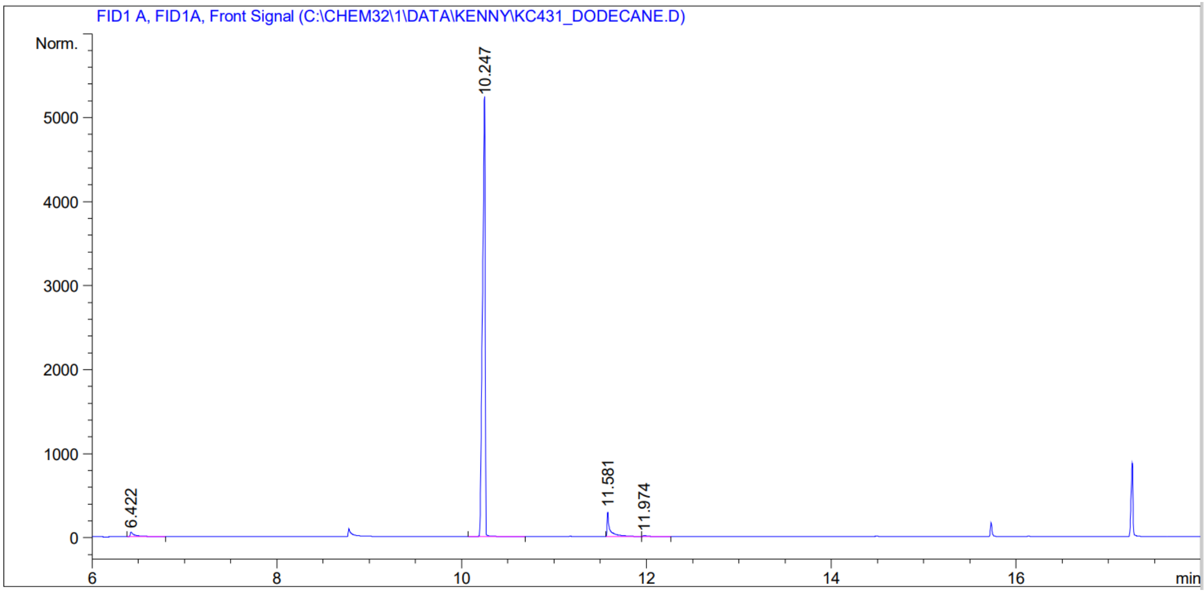

Retention Time	Sample	Peak Area	%Yield
6.422	*m*-Xylene	229.41019	4.1%
10.247	Dodecane	10623.4	N/A
11.581	Biphenyl	710.67035	8.9%
11.974	2,6-Dimethylbiphenyl	76.52856	0.8%

Outcome - The Reaction of Pd^{II}(dppf)(I)(xylyl) **18** with KO^tBu led to comparatively large quantity of biphenyl **9** when compared with **8**. These results are not consistent with that of radical chemistry. The biphenyl **9** in this reaction was found to form *via* liberation of the phenyl groups on phosphines of **18**. (see below)

Treatment of Pd^{II}(dppf)(I)(xylyl) **18** with KO^tBu in C_6D_6 (Scheme 5)

To an oven-dried microwave vial, primed with a stirrer bar, in a glovebox was added Pd(dppf)(xylyl)(I) **18** (67 mg, 0.07 mmol, 1 equiv.), KO^tBu (157 mg, 1.4 mmol, 2 equiv.) and benzene-d_6 (2 mL) with the vial subsequently sealed and stirred at 130 °C for 24 h. Once complete, the crude mixture was allowed to cool to room temperature and dodecane (23 µL) was added and mixed. A 100 µL aliquot of the crude mixture was then analysed by both GCMS and GCFID.

GCFID data including table quantitating components that had been quantitatively calibrated (for calibrations see pages S73 - S77).

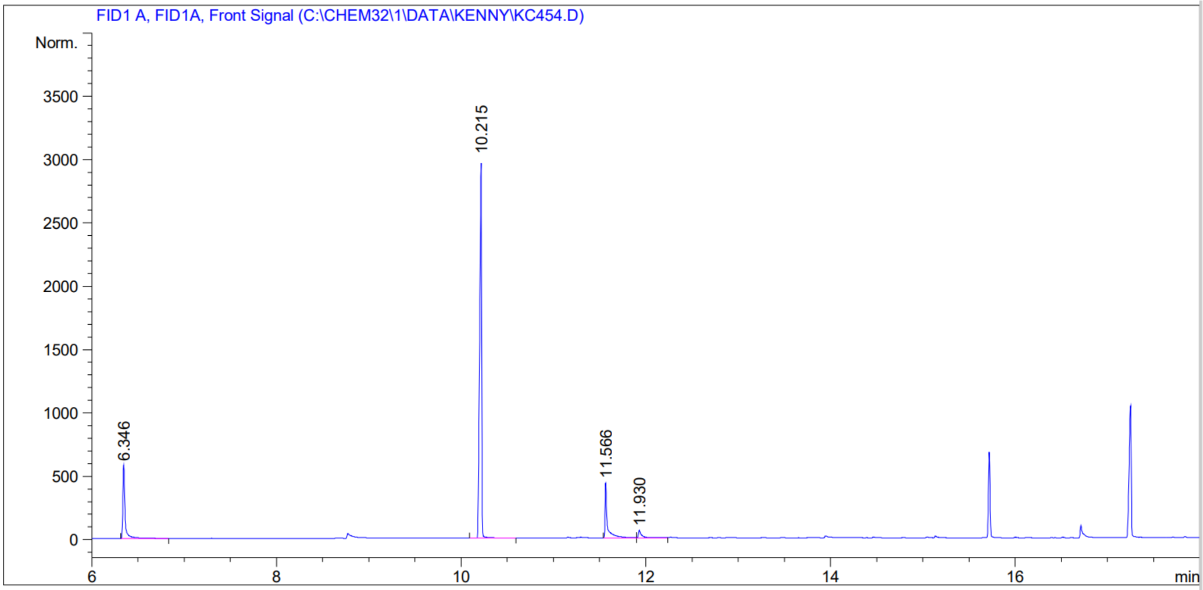

Retention Time	Sample	Peak Area	%Yield
6.346	*m*-Xylene	1009.96545	17.5%
10.215	Dodecane	4542.86377	N/A
11.566	Biphenyl	766.62335	12.1%
11.930	2,6-Dimethylbiphenyl	151.70634	1.5%

*This reaction was carried out in duplicate with the average below

Sample	%Yield
m-Xylene	17.4%
2-Iodo-*m*-xylene	0
Biphenyl	11.2%
2,6-Dimethylbiphenyl	1.3%

For representative mass spectra of **9-*d*$_5$/-*d*$_{10}$**, **8-*d*$_5$** and **10-*d*$_1$** see page S58 et seq.

Outcome - The Reaction of Pd^{II}(dppf)(I)(xylyl) **18** with KO^{t}Bu in C_6D_6 led to comparatively large quantity of biphenyl **9** when compared with **8**. These results are not consistent with that of radical chemistry. The biaryls that formed in this reaction were primarily not labelled, suggesting that the phenyl groups, leading to the formation of **9** and **8** were not resulting from the solvent. The biphenyl **9** in this reaction was found to form *via* liberation of the phenyl groups on phosphines of **18**.

<u>Preparation of Pd^{II}(PCy$_3$)$_2$(I)(xylyl) **19**</u>

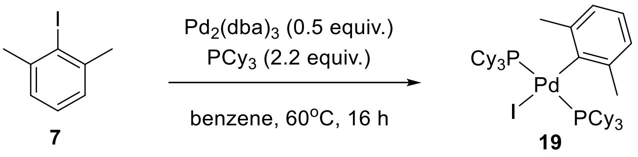

An oven-dried microwave vial was transferred to a glovebox, where $Pd_2(dba)_3$ (457.86 mg, 0.5 mmol, 0.5 equiv.), PCy_3 (617.0 mg, 2.2 mmol, 2.2 equiv.), 2-iodo-m-xylene **7** (173.2 µL, 1.2 mmol, 1.2 equiv.) and benzene (5 mL) was added, with the vial subsequently sealed and removed from the glovebox. The reaction was stirred at room temperature for 2 h, then at 60°C for 16 h yielding a red/brown solution with yellow/green insolubles. The reaction dissolved in toluene (50 mL) and filtered under vacuum to remove Pd black. Solvent was removed under vacuum giving dark orange solid, which was washed with acetone till the washings ran clear yielding a pale orange solid. The solid was then dissolved in DCM, layered with acetone and left overnight at RT yielding **19** as a pale yellow solid (160.8 mg, 0.179 mmol, 18 %). **^{1}H NMR** (400 MHz, C_6D_6) δ 7.01 (t, *J* = 7.3 Hz, 1H), 6.86 (d, *J* = 7.3 Hz, 2H), 2.92 (s, 6H), 2.55 (broad s, 6H), 2.25 – 2.17 (m, 12H), 1.70 (broad d, *J* = 12.0 Hz, 14H), 1.62 – 1.50 (m, 18H), 1.27 – 1.06 (m, 19H). **^{31}P NMR** (162 MHz, C_6D_6) δ 23.90. **HRMS *m/z*** 771.4364 $(M-I)^+$. Calc $C_{44}H_{75}P_2{}^{106}Pd$ 771.4379.

Treatment of $Pd^{II}(PCy_3)_2(I)(xylyl)$ **19** with KO^tBu in C_6H_6

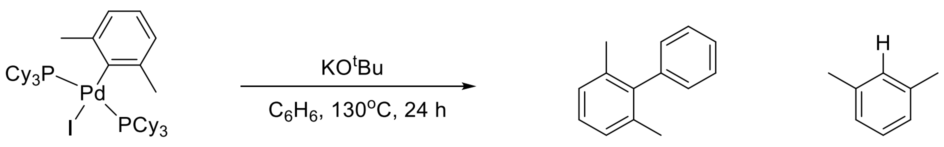

19 **8** **10**

To an oven-dried microwave vial, primed with a stirrer bar, in a glovebox was added $Pd(PCy_3)_2(xylyl)(I)$ **19** (63 mg, 0.07 mmol, 1 equiv.), KOtBu (157 mg, 1.4 mmol, 20 equiv.) and benzene (2 mL) with the vial subsequently sealed and stirred at 130 °C for 24 h. Once complete, the crude mixture was allowed to cool to room temperature and dodecane (25 µL) was added and mixed. A 100 µL aliquot of the crude mixture was then analysed by GCFID.

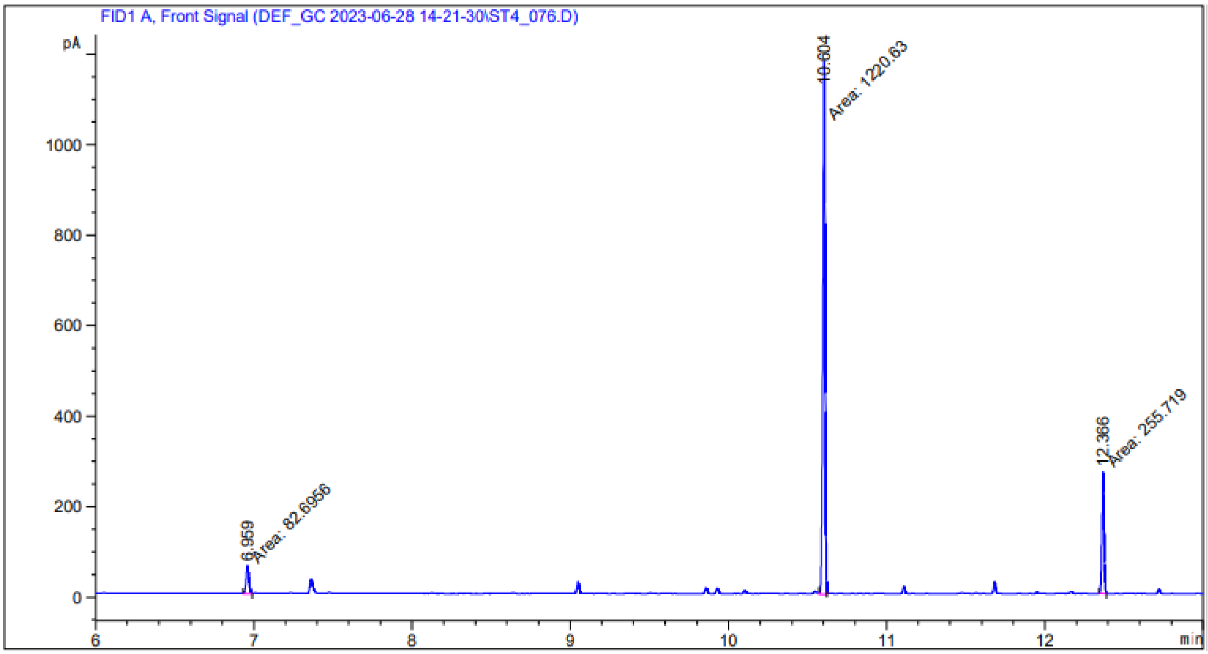

Retention time	Sample	Peak area	% Yield
6.959	*m*-xylene	82.696	15.5
10.604	*n*-dodecane	1220.63	N/A
12.366	2,6-Dimethylbiphenyl	255.719	32.5

In this experiment, dimethylbiphenyl was produced as the major product, with no biphenyl being produced.

Identity of 1,2,3-trimethylbenzene in GCMS from experiment (i) with Pd(OAc)$_2$ and (ii) with organic additive, phenanthroline

Co-injection of 1,2,3-trimethylbenzene to Pd(OAc)$_2$/dppf BHAS reaction

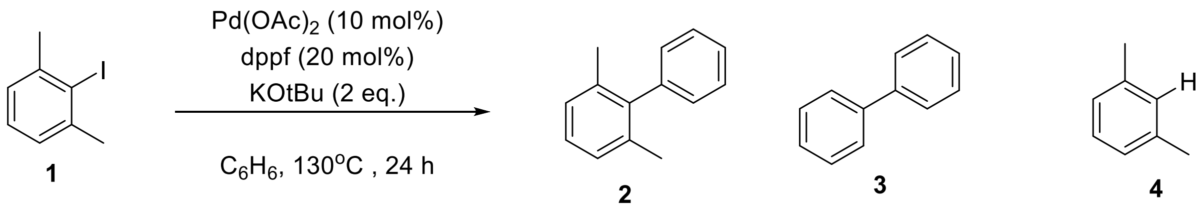

After the reaction and addition of a dodecane IS, a 100 µL aliquot was removed from the reaction mixture and diluted with $CHCl_3$ for analysis by GC-MS/GC-FID. Then 150 µL of a 0.075 M solution of 1,2,3-trimethylbenzene in DCM was added to the reaction mixture, after which a further 100 µL aliquot was removed from the reaction mixture and diluted with $CHCl_3$ for analysis by GC-MS/GC-FID. The increased intensity of the peak at 7.04 mins confirms the identity of the species as 1,2,3-trimethylbenzene.

GC-MS traces without added 1,2,3-trimethylbenzene:

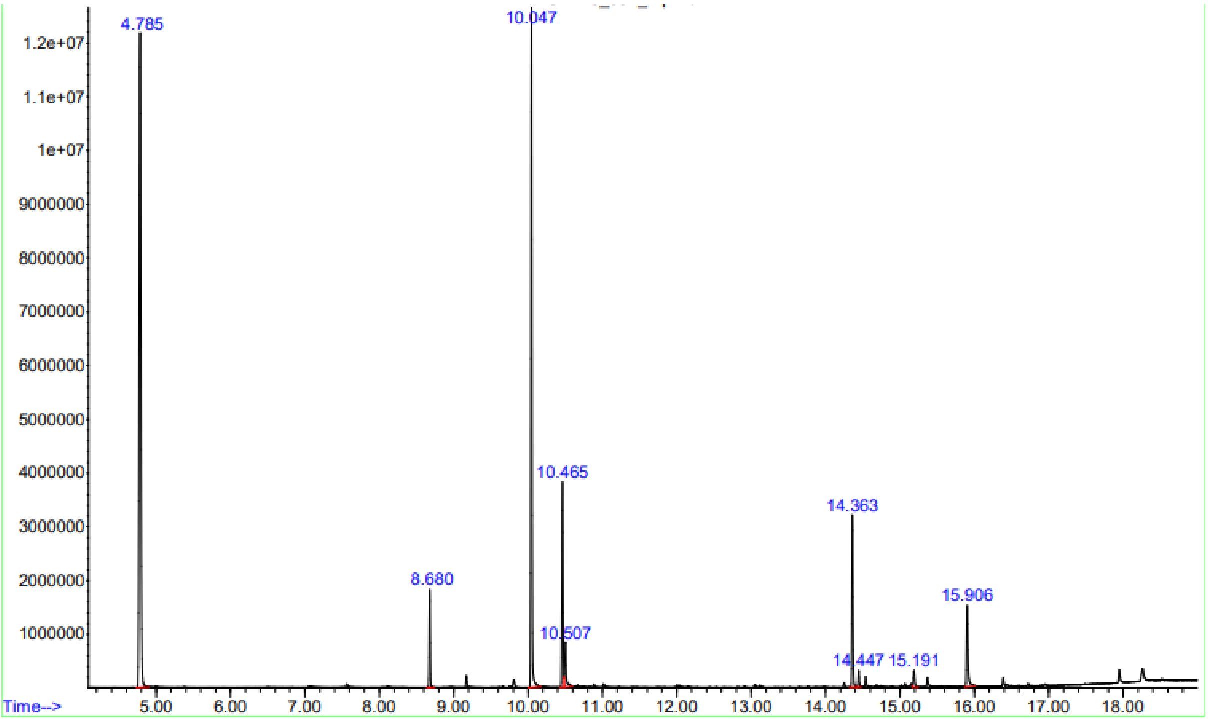

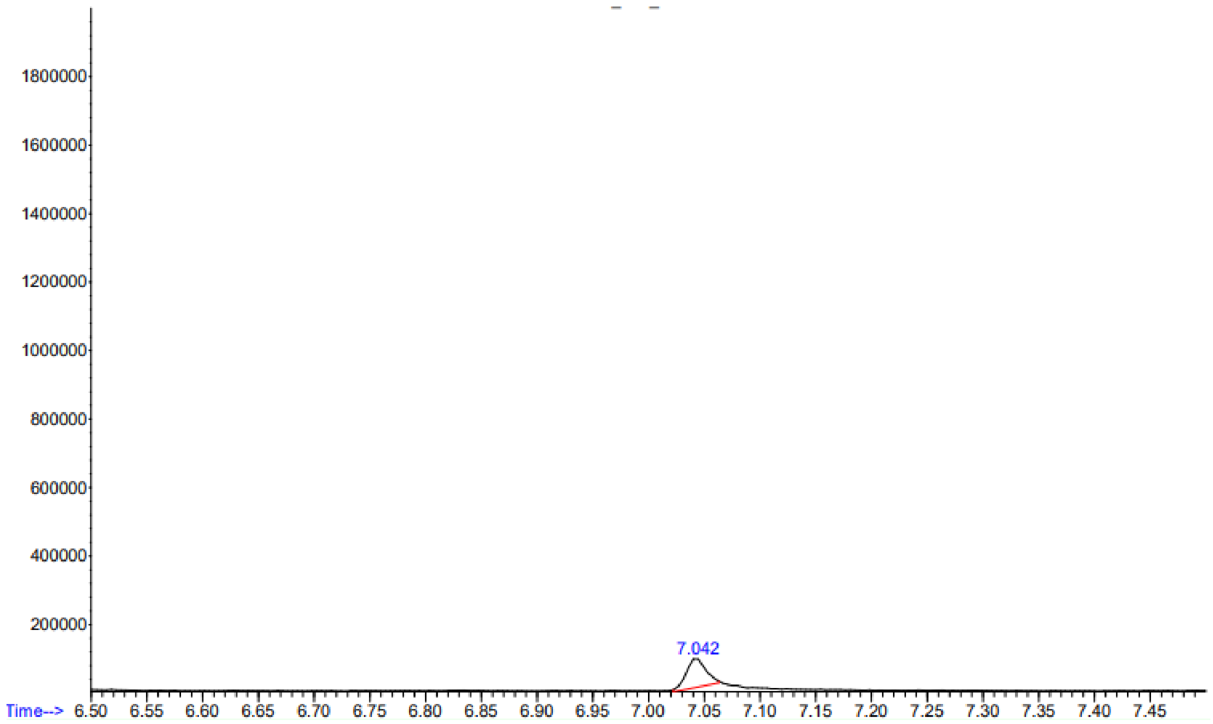

GC-MS traces with added 1,2,3-trimethylbenzene:

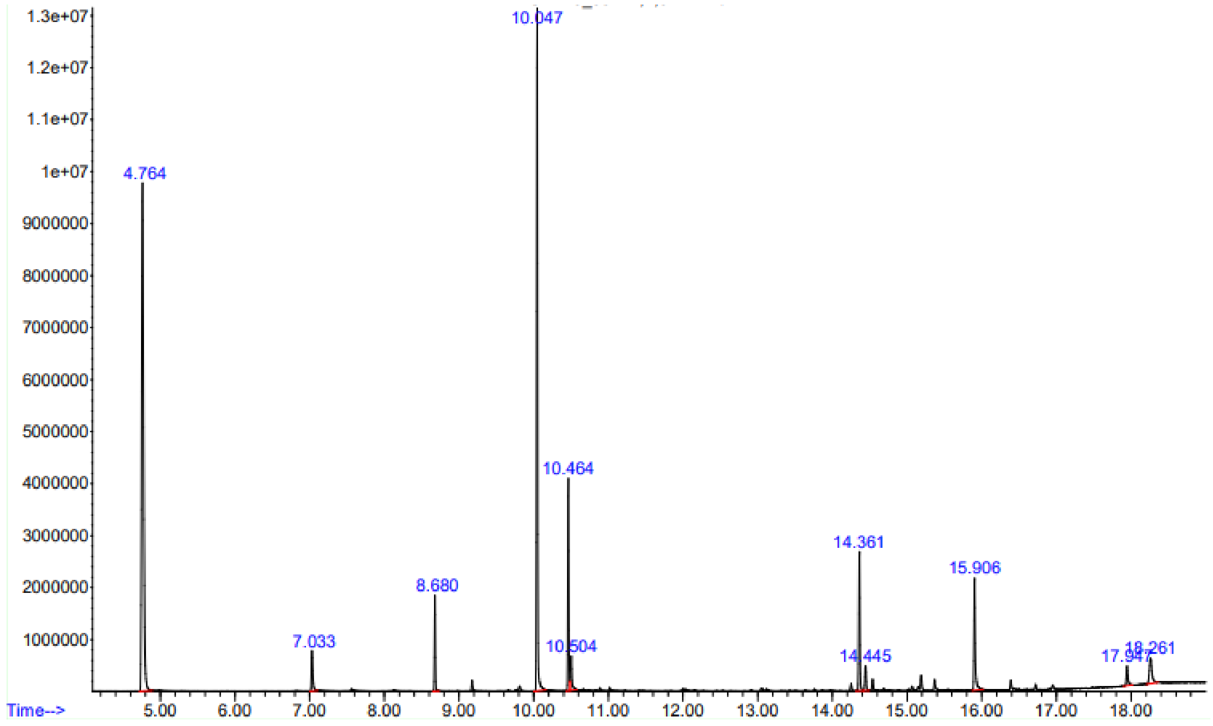

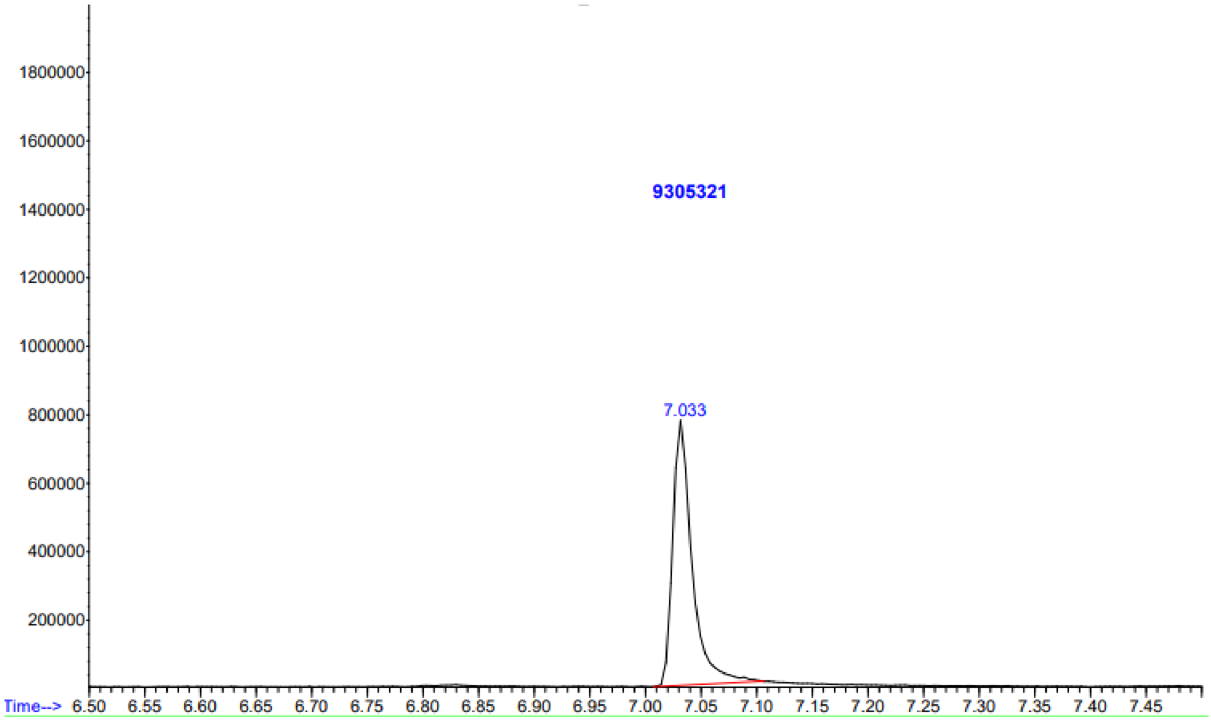

1800000
1600000
1400000
1200000
1000000
800000
600000
400000
200000
9305321
7.033
Time--> 6.50 6.55 6.60 6.65 6.70 6.75 6.80 6.85 6.90 6.95 7.00 7.05 7.10 7.15 7.20 7.25 7.30 7.35 7.40 7.45

Co-injection of 1,2,3-trimethylbenzene to phenanthroline BHAS reaction

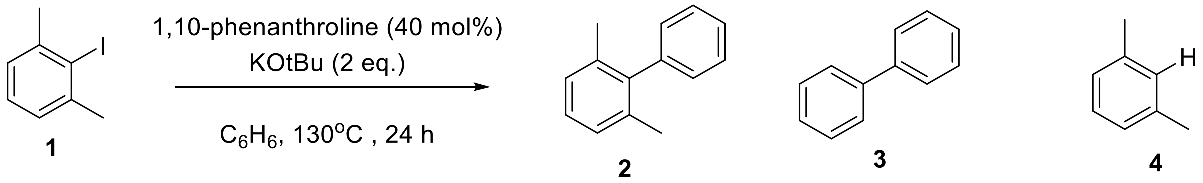

After the reaction and addition of a dodecane IS, a 100 µL aliquot was removed from the reaction mixture and diluted with $CHCl_3$ for analysis by GC-MS/GC-FID. Then 150 µL of a 0.075 M solution of 1,2,3-trimethylbenzene in DCM was added to the reaction mixture, after which a further 100 µL aliquot was removed from the reaction mixture and diluted with $CHCl_3$ for analysis by GC-MS/GC-FID. The increased intensity of the peak at 7.04 mins confirms the identity of the species as 1,2,3-trimethylbenzene.

GC-MS traces without added 1,2,3-trimethylbenzene:

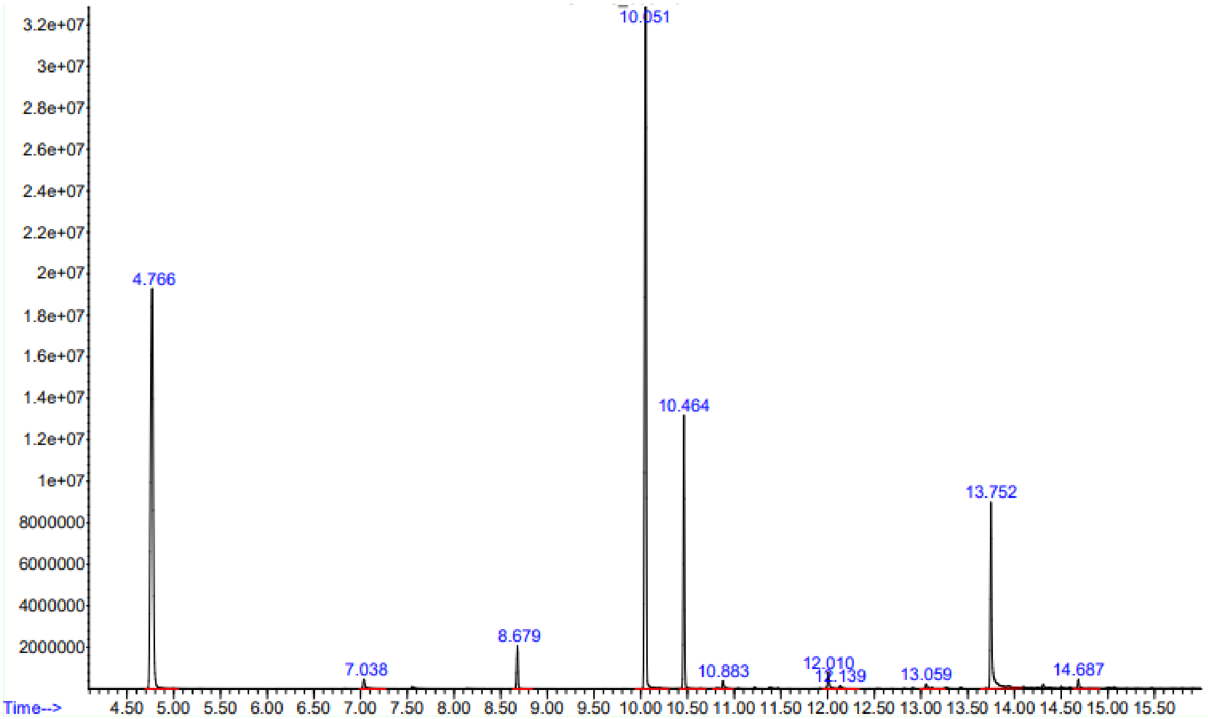

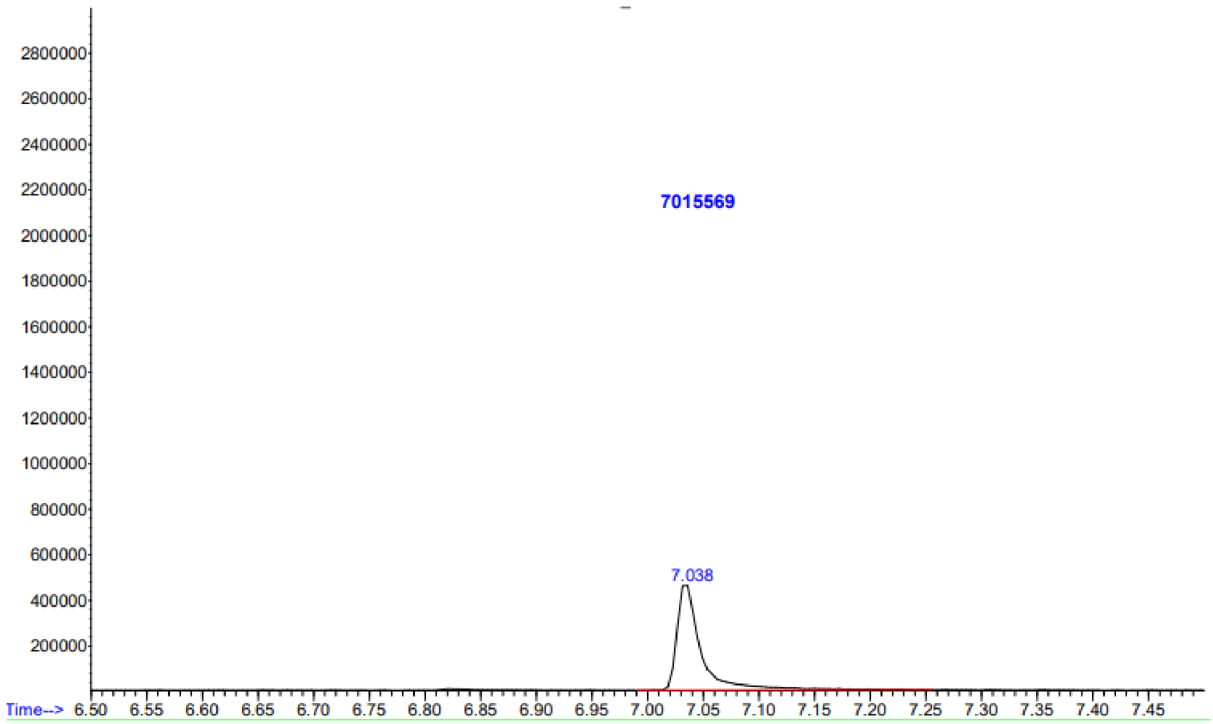

GC-MS traces with added 1,2,3-trimethylbenzene:

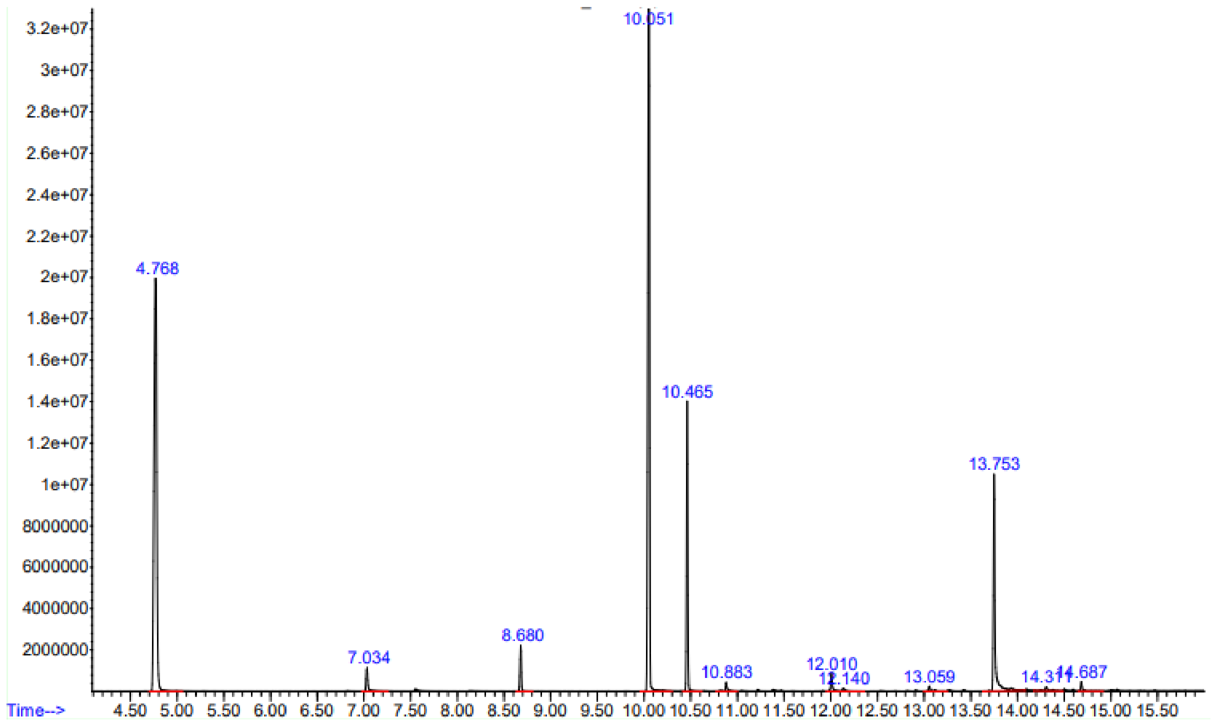

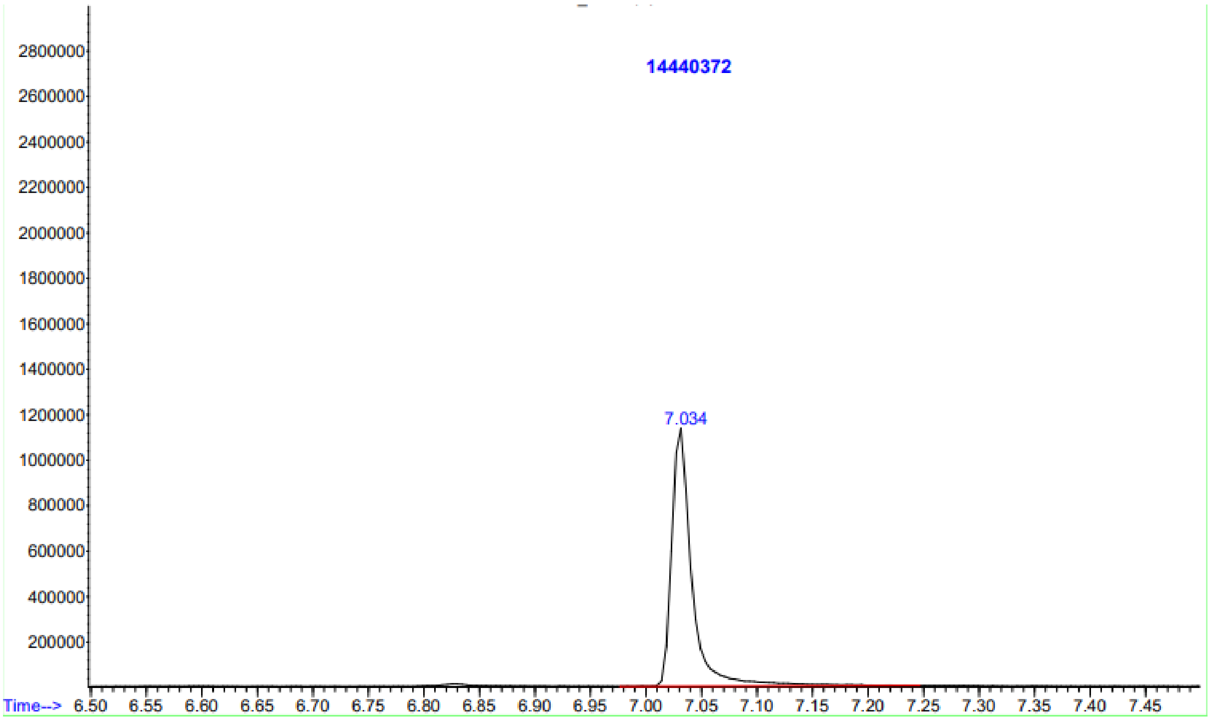
14440372
7.034
2800000
2600000
2400000
2200000
2000000
1800000
1600000
1400000
1200000
1000000
800000
600000
400000
200000
Time-->
6.50 6.55 6.60 6.65 6.70 6.75 6.80 6.85 6.90 6.95 7.00 7.05 7.10 7.15 7.20 7.25 7.30 7.35 7.40 7.45

Identity of D3-trimethylbenzene data

BHAS with $Pd(OAc)_2$ / 20 mol% PPh_3 and d_9-KO^{t}Bu in C_6H_6

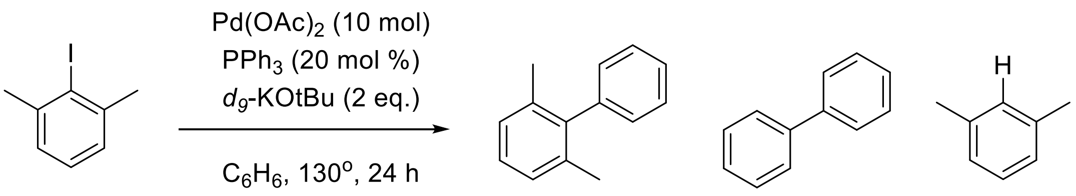

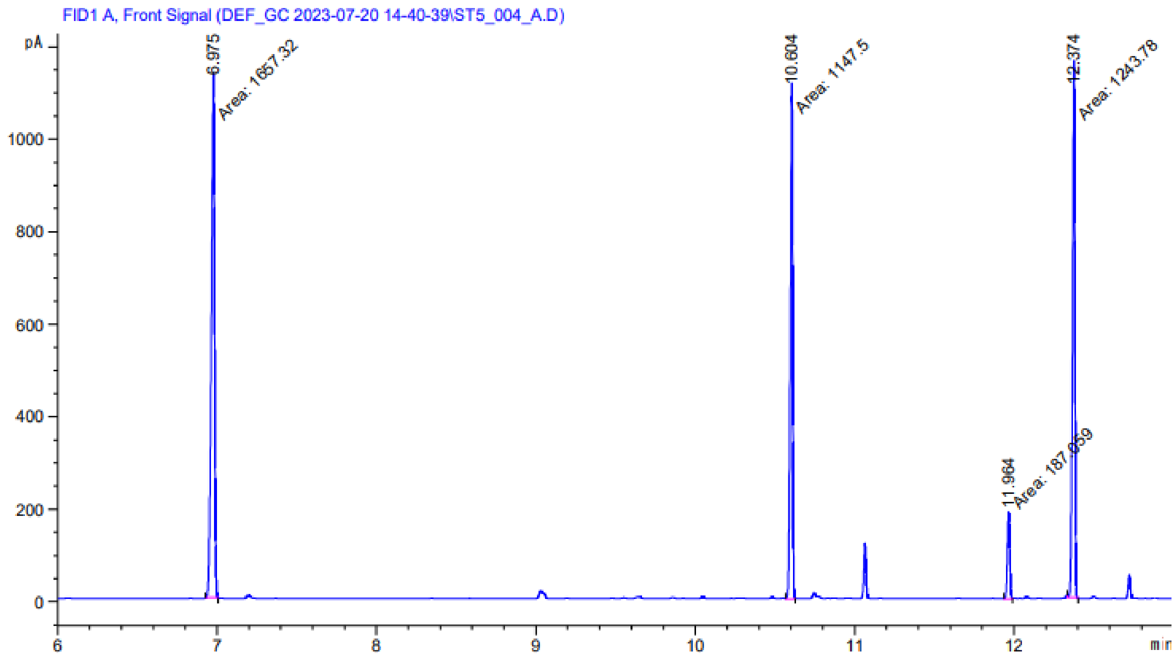

Retention time	Sample	Peak area	% Yield
6.975	*m*-xylene **10**	1657.320	45.1
10.604	*n*-dodecane	1147.503	N/A
11.114	Iodoxylene **7**	0	0
11.964	Biphenyl **9**	187.059	3.8
12.374	2,6-Dimethylbiphenyl **8**	1243.783	23.0

In this experiment, biphenyl and dimethylbiphenyl were completely non-deuterated as expected. The 1,2,3-trimethylbenzene was a mixture of *m/z* 120, 122 and 123 isotopologues, as shown in the below GC-MS trace.

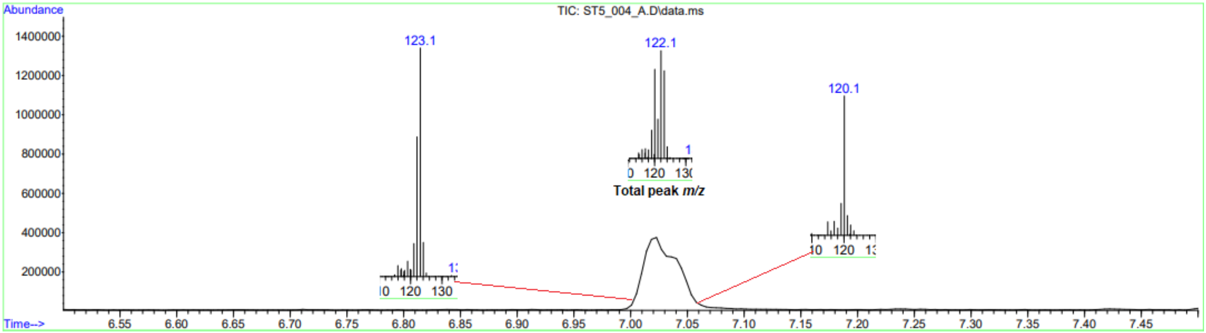

BHAS with $Pd(OAc)_2$ / 20 mol% PPh_3 and d_9-KO^{t}Bu in C_6D_6

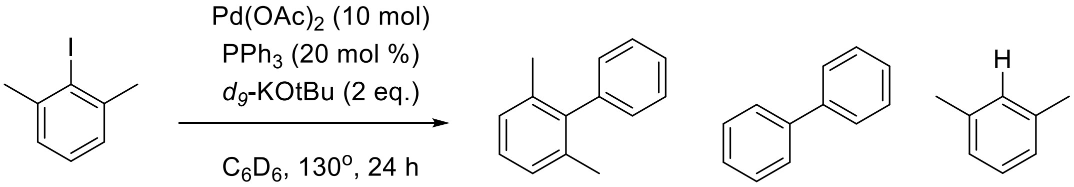

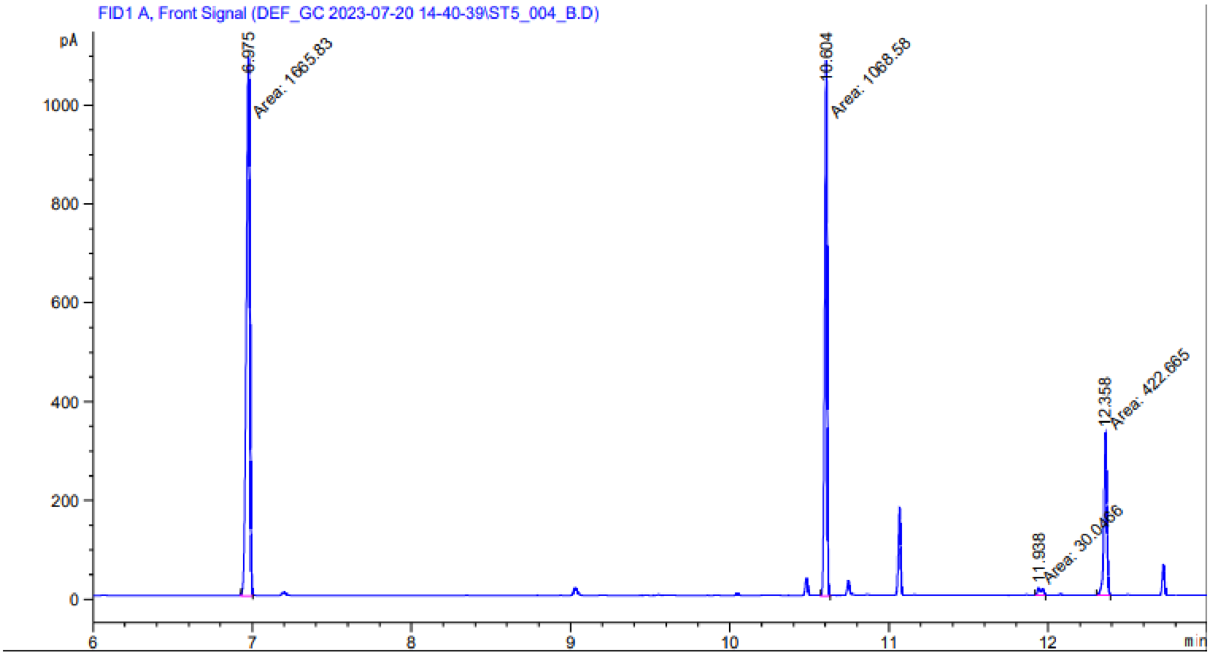

Retention time	Sample	Peak area	% Yield
6.975	*m*-xylene	1665.83	48.7
10.604	*n*-dodecane	1068.585	N/A
11.114	iodoxylene	0	0
11.938	Biphenyl	30.047	0.7
12.358	2,6-Dimethylbiphenyl	422.665	8.4

In this experiment, the biphenyl produced was a mixture of H10 and D10 with small quantities of H5D5. The dimethylbiphenyl was predominantly D5, with small quantities of H5. The 1,2,3-trimethylbenzene was almost entirely the *m/z* 123 isotopologue, as shown in the below GC-MS trace.

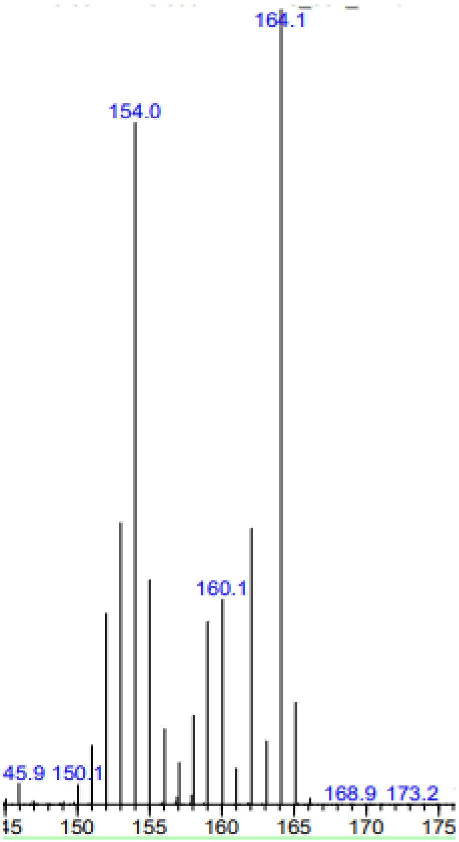

Biphenyl peak

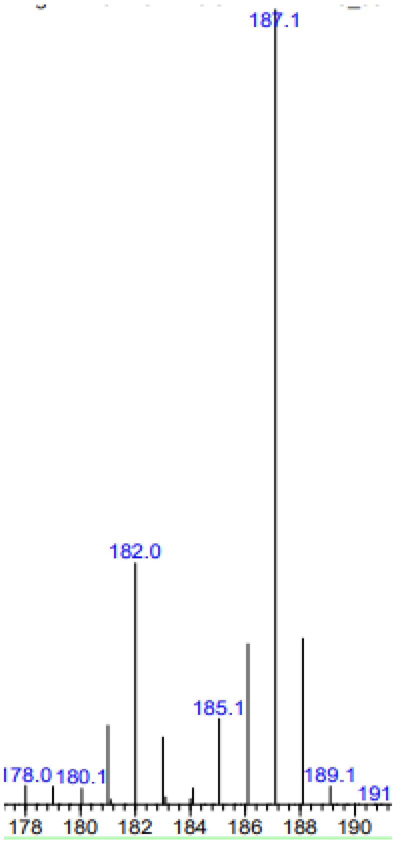

Dimethylbiphenyl peak

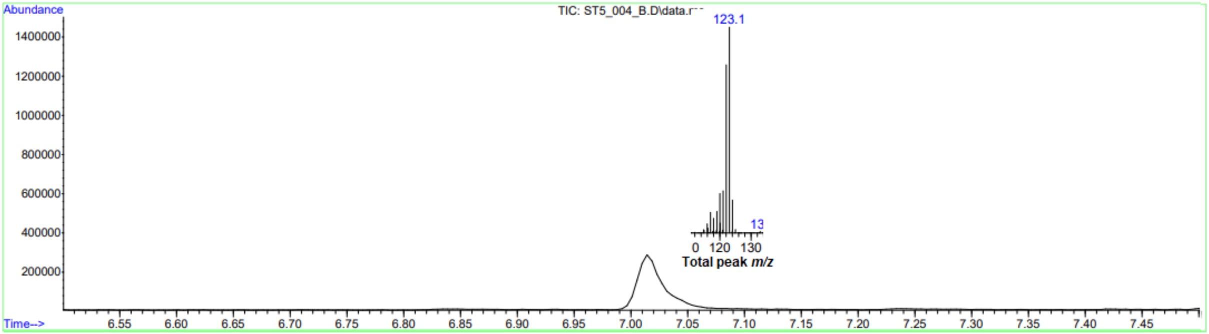

Example GCMS Mass Traces

Mass spectra obtained for the products that were commonly found (together with their approximate retention times on GCMS):

m-Xylene – Retention time = ~6.2 min

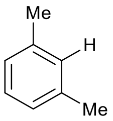

10

Predicted *m/z*: 106.08 (100.0%), 107.08 (8.8%)

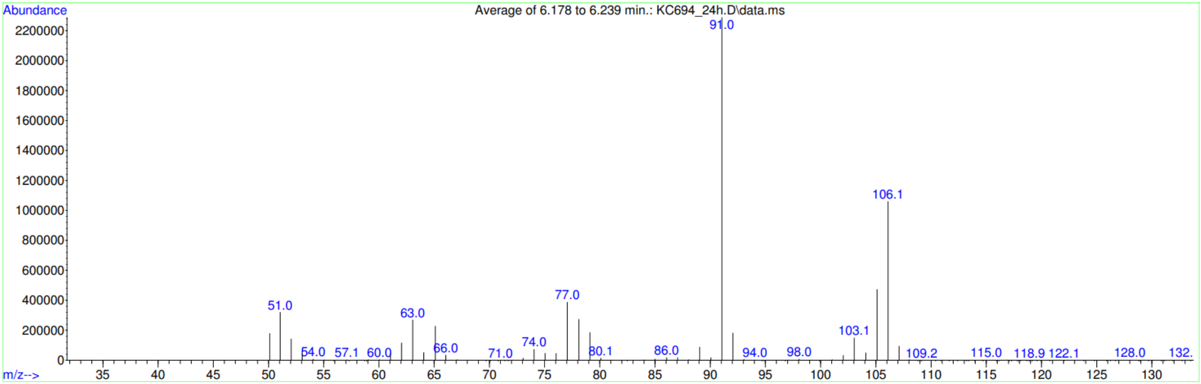

2-Iodo-*m*-xylene – Retention time = ~10.3 min

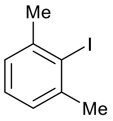

7

Predicted *m/z*: 231.97 (100.0%), 232.98 (8.8%)

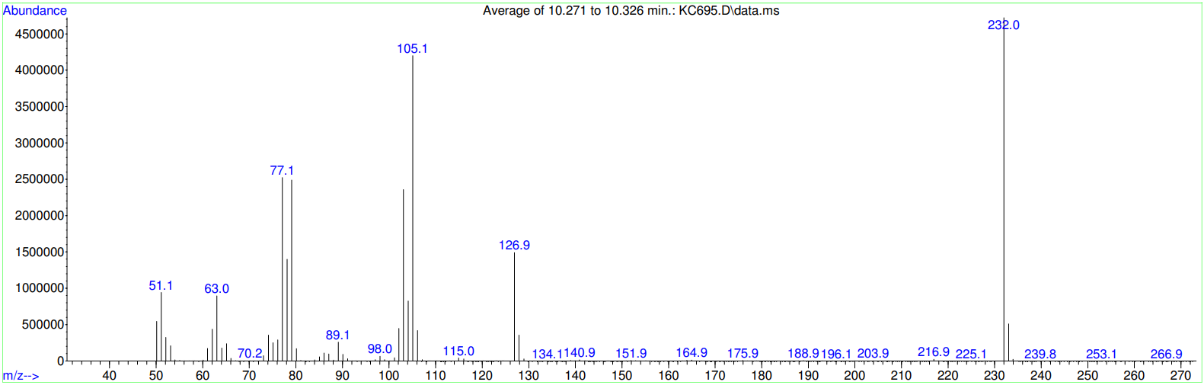

Biphenyl – Retention time = ~11.1 min

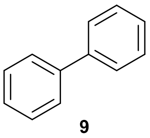

9

Predicted *m/z*: 154.0783 (100.0%), 155.0816 (13.0%)

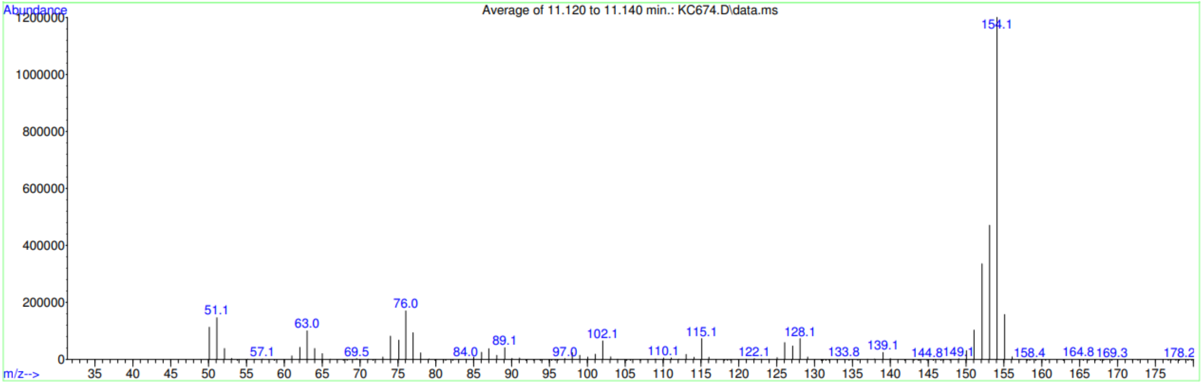

2,6-Dimethylbiphenyl – Retention time = ~11.5 min

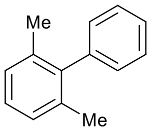

8

Predicted *m/z*: 182.11 (100.0%), 183.11 (15.1%), 184.12 (1.1%)

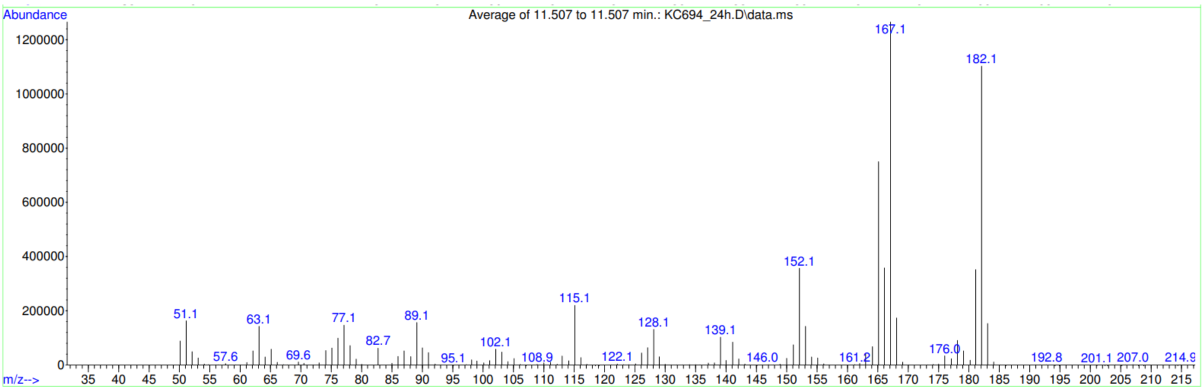

Individual Mass Spectra of Deuterated Compounds

Method of analysis – Picking the peaks on the GCMS software generates an average mass across the peak area. In some cases, the deuterated compounds have slightly differing retention times when compared to their unlabelled counterparts. Analysing across sections of the peak area at different time points can provide different mass ions to show.

This is best demonstrated with *m*-xylene **10**: when conducting the reaction of 2-iodo-*m*-xylene **7** with KO^{t}Bu and 1,10-phenanthroline C_6D_6 the average mass across the whole *m*-xylene peak is the unlabelled compound *m/z* = 106. However, averaging the very start of the peak does indeed highlight that some **10-*d*$_1$** has formed as demonstrated with the mass spectrum below showing the *m/z* of 107.

This method of analysis was used for all isotope studies.

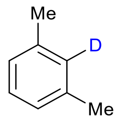

10-*d*$_1$
Predicted *m/z*: 107.0845 (100.0%), 108.0879 (8.7%)
Retention Time = ~6.1 min

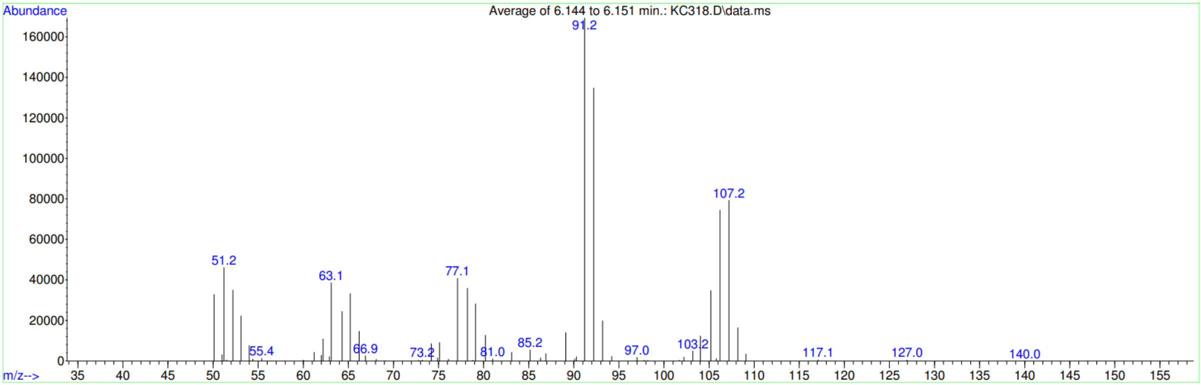

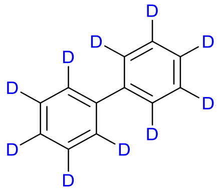

9-*d*$_{10}$
Predicted *m/z*: 164.1410 (100.0%), 165.1444 (13.0%)
Retention Time = ~11.2 min

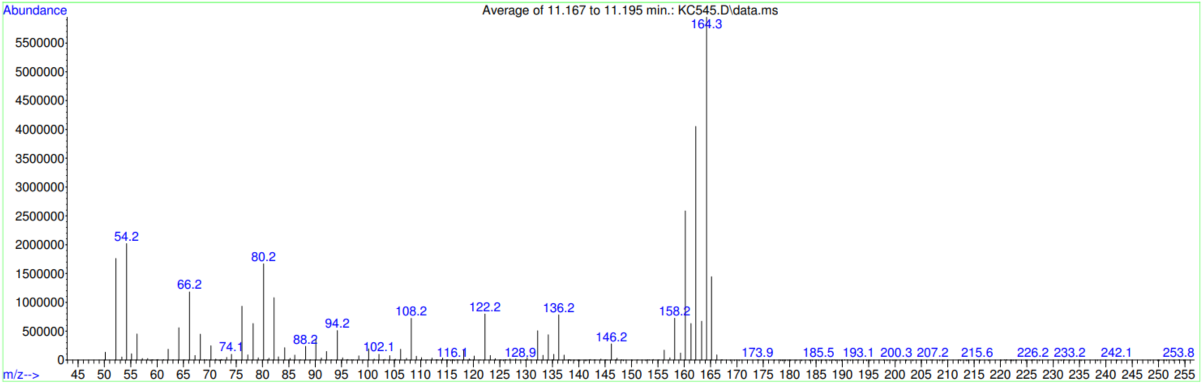

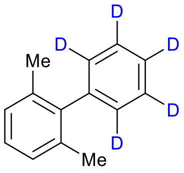

8-d_5
Predicted *m/z*: 187.1409 (100.0%), 188.1443 (15.1%), 189.1476 (1.1%)
Retention Time = ~11.4 min

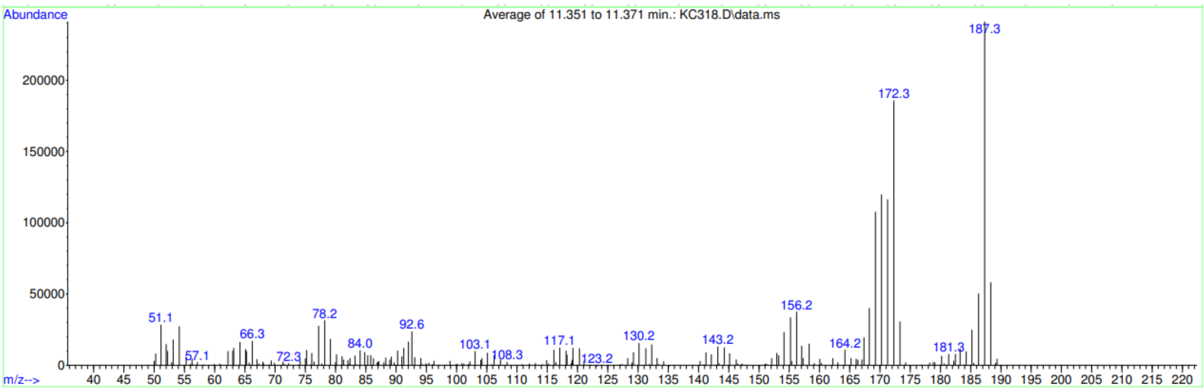

Calibration Information

Method

Commercial samples of 2-iodo-*m*-xylene **7**, biphenyl **9** and *m*-xylene **10** were weighed out at several, different mmol and diluted with chloroform. 2,6-Dimethylbiphenyl **8** was prepared and weighed out at several, different mmol and diluted with chloroform. ~17 µL of dodecane was then added to each sample, with the exact weight of dodecane added noted in each case so the exact mmol added was known. 100 µL aliquots of each solution was then taken and made up to 1 mL with chloroform. Each sample was then run on the GCFID using the GC320 method. Once complete, the peak area for both the dodecane and sample was noted in each case.

To plot graphs for each sample:

- Mmol ratio was calculated by dividing the mmol of sample by mmol of dodecane.
- The area ratio was then calculated by dividing the sample area by the dodecane area.
- Scatter graphs were then plotted using the mmol ratio against the sample area ratio.

Calibration Graphs

The column was changed on the GCFID and so the retention times for all peaks shifted. A repeat calibration was therefore done.

2-Iodo-m-xylene – First Set of Calibration Graphs

2-iodo-*m*-xylene

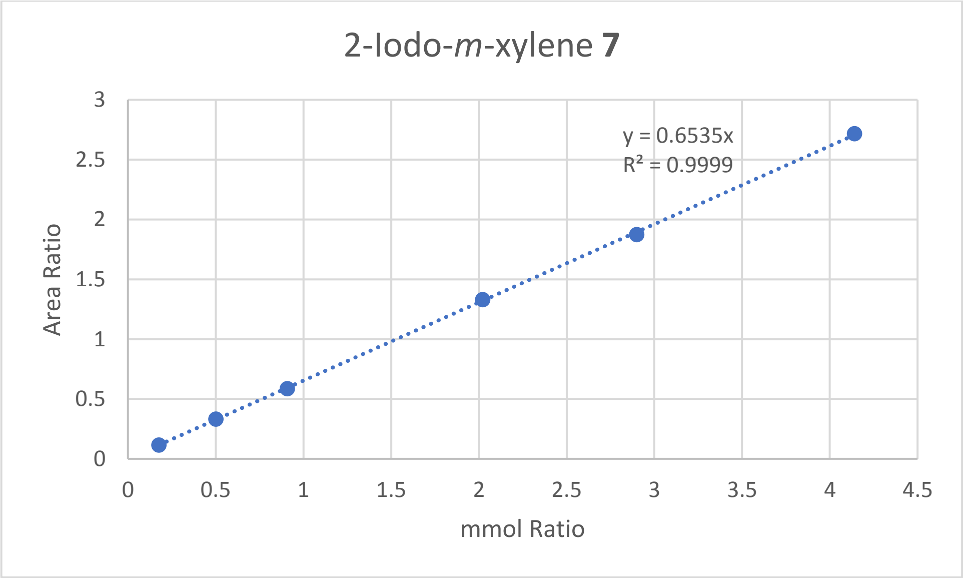

1,1’-Biphenyl

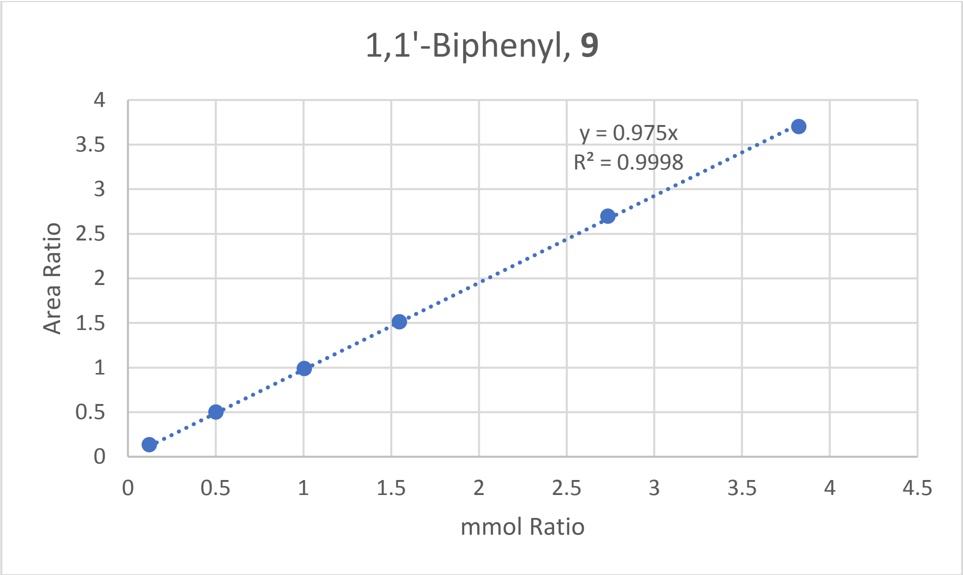

2,6-Dimethylbiphenyl

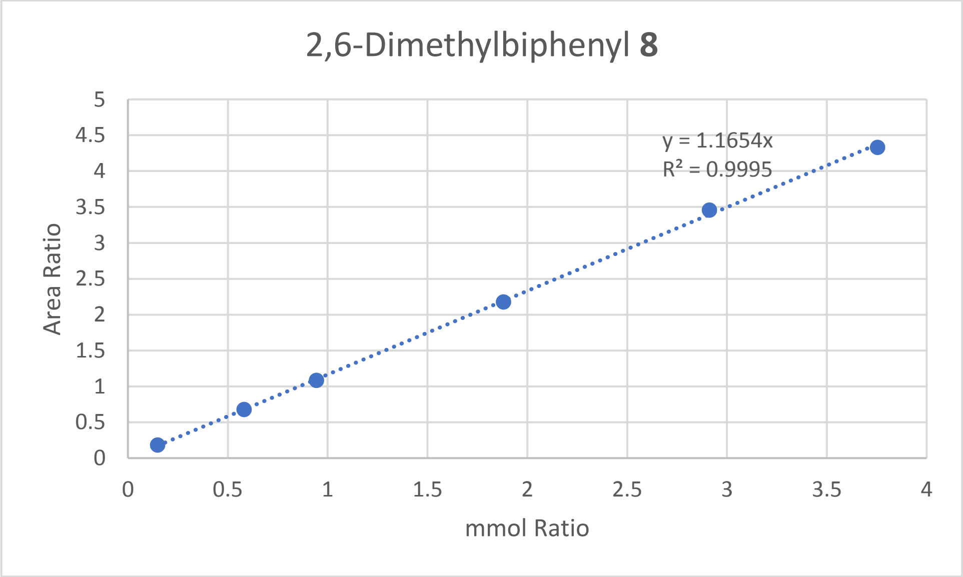

m-Xylene

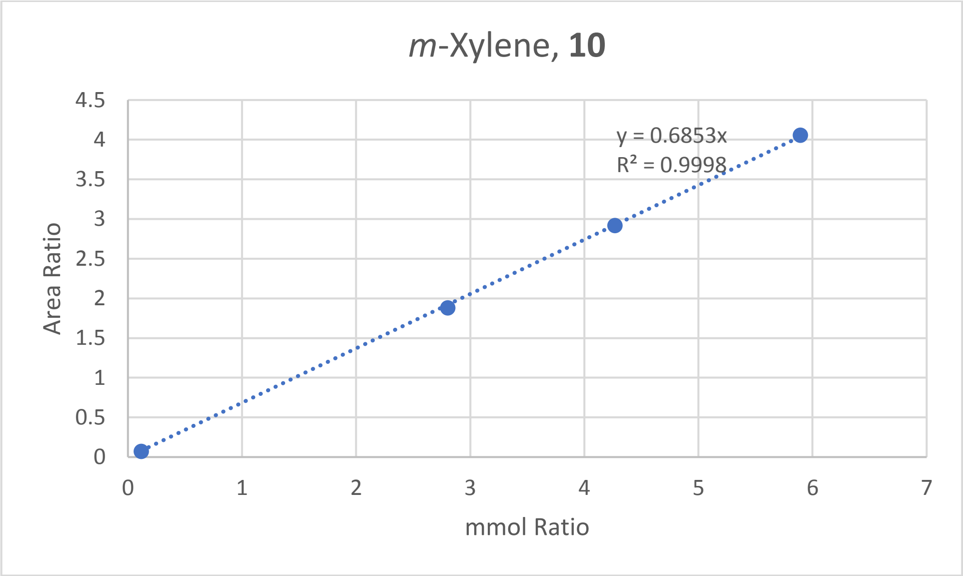

<u>2-Iodo-m-xylene – Second Set of Calibration Graphs</u>

2-iodo-*m*-xylene

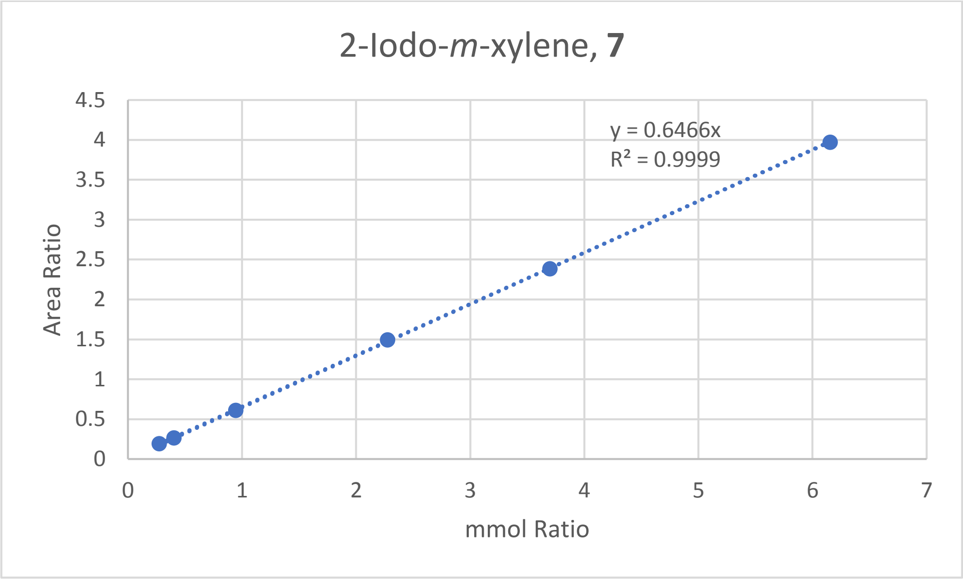

Biphenyl

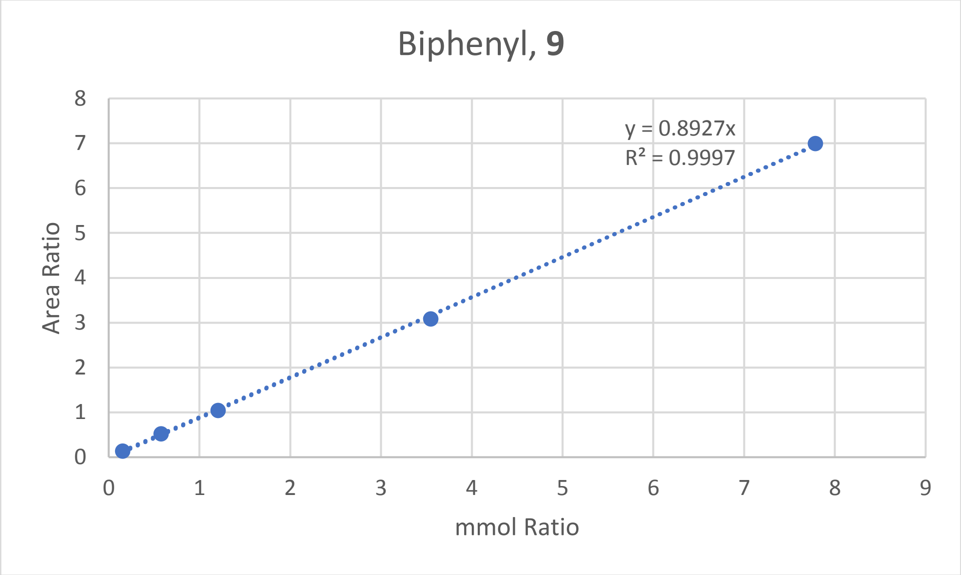

2,6-Dimethylbiphenyl

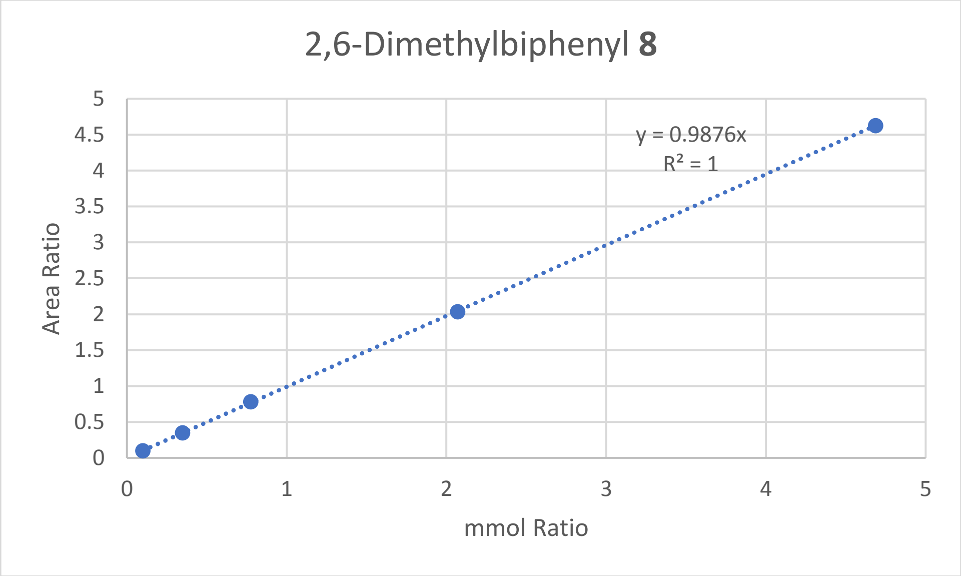

m-Xylene

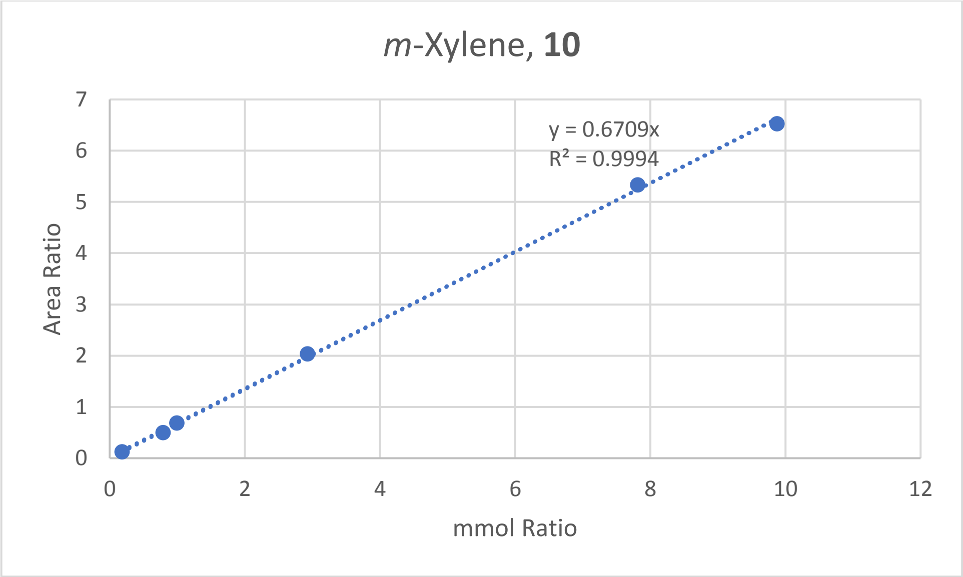
m-Xylene, 10
y = 0.6709x
R² = 0.9994
Area Ratio
mmol Ratio
0
1
2
3
4
5
6
7
0
2
4
6
8
10
12

GC data

2-Iodo-m-xylene – First Set of GCFID Data

2-Iodo-*m*-xylene

0.02 mmol

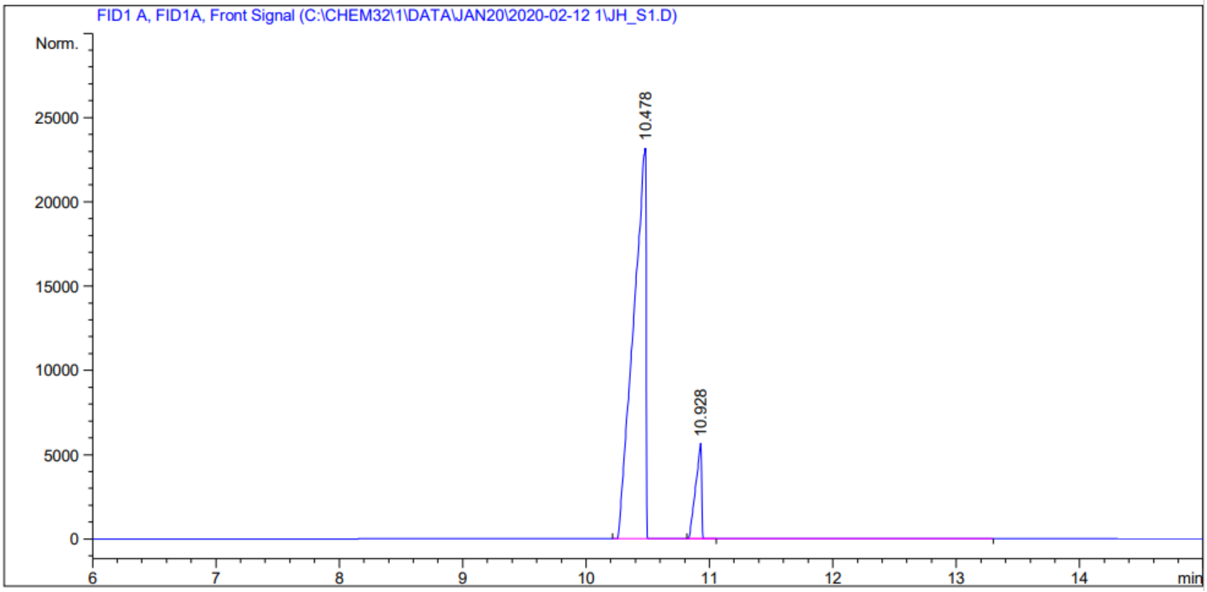

Retention Time	Sample	Peak Area
10.478	Dodecane	164328
10.928	2-Iodo-*m*-xylene **7**	18814.7

0.05 mmol

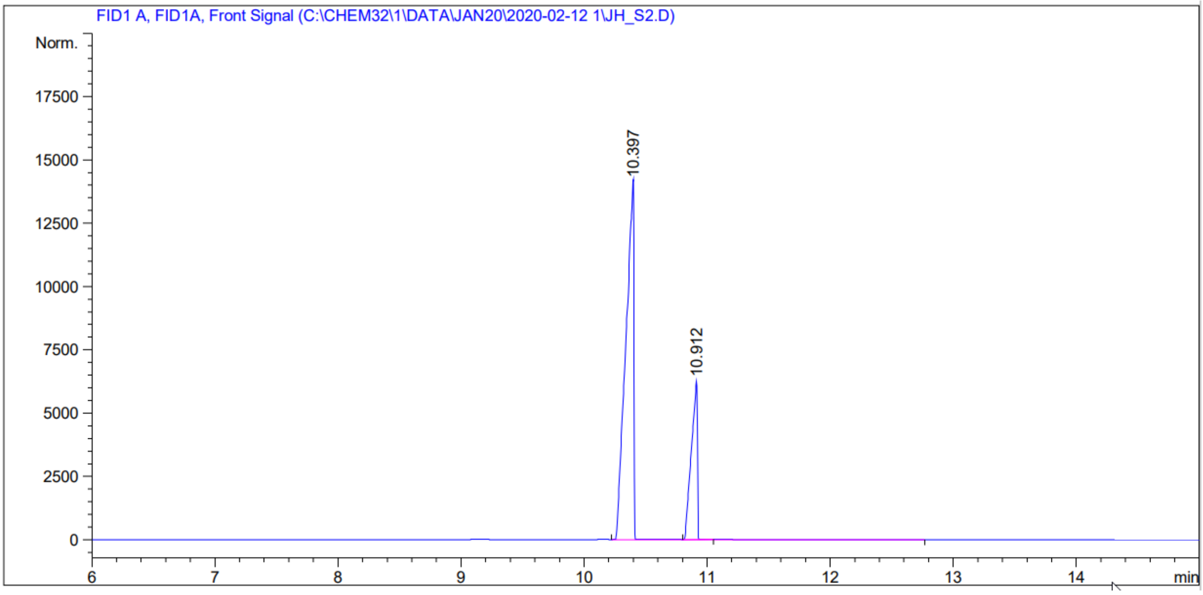

Retention Time	Sample	Peak Area
10.397	Dodecane	64068.3
10.912	2-Iodo-*m*-xylene **7**	21218.7

0.1 mmol

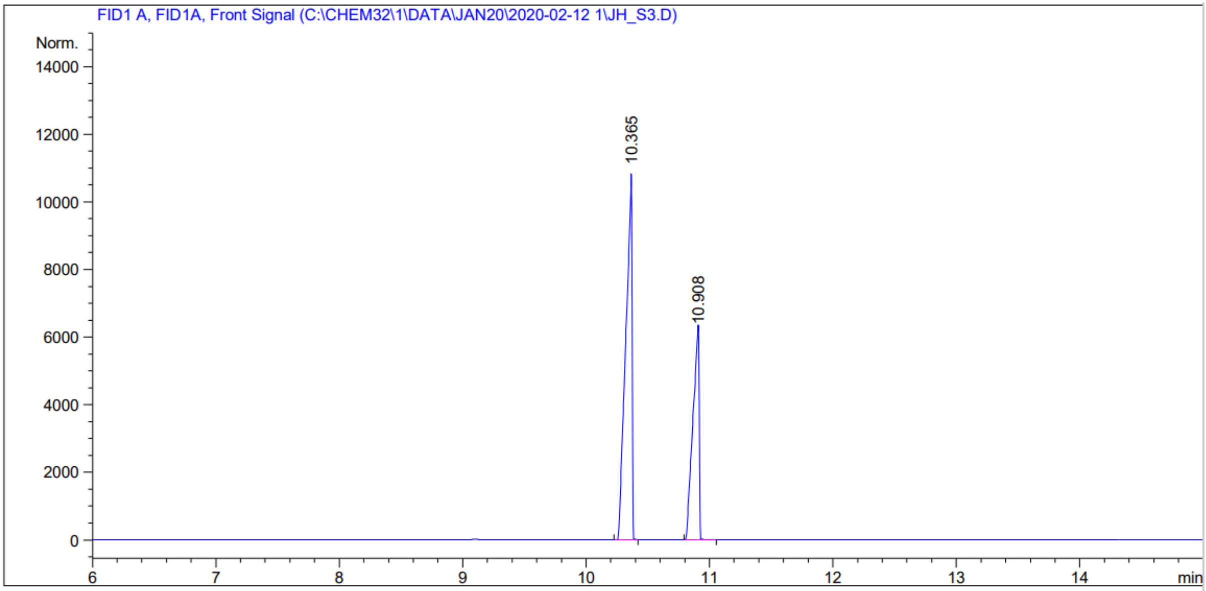

Retention Time	Sample	Peak Area
10.365	Dodecane	37932.2
10.908	2-Iodo-*m*-xylene **7**	22214.8

0.2 mmol

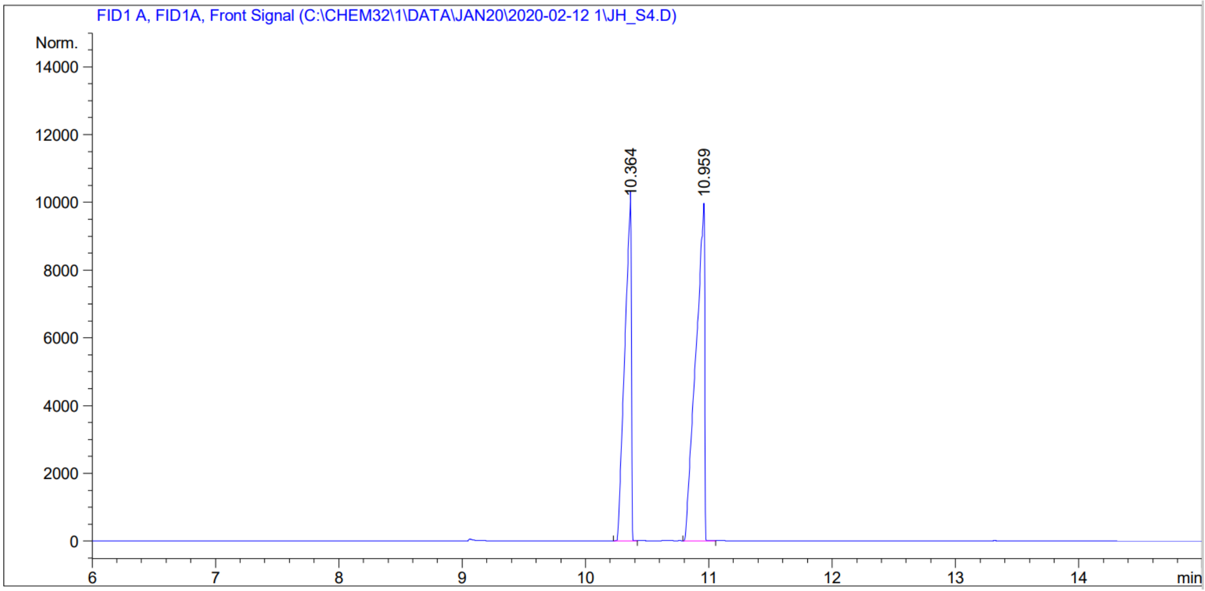

Retention Time	Sample	Peak Area
10.364	Dodecane	37309.1
10.959	2-Iodo-*m*-xylene **7**	49616.7

0.3 mmol

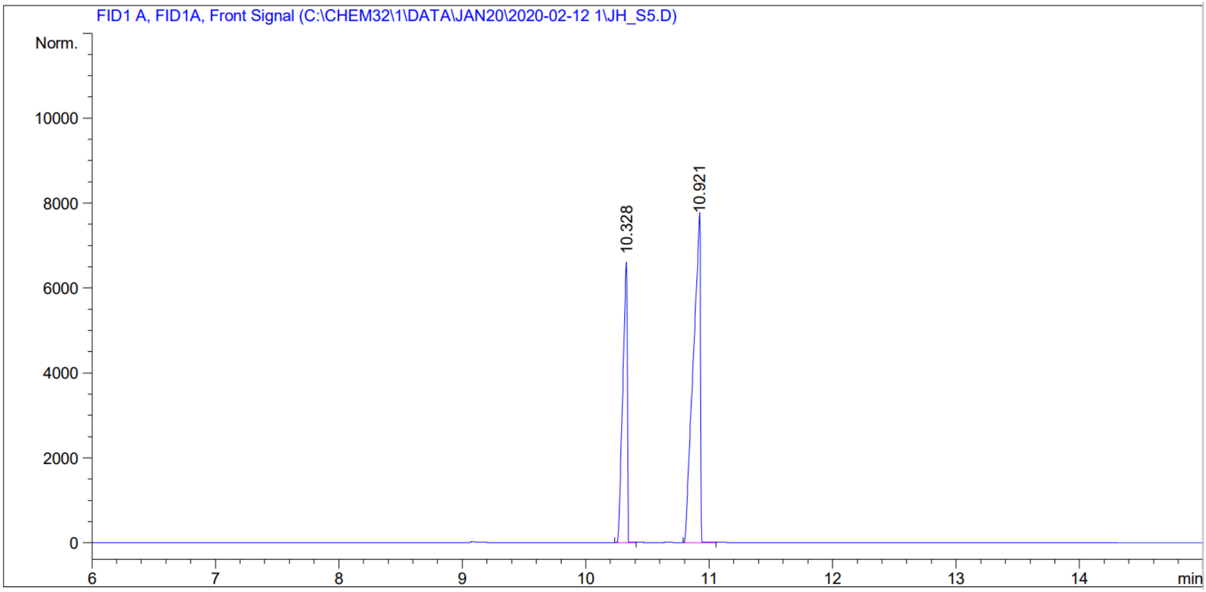

Retention Time	Sample	Peak Area
10.328	Dodecane	16580
10.921	2-Iodo-*m*-xylene **7**	31079.9

0.4 mmol

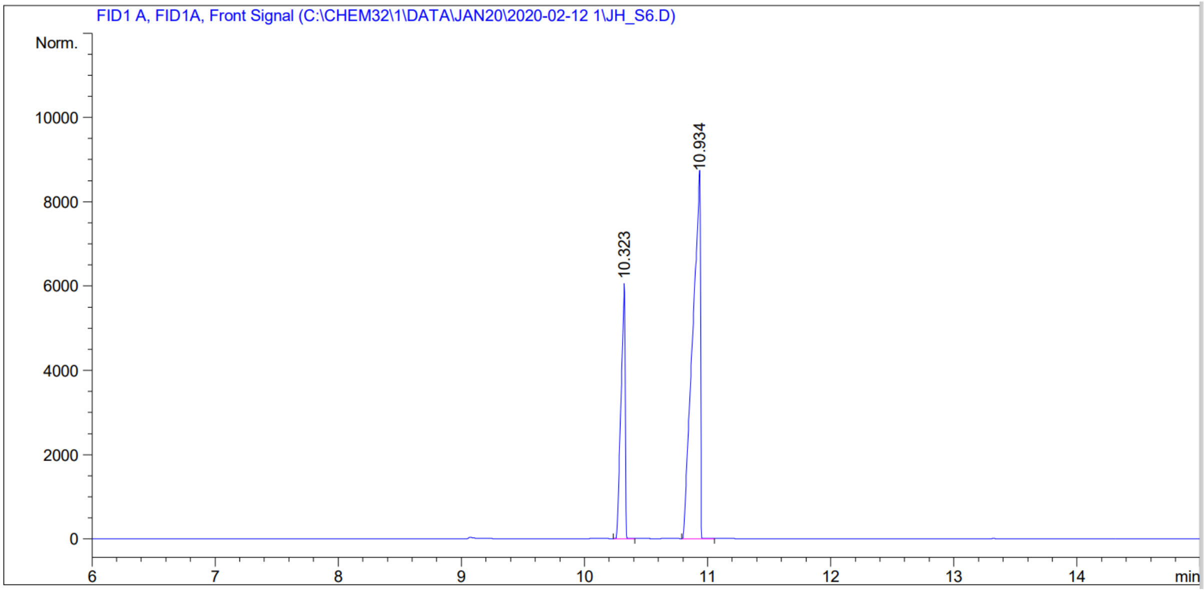

Retention Time	Sample	Peak Area
10.323	Dodecane	14086.6
10.934	2-Iodo-*m*-xylene	38264.4

For determination of yields:

Example used for **Reaction of 2-Iodo-*m*-xylene 7 with piperazinedione 16 and KO^{t}Bu**

For 2-Iodo-*m*-xylene:

- Area of 2-Iodo-*m*-xylene = **7196.2**
- Area of dodecane = **4045.8**
- Exact mass of dodecane added to vial = 16.7 mg = **0.098** mmol
- Area Ratio: 7196.2/4045.8 = **1.778**
- To determine mmol of sample: (Area ratio*mmol of dodecane)/gradient of the calibration curve = (1.778*0.098)/0.6535= **0.267**
- %yield = (mmol of sample/starting mmol of reaction)*100 = (0.267/0.7)*100 = **38.1%**

Biphenyl, 9

0.01 mmol

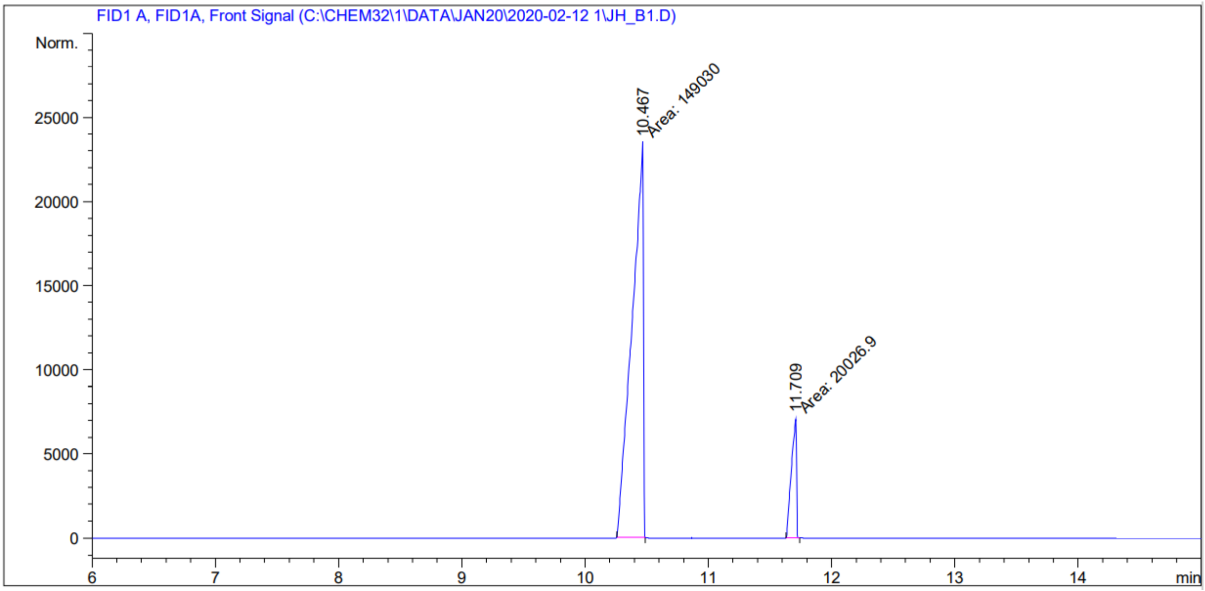

Retention Time	Sample	Peak Area
10.467	Dodecane	149030
11.709	Biphenyl **9**	20026.9

0.05 mmol

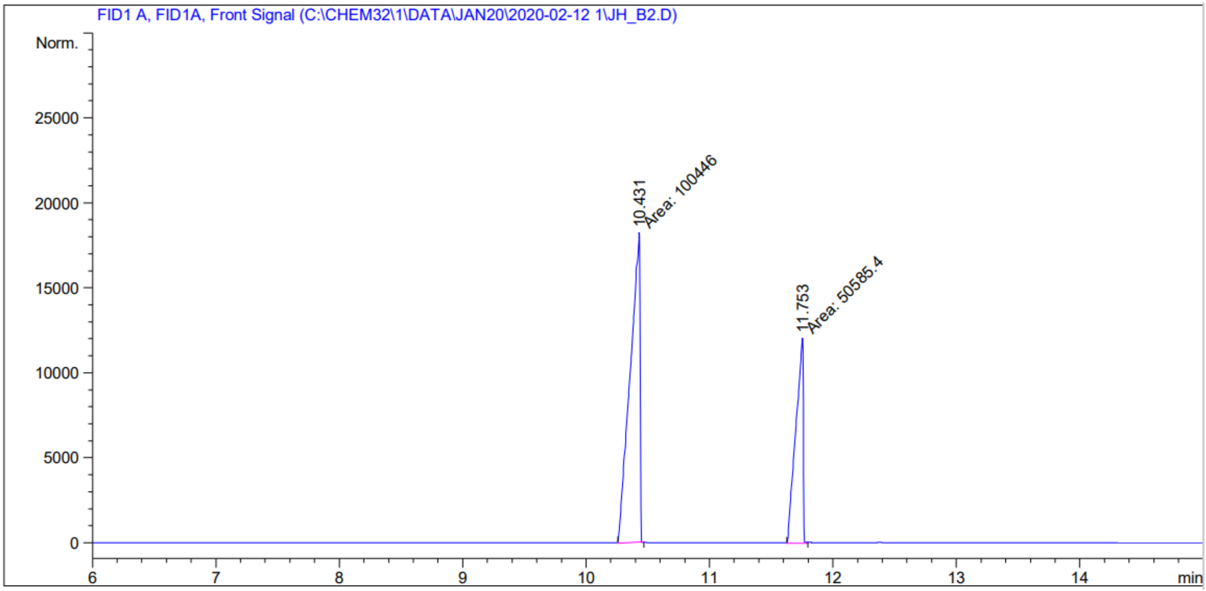

Retention Time	Sample	Peak Area
10.431	Dodecane	100446
11.753	Biphenyl **9**	50585.4

0.1 mmol

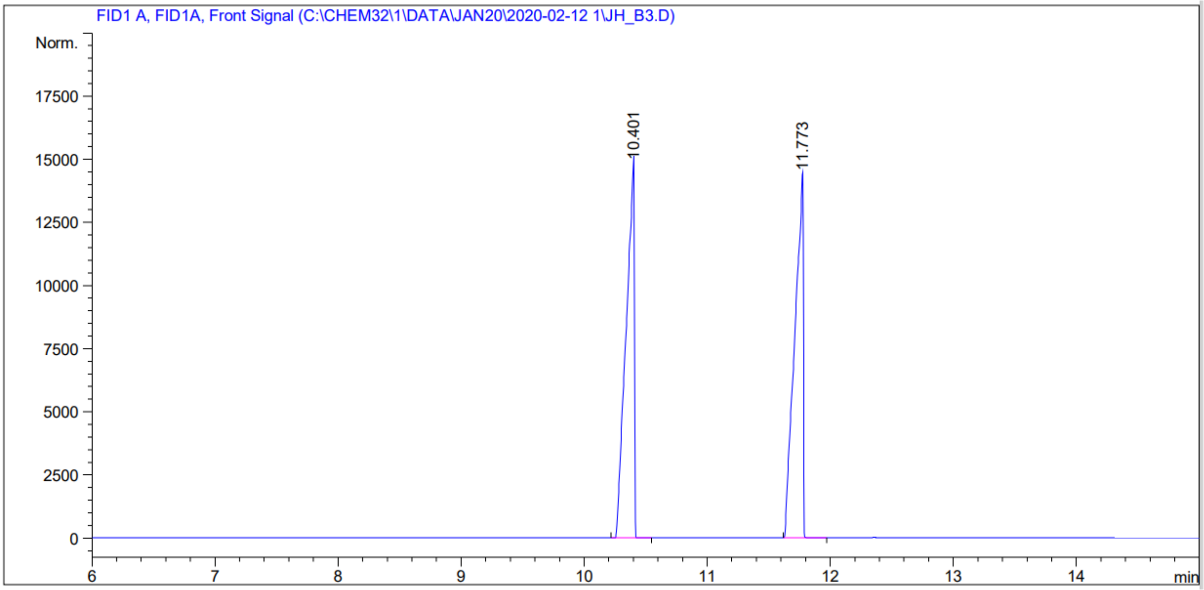

Retention Time	Sample	Peak Area
10.401	Dodecane	70144.2
11.773	Biphenyl **9**	69248.1

0.2 mmol

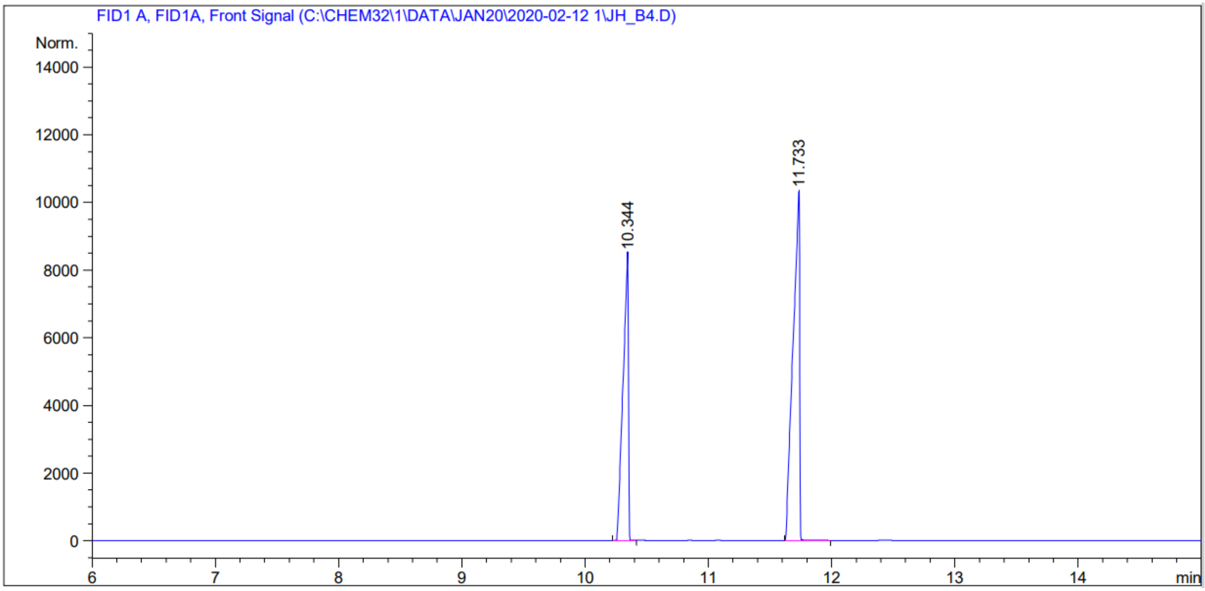

Retention Time	Sample	Peak Area
10.344	Dodecane	25473.5
11.773	Biphenyl **9**	38464.5

0.3 mmol

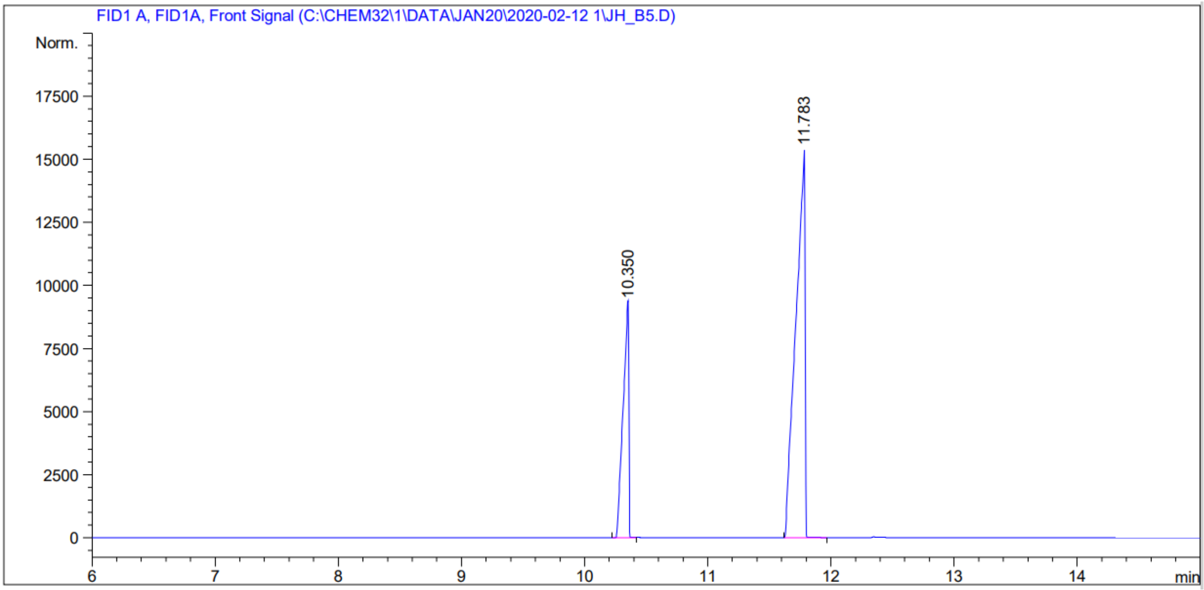

Retention Time	Sample	Peak Area
10.350	Dodecane	29547.5
11.783	Biphenyl **9**	79621.3

0.4 mmol

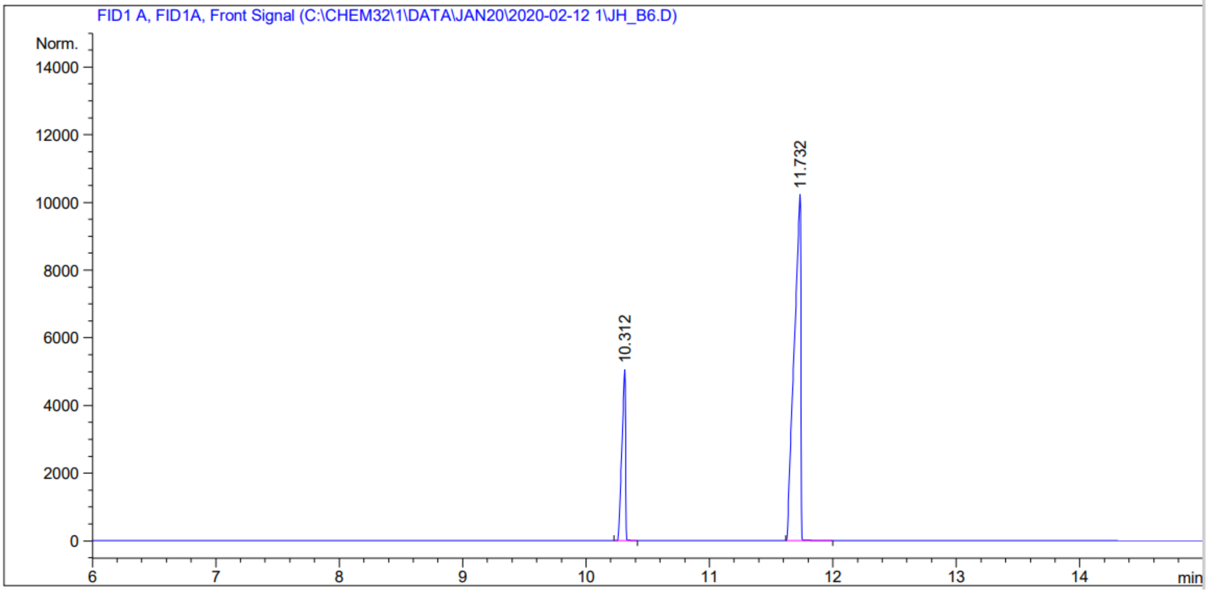

Retention Time	Sample	Peak Area
10.312	Dodecane	10280.9
11.732	Biphenyl 9	38048.0

For determination of yields:

Example used for **Reaction of 2-Iodo-*m*-xylene 7 with piperazinedione 16 and KO^{t}Bu**

For Biphenyl:

- Area of Biphenyl = **3934.5**
- Area of dodecane = **4045.8**
- Exact mass of dodecane added to vial = 16.7 mg = **0.098** mmol
- Area Ratio: 3934.5/4045.8 = **0.972**
- To determine mmol of sample: (Area ratio*mmol of dodecane)/gradient of the calibration curve = (0.972*0.098)/0.975= **0.098**
- %yield = (mmol of sample/starting mmol of reaction)*100 = (0.098/0.7)*100 = **14.0%**

2,6-Dimethylbiphenyl 8

0.01 mmol

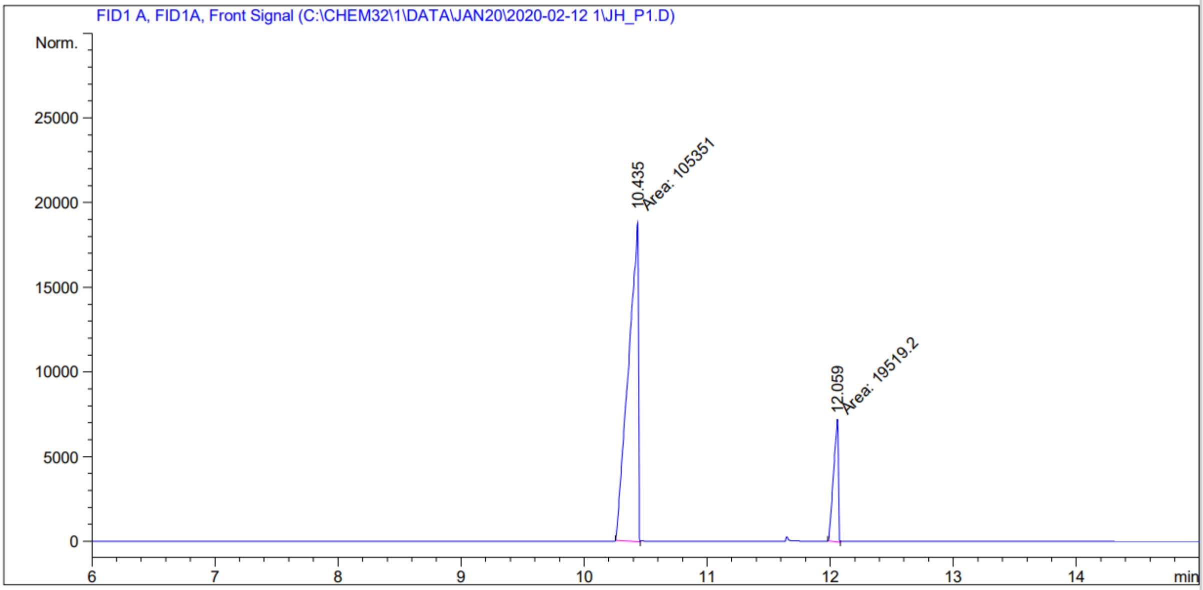

Retention Time	Sample	Peak Area
10.435	Dodecane	105351
12.059	2,6-Dimethylbiphenyl, **8**	19519.2

0.06 mmol

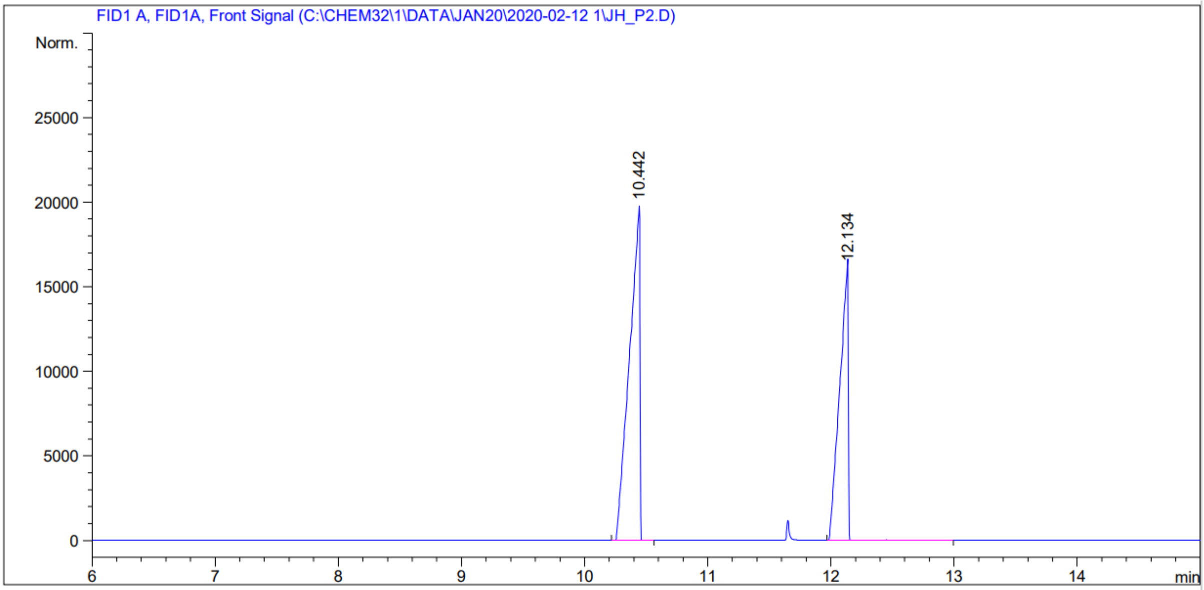

Retention Time	Sample	Peak Area
10.442	Dodecane	115829.0
12.134	2,6-Dimethylbiphenyl, **8**	78888.5

0.1 mmol

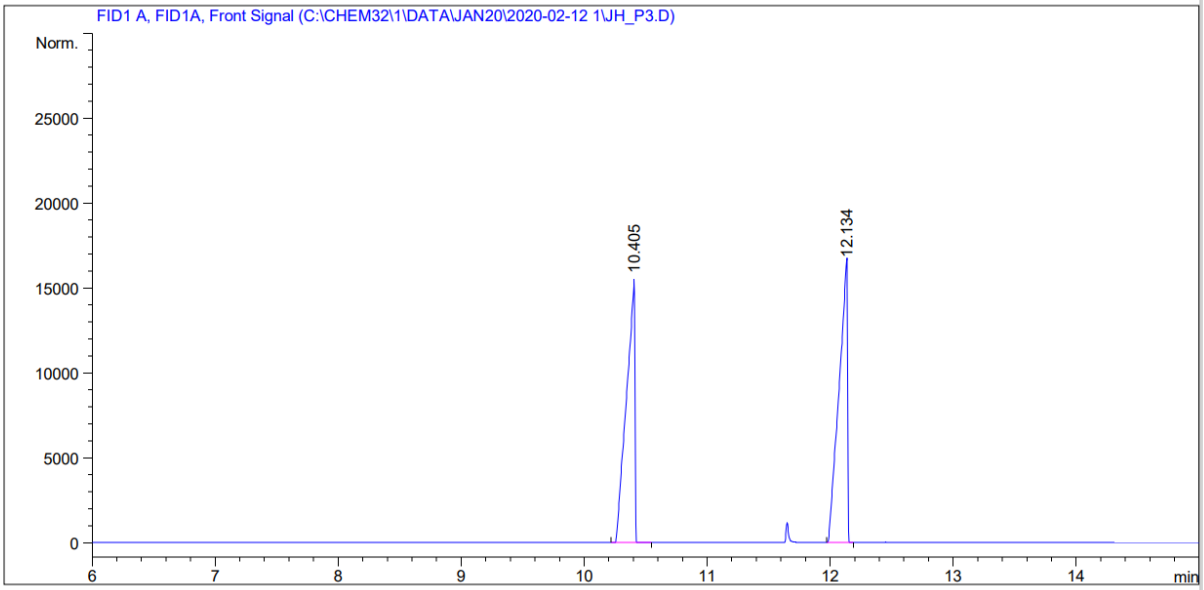

Retention Time	Sample	Peak Area
10.405	Dodecane	74090.1
12.134	2,6-Dimethylbiphenyl, **8**	80332.6

0.2 mmol

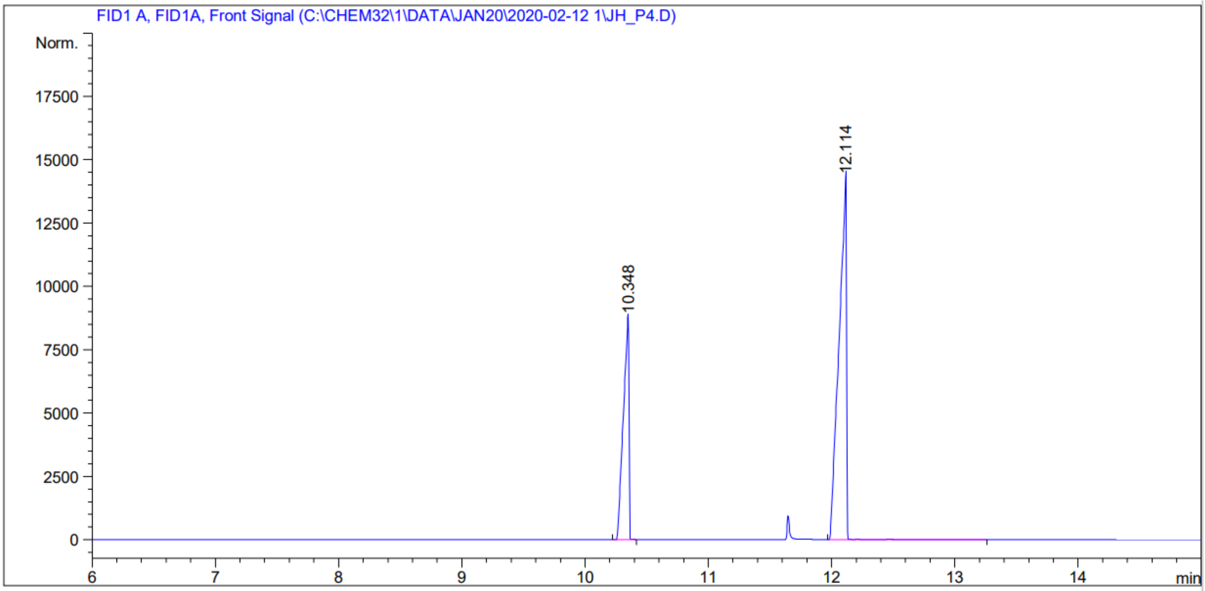

Retention Time	Sample	Peak Area
10.348	Dodecane	27600.9
12.114	2,6-Dimethylbiphenyl, **8**	60205.5

0.3 mmol

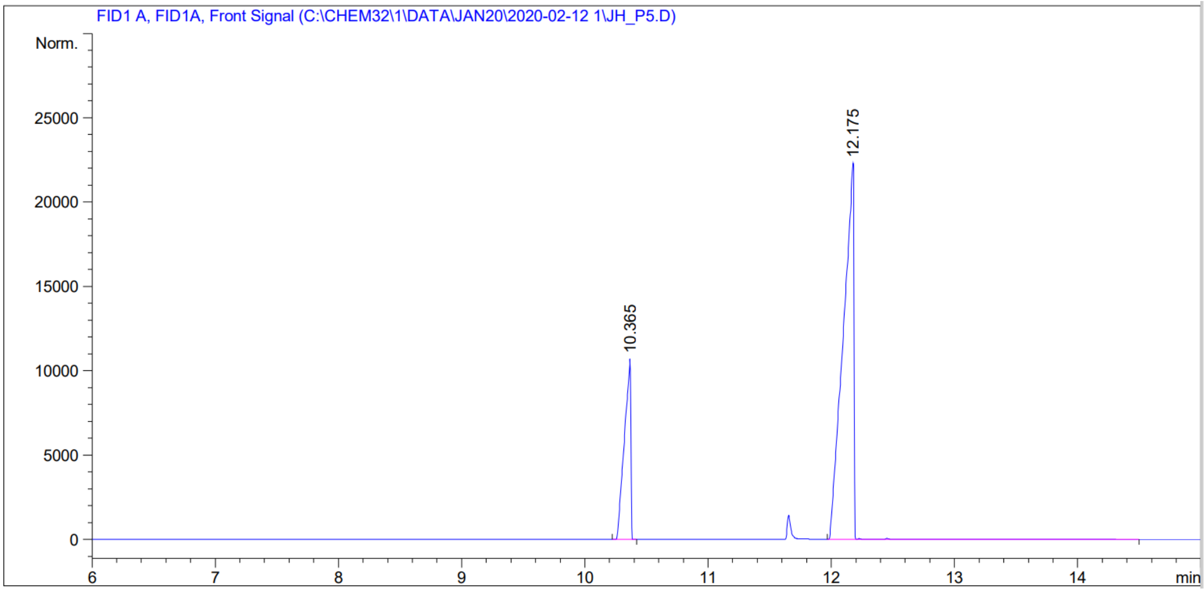

Retention Time	Sample	Peak Area
10.365	Dodecane	38696.9
12.175	2,6-Dimethylbiphenyl, **8**	133795.0

0.4 mmol

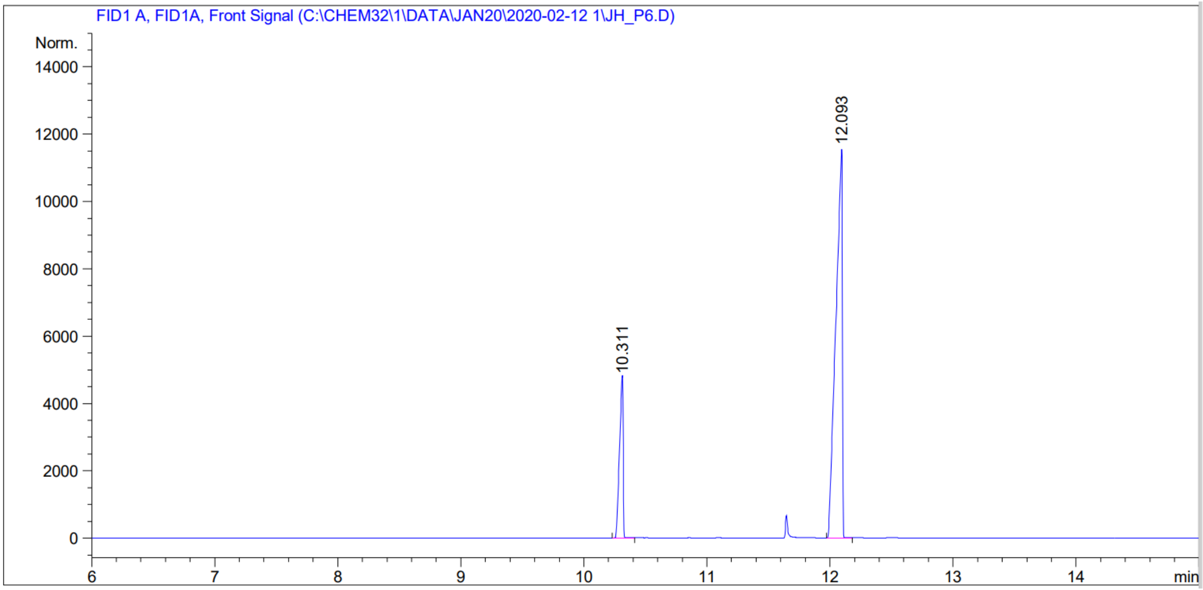

Retention Time	Sample	Peak Area
10.311	Dodecane	9779.35352
12.093	2,6-Dimethylbiphenyl, **8**	42358.7

For determination of yields:

Example used for **Reaction of 2-Iodo-*m*-xylene 7 with Piperazinedione 16 and KO^{t}Bu**

For 2,6-Dimethylbiphenyl:

- Area of 2,6-Dimethylbiphenyl = **820.8**
- Area of dodecane = **4045.8**
- Exact mass of dodecane added to vial = 16.7 mg = **0.098** mmol
- Area Ratio: 820.8/4045.8 = **0.20**
- To determine mmol of sample: (Area ratio*mmol of dodecane)/gradient of the calibration curve = (0.20*0.098)/1.1654= **0.017**
- %yield = (mmol of sample/starting mmol of reaction)*100 = (0.017/0.7)*100 = **2.4%**

***m*-Xylene, `10**

0.01 mmol

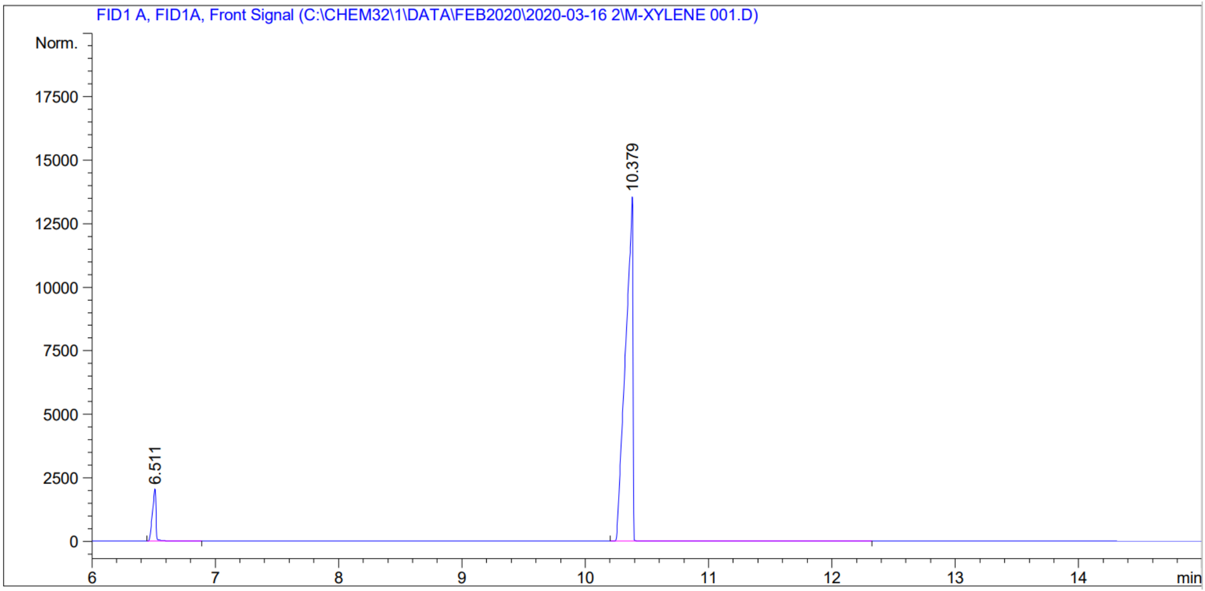

Retention Time	Sample	Peak Area
6.511	*m*-Xylene, **10**	4179.44189
10.379	Dodecane	56734.4

0.3 mmol

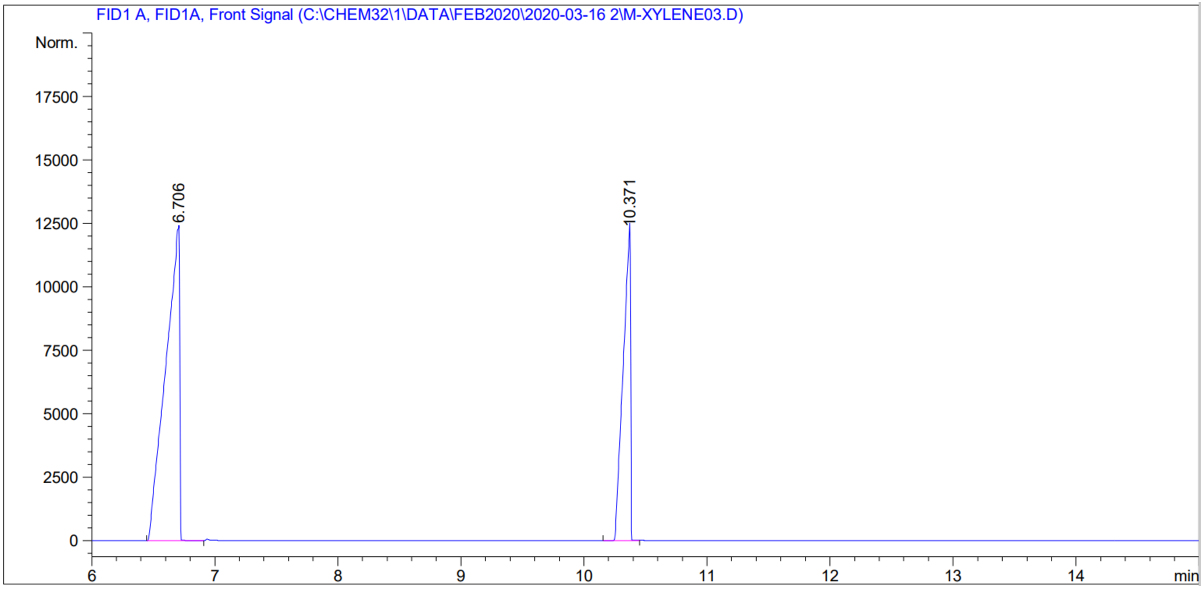

Retention Time	Sample	Peak Area
6.706	*m*-Xylene	97344.0
10.371	Dodecane	51171.6

0.5 mmol

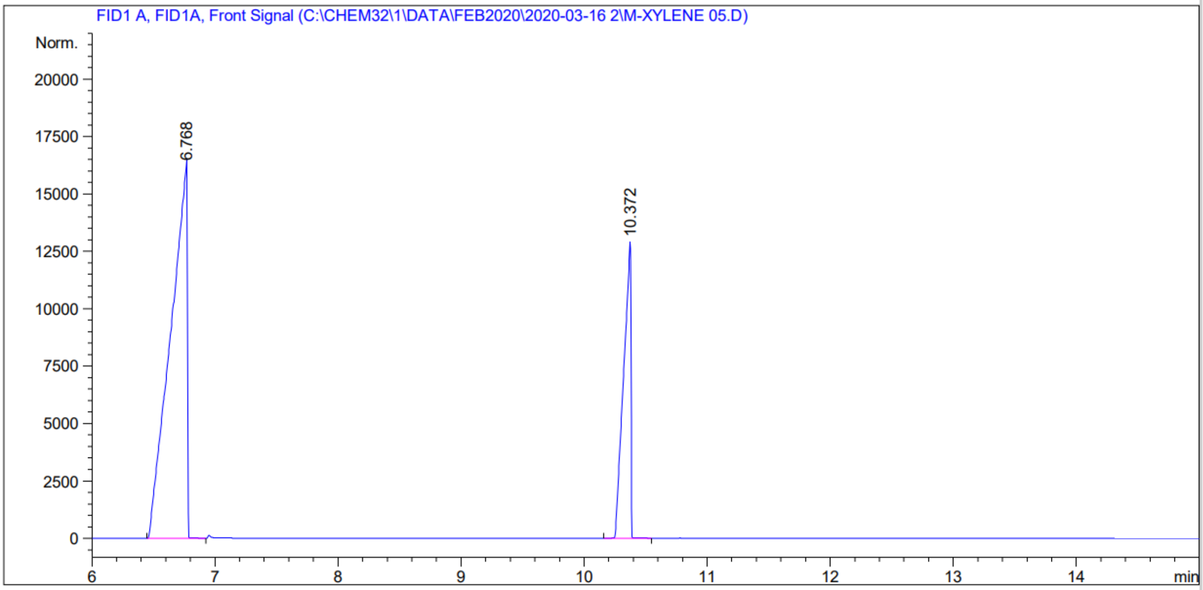

Retention Time	Sample	Peak Area
6.768	*m*-Xylene	153825.0
10.372	Dodecane	52679.4

0.7 mmol

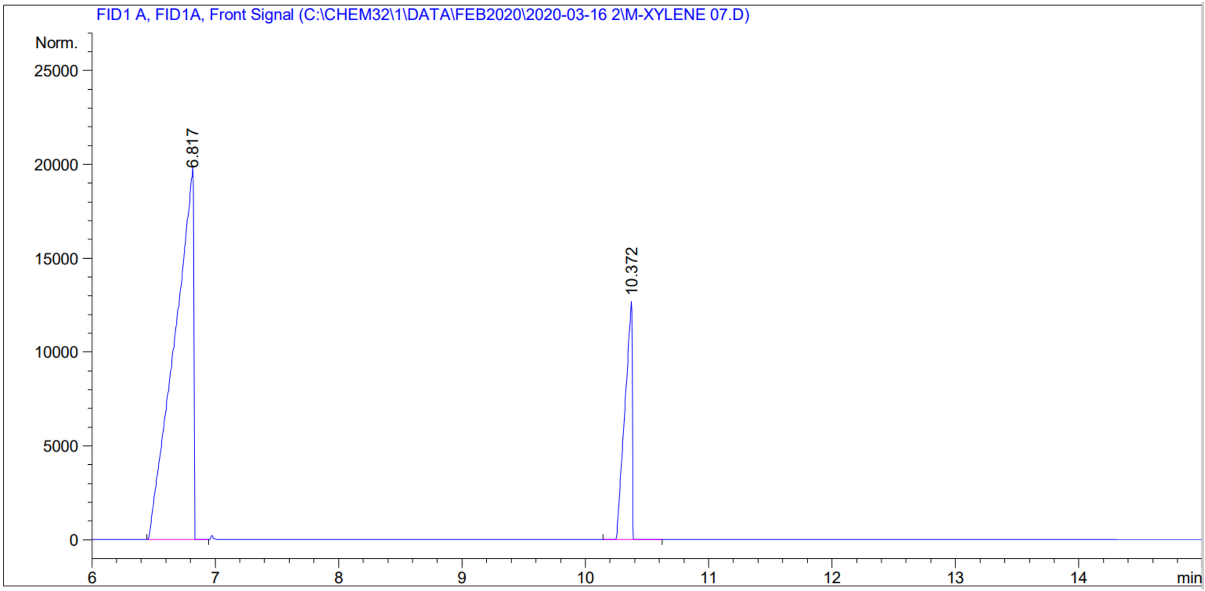

Retention Time	Sample	Peak Area
6.817	*m*-Xylene	211125
10.372	Dodecane	52021.0

For determination of yields:

Example used for **Reaction of 2-Iodo-*m*-xylene 7 with piperazinedione 16 and KO^{t}Bu**

For *m*-Xylene:

- Area of *m*-Xylene = **9449.6**
- Area of dodecane = **4045.8**
- Exact mass of dodecane added to vial = 16.7 mg = **0.098** mmol
- Area Ratio: 9449.6/4045.8 = **2.34**
- To determine mmol of sample: (Area ratio*mmol of dodecane)/gradient of the calibration curve = (2.34*0.098)/0.6853= **0.334**
- %yield = (mmol of sample/starting mmol of reaction)*100 = (0.334/0.7)*100 = **47.7%**

<u>2-Iodo-m-xylene – Second Set of GCFID Data</u>

2-Iodo-*m*-xylene, 7

0.02 mmol

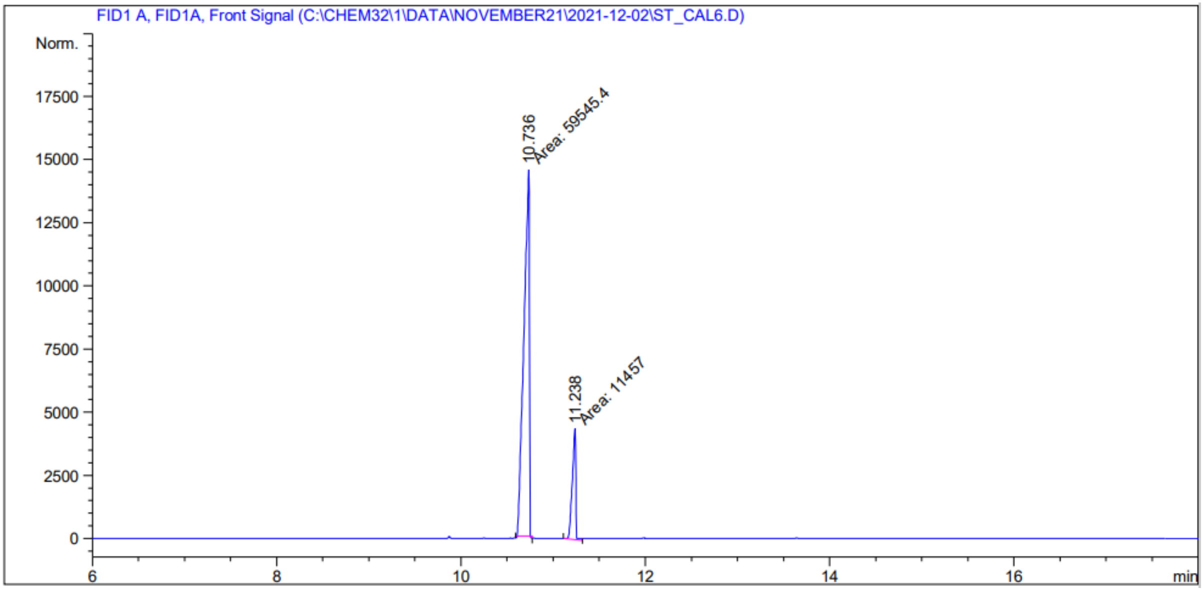

Retention Time	Sample	Peak Area
10.736	Dodecane	59545.4
11.238	2-Iodo-*m*-xylene, **7**	11457.0

0.04 mmol

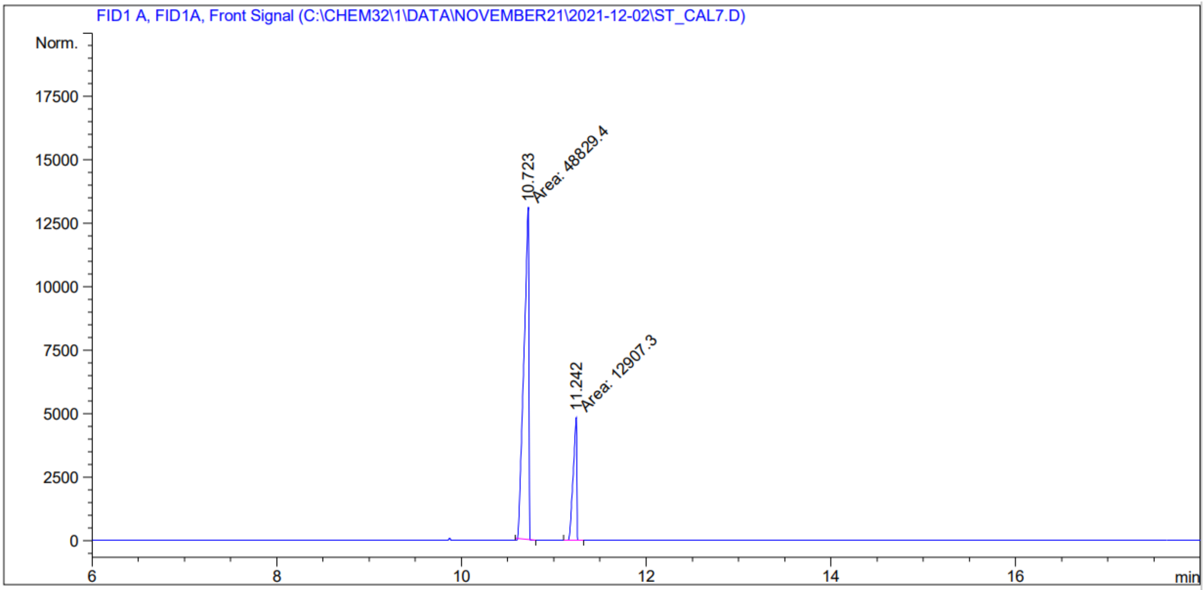

Retention Time	Sample	Peak Area
10.723	Dodecane	48829.4
11.242	2-Iodo-*m*-xylene, **7**	12907.3

0.1 mmol

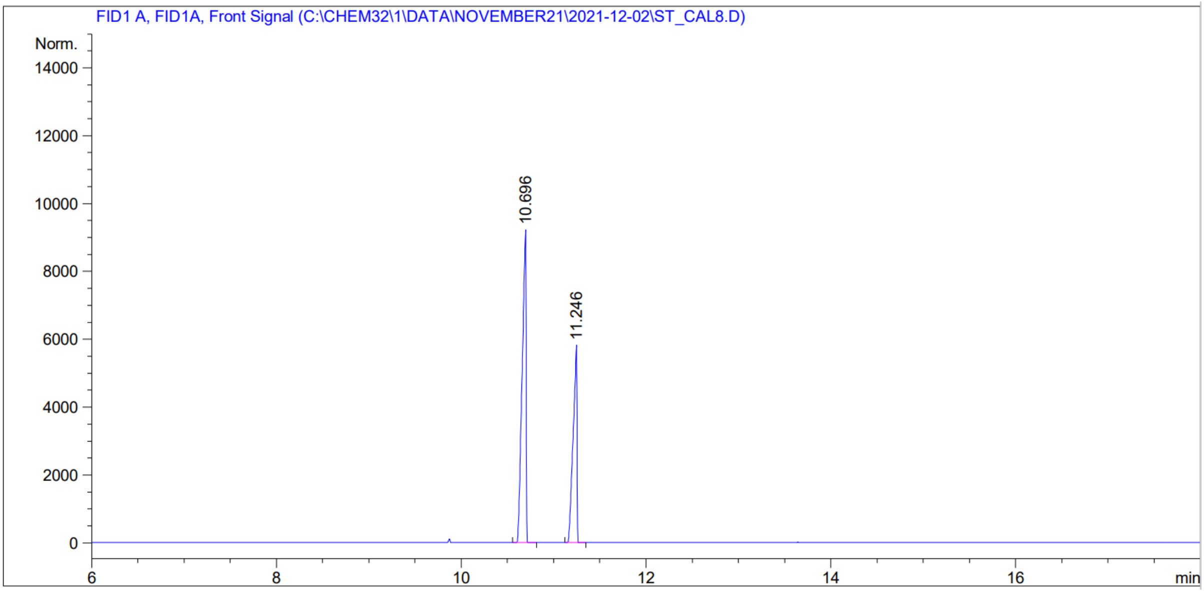

Retention Time	Sample	Peak Area
10.696	Dodecane	27416.6
11.246	2-Iodo-*m*-xylene, **7**	16799.3

0.2 mmol

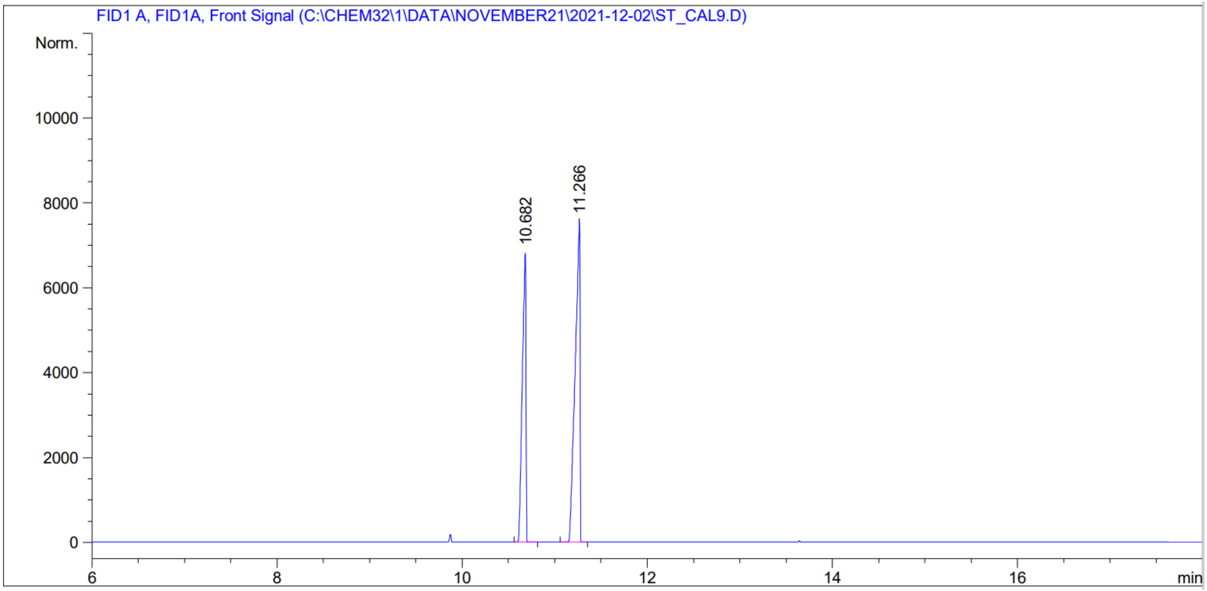

Retention Time	Sample	Peak Area
10.682	Dodecane	18276.5
11.266	2-Iodo-*m*-xylene **7**	27341.3

0.4 mmol

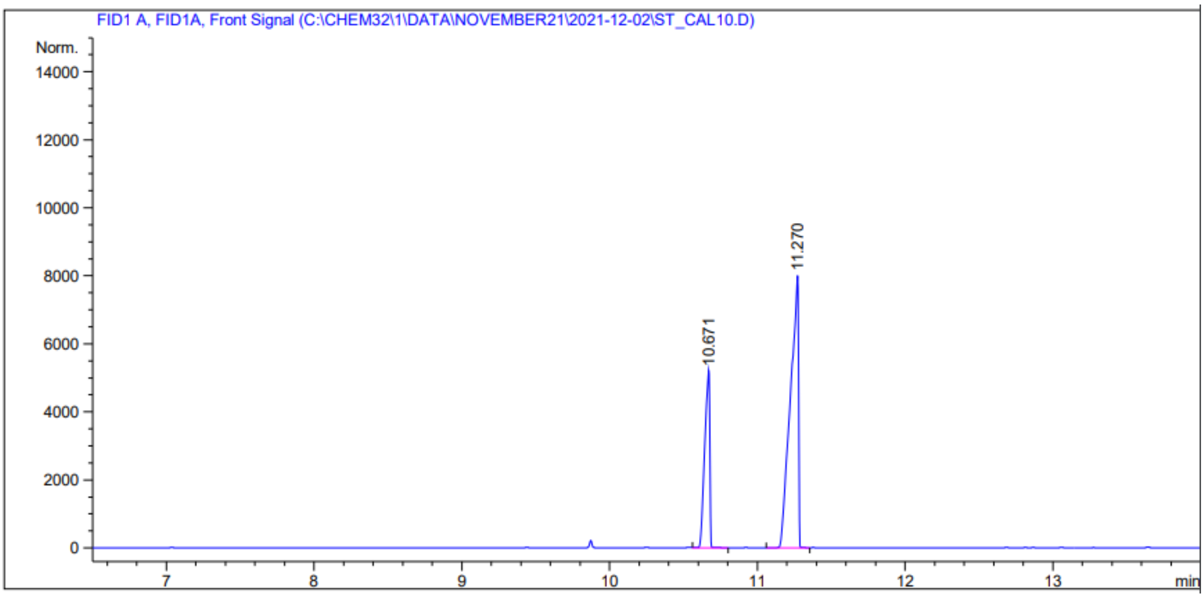

Retention Time	Sample	Peak Area
10.671	Dodecane	12433.8
11.270	2-Iodo-*m*-xylene **7**	29666.8

0.6 mmol

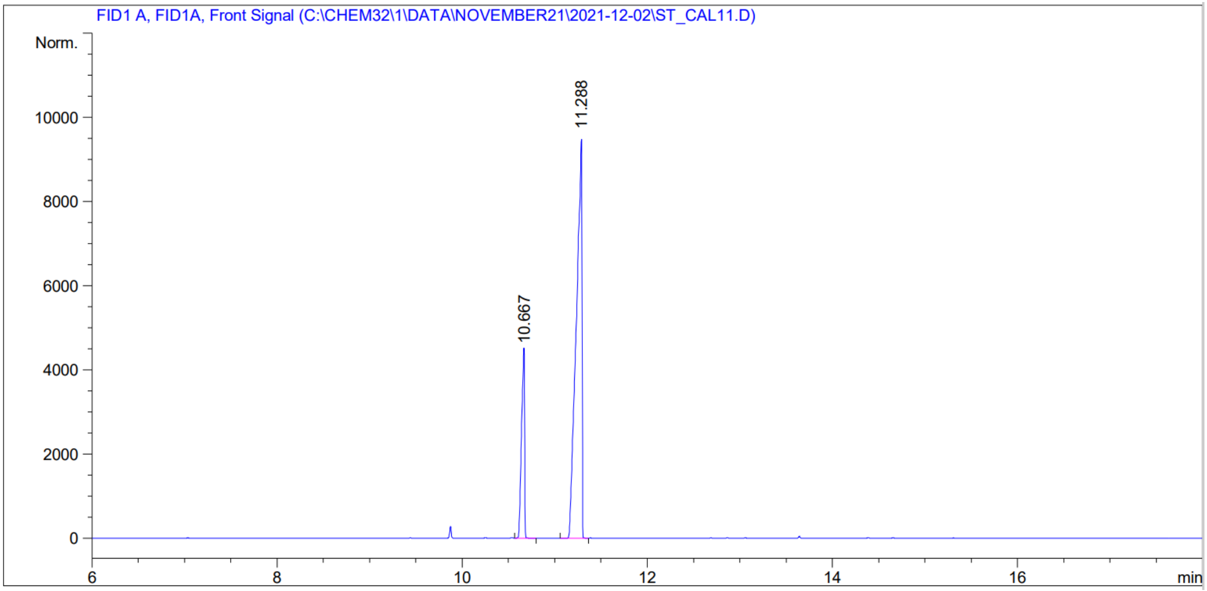

Retention Time	Sample	Peak Area
10.667	Dodecane	10198.2
11.288	2-Iodo-*m*-xylene, **7**	40482.1

Biphenyl

0.01 mmol

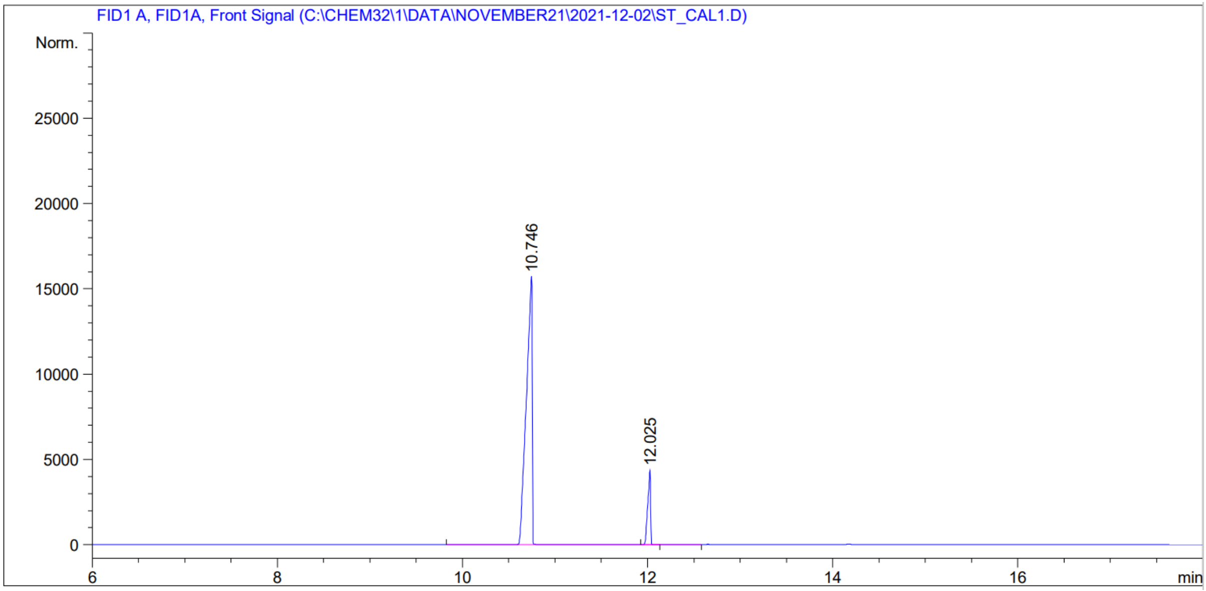

Retention Time	Sample	Peak Area
10.746	Dodecane	68957.4
12.025	Biphenyl, **9**	9428.4

0.04 mmol

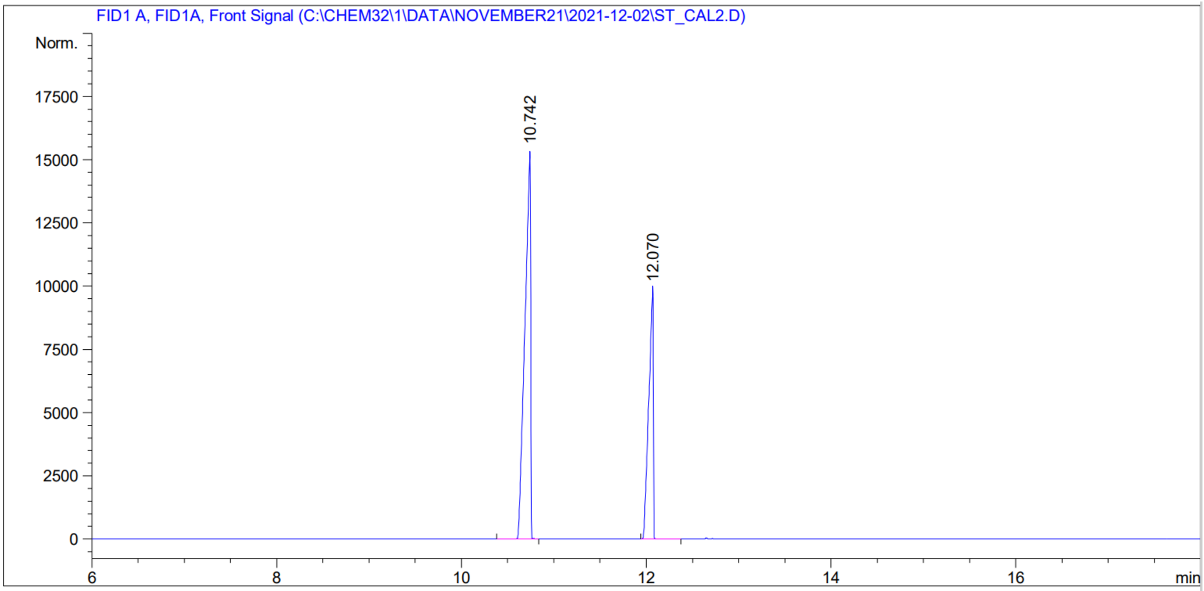

Retention Time	Sample	Peak Area
10.742	Dodecane	65096.8
12.070	Biphenyl, **9**	33761.9

0.1 mmol

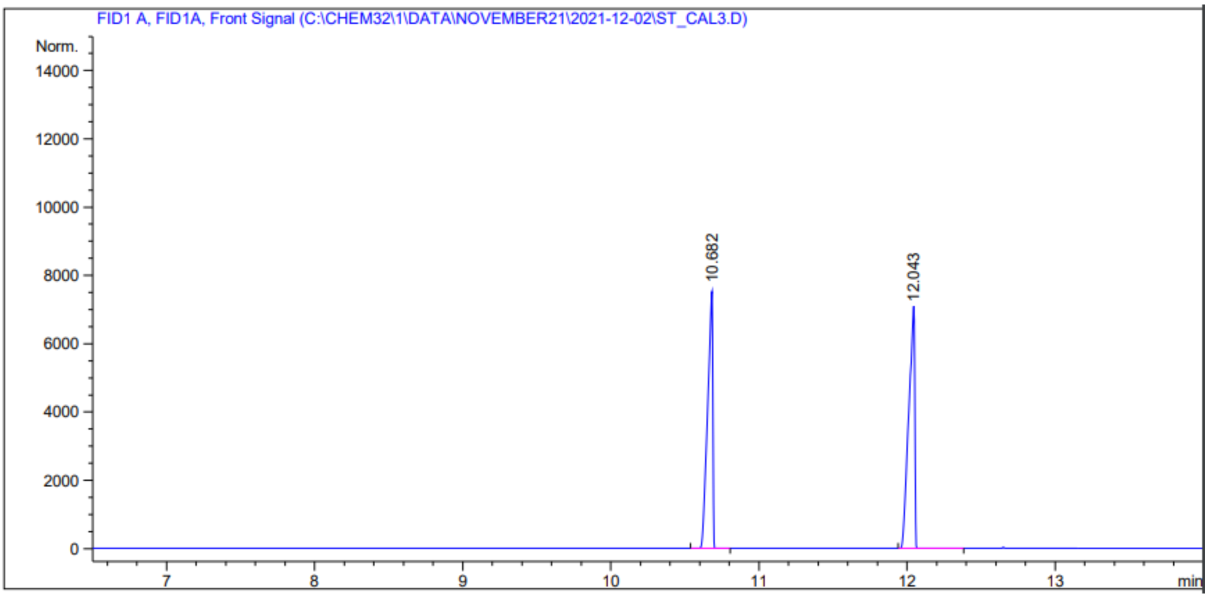

Retention Time	Sample	Peak Area
10.682	Dodecane	18326.8
12.043	Biphenyl, **9**	19101.4

0.3 mmol

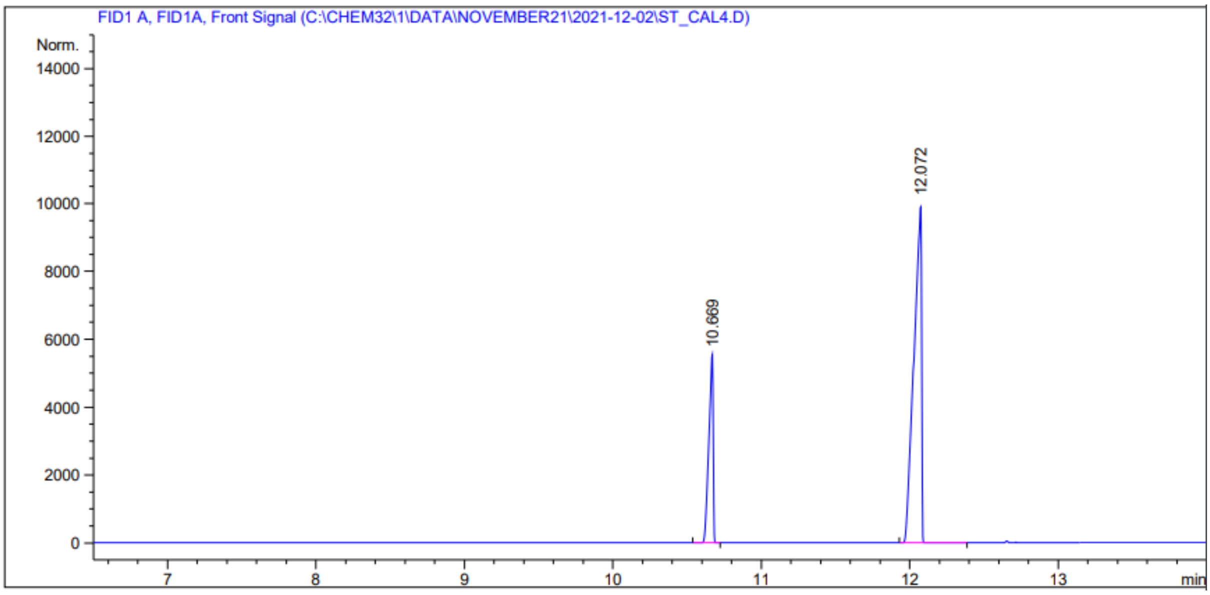

Retention Time	Sample	Peak Area
10.669	Dodecane	11200.1
12.072	Biphenyl, **9**	34515.8

0.7 mmol

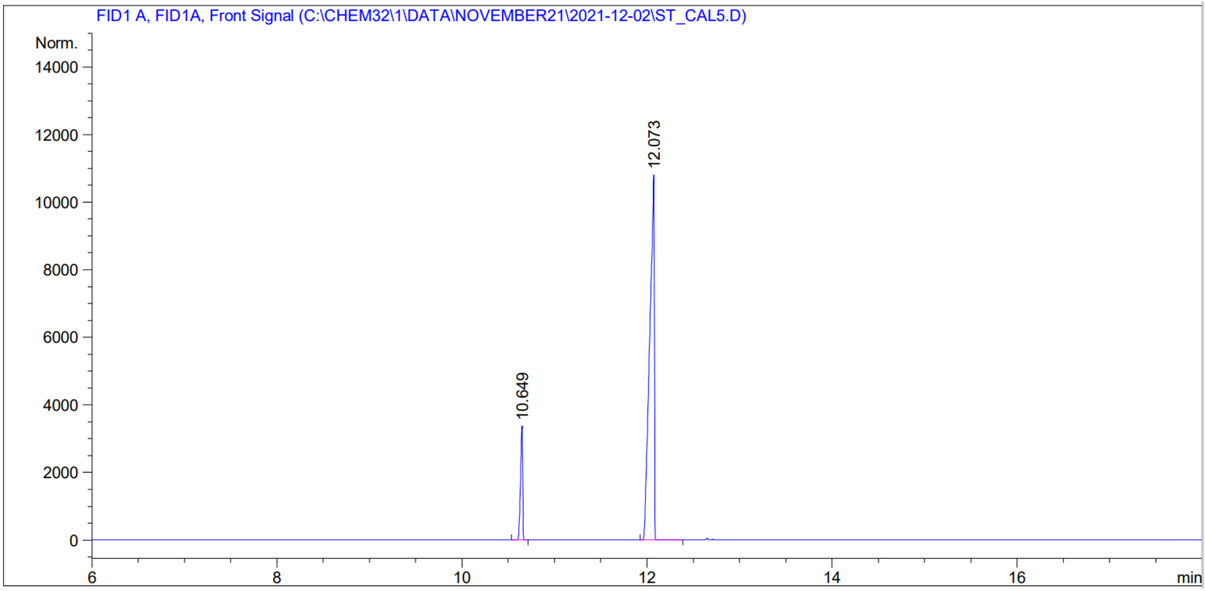

Retention Time	Sample	Peak Area
10.649	Dodecane	5400.0
12.073	Biphenyl, **9**	37752.0

2,6-Dimethylbiphenyl

0.01 mmol

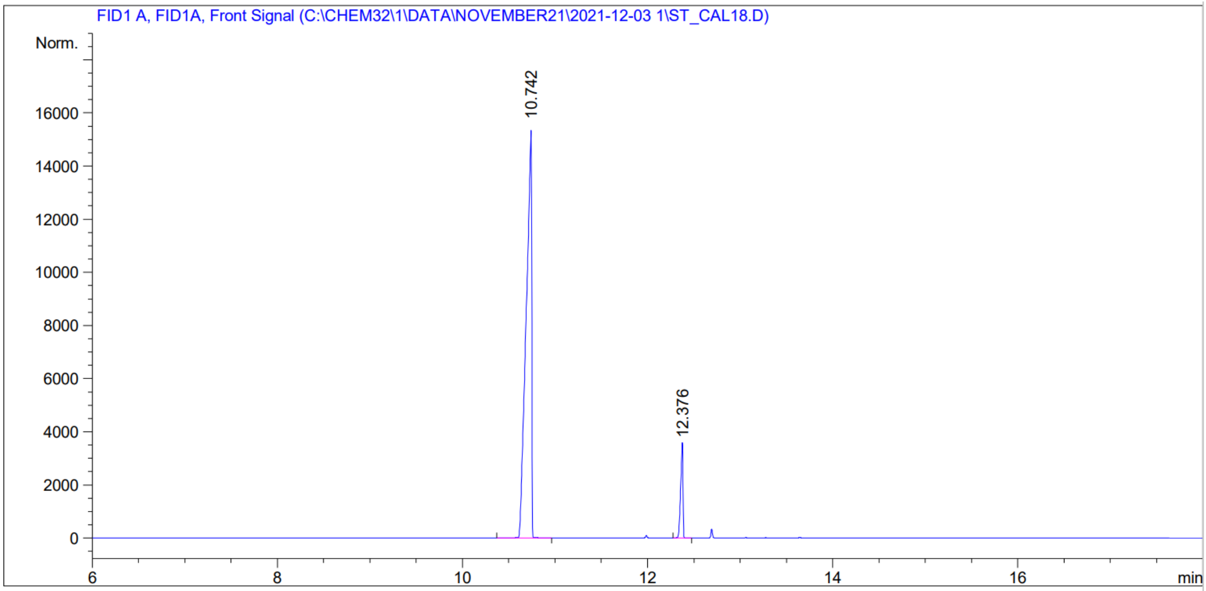

Retention Time	Sample	Peak Area
10.742	Dodecane	62881.5
12.376	2,6-Dimethylbiphenyl, **8**	6115.4

0.04 mmol

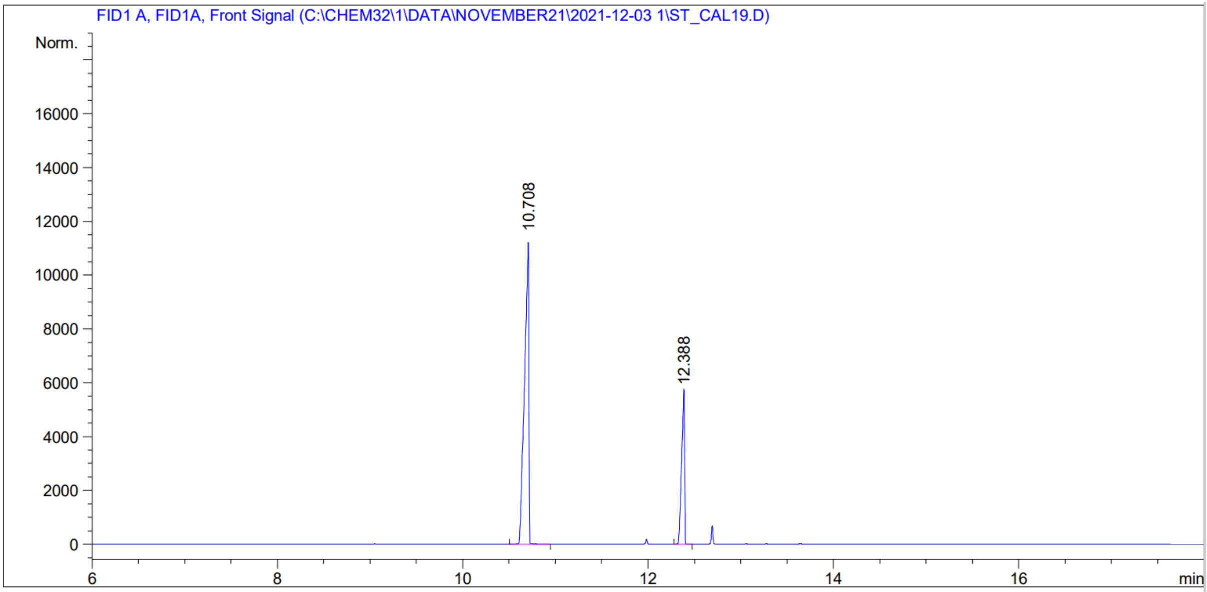

Retention Time	Sample	Peak Area
10.708	Dodecane	36554.7
12.388	2,6-Dimethylbiphenyl, **8**	12765.3

0.08 mmol

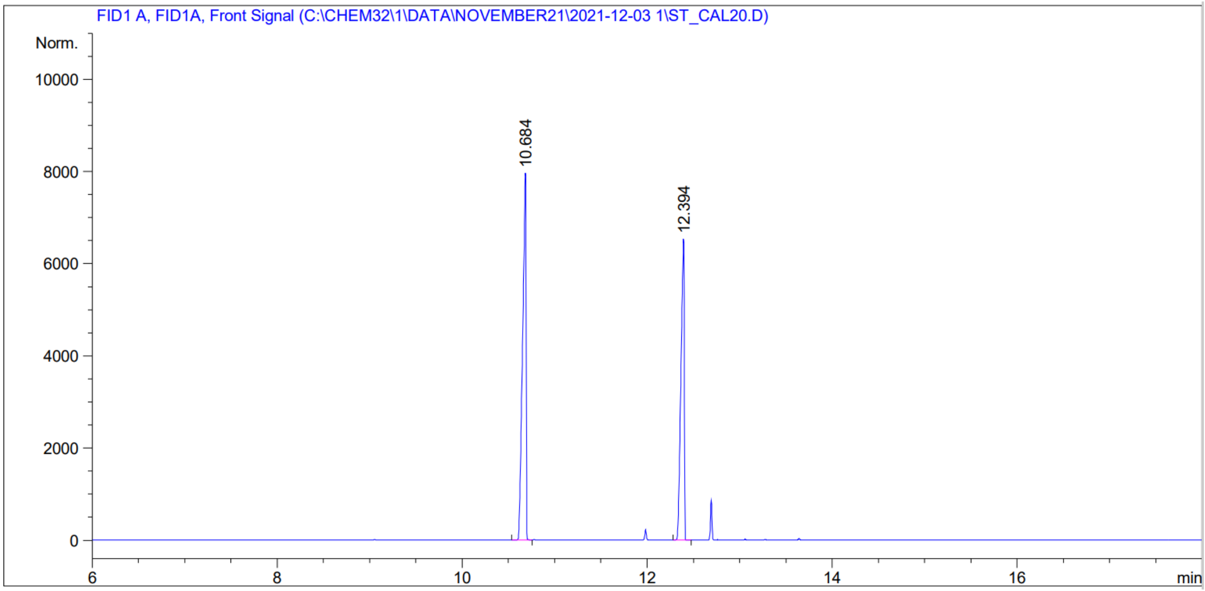

Retention Time	Sample	Peak Area
10.684	Dodecane	20688.2
12.394	2,6-Dimethylbiphenyl, **8**	16138

0.2 mmol

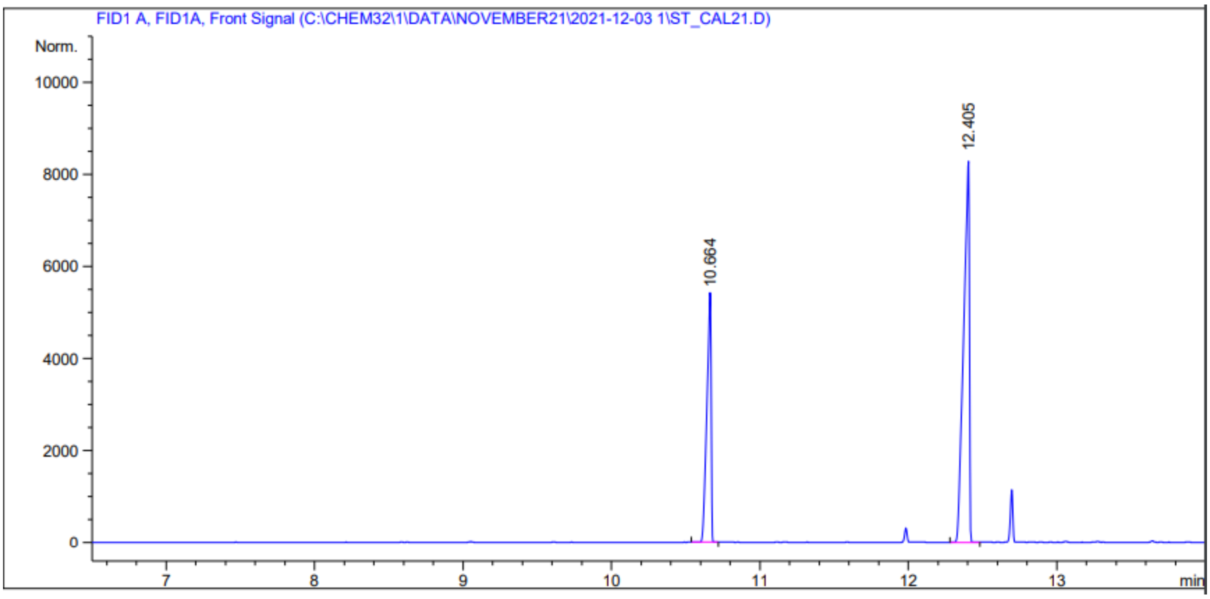

Retention Time	Sample	Peak Area
10.664	Dodecane	10923.1
12.405	2,6-Dimethylbiphenyl, **8**	22237.2

0.5 mmol

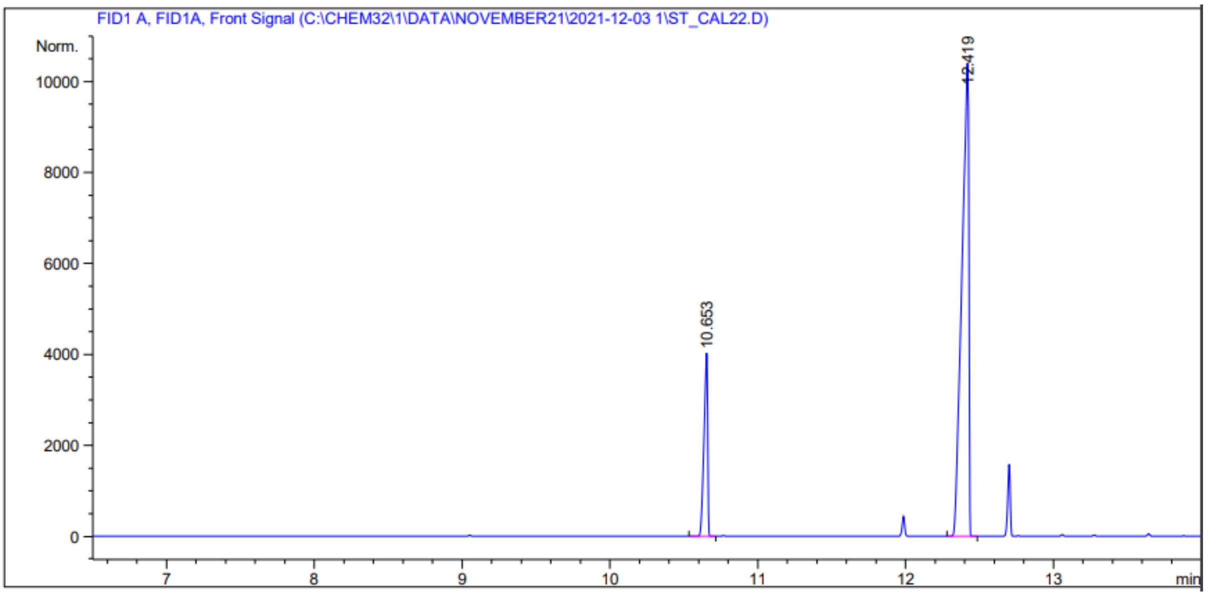

Retention Time	Sample	Peak Area
10.653	Dodecane	6931.01709
12.419	2,6-Dimethylbiphenyl, **8**	32072.9

***m*-Xylene**

0.02 mmol

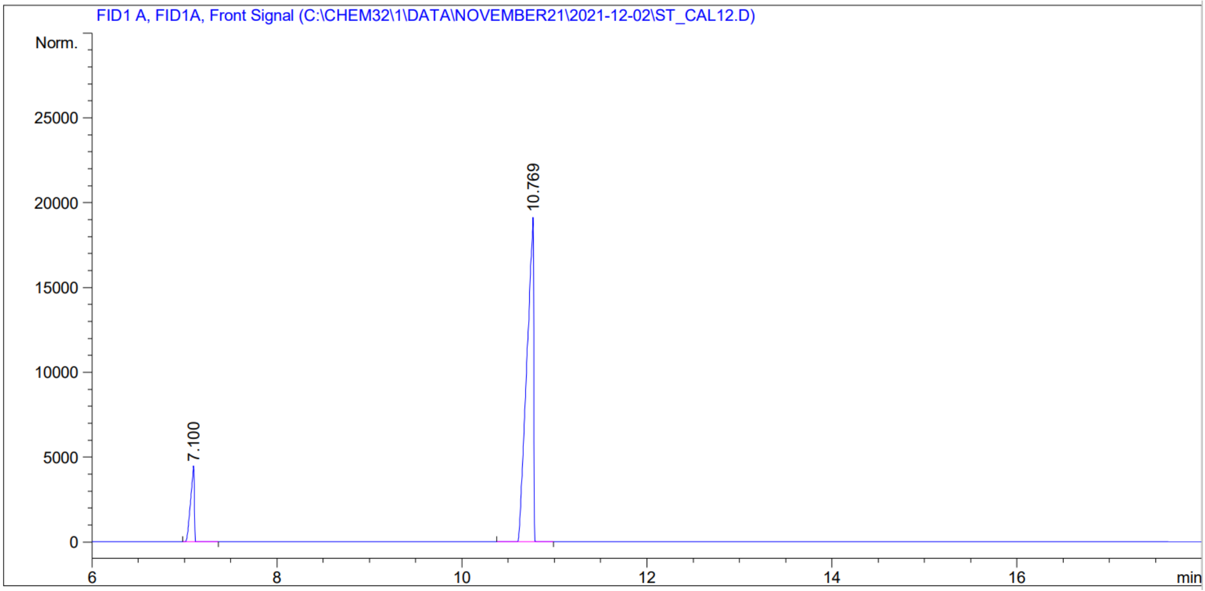

Retention Time	Sample	Peak Area
7.100	*m*-Xylene, **10**	12578.9
10.769	Dodecane	98487.2

0.08 mmol

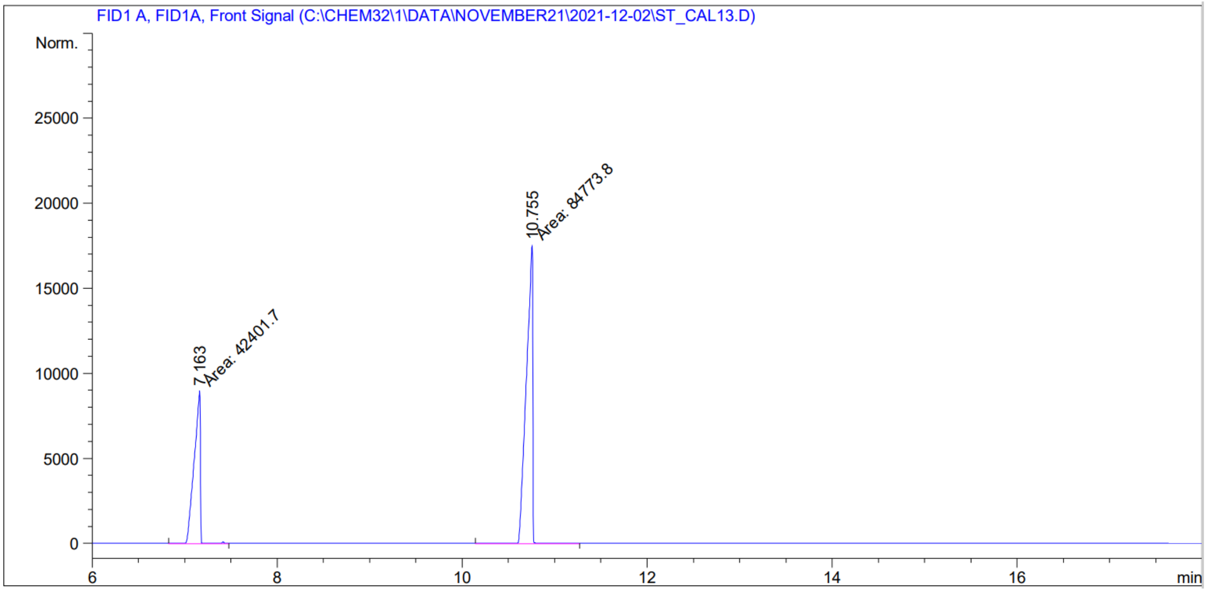

Retention Time	Sample	Peak Area
7.163	*m*-Xylene, **10**	42401.7
10.755	Dodecane	84773.8

0.1 mmol

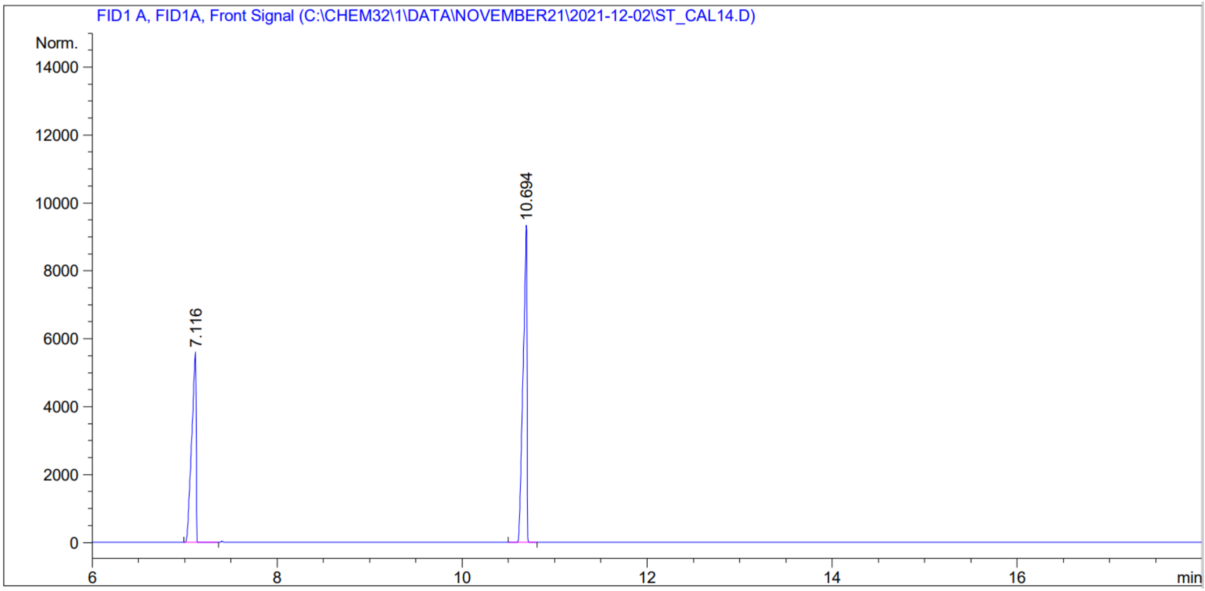

Retention Time	Sample	Peak Area
7.116	*m*-Xylene, **10**	18433.6
10.694	Dodecane	26890.0

0.3 mmol

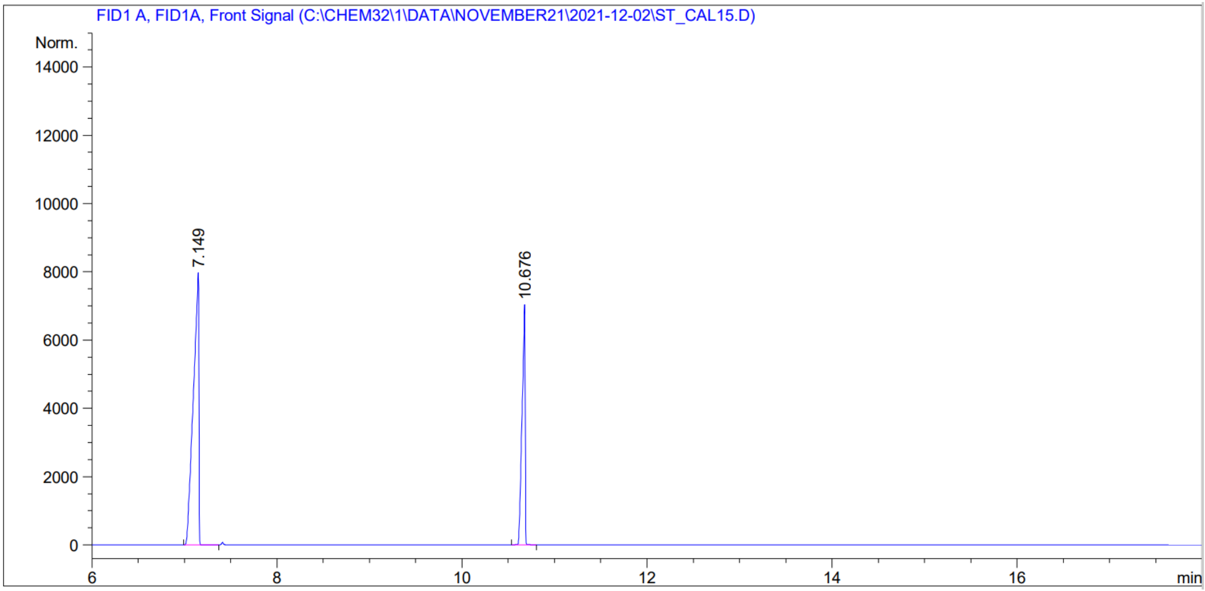

Retention Time	Sample	Peak Area
7.149	*m*-Xylene, **10**	32922.7
10.676	Dodecane	16190.1

0.8 mmol

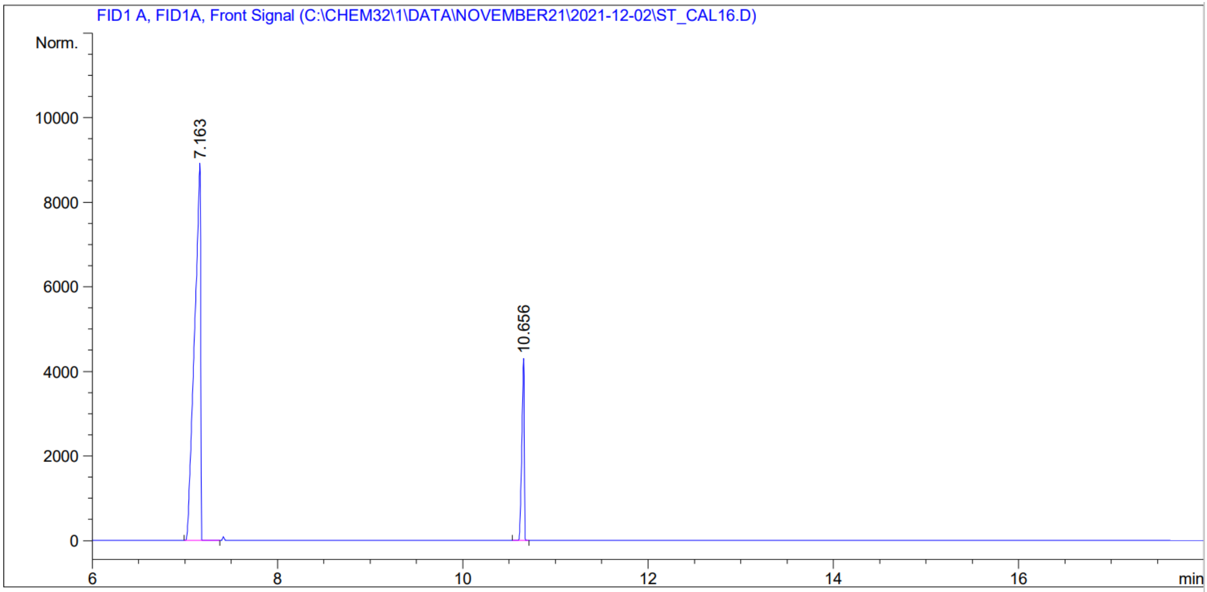

Retention Time	Sample	Peak Area
7.163	*m*-Xylene, **10**	40651.0
10.656	Dodecane	7618.8

1 mmol

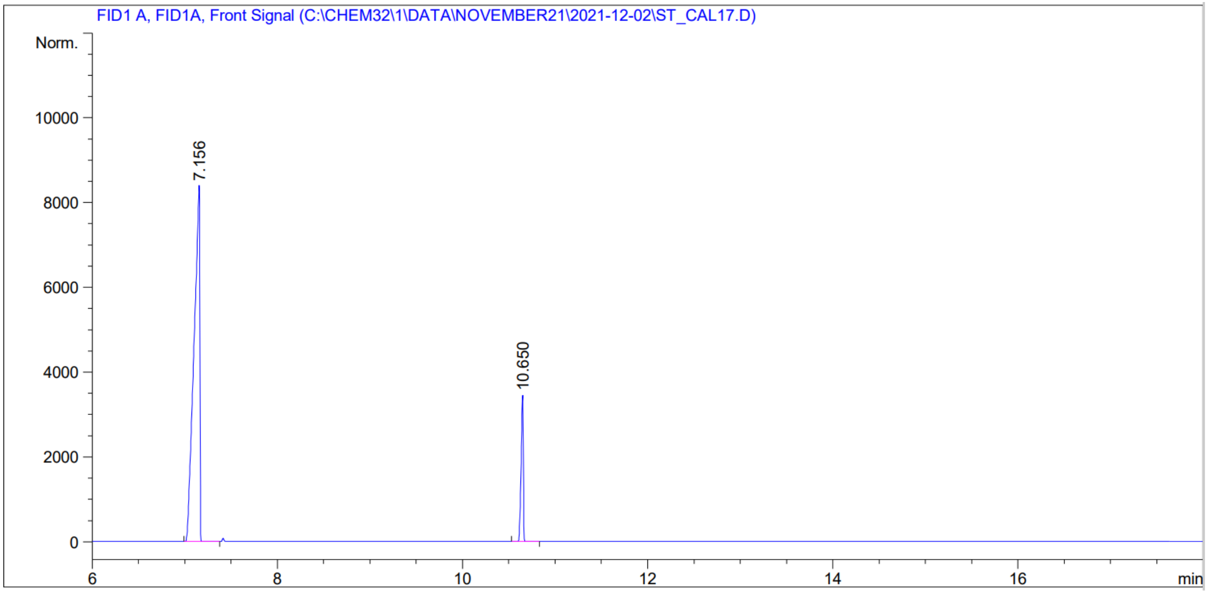

Retention Time	Sample	Peak Area
7.156	*m*-Xylene, **10**	36894.7
10.650	Dodecane	5652.2

NMR Spectra

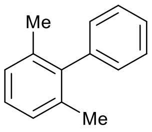

8

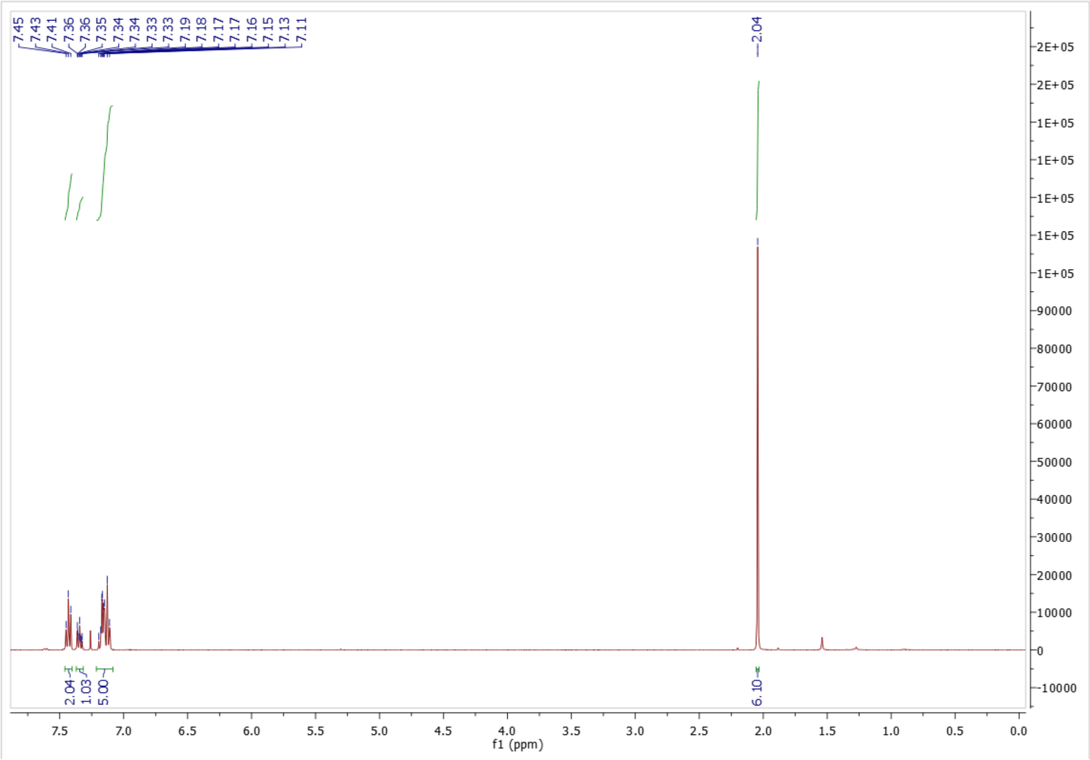

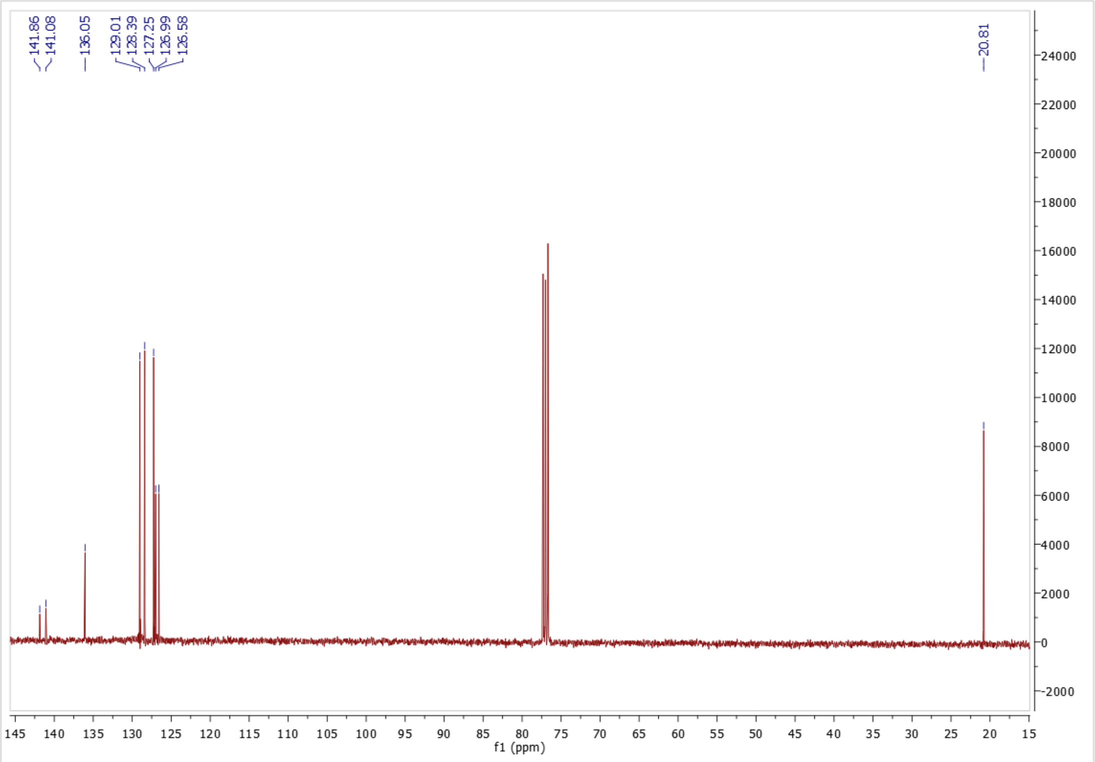

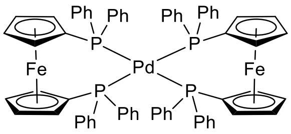

Ph
Ph
Ph
Ph
P
P
Fe
Pd
Fe
P
P
Ph
Ph
Ph
Ph

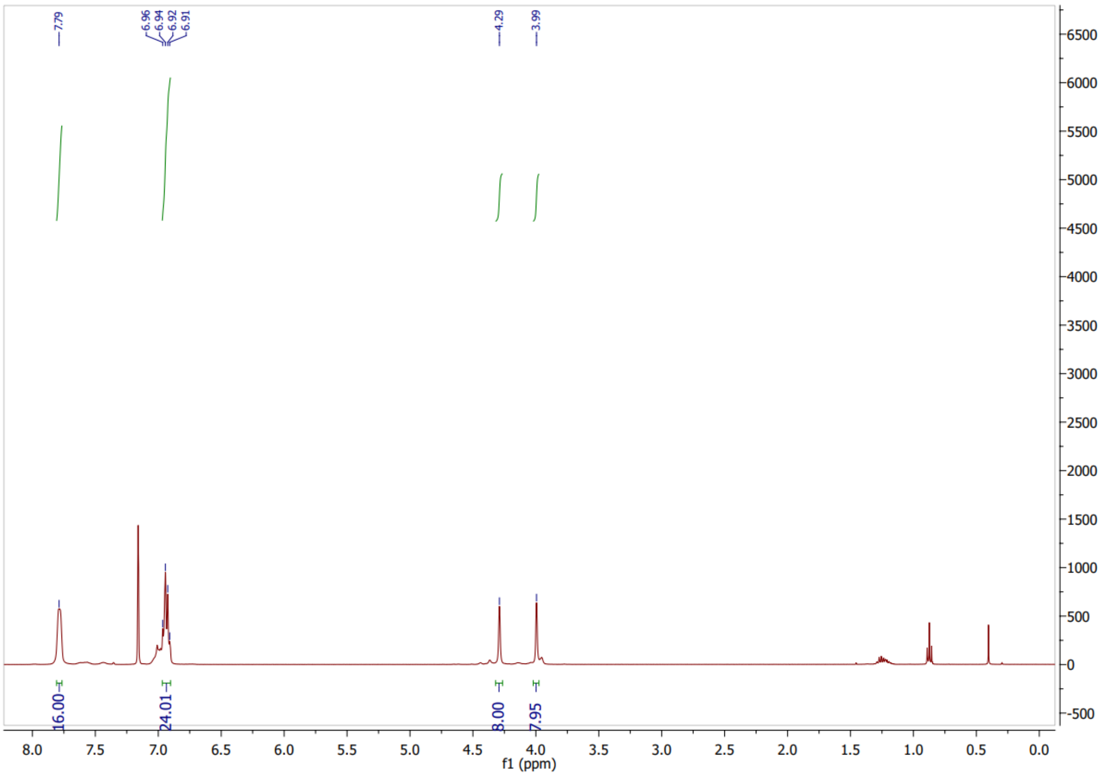

7.79
6.96
6.94
6.92
6.91
4.29
3.99
16.00
24.01
8.00
7.95
f1 (ppm)

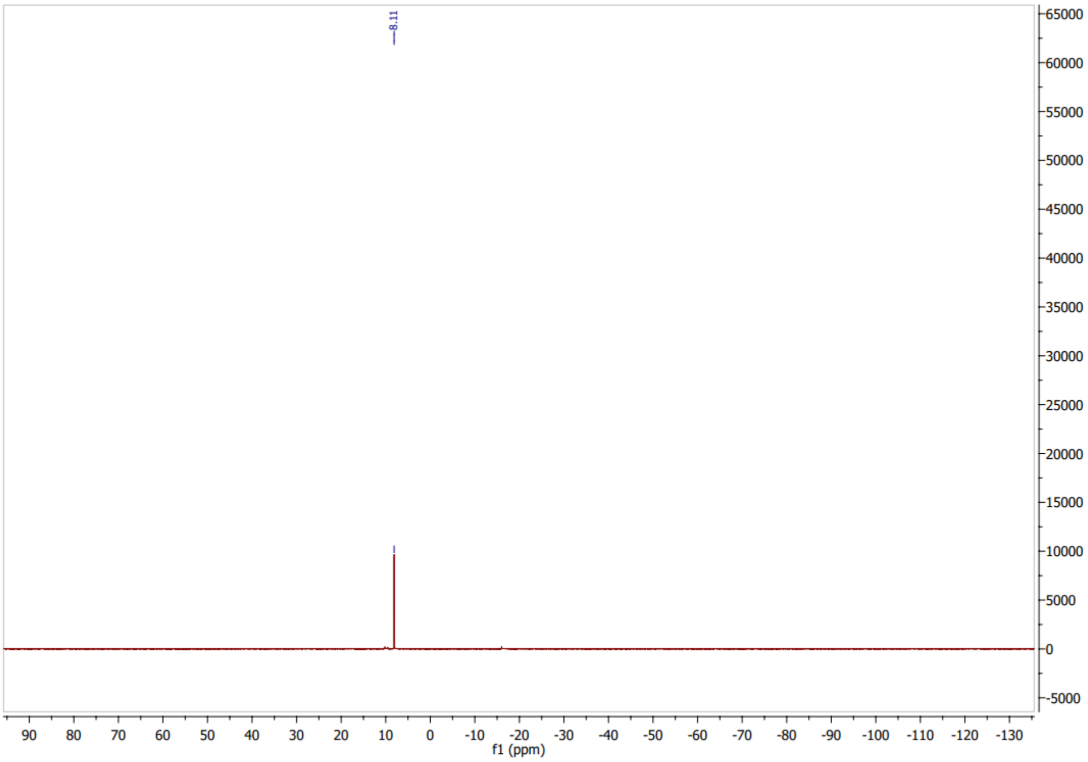

8.11
f1 (ppm)

Pd[P(o-tol)$_3$]$_2$

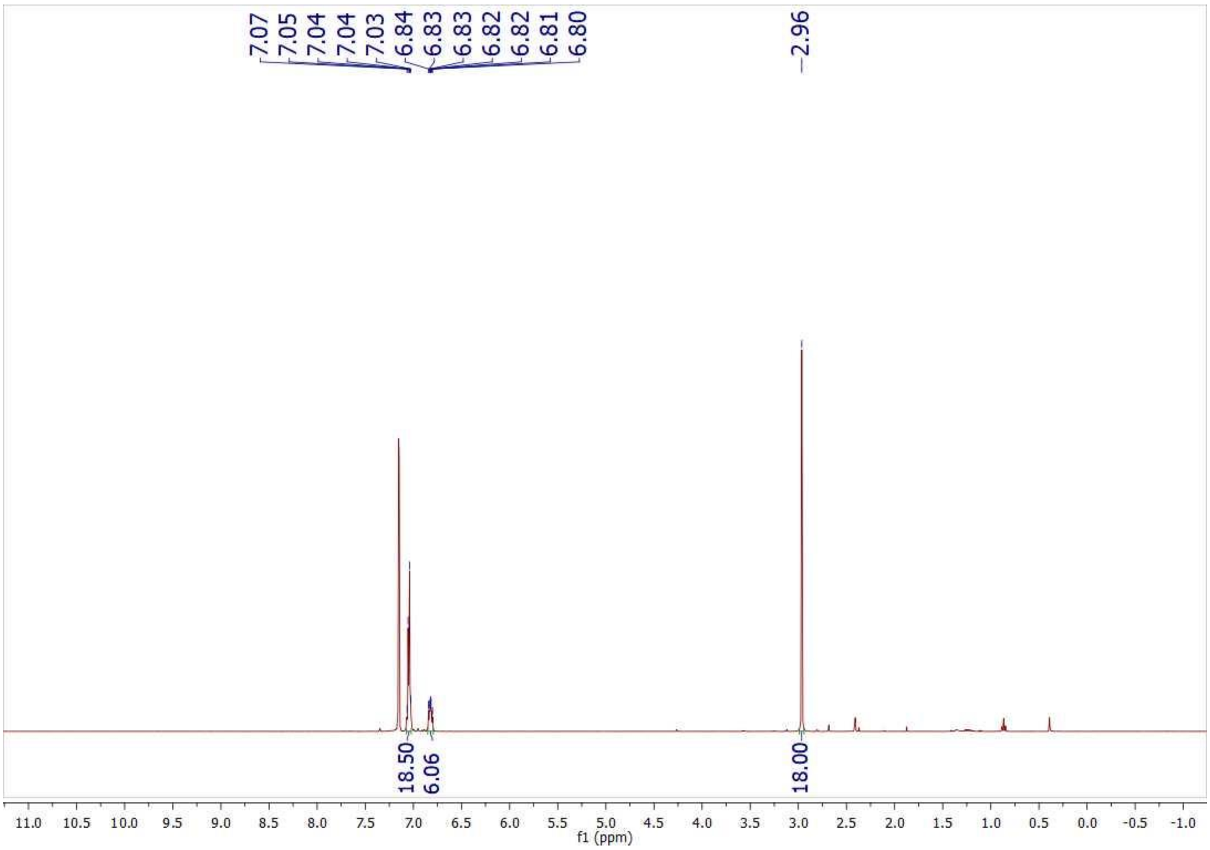

7.07
7.05
7.04
7.04
7.03
6.84
6.83
6.83
6.82
6.82
6.81
6.80
2.96
18.50
6.06
18.00
11.0 10.5 10.0 9.5 9.0 8.5 8.0 7.5 7.0 6.5 6.0 5.5 5.0 4.5 4.0 3.5 3.0 2.5 2.0 1.5 1.0 0.5 0.0 -0.5 -1.0
f1 (ppm)

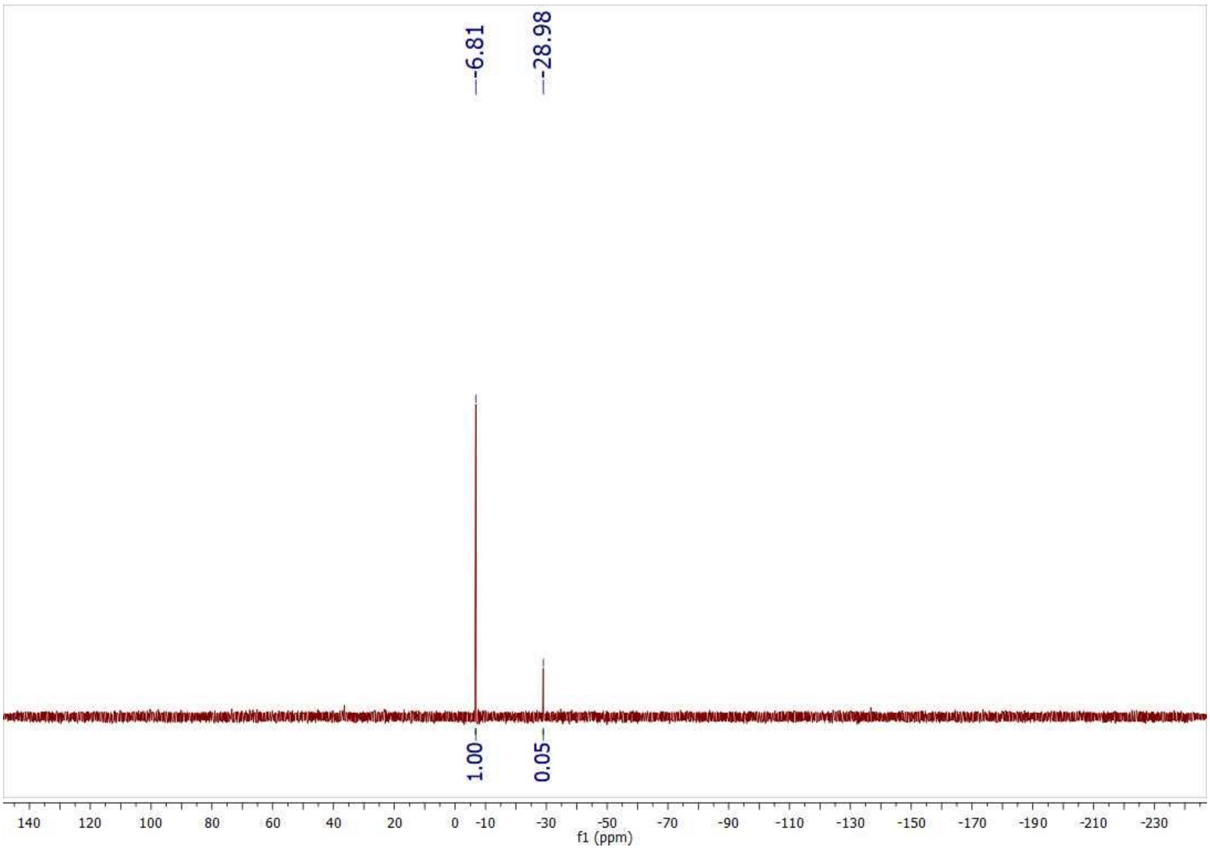

-6.81
-28.98
1.00
0.05
140 120 100 80 60 40 20 0 -10 -30 -50 -70 -90 -110 -130 -150 -170 -190 -210 -230
f1 (ppm)

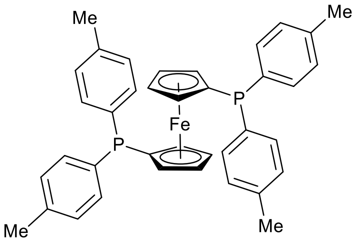
Me
Me
P
Fe
P
Me
Me

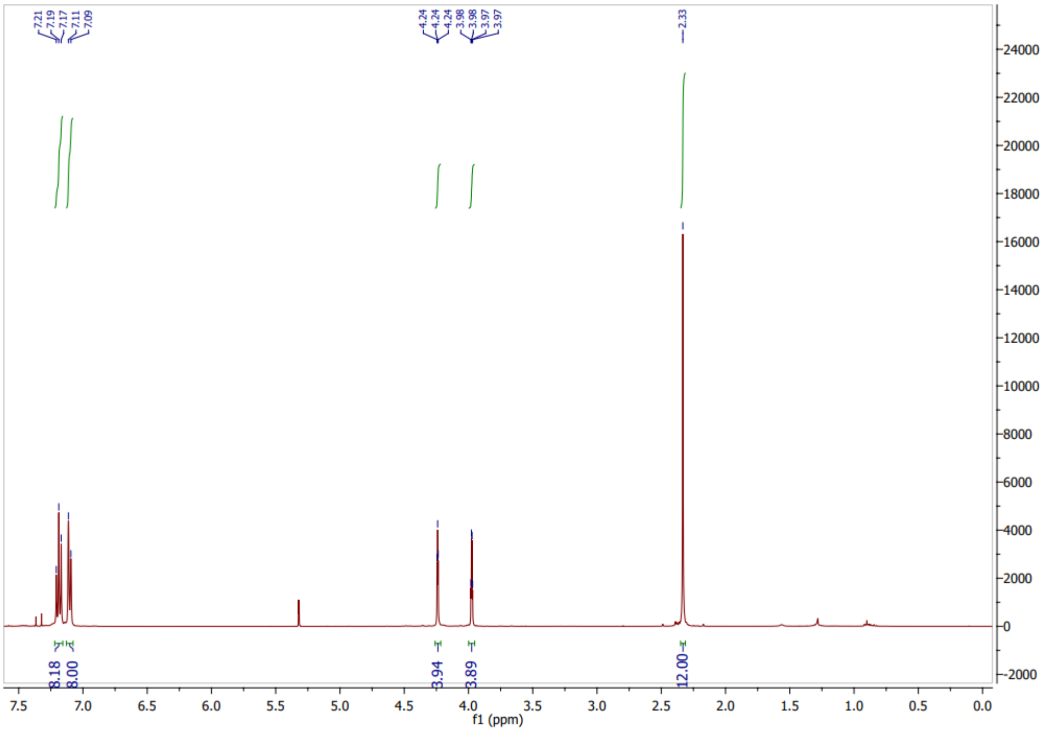
7.21
7.19
7.17
7.11
7.09
4.24
4.24
4.24
3.98
3.98
3.97
3.97
2.33
8.18
8.00
3.94
3.89
12.00
f1 (ppm)

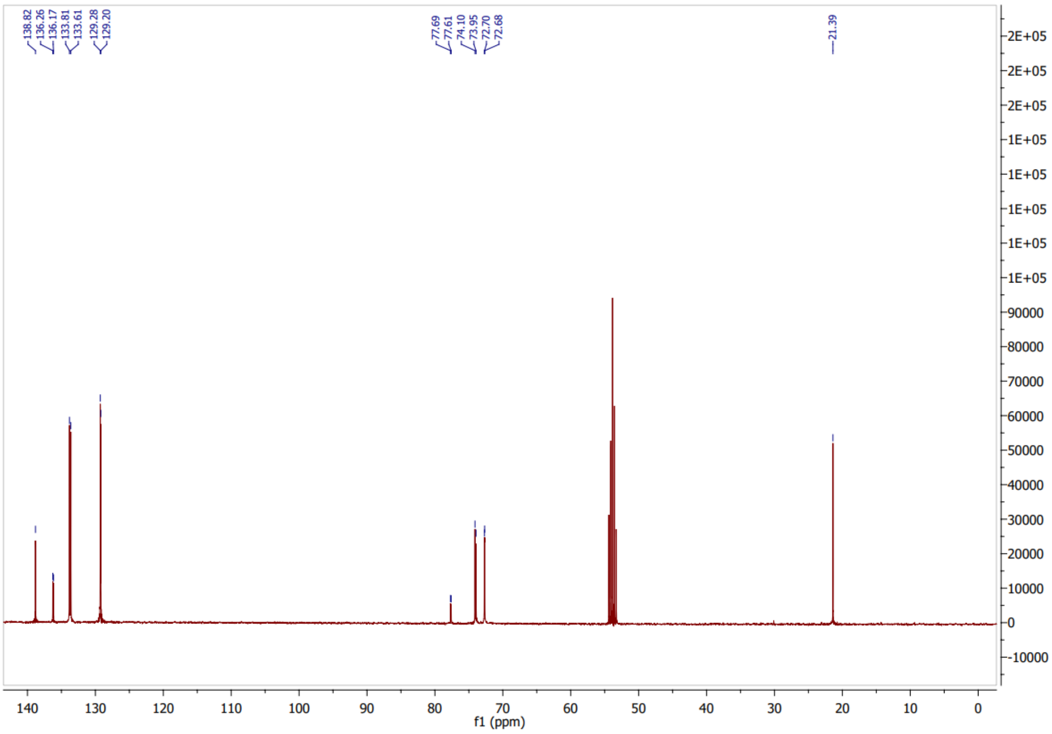
138.82
136.26
136.17
133.81
133.61
129.28
129.20
77.69
77.61
74.10
73.95
72.70
72.68
21.39
f1 (ppm)

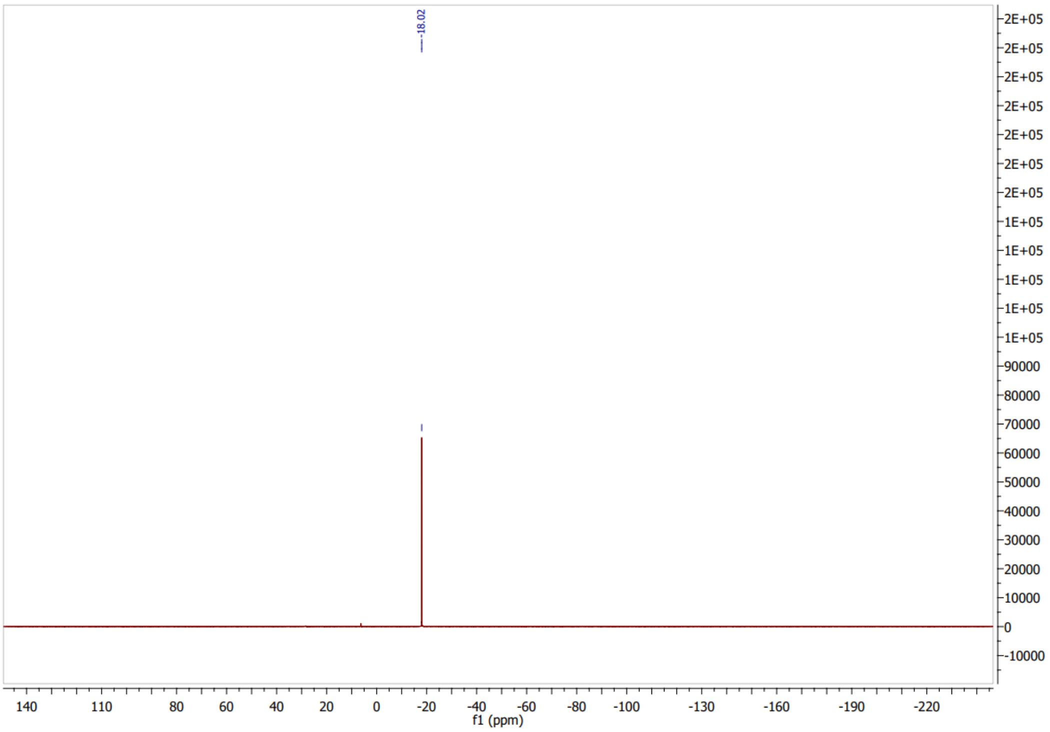
-18.02
f1 (ppm)

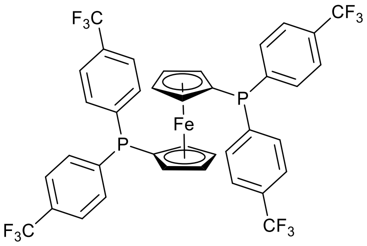
F3C
CF3
P
Fe
P
F3C
CF3

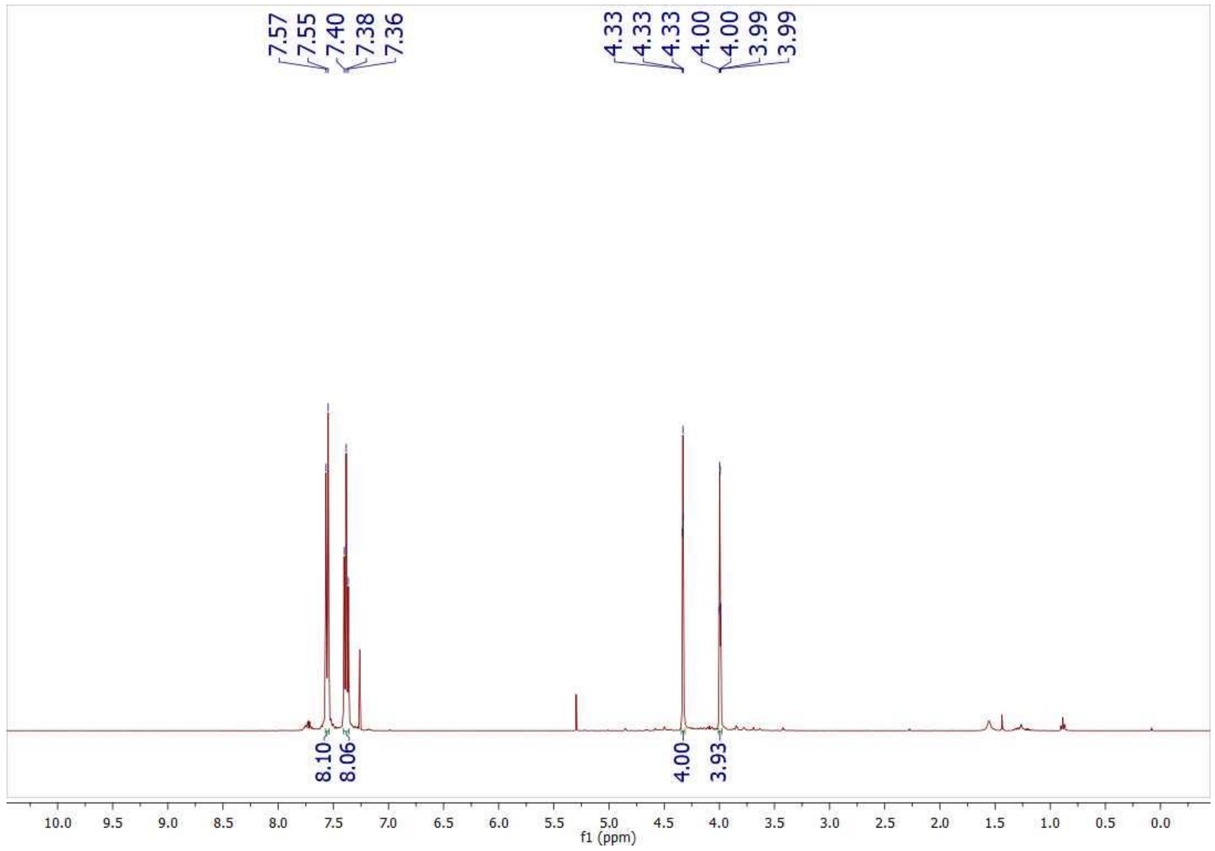
7.57
7.55
7.40
7.38
7.36
4.33
4.33
4.33
4.00
4.00
3.99
3.99
8.10
8.06
4.00
3.93
10.0 9.5 9.0 8.5 8.0 7.5 7.0 6.5 6.0 5.5 5.0 4.5 4.0 3.5 3.0 2.5 2.0 1.5 1.0 0.5 0.0
f1 (ppm)

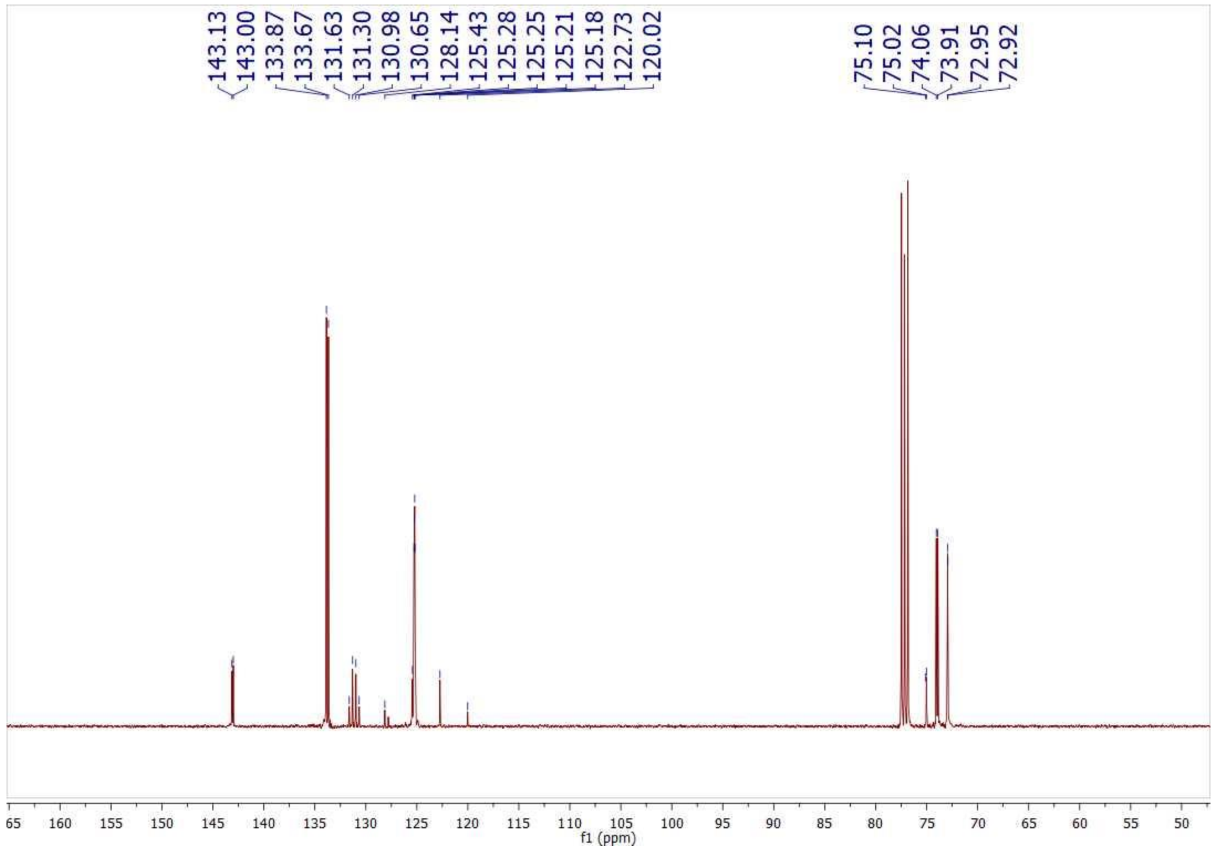
143.13
143.00
133.87
133.67
131.63
131.30
130.98
130.65
128.14
125.43
125.28
125.25
125.21
125.18
122.73
120.02
75.10
75.02
74.06
73.91
72.95
72.92
65 160 155 150 145 140 135 130 125 120 115 110 105 100 95 90 85 80 75 70 65 60 55 50
f1 (ppm)

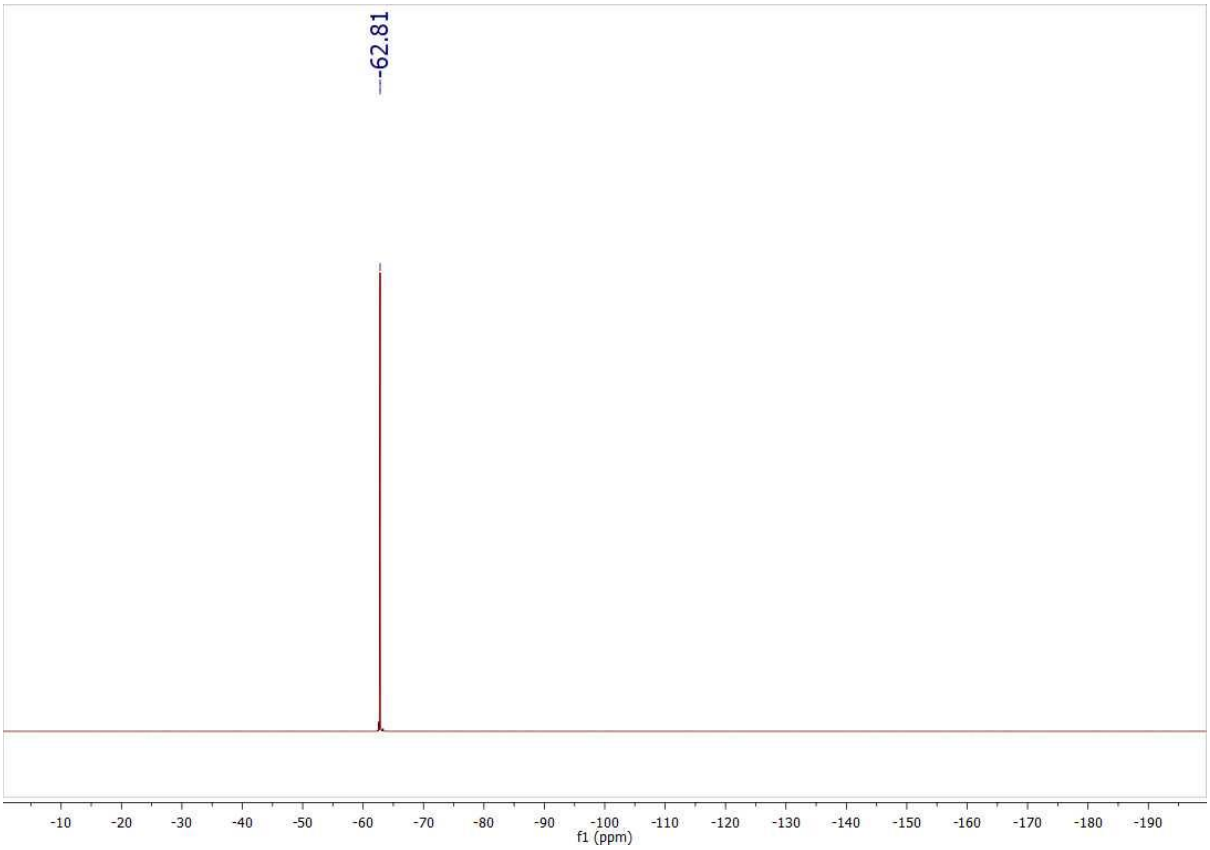

-62.81
-10 -20 -30 -40 -50 -60 -70 -80 -90 -100 -110 -120 -130 -140 -150 -160 -170 -180 -190
f1 (ppm)

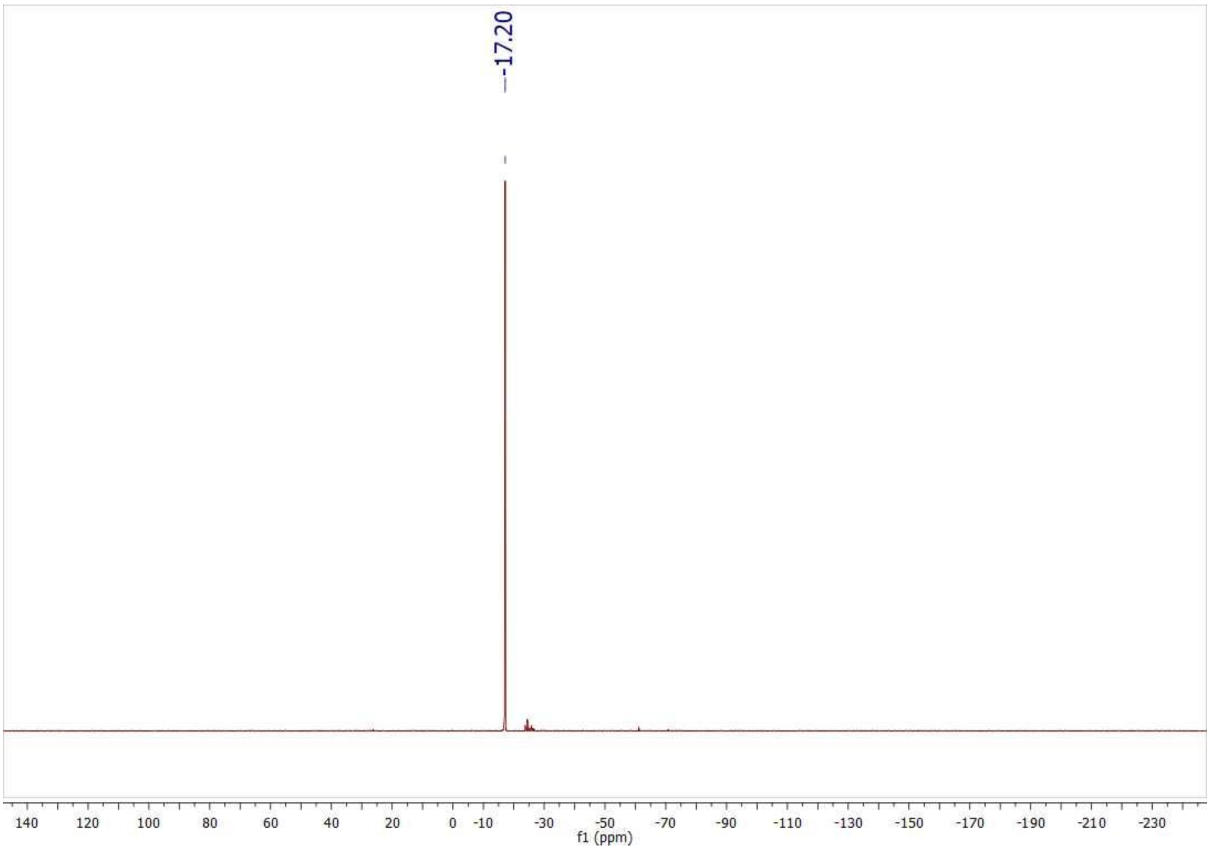

-17.20
140 120 100 80 60 40 20 0 -10 -30 -50 -70 -90 -110 -130 -150 -170 -190 -210 -230
f1 (ppm)

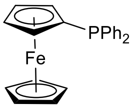
PPh2
Fe

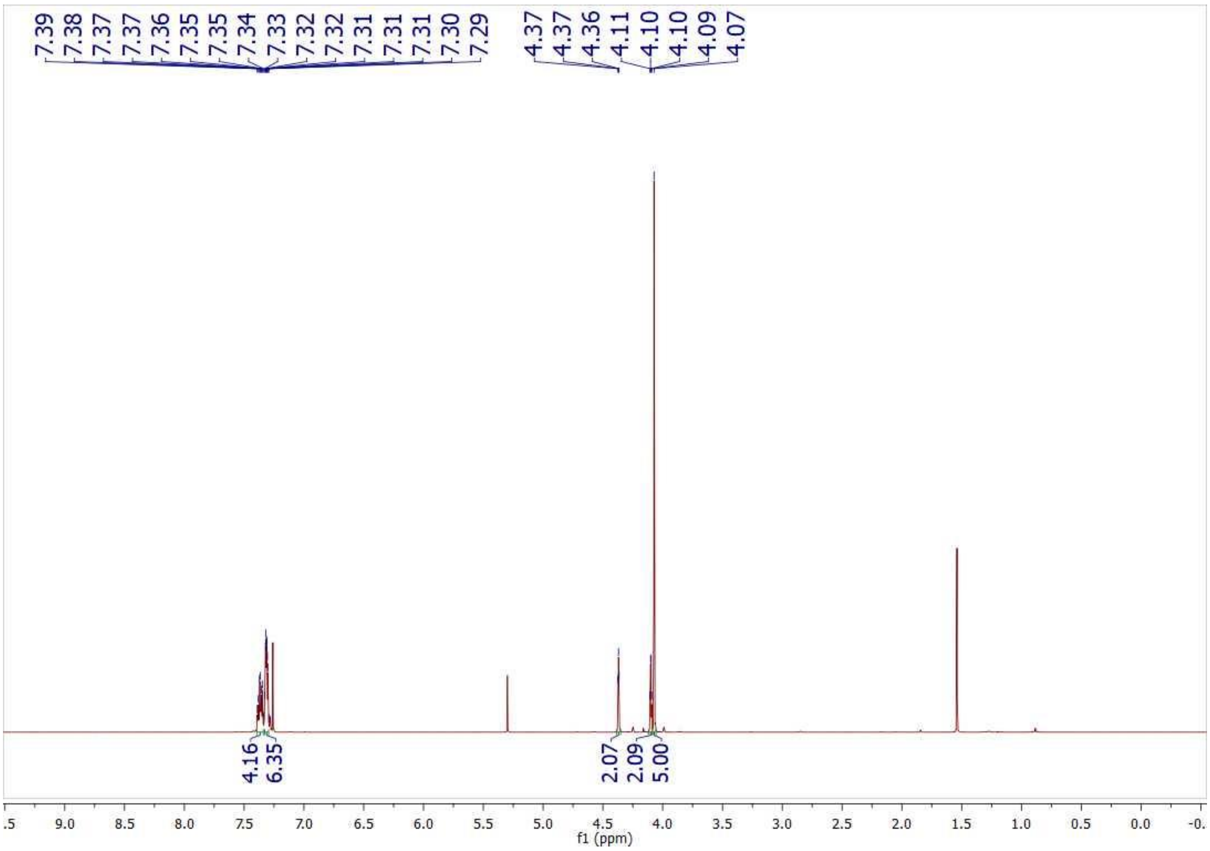
7.39
7.38
7.37
7.37
7.36
7.35
7.35
7.34
7.33
7.32
7.32
7.31
7.31
7.31
7.30
7.29
4.37
4.37
4.36
4.11
4.10
4.10
4.09
4.07
4.16
6.35
2.07
2.09
5.00
f1 (ppm)

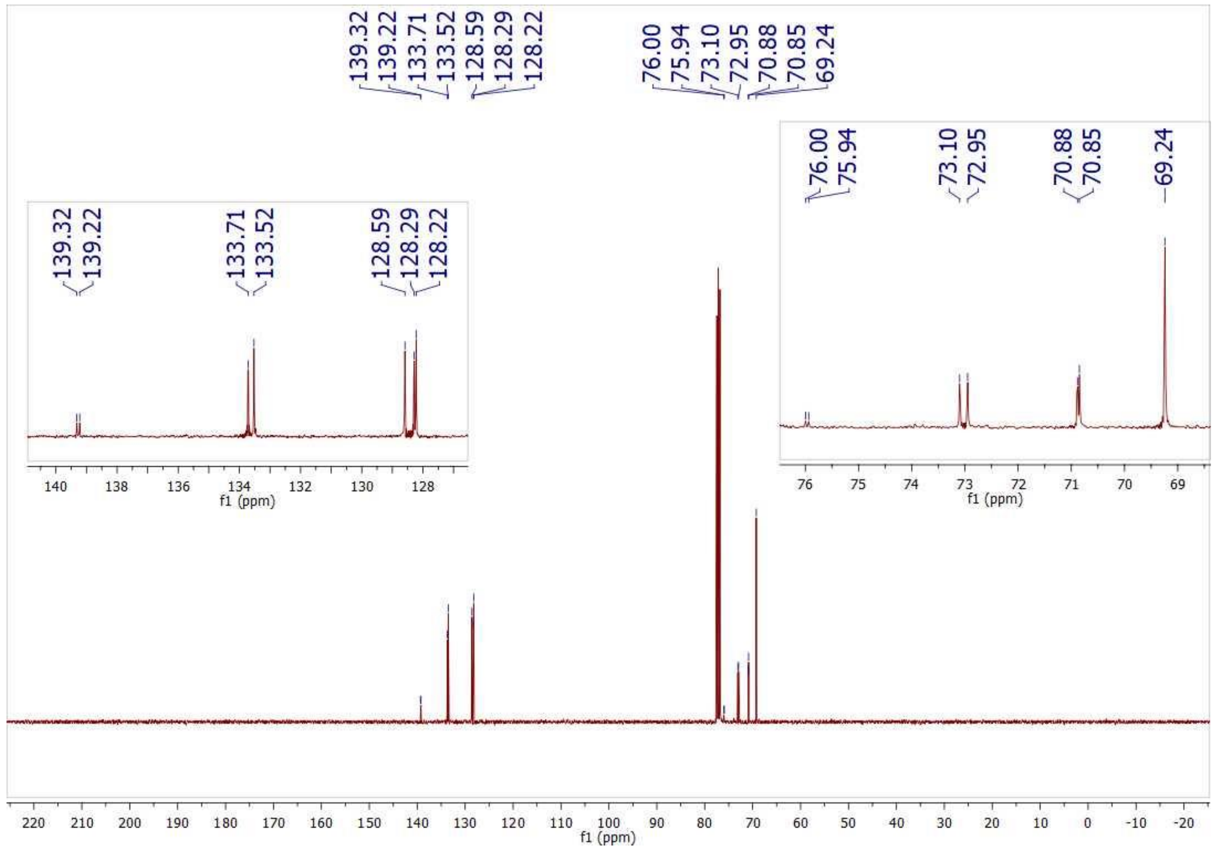
139.32
139.22
133.71
133.52
128.59
128.29
128.22
76.00
75.94
73.10
72.95
70.88
70.85
69.24
220 210 200 190 180 170 160 150 140 130 120 110 100 90 80 70 60 50 40 30 20 10 0 -10 -20
f1 (ppm)

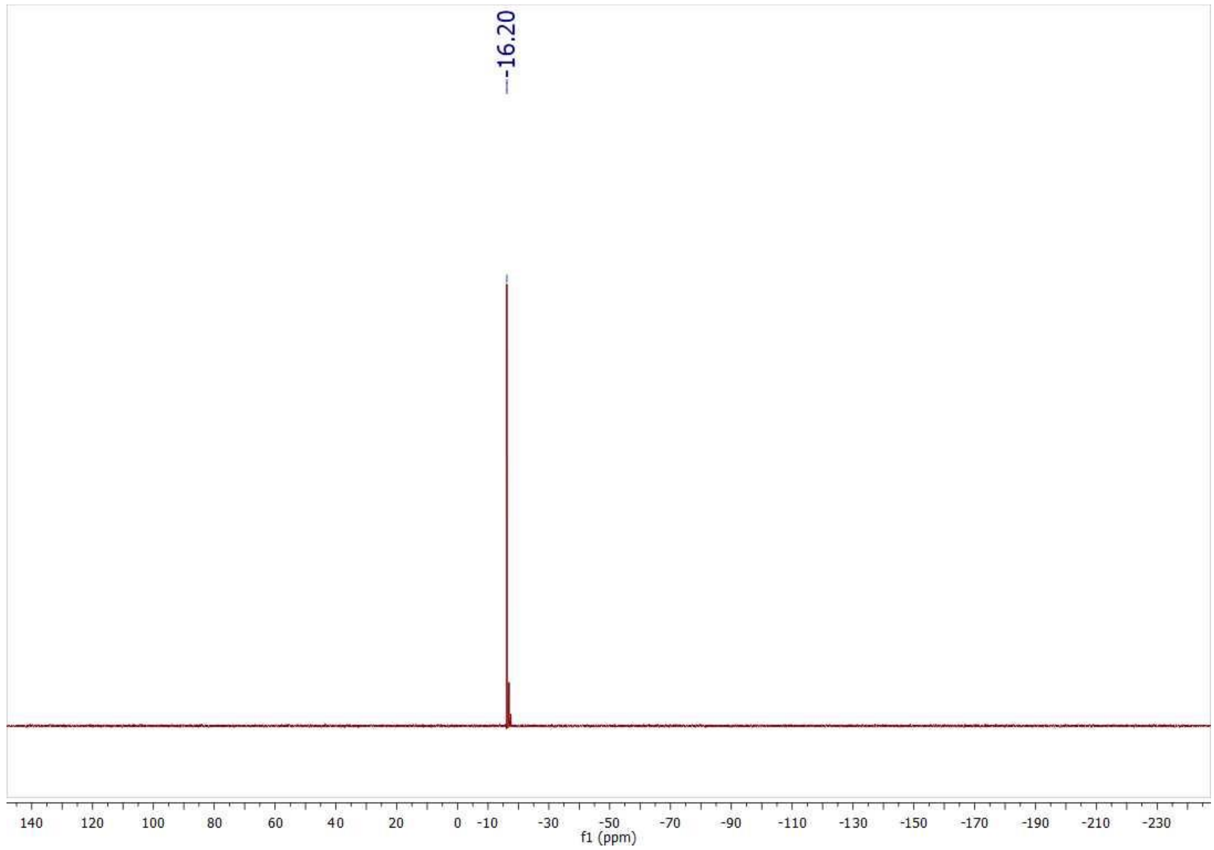
-16.20
140 120 100 80 60 40 20 0 -10 -30 -50 -70 -90 -110 -130 -150 -170 -190 -210 -230
f1 (ppm)

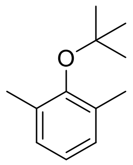

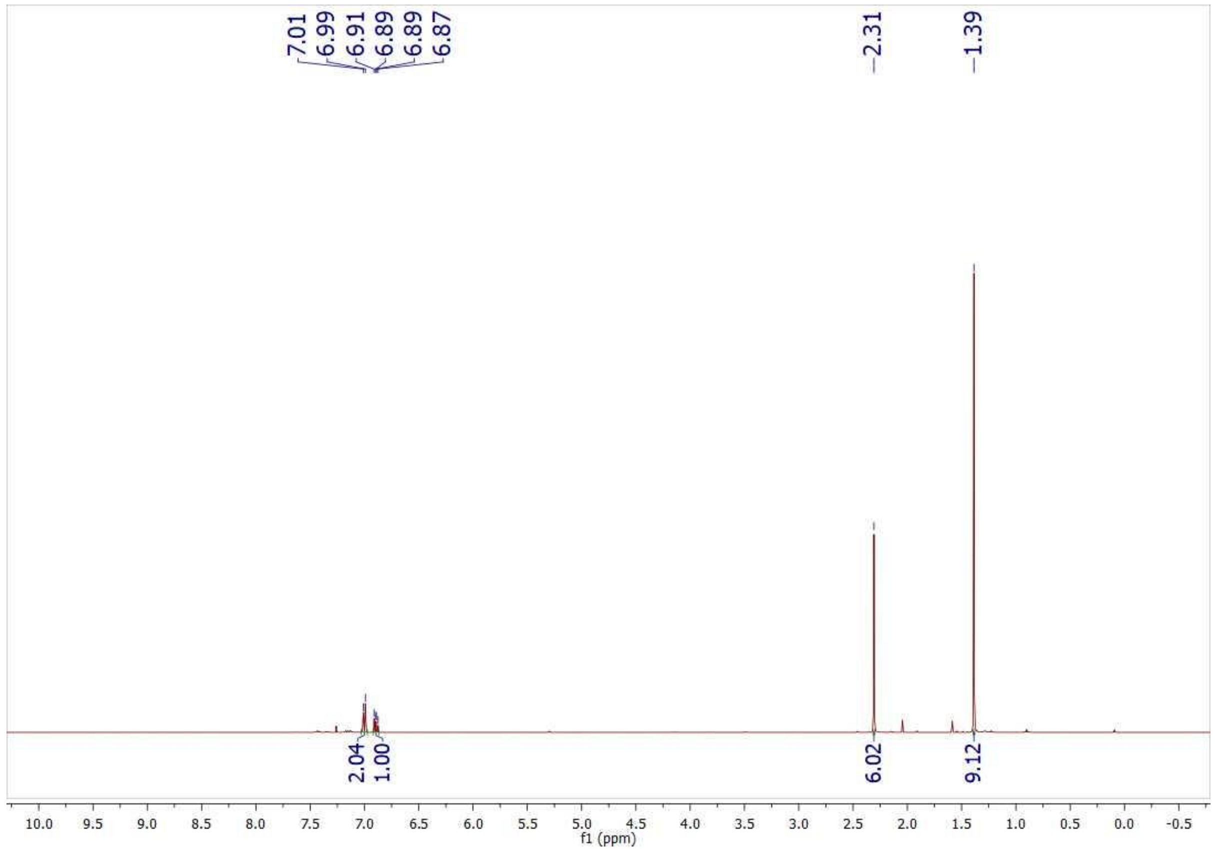
7.01
6.99
6.91
6.89
6.89
6.87
2.31
1.39
2.04
1.00
6.02
9.12
10.0 9.5 9.0 8.5 8.0 7.5 7.0 6.5 6.0 5.5 5.0 4.5 4.0 3.5 3.0 2.5 2.0 1.5 1.0 0.5 0.0 -0.5
f1 (ppm)

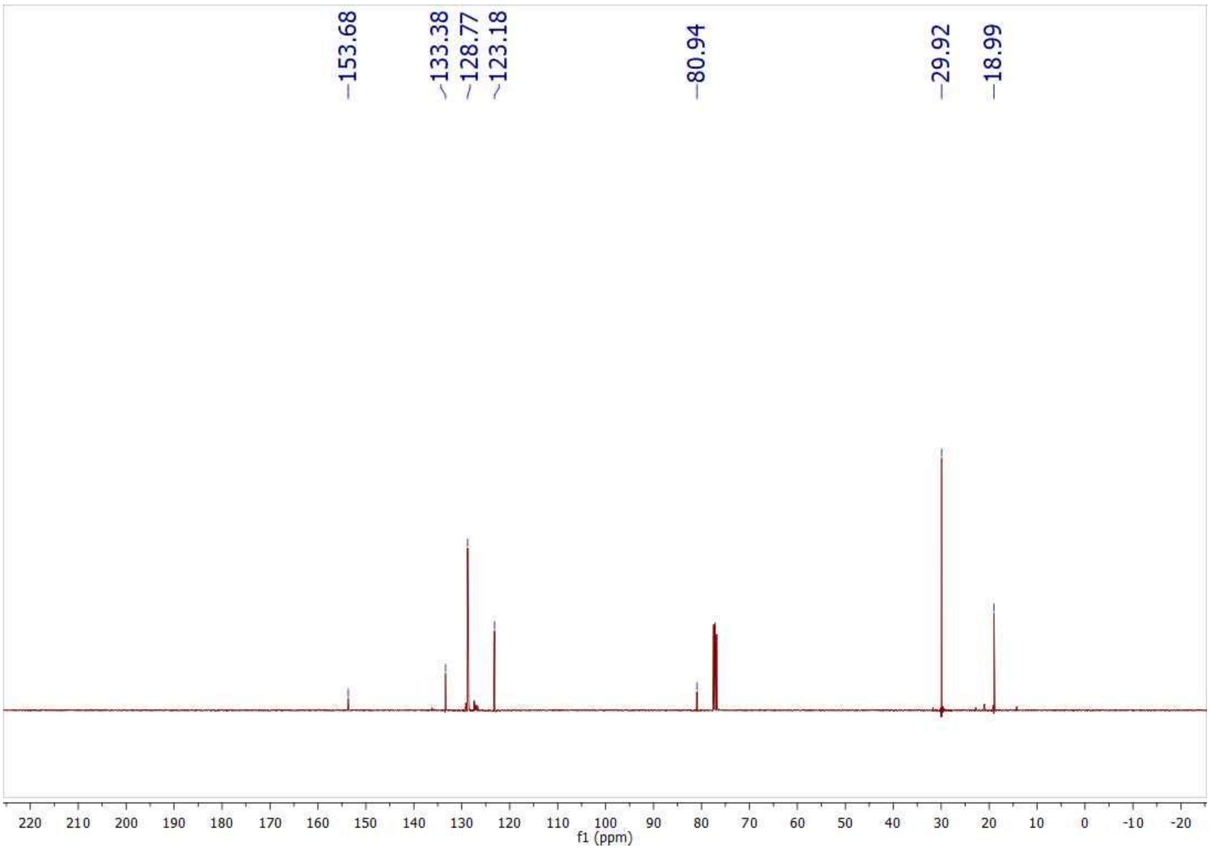

153.68
133.38
128.77
123.18
80.94
29.92
18.99
220 210 200 190 180 170 160 150 140 130 120 110 100 90 80 70 60 50 40 30 20 10 0 -10 -20
f1 (ppm)

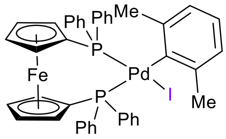

Ph
Ph
Me
P
Fe
Pd
I
P
Me
Ph
Ph

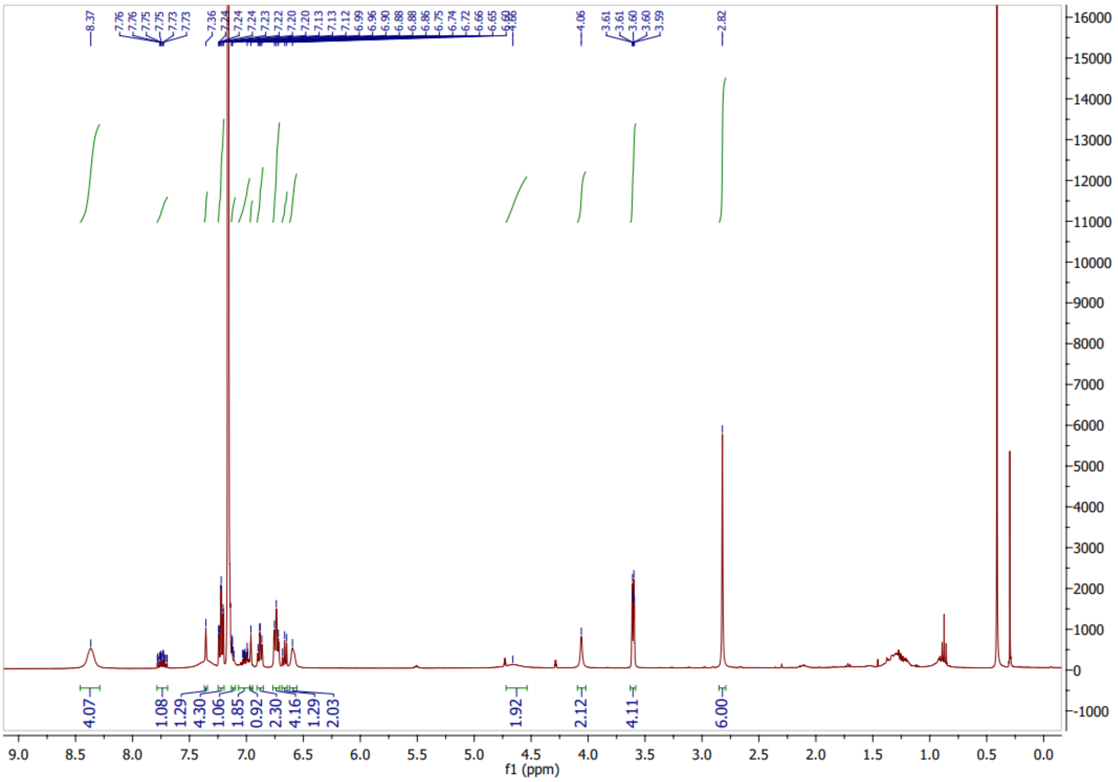

8.37
4.06
2.82
4.07
1.08
1.29
4.30
1.06
1.85
0.92
2.30
4.16
1.29
2.03
1.92
2.12
4.11
6.00
9.0 8.5 8.0 7.5 7.0 6.5 6.0 5.5 5.0 4.5 4.0 3.5 3.0 2.5 2.0 1.5 1.0 0.5 0.0
f1 (ppm)
16000 15000 14000 13000 12000 11000 10000 9000 8000 7000 6000 5000 4000 3000 2000 1000 0 -1000

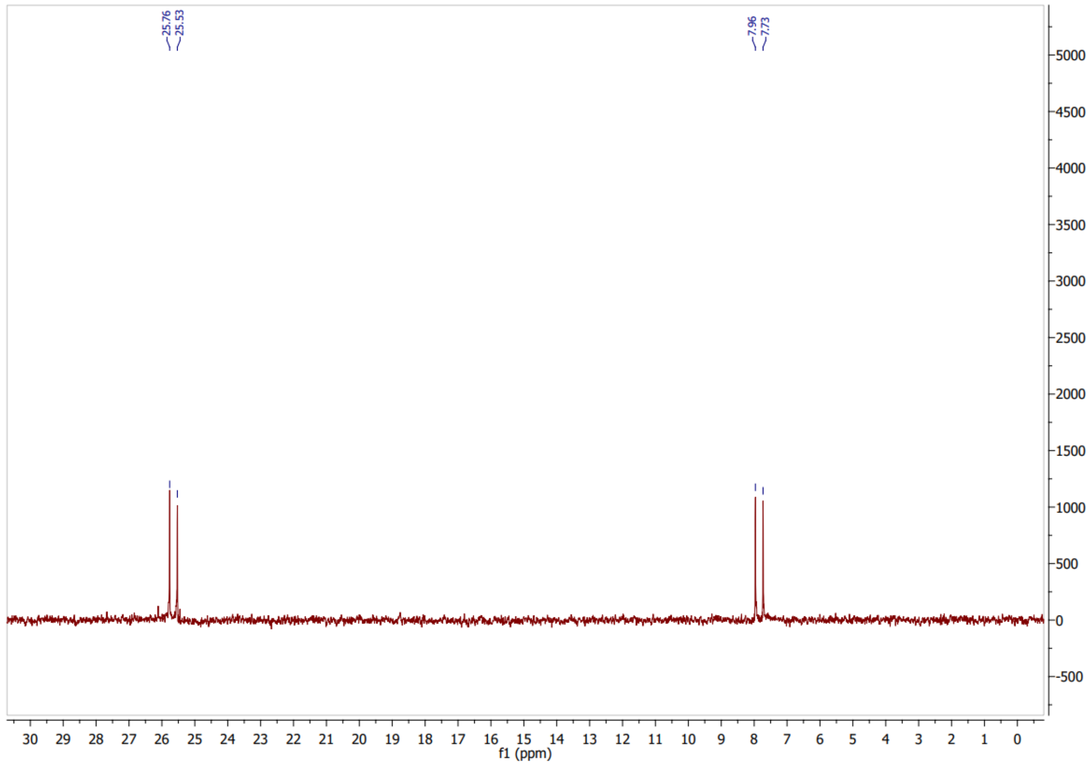

25.76
25.53
7.96
7.73
f1 (ppm)

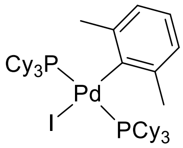

Cy3P
Pd
I
PCy3

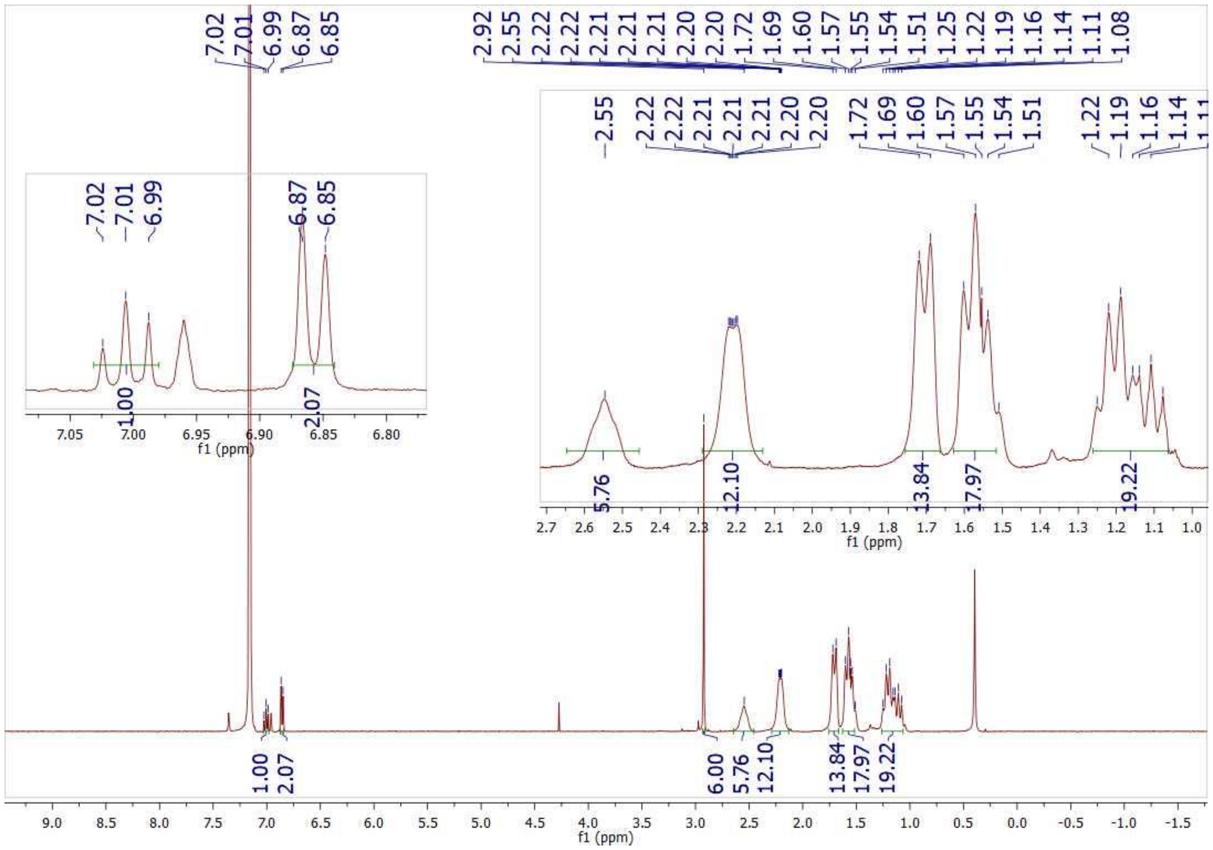

7.02
7.01
6.99
6.87
6.85
2.92
2.55
2.22
2.22
2.21
2.21
2.21
2.20
2.20
1.72
1.69
1.60
1.57
1.55
1.54
1.51
1.25
1.22
1.19
1.16
1.14
1.11
1.08
1.00
2.07
5.76
12.10
13.84
17.97
19.22
6.00
f1 (ppm)

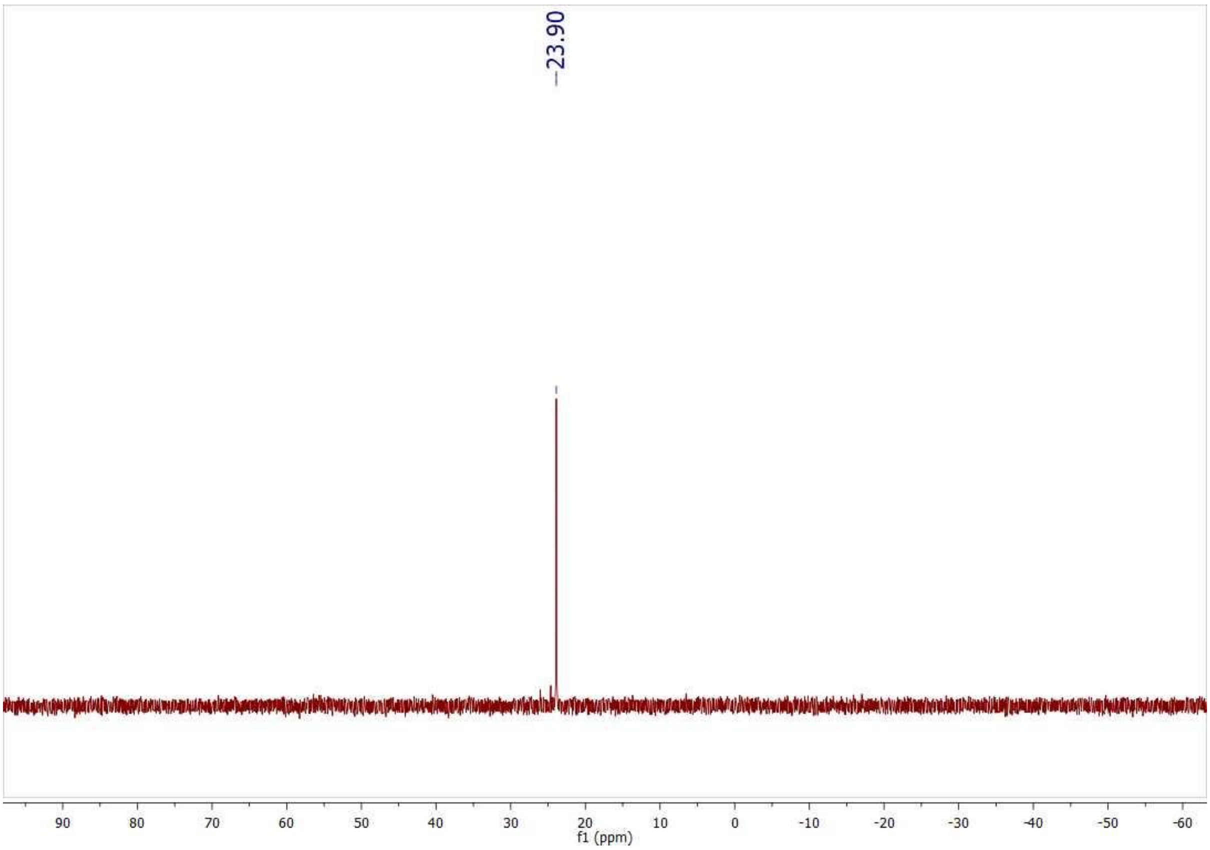

References.

1. Rendón-Nava, D.; Álvarez-Hernández, A.; Rheingold, A. L.; Suárez-Castillo, O. R.; Mendoza-Espinosa, D. Hydroxyl-functionalized triazolylidene-based PEPPSI complexes: metallacycle formation effect on the Suzuki coupling reaction, *Dalton Trans*., **2019**, *48*, 3214–3222.

2. Alcazar-Roman, L. M.; Hartwig, J. F.; Rheingold, A. L. ; Liable-Sands, L. M.; Guzei, I. A. Mechanistic Studies of the Palladium-Catalyzed Amination of Aryl Halides and the Oxidative Addition of Aryl Bromides to $Pd(BINAP)_2$ and $Pd(DPPF)_2$: An Unusual Case of Zero-Order Kinetic Behavior and Product Inhibition, *J. Am. Chem. Soc.*, **2000**, *122*, 4618–4630.

3. Al, N.; Stolley, R. M.; Staudaher, N. D.; Vanderlinden, R. T.; Louie, J. Electronic Effect of Ligands on the Stability of Nickel–Ketene Complexes, *Organometallics*, **2018**, *37*, 3750–3755.

4. Hoff, L. V.; Schnell, S. D.; Tomio, A.; Linden A.; Gademann, K. Cross-Coupling Reactions of Monosubstituted Tetrazines, *Org. Lett.* **2021**, *23*, 5689–5692.

5. Guo, Z.; Li, M. Mou, X-Q., He, G.; Xue, X.-S.; Chen, G. Radical C–H Arylation of Oxazoles with Aryl Iodides: dppf as an Electron-Transfer Mediator for Cs_2CO_3, *Org. Lett.* **2018**, *20*, 1684–1687.

6. Ferguson, D. M.; Bour, J. R.; Canty, A. J.; Kampf, J. W.; Sanford, M. S. Stoichiometric and Catalytic Aryl–Perfluoroalkyl Coupling at Tri-*tert*-butylphosphine Palladium(II) Complexes**,** *J. Am. Chem. Soc.***2017**, *139*, 11662–11665.

7. Paul, F.; Patt, J.; Hartwig, J. F. Structural Characterization and Simple Synthesis of {Pd[P(o-Tol)3]2}. Spectroscopic Study and Structural Characterization of the Dimeric Palladium(II) Complexes

Obtained by Oxidative Addition of Aryl Bromides and Their Reactivity with Amines**,** *Organometallics*, **1995**, *14*, 3030–3039

Crystal Data

Data for $[Pd(dppf)(C_8H_9)I].0.5C_6D_6$ were measured by the National Crystallography Service at the University of Southampton.[1] Data collection and processing used CrysalisPro software.[2] The structures were refined to convergence against F^2 using all independent reflections and the program SHELXL-2018 as implemented within WinGX.[3,4] The model was refined as a twin by a 180 ° rotation about 010 using a hklf 5 formatted reflection file. The contribution of the minor twin component refined to 0.49661(37). Selected crystallographic data and refinement parameters are presented below. Deposition number 2289894 contains the full supplementary crystallographic data for this paper in cif format. These data are provided free of charge by the joint Cambridge Crystallographic Data Centre and Fachinformationszentrum Karlsruhe Access Structures service www.ccdc.cam.ac.uk/structures.

References.

1. Cole, S. J. & Gale, P. A., *Chem. Sci.***,** 2012,**3**, 683-689.
2. *CrysalisPro*. (Rigaku Oxford Diffraction, 2019).
3. Sheldrick, G. M., *Acta Crystallogr. Sect. C Struct. Chem.* (2015). **71**, 3–8.
4. Farrugia, L., J. Appl. Cryst., (2012), **45**, 849-854.

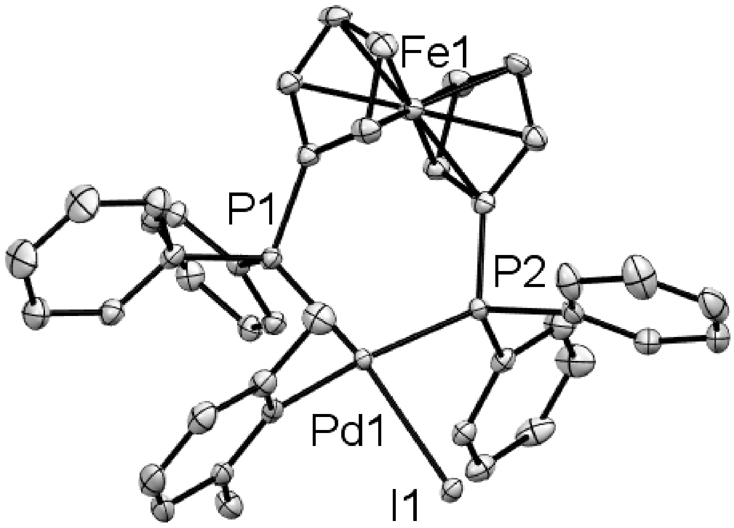

Above. Displacement ellipsoid plot of one of the two crystallographically unique complex molecules present in the asymmetric unit. Ellipsoids are drawn at the 50 % probability level. H atoms omitted for clarity.

Table 1. Selected crystal data and structure refinement parameters.

Empirical formula	$C_{45}H_{37}D_3FeIP_2Pd$
Formula weight	934.88
Temperature	100(2) K
Wavelength	0.71075 Å
Crystal system	Monoclinic

Space group	P 2_1/c	
Unit cell dimensions	a = 19.7987(3) Å	a= 90°.
	b = 18.7977(2) Å	b= 91.5280(10)°.
	c = 20.8830(4) Å	g = 90°.
Volume	7769.3(2) $Å^3$	
Z	8	
Density (calculated)	1.599 Mg/m^3	
Absorption coefficient	1.745 mm^{-1}	
Theta range for data collection	1.769 to 27.512°	
Reflections collected	42803	
Completeness to theta = 27.000°	100.0 %	
Absorption correction	Semi-empirical from equivalents	
Max. and min. transmission	1.00000 and 0.53921	
Data / restraints / parameters	42803 / 0 / 906	
Goodness-of-fit on F^2	1.058	
Final R indices [I>2sigma(I)]	R1 = 0.0322, wR2 = 0.0742	
R indices (all data)	R1 = 0.0447, wR2 = 0.0776	
Largest diff. peak and hole	0.949 and -0.622 e.$Å^{-3}$	

DFT data
General

For conformationally-flexible molecules, xTB 6.4.1 [a]and CREST 2.12 [b] were used to generate conformer libraries, and single-point DFT calculations were used to select the lowest energy conformer and those within 2 kcal/mol for optimisation using DFT calculations.

All DFT calculations were carried out using Gaussian16 Rev. C.01. Geometry optimisations were performed using the MN15-L functional [c] and the def2-svp basis set on all atoms, without symmetry constraints. The nature of each stationary point was confirmed using frequency calculations (zero imaginary frequencies for minima; one imaginary frequency for transition states). Each transition state was checked by either using IRC calculations or by manually perturbing the structure along the reaction coordinate, to ensure that it linked the minima of interest. Energies were refined using single point calculations, using the MN15 functional [d]and the def2-tzvp basis set on all atoms, benzene solvation implemented using the SMD model,[e] and a superfine grid. A correction of 1.89 kcal/mol was added to the free energy of each structure to better reflect a 1 mol/L standard state. [f] All energies are free energies in kcal/mol.

Structures for each compound can be obtained from this ESI file, or from the ioChem-BD [g]database (see the manuscript for a DOI).

Reactions of Methyl Radical with Benzene, *m*-Xylene, and 2-Iodo-*m*-xylene

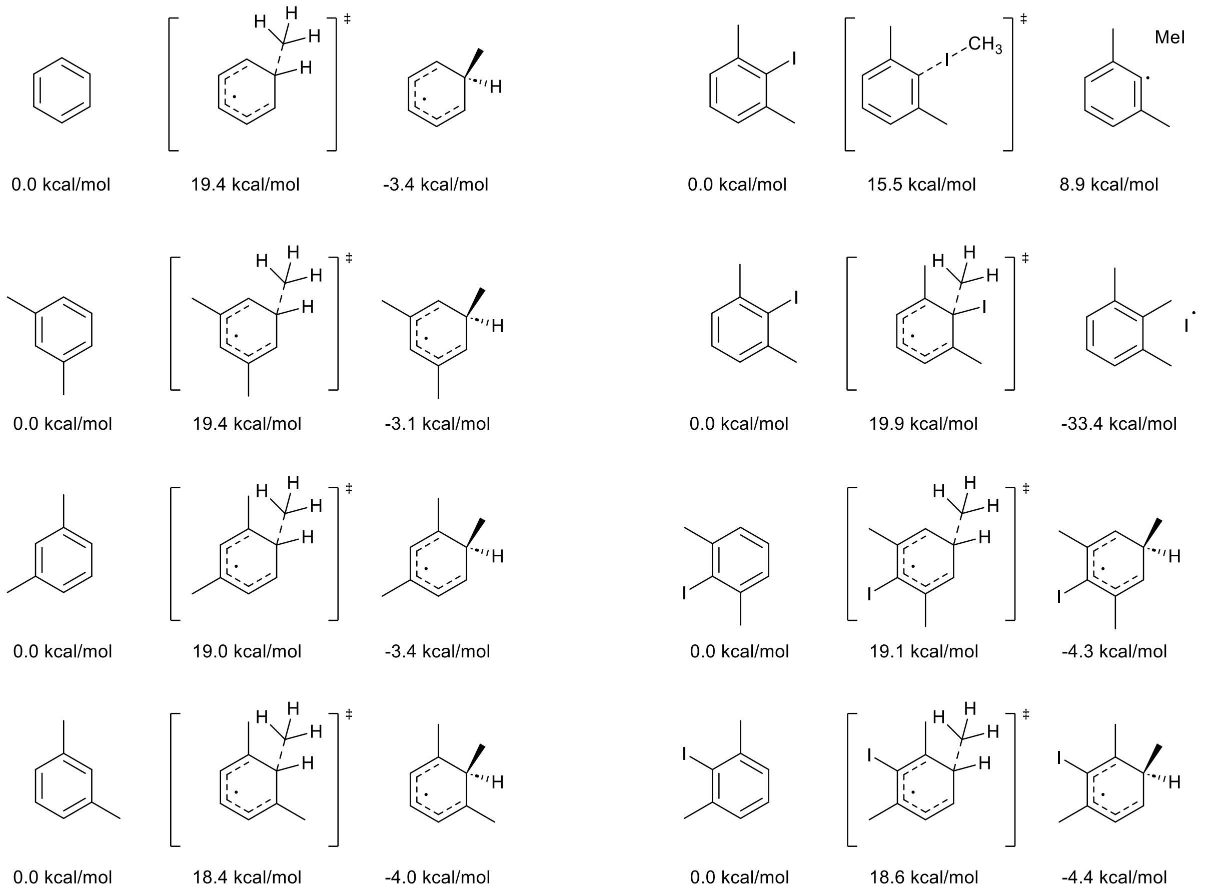

Energies of structures **(in Hartrees)**

	MN15-L/def2-SVP/no solvent			MN15/def2-TZVP/SMD(benzene)
**Structure**	**E**	**H_{corr}**	**G_{corr}**	**E**
Methyl radical	-39.749393	0.033574	0.009520	-39.788861
Benzene	-231.828039	0.106100	0.073263	-232.033654
TS	-271.560397	0.141359	0.102451	-271.808323
Me+Benzene Product	-271.598101	0.144370	0.106075	-271.848215
Xylene	-310.307599	0.164111	0.121712	-310.588054
TS (5-pos)	-350.040119	0.199352	0.151089	-350.362880
product (5-pos)	-350.042471	0.199382	0.151818	-350.364261
TS (4-pos)	-350.042471	0.199382	0.151818	-350.364261
product (4-pos)	-350.081956	0.201775	0.157697	-350.405852
TS (2-pos)	-350.044608	0.199405	0.152483	-350.365900
product (2-pos)	-350.083740	0.202967	0.158016	-350.407056
XyI	-606.4219073	0.155495	0.110806	-606.981160
TS (5-pos)	-646.154894	0.190870	0.140131	-646.756330
product (5-pos)	-646.193240	0.194255	0.144095	-646.797664
TS (4-pos)	-646.156108	0.190724	0.139867	-646.756953
product (4-pos)	-646.194304	0.194069	0.143671	-646.797387
TS (2-pos)	-646.157063	0.190834	0.141316	-646.756362
Product (2-pos)	-646.247742	0.196404	0.144591	-646.844466
TS (I abstraction)	-646.154863	0.191627	0.135559	-646.757518
product (I abstraction)	-646.163594	0.193829	0.132719	-646.765197

Formation and Reactions of Phosphoranyl Radicals **(Free energies in kcal/mol)**

Reactions with phenyl radical

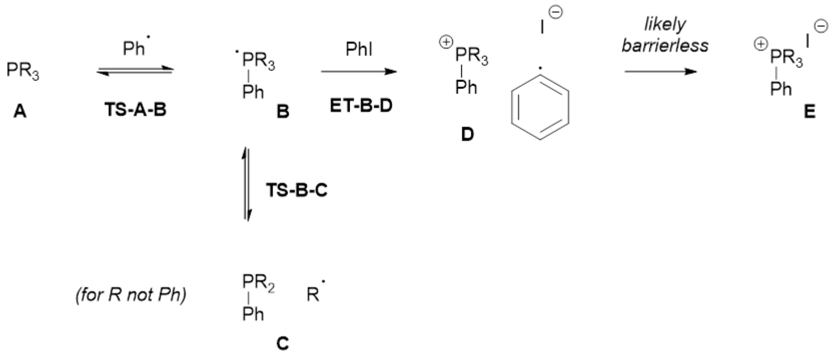

Phosphine:	*dppf*	*PPh_3*	*PCy_3*	*dippf*
**A**	0	0	0	0
**TS-A-B**	14.6	12.9	12.5	15.1
**B**	-3.1	-7.2	-2.0	-5.0
**TS-B-C**			9.5	7.5
**C**			-7.2	
**ET-B-D**	20.6	20.2	21.2	19.6
**D**	20.5	18.3	19.3	14.7
**E**	-5.0			
**ΔG‡ (fragment'n of B)**	17.7	20.1	11.5	12.5
**ΔG‡ (E transfer from B)**	23.7	27.4	23.2	19.6

Reactions with methyl radical

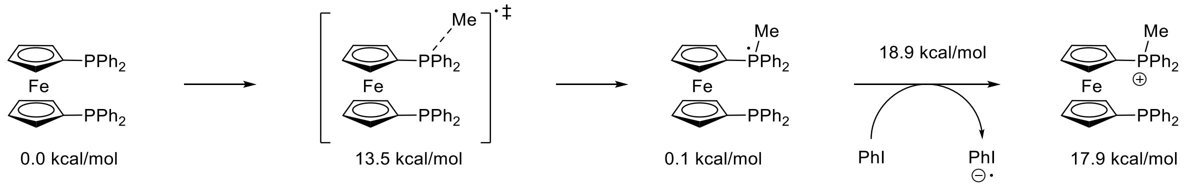

Energies of Structures (in Hartrees)

	MN15-L/def2-SVP /no solvent			MN15/def2-TZVP /SMD(benzene)
**Structure**	**E**	**H_{corr}**	**G_{corr}**	**E**
Iodobenzene	-527.938541	0.097082	0.058546	-528.423460
Iodobenzene radical anion	-527.944827	0.094834	0.050632	-528.478533
Phenyl radical	-231.147647	0.093016	0.059624	-231.347879
Dppf	-3256.085776	0.550009	0.454481	-3257.729135
Dppf + Ph radical TS	-3487.233844	0.644418	0.536537	-3489.073167
Dppf-Ph radical	-3487.273356	0.644999	0.53957	-3489.104388
Dppf-Ph radical (single point as cation)				-3488.999519
Dppf-Ph cation	-3487.141662	0.648956	0.542535	-3489.011716
Dppf-Ph cation (single point as radical)				-3489.098879
$[Dppf\text{-}Ph]^{+}[I]^{-}$	-3784.045094	0.650474	0.536248	-3786.180807
PCy3	-1045.647869	0.502277	0.436298	-1046.402084
PCy3 + Ph radical TS	-1276.795424	0.596035	0.515934	-1277.747068
PhPCy3 radical	-1276.812604	0.597478	0.51879	-1277.772949
PhPCy3 radical (single point as cation)				-1277.669811
PhPCy3 radical TS for Cy loss	-1276.803727	0.595868	0.517297	-1277.753233
PhPCy2 + cyclohexyl radical	-1276.822464	0.596028	0.509977	-1277.772454
PhPCy3 cation	-1276.697508	0.601889	0.524944	-1277.685437
PhPCy3 cation (single point as radical)				-1277.764867
PPh3	-1034.900197	0.291062	0.226992	-1035.576006
PPh3 + Ph radical TS	-1266.050231	0.385169	0.309744	-1266.923507
PPh4 radical	-1266.090225	0.385524	0.310608	-1266.956347
PPh4 radical (single point as cation)				-1266.847712
PPh4 cation	-1265.953466	0.389608	0.316342	-1266.858427
PPh4 cation (single point as radical)				-1266.951064
Dippf	-2804.316929	0.560461	0.474458	-2805.643049
Dippf + Ph radical TS	-3035.464453	0.654206	0.555761	-3036.985541
Dippf-Ph radical	-3035.490952	0.655493	0.557425	-3037.010525
Dippf-Ph radical (single point as cation)				-3036.915375
Dippf-Ph cation	-3035.372083	0.659629	0.562321	-3036.928948
Dippf-Ph cation (single point as radical)				-3037.007692
Dippf-Ph radical TS for propyl loss	-3035.47925	0.65422	0.556827	-3036.998742
Dppf + Me radical TS	-3295.830831	0.585836	0.485608	-3297.515114
Dppf-Me radical	-3295.855694	0.587498	0.488649	-3297.536421
Dppf-Me radical (single point as cation)				-3297.434938
Dppf-Me cation	-3295.729242	0.591443	0.492708	-3297.448890
Dppf-Me cation (single point as radical)				-3297.530667

References

a Bannwarth, C.; Caldeweyher, E.; Ehlert, S.; Hansen, A.; Pracht, P.; Seibert, J.; Spicher, S.; Grimme, S. Extended tight-binding quantum chemistry methods, *WIREs Comput Mol Sci*. **2021**;11:e1493. https://wires.onlinelibrary.wiley.com/doi/10.1002/wcms.1493

b Pracht, P.; Bohle, F.; Grimme, S. Automated exploration of the low-energy chemical space with fast quantum chemical methods *Phys. Chem. Chem. Phys.*, **2020**, *22*, 7169-7192. https://pubs.rsc.org/en/content/articlelanding/2020/CP/C9CP06869D

c Yu, H. S.; He, X.; Truhlar, D. G. MN15-L: A New Local Exchange-Correlation Functional for Kohn–Sham Density Functional Theory with Broad Accuracy for Atoms, Molecules, and Solids, *J. Chem. Theory Comput.* **2016**, *12*, 1280–1293. https://doi.org/10.1021/acs.jctc.5b01082

d Yu, H. S.; He, X.; Li, SD. L.; Truhlar, D. G. MN15: A Kohn–Sham global-hybrid exchange–correlation density functional with broad accuracy for multi-reference and single-reference systems and noncovalent interactions, *Chem. Sci.*, **2016**, *7*, 5032–5051 https://doi.org/10.1039/C6SC00705H

e Marenich, A. V.; Cramer, C. J.; Truhlar, D. G. Universal Solvation Model Based on Solute Electron Density and on a Continuum Model of the Solvent Defined by the Bulk Dielectric Constant and Atomic Surface Tensions, *J. Phys. Chem. B* 2009, 113, 18, 6378–6396. https://doi.org/10.1021/jp810292n

f Harvey, J. M.; Himo, F.; Maseras, F.; Perrin, L. Scope and Challenge of Computational Methods for Studying Mechanism and Reactivity in Homogeneous Catalysis *ACS Catal.* **2019**, *9*, 6803–6813. https://doi.org/10.1021/acscatal.9b01537

g Moreno, M. A.; de Graaf, C.; Lopez, N.; Maseras, F.; Poblet, J. M.; Bo, C. Managing the Computational Chemistry Big Data Problem: The ioChem-BD Platform *J. Chem. Inf. Model.* 2015, 55, 1, 95–103 https://pubs.acs.org/doi/10.1021/ci500593j
